# Iron-Catalyzed Defluorinative Alkylation for Accessing Cyclic Acetal–*gem*-Difluoroalkenes

**DOI:** 10.1021/acs.orglett.6c03239

**Published:** 2026-09-22

**Authors:** Xiao-Tong Wang, Pui Ying Choy, Chi Wai Cheung, Fuk Yee Kwong

**Affiliations:** † State Key Laboratory of Synthetic Chemistry and Department of Chemistry, 26451 The Chinese University of Hong Kong, Shatin, New Territories, Hong Kong, China

## Abstract

Cyclic acetals and *gem*-difluoroalkenes are proven structural motifs in medicinal chemistry, yet their modular incorporation into a densely functionalized hybrid architecture remains an uncharted frontier. Herein, we disclose an Fe-catalyzed reductive defluorinative cross-coupling between trifluoromethyl alkenes and 1,3-dioxolanyl- or 1,3-dioxepanyl-substituted alkyl iodides, enabling the rapid assembly of five- or seven-membered cyclic acetal-embodied fluorinated scaffolds, respectively. This protocol enriches defluorinative coupling versatility and offers streamlined access to structurally diverse pharmaceutically relevant scaffolds.

## Graphical abstract

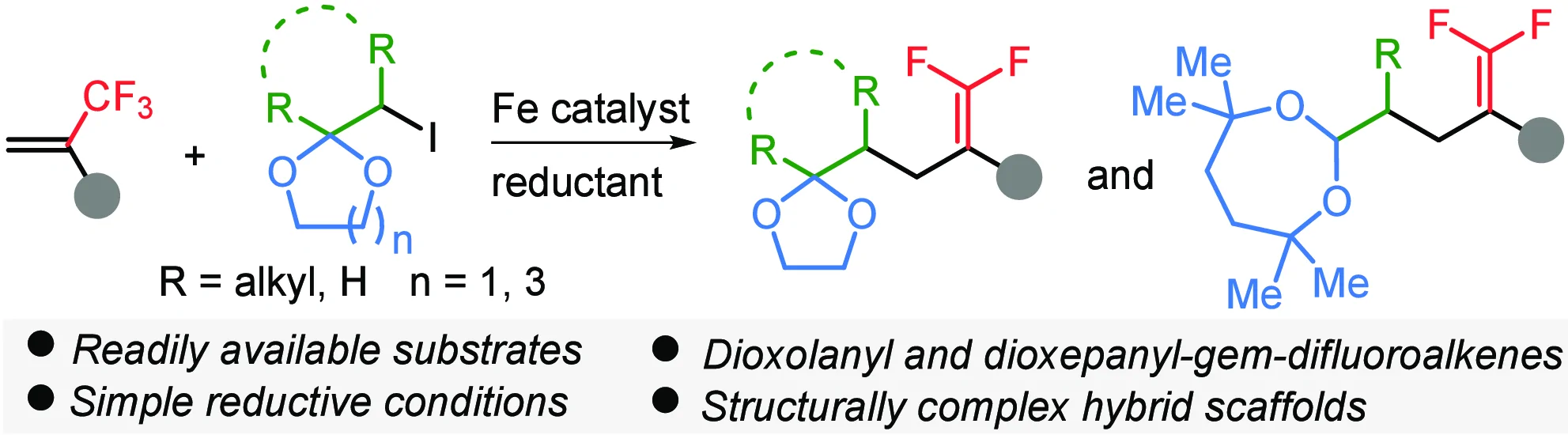

Fluorinated motifs represent a privileged position in contemporary medicinal chemistry, as a subtle shift of fluorine content and placement can profoundly alter lipophilicity, p*K*_a_, metabolic stability, and receptor-binding affinity.^1^ Under this broad class, *gem*-difluoroalkenyl unit **1** has emerged as valuable carbonyl bioisostere (Scheme 1A, top left). This component exhibits key electronic features and a planar geometry that closely resemble those of carbonyl groups while offering enhanced stability and unique orthogonal reactivity.^2^ In parallel, cyclic acetals **2**,^3,4^ such as 1,3-dioxolanes^5^ and 1,3-dioxepanes,^6^ are widely employed as structural motifs in bioactive molecules and pharmaceuticals (Scheme 1A, top right). In particular, 1,3-dioxolanes have also been reported as bioisosteric surrogates for pyrrolidines,^7^ a prominent class of nitrogen-containing frameworks in drug discovery. These cyclic acetals are three-dimensional, conformationally constrained, and aliphatic-rich; consequently, they are capable of modulating molecular conformation, polarity, and lipophilicity, as well as site-selective interactions. Indeed, the complementary attributes of *gem*-difluoroalkenes and cyclic acetals make both classes highly attractive for molecular design, as reflected by numerous bioactive molecules and drug candidates bearing either of these functionalities^4,8^ (Scheme 1A, middle). However, the deliberate fusion of these two moieties into a single compact hybrid scaffold **3** remains underexplored (Scheme 1A, bottom), despite the significant potential of merging their distinctive physicochemical and conformational properties.^9^ It is worth noting that a recently reported dioxolanyl-embedded *gem*-difluoroalkene **4** exhibited promising antifungal activity,^10^ highlighting the rich potential of this hybrid architecture for accessing related bioactive molecules. In addition, the cyclic acetal motif can be retained as a medicinally useful structural feature while also offering an opportunity for downstream diversification.^11^ Thus, cyclic acetal–*gem*-difluoroalkenes are valuable modular hybrid scaffolds for pharmaceutical applications.

**Scheme 1:**
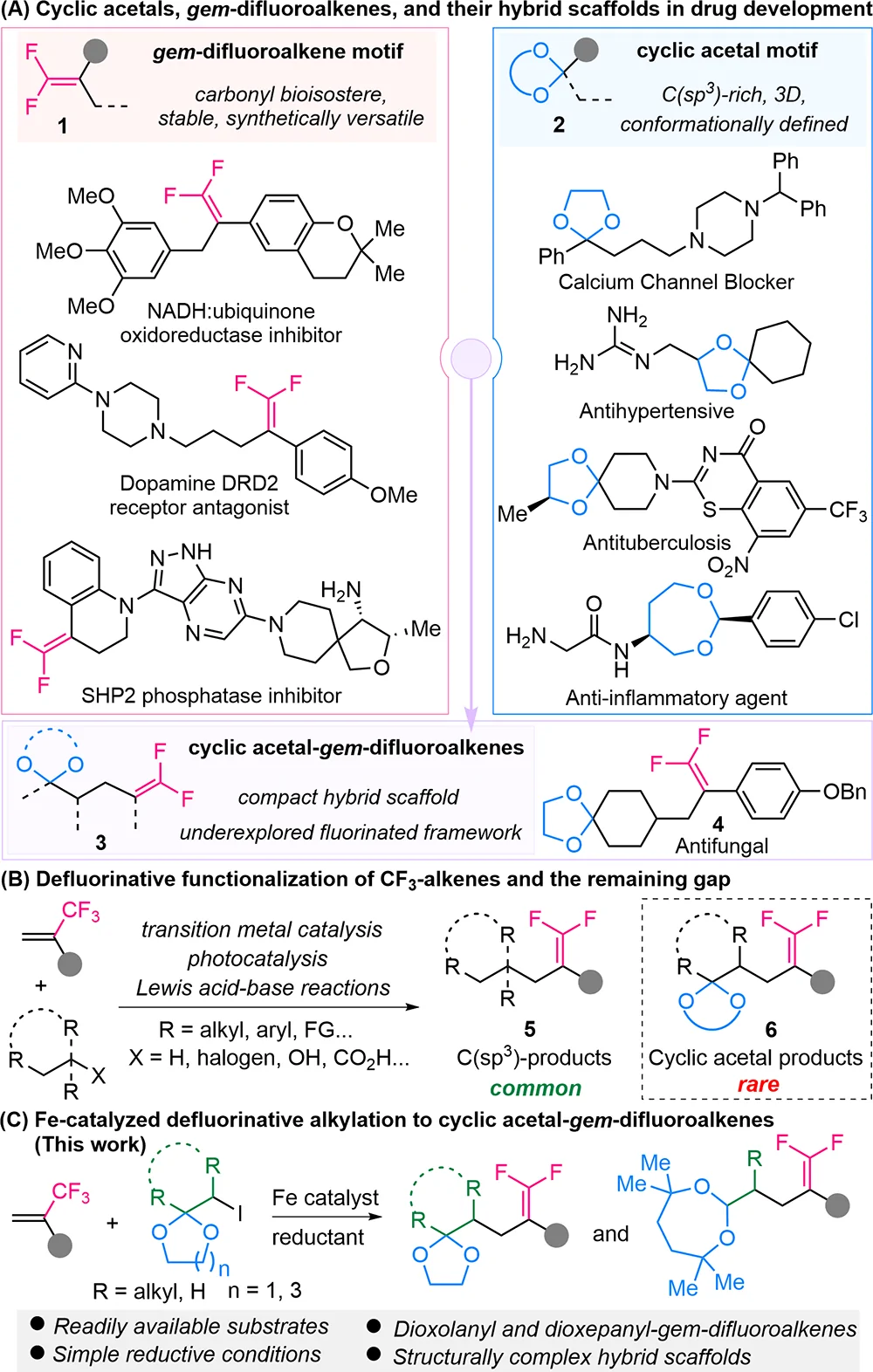
Development of Cyclic Acetals, *gem*-Difluoroalkenes, and Their Hybrid Scaffolds

Defluorinative functionalization of trifluoromethyl-substituted alkenes has emerged as a powerful tool for the construction of *gem*-difluoroalkene frameworks.^12^ Although diverse C­(sp^3^)-based fragments,^13^ particularly those derived from alkyl halides,^14^ have been incorporated to furnish structurally varied alkyl-substituted *gem*-difluoroalkenes **5** (Scheme 1B), the general synthesis of cyclic acetal-containing *gem*-difluoroalkenes **6** remains underdeveloped,^15^ particularly for highly substituted^16^ and spirocyclic^17^ analogues. This limitation stems largely from the scarcity of suitable cyclic acetal-based aliphatic substrates. Indeed, these hybrid scaffolds are rich in C­(sp^3^) character^16,17^ and highly three-dimensional,^18^ which are attributes that are often associated with improved hit identification and favorable lead-like properties. To address this gap, we herein disclose an iron-catalyzed reductive defluorinative coupling of cyclic acetal-substituted alkyl iodides with CF_3_-substituted alkenes (Scheme 1C). Notably, acetals are not necessarily inert and C–O cleavage can take place under reductive conditions.^19^ Accordingly, achieving selective C–I activation while preserving the cyclic acetal represents an additional chemoselectivity challenge. As part of our ongoing interest in organofluorine chemistry,^20^ we report a simple iron catalyst system for the efficient and chemoselective access to a variety of 1,3-dioxolanyl- and 1,3-dioxepanyl-substituted *gem*-difluoroalkenes.

The optimization study commenced by employing CF_3_-substituted vinylbiphenyl **7a** and cyclic acetal-embodied alkyl iodide **8a** as the model substrates (Table S1). A range of solvents were evaluated with FeCl_3_·6H_2_O (30 mol %) as the catalyst, Zn powder (3.0 equiv) as the reductant, and TMSCl (2.0 equiv) as the additive (Table S1, entries 1–7). *N*,*N*-Dimethylacetamide (DMA) was found to be optimal, affording 1,3-dioxolanyl-substituted *gem*-difluoroalkene **9a** in 58% yield (Table S1, entry 7). Subsequent screening of iron salts showed that FeCl_3_·6H_2_O was the most effective among other common Fe­(II) and Fe­(III) salts examined (Table S1, entries 7–9). Control experiments confirmed the essential role of the iron catalyst (Table S1, entry 10). A significant decrease in yield upon reducing the TMSCl loading (Table S1, entry 11) indicated that this additive serves a function beyond merely activating the Zn reductant. Increasing both the stoichiometry of alkyl iodide **8a** and the reaction concentration improved the yield to 70% (Table S1, entries 12 and 13, respectively). Among the metal reductants screened, zinc powder proved to be optimal, manganese powder delivered a lower yield, and iron powder proved to be ineffective (Table S1, entries 14 and 15). Decreasing the temperature to 60 °C further improved the efficiency (Table S1, entry 16). Finally, catalyst tuning identified bipyridine-ligated iron­(III) complex Fe­(bipy)­Cl_3_ as superior, allowing for a slightly reduced catalyst loading and affording product **9a** in 89% yield (Table S1, entries 17–19), thereby establishing the optimal reaction parameters. Notably, **9a** was obtained in high yield with complete retention of the cyclic acetal, and no secondary defluorinative alkylation product was detected, demonstrating selective C–I activation without competing acetal C–O cleavage or further C–F functionalization.

With the optimized conditions in hand, we then investigated the substrate scope of iron-catalyzed defluorinative alkylation with respect to CF_3_-substituted alkenes (Scheme 2A, i). The reaction accommodated a broad range of aryl trifluoromethyl alkenes, tolerating various electronically and structurally diverse aromatic substituents. *para*-Substituted phenyl derivatives bearing electron-donating groups, including phenyl (product **9a**), thioether (product **9b**), benzyloxy (product **9d**), and methoxy (product **9e**) groups, as well as electron-withdrawing groups such as chloro (for product **9c**) and sulfone (product **9i**) groups, were all well tolerated, affording the corresponding 1,3-dioxolanyl-substituted *gem*-difluoroalkenes in moderate to high yields. *ortho*-Methoxy-substituted (product **9f**) and *meta*-methoxy-substituted aryl CF_3_-alkenes (product **9g**) also reacted smoothly, furnishing the desired products in 59% and 71% yields, respectively. Remarkably, functional groups such as thioether (product **9b**), chloro (product **9c**), sulfone (product **9i**), and amino moieties (product **9h**) remained intact, thus serving as useful moieties for further derivatization.^21^ Substrates bearing fused (hetero)­aromatic π-systems, including naphthyl (product **9j**) and dibenzofuryl (product **9k**) derivatives as well as a benzothiophene-containing substrate (product **9l**), also underwent the coupling efficiently. Unfortunately, the quinolinyl substituent gave a relatively low product yield (product **9m**) in the reaction. Benzyl-substituted α-trifluoromethyl alkene **7t** remained unreacted (see page S4 of the Supporting Information for details), possibly because alkyl radical addition would generate a non-resonance-stabilized homobenzylic radical rather than the benzylic radical formed from α-trifluoromethyl styrene.

**Scheme 2:**
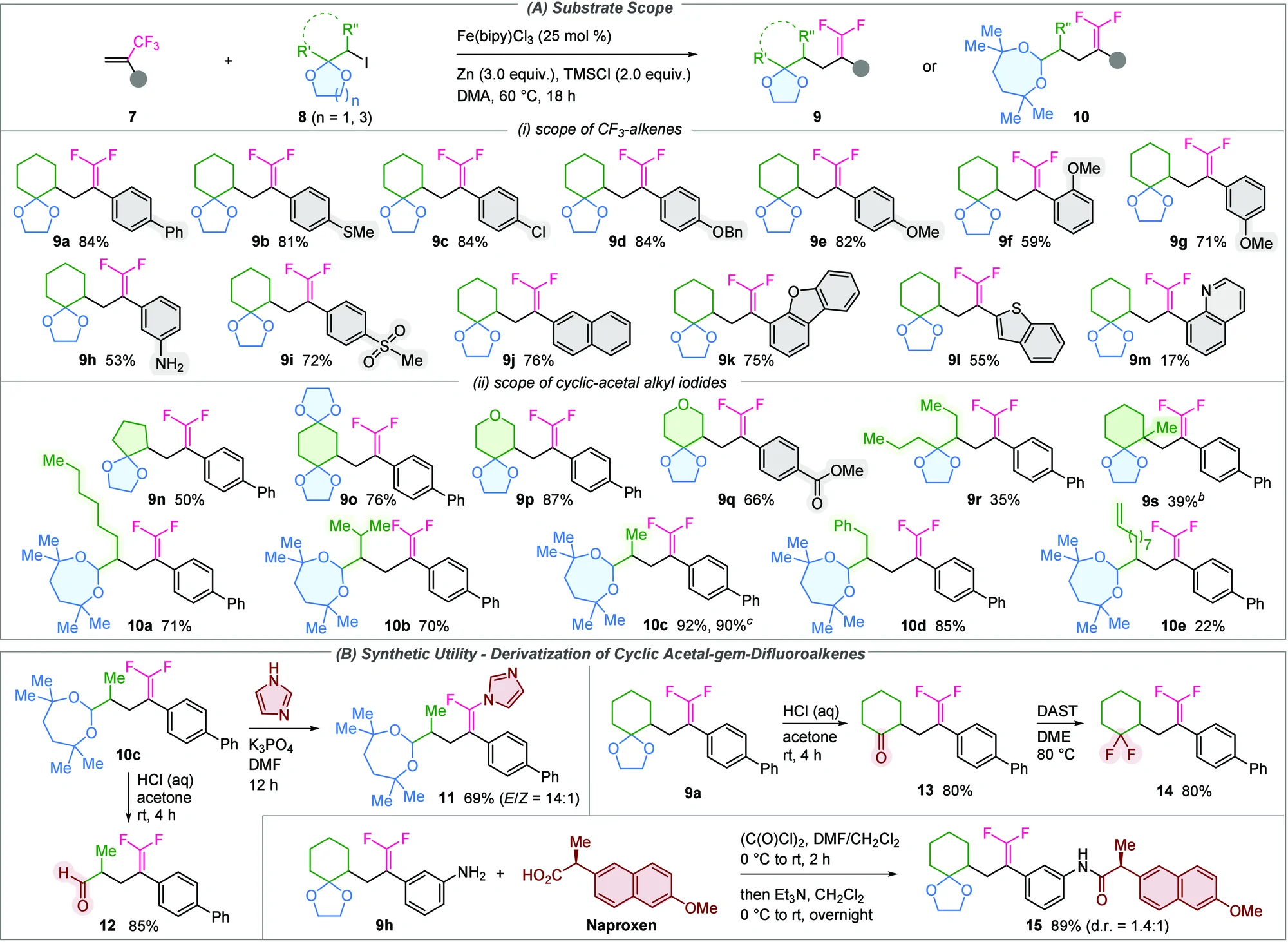
Substrate Scope and Synthetic Utility^a^

a Reaction conditions: CF_3_-alkene **7** (0.10 mmol), cyclic acetal–alkyl iodide **8** (0.25 mmol), Fe­(bipy)­Cl_3_ (25 mol %), Zn (0.30 mmol), TMSCl (0.20 mmol), DMA (1.5 mL), 60 °C, N_2_ atmosphere, 18 h.
b Cyclic acetal–alkyl bromide **8s** was used instead.
c Scale-up experiment (2.5 mmol scale).

The scope of the cyclic acetal-derived alkyl iodide component was next investigated (Scheme 2A, ii). Dioxolanyl-substituted cyclopentyl (product **9n**), bis-dioxolanyl cyclohexyl iodide (product **9o**), and tetrahydropyranyl iodides (products **9p** and **9q**) all reacted smoothly to afford the corresponding *gem*-difluoroalkenes in moderate to good yields. An acyclic dioxolanyl-based 3-heptyl iodide (product **9r**) was also tested. However, a lower product yield was obtained, presumably due to increased steric hindrance. A tertiary alkyl bromide also underwent the coupling smoothly, affording cyclic acetal-containing *gem*-difluoroalkene **9s**. In addition to the dioxolanyl moiety, a variety of 1,3-dioxepanyl-incorporated alkyl iodides were found to be suitable coupling partners. Substrates bearing iodoheptyl (product **10a**), iodoisobutyl (product **10b**), iodoethyl (product **10c**), iodophenethyl (product **10d**), and iododecenyl (product **10e**) groups were examined, and the corresponding dioxepanyl-substituted *gem*-difluoroalkenes were produced effectively. Furthermore, the transformation proved to be readily amenable to scale-up, generating *gem*-difluoroalkene **10c** in comparable yield. The synthetic utility of this transformation was also demonstrated (Scheme 2B and Figure S3). For instance, defluorinative C–N bond formation of **10c** furnished imidazolyl-substituted derivative **11** with high *E* selectivity. Acid hydrolysis of the cyclic acetal units in **10c** and **9a** gave formyl- and ketyl-substituted *gem*-difluoroalkenes **12** and **13**, respectively. Subsequent fluorination of compound **13** afforded *gem*-difluoroalkyl-based compound **14** in 80% yield. Product **9h** also underwent C–N coupling with naproxen to afford amide derivative **15**. These transformations highlight the synthetic versatility of the cyclic acetal–*gem*-difluoroalkenes while maintaining their role as the central scaffold class in this study.

To gain insight into the reaction mechanism, a series of control experiments were conducted. First, the involvement of radical intermediates was probed by adding radical scavenger 1,1-diphenylethylene (DPE) to the model mixture (Scheme 3A). Under these conditions, desired product **9a** was obtained in a slightly diminished yield, together with trapping product **16**, which arise from the addition of dioxolanyl–cyclohexyl radical **Rad-i** to DPE, followed by oxidation. These results support single-electron reduction of the alkyl iodide to generate **Rad-i**, which subsequently participates in the defluorinative alkylation. To further investigate the nature of the active iron species responsible for the reductive coupling, stoichiometric amounts of iron(0) and iron­(II) species, e.g., Fe_2_(CO)_9_ and FeBr_2_, were examined under otherwise identical conditions (Scheme 3B). These iron sources either failed to yield the desired product or produced only trace amounts, suggesting that Fe(0) and Fe­(II) species are unlikely to serve as the direct low-valent intermediates for alkyl iodide reduction. Instead, an Fe­(I) species, which has commonly been proposed as a key reducing intermediate for alkyl halide activation in reductive coupling reactions,^22^ is more likely to be the active species in the present system. Reported redox potentials indicate that reduction of Fe­(bipy)­Cl_3_ ^23^ by Zn^24^ to Fe­(I) is thermodynamically accessible, further supporting its involvement in the reductive activation of alkyl iodide.

**Scheme 3:**
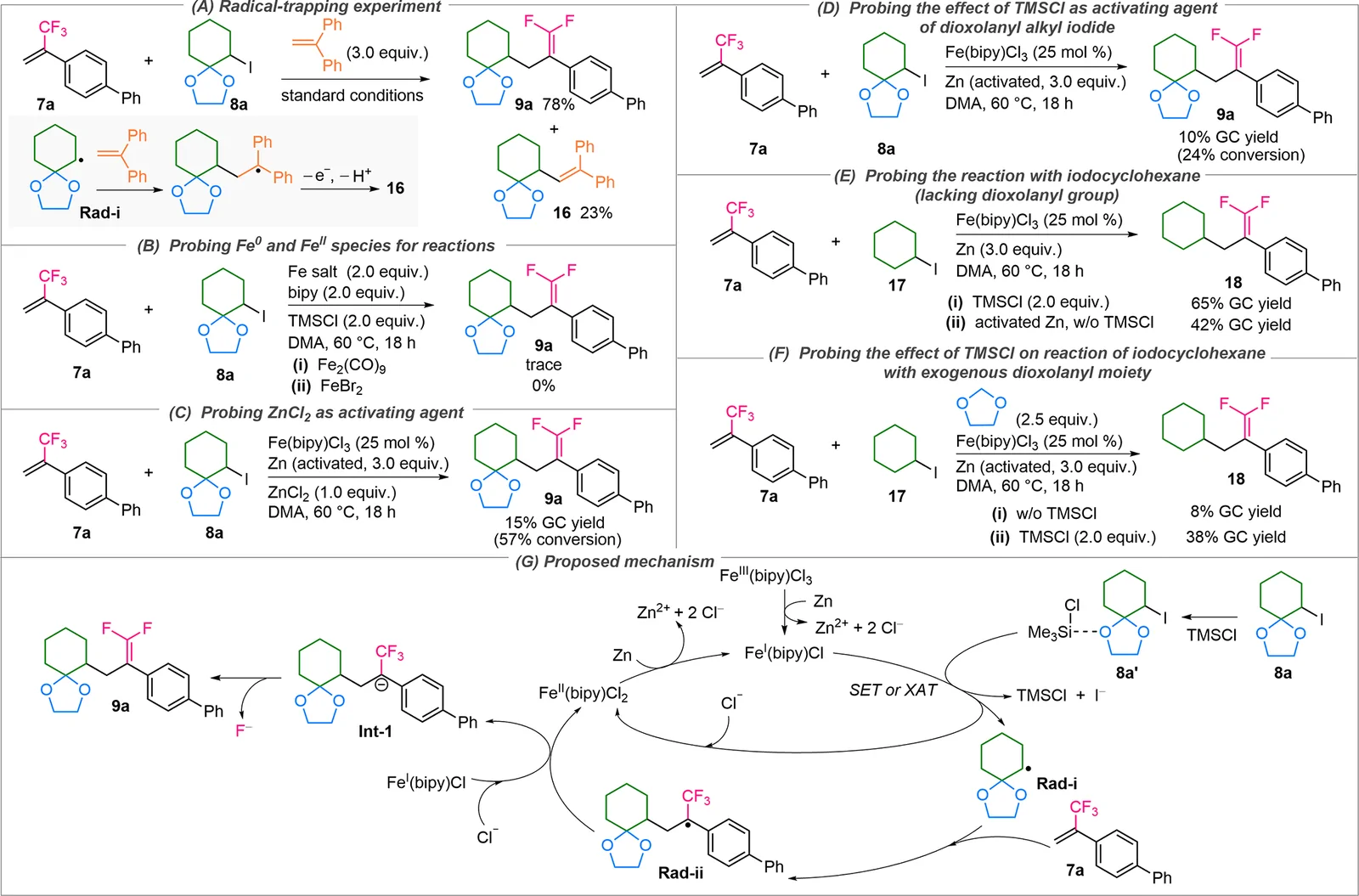
Control Experiments and Proposed Mechanism

Given that Zn ions are generated upon oxidation of Zn and that TMSCl is known to both activate metal reductants^25^ and interact with oxygen-containing functional groups, we hypothesized that either Zn ions or TMSCl could serve as an activating additive to improve the reaction efficiency. To evaluate the role of Zn ions, TMSCl was replaced with ZnCl_2_ in the model reaction mixture (Scheme 3C). The modification proved to be inefficient, affording **9a** in only 15% yield, thus ruling out Zn ions as the key activating species. Furthermore, in the absence of TMSCl, the model reaction also provided **9a** in low yield even when activated Zn was employed (Scheme 3D), suggesting that TMSCl plays a role beyond mere Zn activation. Specifically, it likely functions as an oxophilic additive that facilitates activation of the dioxolanyl alkyl iodide. Consistent with this proposal, iodocyclohexane (**17**), which lacks a dioxolanyl group, afforded *gem*-difluoroalkene **18** in 65% yield under the standard conditions (Scheme 3E, i), and the reaction remained productive using activated Zn without TMSCl (42% (Scheme 3E, ii)). In contrast, addition of excess dioxolane in the absence of TMSCl suppressed the level of formation of **18** to 8% (Scheme 3F, i), whereas inclusion of TMSCl increased the yield of **18** to 38% (Scheme 3F, ii). On the basis of these control experiments, we propose that the dioxolanyl group would interfere with the Fe-promoted alkyl iodide activation through coordination of its oxygen atoms to the Fe catalyst.^26^ TMSCl may attenuate this unproductive interaction by preferentially engaging the dioxolanyl oxygen atoms, thereby restoring productive Fe-mediated C–I activation and radical generation.

Based on these experimental results and previous reports,^12–14^ a plausible mechanism for the defluorinative coupling reaction is proposed (Scheme 3G). Initially, Zn powder, activated by TMSCl through removal of the surface oxide layer,^25^ reduces Fe^III^(bipy)­Cl_3_ (**Fe** ^**III**^) to a low-valent Fe^I^(bipy)Cl species (**Fe** ^**I**^). Meanwhile, TMSCl interacts with an oxygen atom of the dioxolanyl group in alkyl iodide **8a** to form species **8a′**, thereby inhibiting undesirable coordination of the dioxolanyl moiety to the Fe catalyst.^26^ The resulting **Fe** ^**I**^ species then reduces the alkyl iodide through single-electron transfer (SET)^22a–22c^ or halogen-atom transfer (XAT),^22d^ generating the dioxolanyl-cyclohexyl radical (**Rad-i**) together with Fe^II^(bipy)­Cl_2_ (**Fe** ^**II**^). Subsequently, **Rad-i** adds to the terminal carbon of CF_3_-alkene **7a** to form α-CF_3_ alkyl radical **Rad-ii**.^12–14^ Further reduction of **Rad-ii** by **Fe** ^**I**^ gives anionic intermediate **Int-1**, which then undergoes fluoride elimination to furnish dioxolanyl-*gem*-difluoroalkene **9a**. Finally, **Fe** ^**II**^ is reduced by Zn to regenerate the **Fe** ^**I**^ system.

In summary, cyclic acetal–*gem*-difluoroalkenes are indeed highly attractive bioisosteres of carbonyls and valuable pharmaceutical intermediates. Nevertheless, a streamlined, operationally simple, and inexpensive protocol for accessing these structural motifs remains elusive. Here we disclose an Fe-catalyzed reductive defluorinative cross-coupling between cyclic acetal-substituted alkyl iodides and trifluoromethyl alkenes, allowing the efficient modular assembly of these hybrid scaffolds. The inexpensive Fe catalyst system tolerates a range of functional groups, in particular the free amines and halides. This compatibility thus offers excellent opportunities for further structural manipulation via C–N and C–C bond-forming coupling reactions.

## Supplementary Material



## Data Availability

The data underlying this study are available in the published article, in its Supporting Information, and openly available at DOI: 10.5061/dryad.zs7h44jnd.

## Notes

Experimental details, procedures, ^1^H, ^13^C, and ^19^F NMR spectra, and characterization data of the products (PDF)
