# Supplementary material for: Iron-Catalyzed Defluorinative Alkylation for Accessing Cyclic Acetal–gem-Difluoroalkenes
Source: Org Lett. 2026 Sep 22;28(39):12195–201. doi: 10.1021/acs.orglett.6c03239 (PMC13640427; doi:10.1021/acs.orglett.6c03239)
Supplement: Supplementary file 1 [file ol6c03239_si_001.pdf]

Supporting Information

Iron-Catalyzed Defluorinative Alkylation for Accessing Cyclic Acetal-*gem*-Difluoroalkenes

Xiao-Tong Wang, Pui Ying Choy, Chi Wai Cheung* and Fuk Yee Kwong*

State Key Laboratory of Synthetic Chemistry and Department of Chemistry, The Chinese University of Hong Kong, Shatin, New Territories, Hong Kong, China

E-mail: cw.cheung@cuhk.edu.hk; fykwong@cuhk.edu.hk

Table of contents

1. General Information S2
2. Synthesis of Substrates S3
   2.1. General procedures for the synthesis of trifluoromethyl alkenes S3
   2.2. General procedures for the synthesis of cyclic-acetal alkyl halides S4
3. Optimization Experiments S6
4. Mechanistic Studies: Radical-Trapping Experiment S8
   4.1. 1,1-Diphenylethylene Radical-Trapping Experiment S8
   4.2. TEMPO Radical-Trapping Experiment S9
5. General Procedures for Fe-Catalyzed Defluorinative Synthesis of Cyclic Acetal *gem*-Difluoroalkene S11
6. Characterization Data for All Defluorinated Alkylation Products S11
7. General Procedures for Scale-up Synthesis of Product **10c** S21
8. Derivatization of Products S22
   8.1. Synthesis of compound **11** S22
   8.2. Acid-promoted hydrolysis of **10c** S23
   8.3. Acid-promoted hydrolysis of **9a** S24
   8.4. Synthesis of compound **14** S24
   8.5. Synthesis of compound **15** S25
   8.6. Synthesis of compound **19** S26
   8.7. Synthesis of compound **20** S26
   8.8. Synthesis of compound **21** and **21’** S27
   8.9. Synthesis of compound **22** S28
   8.10. Synthesis of compound **23**, **23’** and **24** S29
9. ^{1}H, ^{13}C, and ^{19}F NMR Spectra S31
10. References S103

1. General Information

Unless otherwise noted, all reagents were purchased from commercial suppliers and used without purification. All cascade reactions were performed in a resealable screw-capped Schlenk flask (approx. 20 mL volume) in the presence of Teflon coated magnetic stirrer bar (4 mm × 10 mm). Dimethylacetamide (DMA, 99.8%, Superdry, with molecular sieves) were purchased from J&K Chemical Toluene and tetrahydrofuran (THF) were distilled from sodium under nitrogen.[1] Other solvents were purchased from commercial suppliers. Unless otherwise noted, all reagents were purchased from commercial suppliers and used without purification. Thin-layer chromatography was performed on precoated silica gel 60 F_{254} plates. Silica gel (230-400 mesh) was used for column chromatography. Preparative thin-layer chromatography (PTLC) for purification of compounds was carried out using preparative TLC ($HSGF_{254}$, 0.9-1.0 mm, 20 × 20 cm). ^{1}H NMR spectra were recorded on a 400 or 500 MHz spectrometer. Spectra were referenced internally to the residual proton resonance in $CDCl_3$ (δ = 7.26 ppm) or with tetramethylsilane (TMS, δ = 0.00 ppm) as the internal standard. ^{13}C NMR spectra were recorded on a 100 or 125 MHz spectrometer and the spectra were referenced to $CDCl_3$ (δ 77.16 ppm, the middle peak). ^{19}F NMR spectra (comp. pulse decoupling or no decoupling) were recorded on a 377 or 471 MHz spectrometer and the spectra were referenced to trichlorofluoromethane ($CFCl_3$, 0 ppm). Coupling constants (*J*) were reported in Hertz (Hz). Mass spectra (EI-MS and ES-MS) were recorded on a mass spectrometer. High-resolution mass spectra (HRMS) were obtained by Q Exactive Focus Orbitrap with APCI and ESI source. GC-MS analysis was conducted on a GCD system using a column with 30 m × 0.25 mm. The products described in GC yield were accorded to the authentic samples/tetradecane calibration standard from GC-FID system. Compounds described in the literature were characterized by comparison of their ^{1}H, and/or ^{13}C NMR spectra to the previously reported data. Fe(bipy)Cl_3 was prepared according to the literature procedure.[2]

2. Synthesis of Substrates

2.1. General procedures for the synthesis of trifluoromethyl alkenes

General procedures A for the preparation of aryl-substituted trifluoromethyl alkenes

According to the previous reports, to a Schlenk tube equipped with stir bar, arylboronic acid (1.0 equiv., 3 mmol) and $Pd(PPh_3)_2Cl_2$ (3 mol %, 0.09 mmol, 63.2 mg) were added. The vessel was evacuated and filled with argon (three times), and then aqueous K_2CO_3 (2.0 M, 6 mL) and THF (9 mL) were added. After addition of 2-bromo-3,3,3-trifluoro-1-propene (1.5 equiv., 4.5 mmol, 0.47 mL), the solution was stirred at 60 °C with heating mantle for 12 hours (TLC tracking detection). The solvent was quenched with water, diluted with EtOAc (10 mL) and washed with brine (15 mL) The aqueous layer was extracted with EtOAc (3 x 10 mL). The combined organic layers were dried over Na_2SO_4, filtered and concentrated in vacuum and the residue was purified by column chromatography to afford the corresponding trifluoromethyl alkene **7a-7q**.

General procedures B for the preparation of alkyl-substituted trifluoromethyl alkenes

Step 1: LiCl (252 mg, 6 mmol) was dried with a flame under high vacuum then technical grade Zn powder (390 mg, 6 mmol) was added. The mixture was heated again under high vacuum then allowed to cool under an argon atmosphere. Anhydrous THF (2.0 mL), 1,2-dibromoethane (86 μL, 1.0 mmol) and trimethylsilyl chloride (25 μL, 0.2 mmol) were added via syringe. Then iodine (51 mg, 0.2 mmol) was added. The resulting mixture was stirred until the brown color disappeared. Benzylbromide (4 mmol) in THF (2.0 mL) was then added. After stirring for 3 h at 25 °C, the gray solution was filtered and the filtrate was stored under argon in a Schlenk tube, the solution of the benzylzinc reagent was titrated with I_2 according to Knochel's method.

Step 2: To a 25 mL of Schlenk tube were added $Pd(TFA)_2$ (3 mol %), **SPhos** (3 mol%). The mixture was evacuated and backfilled with argon for three times, then benzylzinc reagent (0.2 mmol, 1.0 equiv), 2-bromo-3,3,3-trifluoropropane 2 (0.4 mmol, 2.0 equiv) and THF (2.0 mL) were added subsequently. The Schlenk tube was screw capped and put into a preheated oil bath (90 °C). After stirring for 8 h, the reaction mixture was cooled to room temperature and fluorobenzene (1.0 equiv) was added. The resulting mixture was filtered and concentrated, and the residue was purified with silica gel chromatography (petroleum) to give product **7t**.

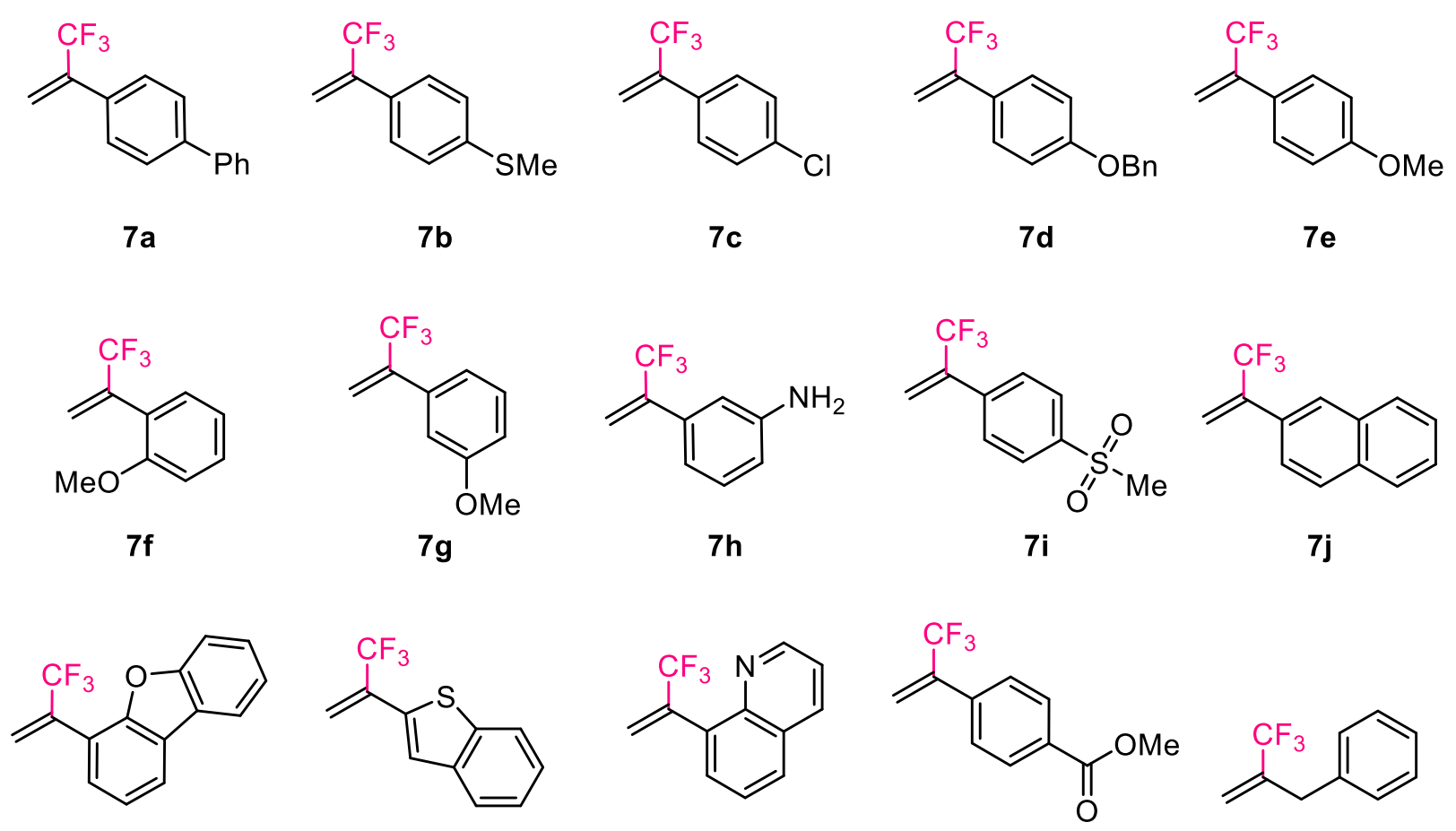

Figure S1. *α*-Trifluoromethyl alkenes **7**

α-Trifluoromethyl alkenes **7a**, **7c**, **7j**, **7l**, **7q** were prepared according to reported literature without modification;[3] **7b**, **7e**, **7f**, **7g**, **7h**, **7i** were prepared according to reported literature without modification;[4] **7d**, **7k** were prepared according to reported literature without modification;[5] **7m** was prepared according to reported literature without modification;[6] **7t** was prepared according to reported literature without modification.[7]

__Note__: **7t** is not applicable substrate for the defluorinative alkylation.

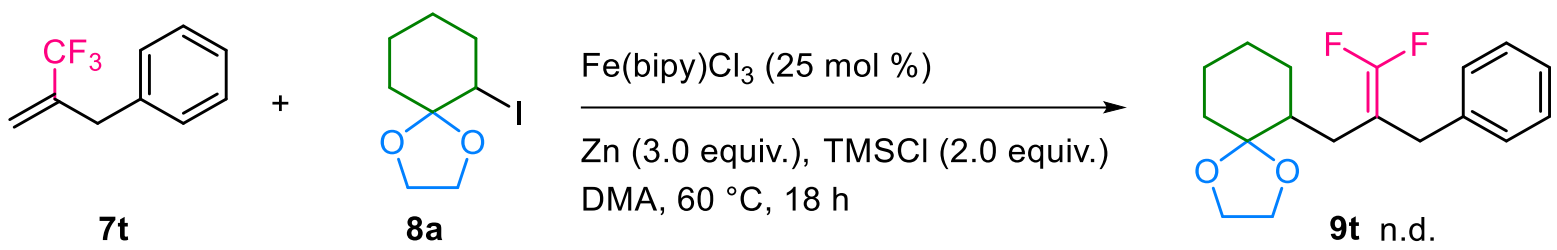

2.2. General procedures for the synthesis of cyclic-acetal alkyl halides[8]

General procedures A for the preparation of α-iodoketals

Step 1: To a solution of the suitable ketone (10 mmol, 1 equiv.) in anhydrous methanol were added iodine (1.0 equiv.) and copper (II) oxide (1 equiv.). The mixture was stirred at room temperature for 5 min and then refluxed until no starting material was detected by TLC. The reaction was cooled, filtered and the solvent was removed. The residue was dissolved in ethyl acetate (10 mL) and washed with a solution of 10% $Na_2S_2O_3$ (20 mL). The aqueous phase was extracted with EtOAc (2 x 20 mL). The combined organic layers were dried over anhydrous Na_2SO_4 and the solvent was evaporated. The α-iodoketones thus obtained were used for the next reaction without further purification.

Step 2: A mixture of iodoketone (5 mmol, 1 equiv.), TMOF (1.2 equiv.) and 30 mol % PTSA in ethylene glycol (10 mL) was stirred at room temperature for 12 h. Then 15 mol % $NaHCO_3$ was added and stirred for a few minutes. After addition of H_2O (20 mL) and extracted with EtOAc (2 x 20 mL), the combined organic layers were washed with brine (20 mL), dried over anhydrous Na_2SO_4, and concentrated under reduced pressure. Column chromatography on silica gel provided the product **8a-8r**.

General procedures B for the preparation of α-iodoacetals

Step 1: A flask charged with aldehyde (10 mmol) in CH_2Cl_2 (40 mL) was cooled to 0 °C. N-iodosuccinimide (4.0 g, 18 mmol) and proline (0.32 g, 2.8 mmol) were added, and the reaction mixture was warmed to room temperature. It was allowed to stir for 8 hours until the starting aldehyde was consumed. Upon completion, the reaction was diluted with pentanes and filtered. The crude filtrate was concentrated carefully and carried forward without further purification.

Step 2: A mixture of iodo aldehyde (5 mmol, 1.0 eq), 30 mol % PTSA and diols (5.0 eq) in CH_2Cl_2 (10 mL) was stirred at room temperature for 12 h. Then 15 mol % $NaHCO_3$ was added and stirred for a few minutes. After addition of H_2O (30 mL), and extracted with CH_2Cl_2 (2 x 20 mL), the combined organic layers were washed with brine (20 mL), dried over anhydrous Na_2SO_4, and concentrated under reduced pressure. Column chromatography on silica gel provided the product **8t-8x**.

General procedures C for the preparation of α-bromoacetals

To a stirred solution of the ketone (1.12g, 10 mmol) in THF (12 mL) and the ethylene glycol (12 mL) is added phenyl trimethylammonium tribromide (5.64 g, 15 mmol). The homogeneous reaction mixture is stirred at 28 °C for 24 h and then poured into a cold solution of 10% sodium hydrogen carbonate (250 ml) and 5% sodium thiosulphate (25 ml) and extracted with ether (2×100 ml). The combined organic layer is washed with water and brine and dried with anhydrous sodium sulfate. The solvent is removed under reduced pressure to yield the product which is further purified by chromatography, provided the compound **8s**.

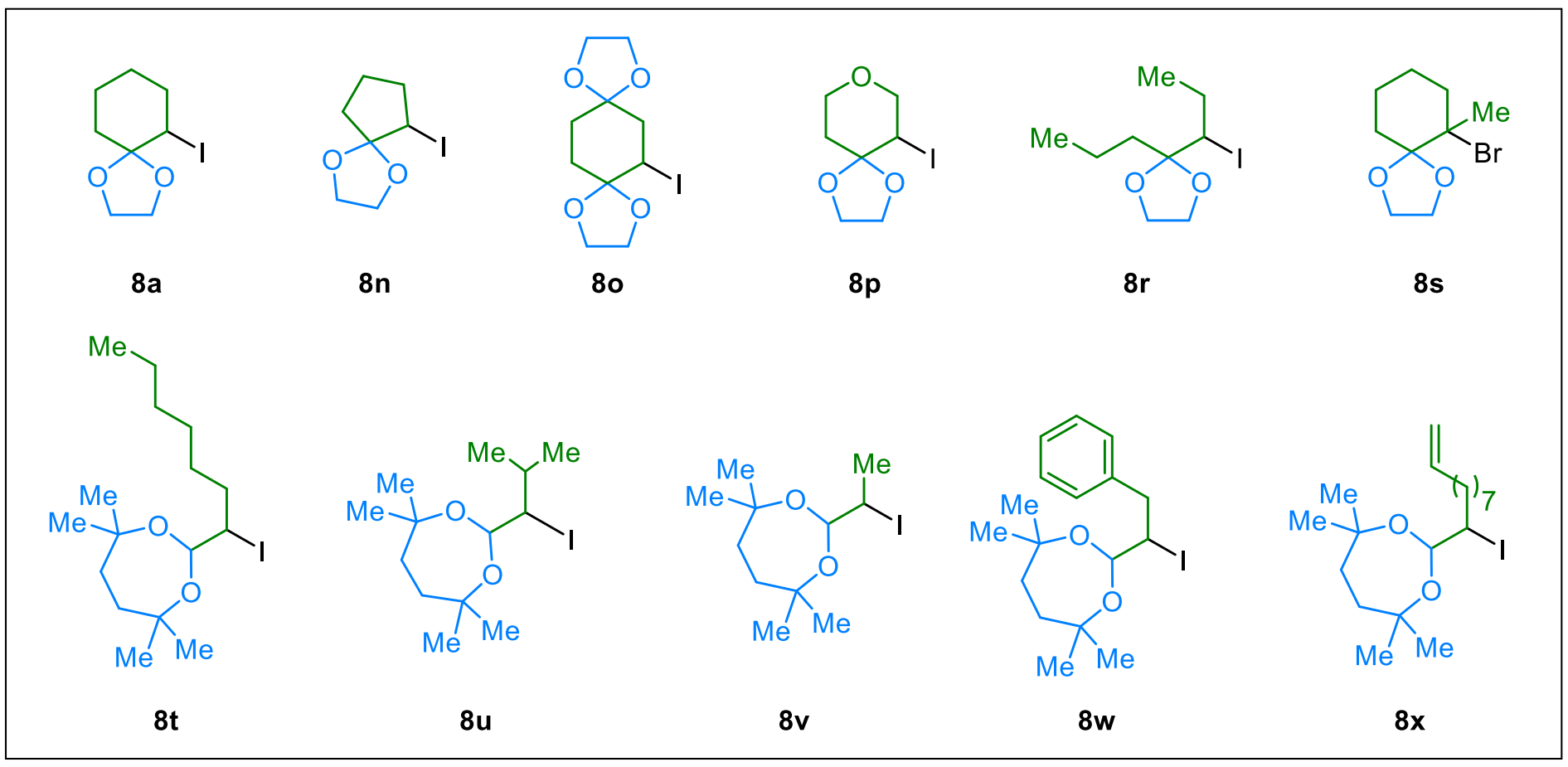

Figure S2. Cyclic acetal-alkyl halides **8**

Cyclic acetal-alkyl iodides **8a-8r, 8t-8x** were prepared according to reported literature without modification;[8] Cyclic acetal-alkyl bromide **8s** was prepared according to reported literature without modification.[9]

3. Optimization Experiments

General procedures of optimization experiments: To a flame-dried tube equipped with a Teflon-coated magnetic stir bar were added the appropriate Fe salt, reductant, 4-(3,3,3-trifluoroprop-1-en-2-yl)-1,1'-biphenyl (24.8 mg, 0.1 mmol, 1.0 equiv.) and 6-iodo-1,4-dioxaspiro[4.5]decane. The system was further evacuated and flushed with nitrogen for three cycles. Solvent and TMSCl were added with stirring at room temperature for several minutes. The tube was then placed into a pre-heated oil bath and stirred for the duration as indicated. The reaction tube was allowed to cool to room temperature. Ethyl acetate (2 mL), tetradecane (26 μL, internal standard), and water (2 mL) were added. The organic layer was subjected to GC analysis. The GC yield was previously calibrated by authentic sample/*n*-tetradecane calibration curve.

Table S1. Optimization of the reaction conditions of CF_3-substituted vinylbiphenyl **7a** with α-iodoketal **8a**[a]

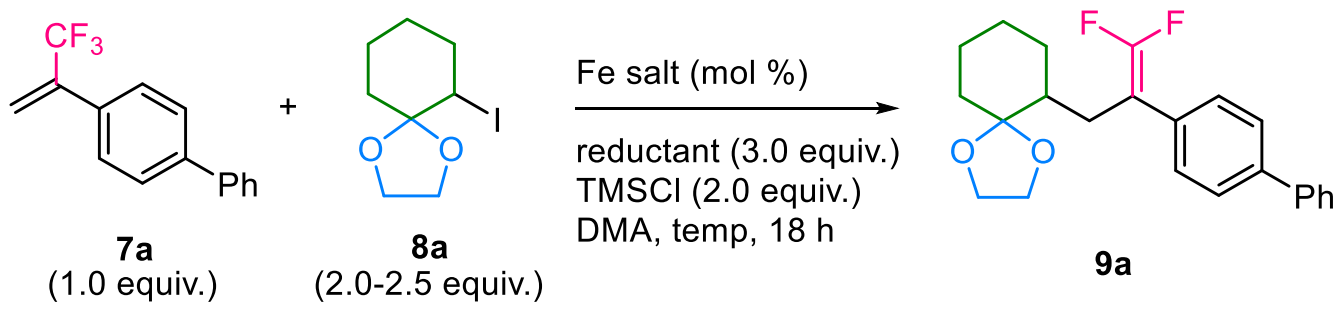

entry	**8a** (equiv.)	Fe catalyst (mol %)	reductant	solvent	yield (%)[b]
1	2	$FeCl_3•6H_2O$ (30)	Zn	DMF	53
2	2	$FeCl_3•6H_2O$ (30)	Zn	NMP	54
3	2	$FeCl_3•6H_2O$ (30)	Zn	DMSO	29
4	2	$FeCl_3•6H_2O$ (30)	Zn	MeCN	35
5	2	$FeCl_3•6H_2O$ (30)	Zn	CH_2Cl_2	n.r.
6	2	$FeCl_3•6H_2O$ (30)	Zn	1,4-Dioxane	<5
7	2	$FeCl_3•6H_2O$ (30)	Zn	DMA	58
8	2	$FeBr_2$ (30)	Zn	DMA	51
9	2	$Fe(OTf)_3$ (30)	Zn	DMA	52
10	2	$FeCl_3•6H_2O$ (0)	Zn	DMA	<5
11[c]	2	$FeCl_3•6H_2O$ (30)	Zn	DMA	18
12	2.5	$FeCl_3•6H_2O$ (30)	Zn	DMA	64
13[d]	2.5	$FeCl_3•6H_2O$ (30)	Zn	DMA	70
14[d]	2.5	$FeCl_3•6H_2O$ (30)	Mn	DMA	56
15[d]	2.5	$FeCl_3•6H_2O$ (30)	Fe	DMA	<5
16[d,e]	2.5	$FeCl_3•6H_2O$ (30)	Zn	DMA	76
17[d,e]	2.5	$FeCl_3•6H_2O$ (25)	Zn	DMA	74
18[d,e]	2.5	$Fe(phen)Cl_3$ (25)	Zn	DMA	52
19[d,e]	2.5	$Fe(bipy)Cl_3$ (25)	Zn	DMA	89
20[d,e]	2.5	$Fe(bipy)Cl_3$ (20)	Zn	DMA	52

[a]Reaction conditions: **7a** (1.0 equiv., 0.1 mmol), **8a** (2.0–2.5 equiv.), Fe salt (0–30 mol %), metal reductant (3.0 equiv., 0.3 mmol), TMSCl (2.0 equiv., 0.2 mmol), DMA (2 mL), 80 °C, N_2 atmosphere, 18 h. [b]GC yield using tetradecane as the internal standard. [c]1.0 equiv. of TMSCl was used. [d]1.5 mL of DMA was used. [e]Reaction was performed at 60 °C.

4. Mechanistic Studies: Radical-Trapping Experiment

4.1. 1,1-Diphenylethylene Radical-Trapping Experiment

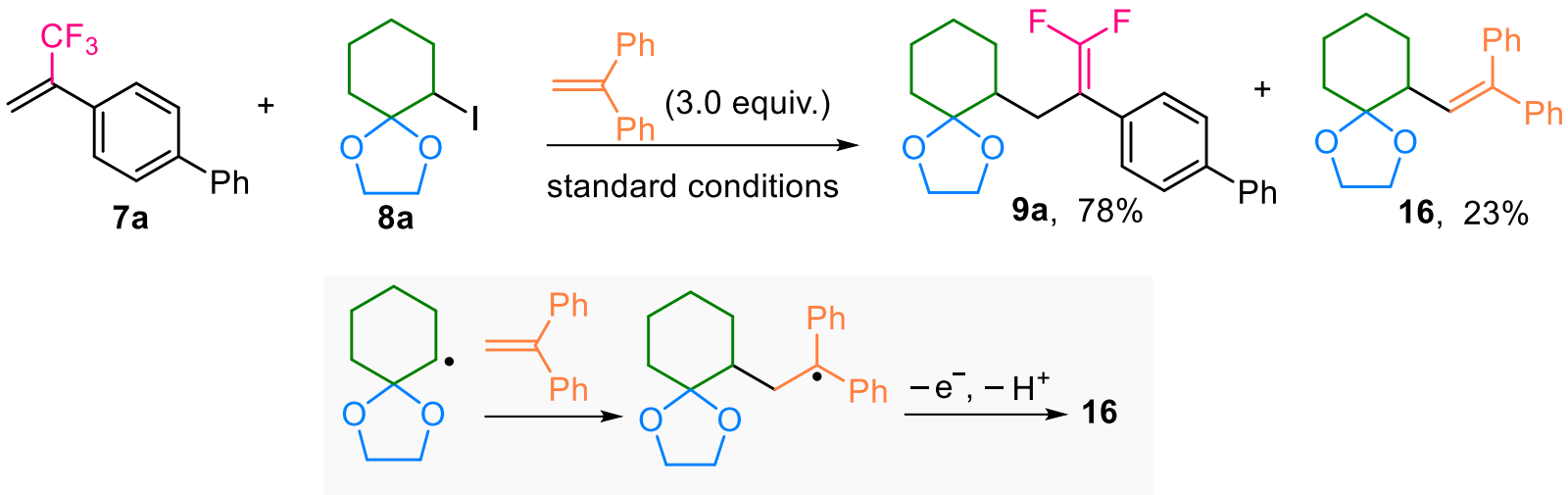

The involvement of radical intermediates was probed by adding the radical scavenger 1,1-diphenylethylene (DPE) to the model reaction (Table S1, entry 19). Under these conditions, the desired product **9a** was obtained in a slightly diminished yield, together with trapping products **16**, which arise from the addition of the dioxolanyl–cyclohexyl radical **Rad-i** to DPE, followed by oxidation. These results support single-electron reduction of the alkyl iodide to generate Rad-i, which subsequently partic-ipates in the defluorinative alkylation.

General procedures for DPE radical-trapping experiment:

To a flame-dried tube equipped with a Teflon-coated magnetic stir bar were added the Fe(bipy)Cl_3 (8.0 mg, 25 mol %), Zn (19.5 mg, 0.30 mmol), DPE (53.0 µL, 0.30 mmol), CF_3-alkene **7a** (0.1 mmol, 1.0 equiv.) and acetal-alkyl iodide **8a** (0.25 mmol). The system was further evacuated and flushed with nitrogen for three cycles. DMA (1.5 mL) and TMSCl (25.4 µL) were added with stirring at room temperature for several minutes. The tube was then placed into a pre-heated oil bath (60 °C) and stirred for 18 h. After completion of reaction, the reaction tube was allowed to cool to room temperature. Ethyl acetate (~3 mL) and water (~3 mL) were added. The desired product **9a** was obtained in a slightly diminished yield calculated by GC-FID analysis using *n*-dodecane as the internal standard. Concurrently, the radical trapping adducts **16** were confirmed by HRMS analysis and were quantified in 23% GC yields.

HRMS result:

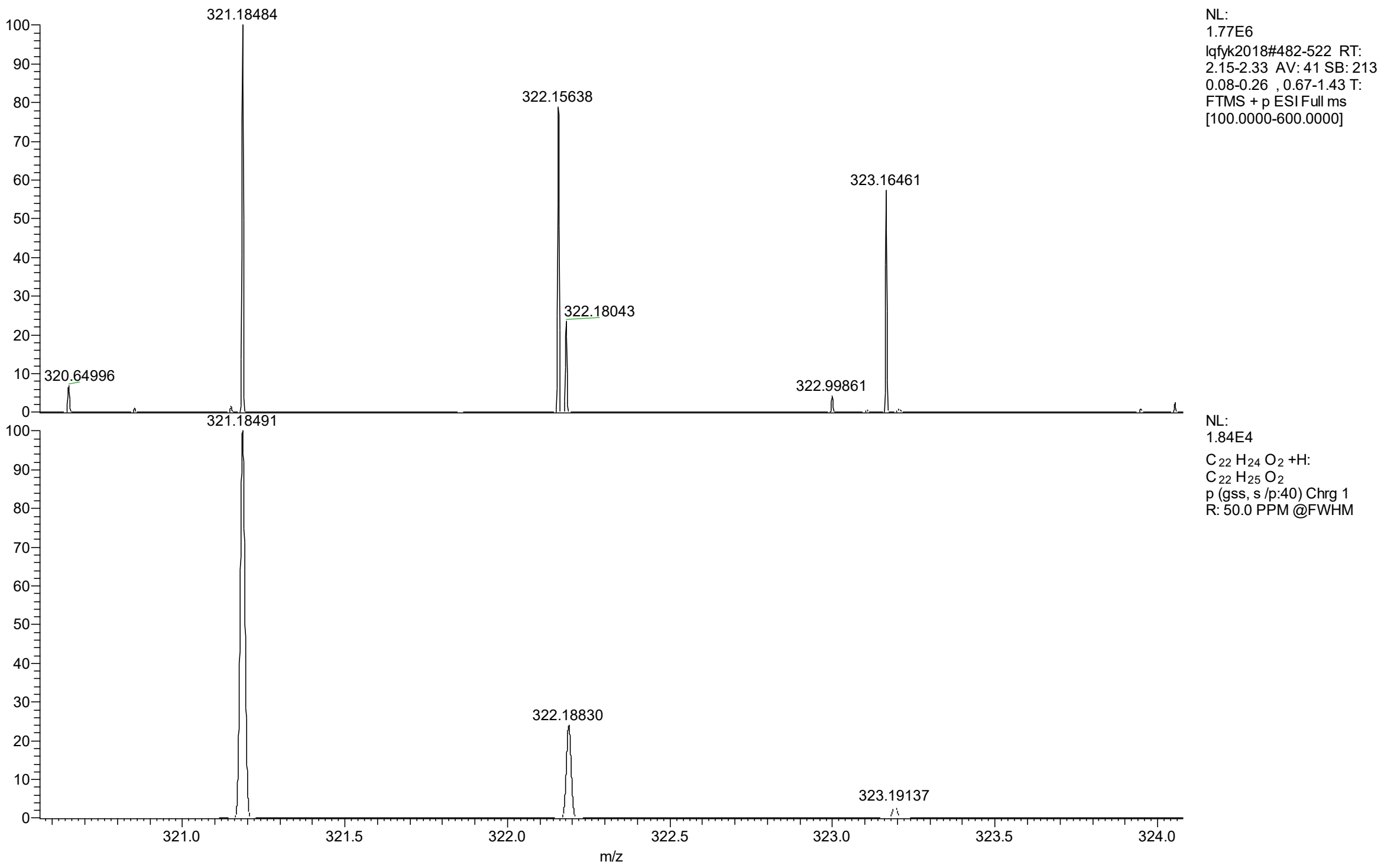

HRMS (ESI) *m/z*: $[M+H]^+$ Calcd for $C_{22}H_{25}O_2$ **(16)** 321.1849; Found 321.1848.

4.2. TEMPO Radical-Trapping Experiment

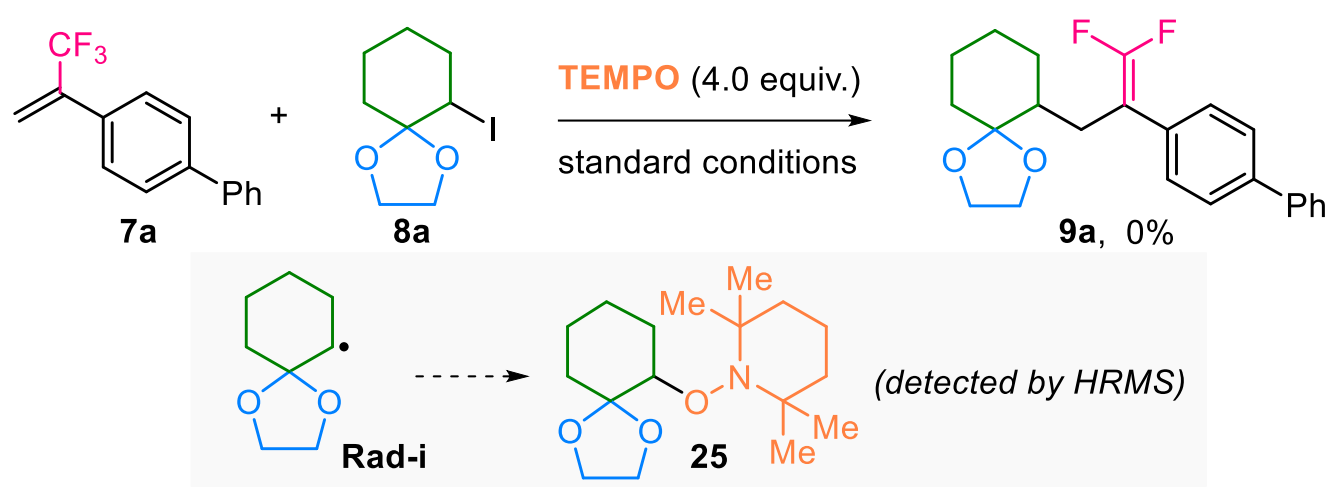

To probe the radical nature of the reaction, 2,2,6,6-tetramethylpiperidin-1-oxyl (TEMPO), an efficient radical trap, was added to the model reaction (Table S1, entry 19). Under these conditions, the desired coupling product **9a** was not formed, whereas adduct **25**, derived from TEMPO and the dioxolanyl-cyclohexyl radical **Rad-i**, was detected by high-resolution mass spectrometry (HRMS). This result suggests that the alkyl iodide is reduced to **Rad-i**, which subsequently participates in the defluorinative alkylation.

General procedures for TEMPO radical-trapping experiment:

To a flame-dried tube equipped with a Teflon-coated magnetic stir bar were added the Fe(bipy)Cl_3 (8.0 mg, 25 mol %), Zn (19.5 mg, 0.30 mmol), TEMPO (61.6 mg, 0.40 mmol), CF_3-alkene **7a** (0.1 mmol, 1.0 equiv.) and acetal-alkyl iodide **8a** (0.25 mmol). The system was further evacuated and

flushed with nitrogen for three cycles. DMA (1.5 mL) and TMSCl (25.4 μL) were added with stirring at room temperature for several minutes. The tube was then placed into a pre-heated oil bath (60 °C) and stirred for 18 h. After completion of reaction, the reaction tube was allowed to cool to room temperature. Ethyl acetate (~3 mL) and water (~3 mL) were added. No product **9a** was detected by TLC and GC-MS analysis, while the TEMPO-trapped adducts **25** were identified by HRMS analysis.

HRMS result:

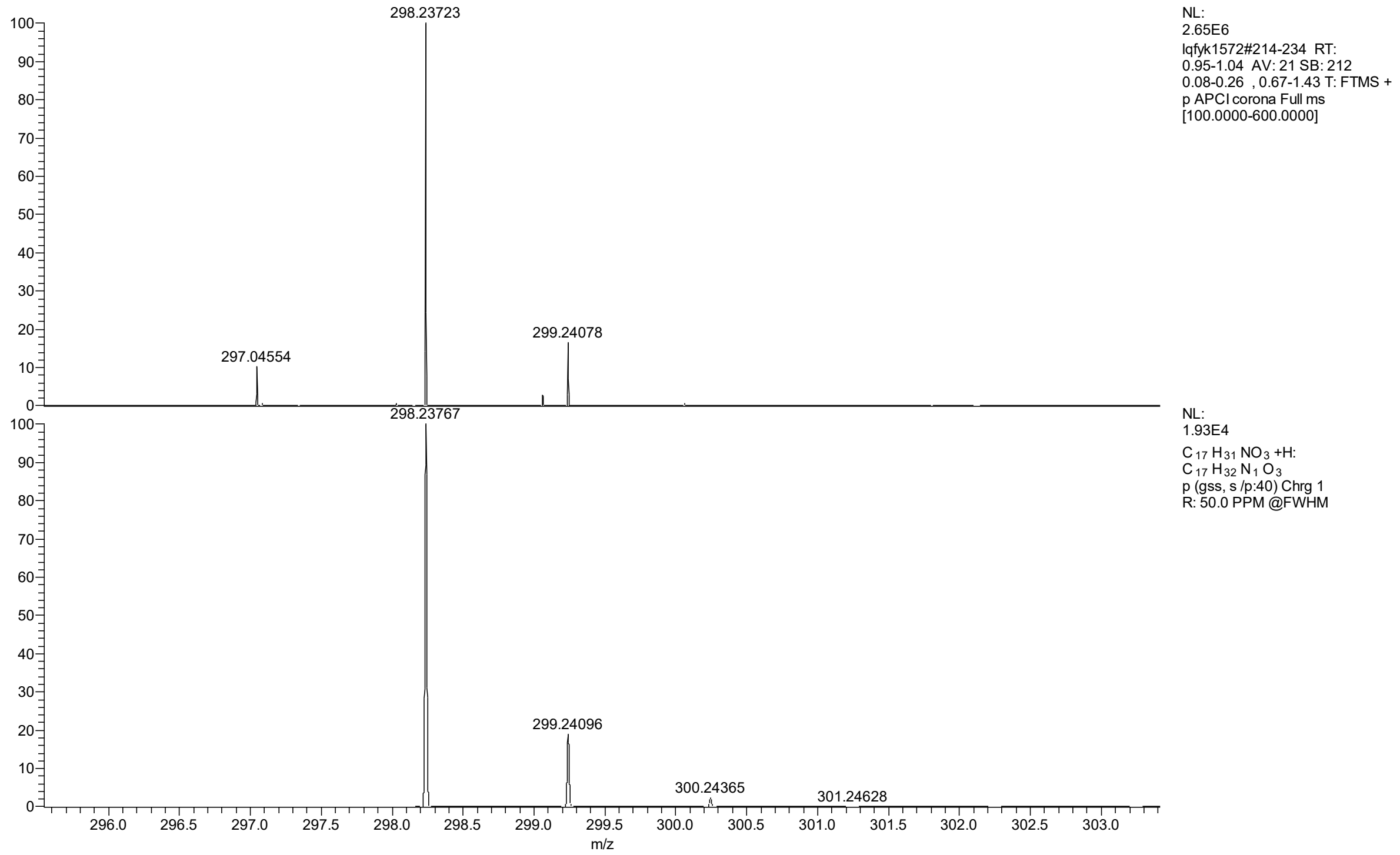

HRMS (ESI) *m/z*: $[M+H]^+$ Calcd for $C_{17}H_{32}NO_3$ **(25)** 298.2377; Found 298.2372.

5. General Procedures for Fe-Catalyzed Defluorinative Synthesis of Cyclic Acetal *gem*-Difluoroalkene

To a flame-dried tube equipped with a stir bar were added the Fe(bipy)Cl_3 (8.0 mg, 25 mol %), Zn (19.5 mg, 0.30 mmol), CF_3-alkene **7** (0.1 mmol, 1.0 equiv.) and acetal-alkyl iodide **8** (0.25 mmol). The system was further evacuated and flushed with nitrogen for three cycles. DMA (1.5 mL) and TMSCl (25.4 µL) were added with stirring at room temperature for several minutes. The tube was then placed into a pre-heated oil bath (60 °C) and stirred for 18 h. After completion of reaction, the reaction tube was allowed to cool to room temperature. Ethyl acetate (~3 mL) and water (~3 mL) were added and the organic layer was subjected to GC analysis. The aqueous layer was further extracted by ethyl acetate (~3 mL x 3 times). The combined organic layer was concentrated in *vacuo*. The crude product was purified by preparative thin-layer chromatography on silica gel.

6. Characterization Data for All Defluorinated Alkylation Products

6-(2-([1,1'-Biphenyl]-4-yl)-3,3-difluoroallyl)-1,4-dioxaspiro[4.5]decane (Scheme 2, product 9a)

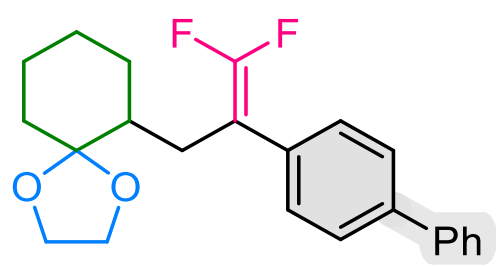

Yellow oily liquid (84%, 31.1 mg) was obtained. EtOAc: Hexane = 1:15, R_f = 0.30. **^{1}H NMR (500 MHz, Chloroform-*d*)** δ 7.63 – 7.57 (m, 4H), 7.48 – 7.41 (m, 4H), 7.38 – 7.33 (m, 1H), 4.03 – 3.92 (m, 4H), 2.80 – 2.71 (m, 1H), 2.38 – 2.28 (m, 1H), 1.80 – 1.71 (m, 2H), 1.68 – 1.57 (m, 3H), 1.52 – 1.43 (m, 1H), 1.34 – 1.25 (m, 2H), 1.17 – 1.09 (m, 1H). **^{13}C{^{1}H} NMR (125 MHz, Chloroform-*d*)** δ 154.2 (dd, J_{CF} = 291.0, 286.5 Hz), 140.6, 139.8, 132.5 (t, J_{CF} = 3.9 Hz), 128.8, 128.6 (t, J_{CF} = 3.6 Hz), 127.4, 127.1, 127.0, 110.5, 90.6 (dd, J_{CF} = 21.7, 12.6 Hz), 64.8, 64.7, 42.2, 34.5, 28.6, 25.9, 24.4, 23.8. **^{19}F NMR (471 MHz, Chloroform-*d*)** δ –89.98 (d, J_{FF} = 43.1 Hz), –90.28 (d, J_{FF} = 42.4 Hz). **HRMS (APCI)** *m/z*: [M+H]$^+$ Calcd for $C_{23}H_{25}F_2O_2$ 371.1817; Found 371.1809.

6-(3,3-Difluoro-2-(4-(methylthio)phenyl)allyl)-1,4-dioxaspiro[4.5]decane (Scheme 2, product 9b)

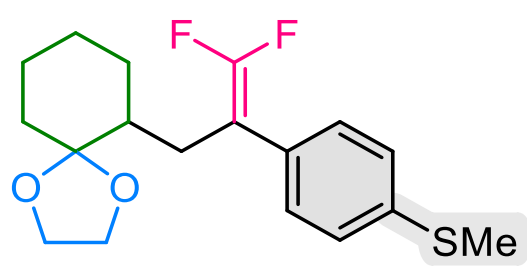

Light yellow oily liquid (81%, 27.5 mg) was obtained. EtOAc: Hexane = 1:10, R_f = 0.25. **^{1}H NMR (400 MHz, Chloroform-*d*)** δ 7.29 – 7.20 (m, 4H), 4.01 – 3.89 (m, 4H), 2.71 – 2.63 (m, 1H), 2.48 (s,

3H), 2.31 – 2.23 (m, 1H), 1.78 – 1.72 (m, 1H), 1.70 – 1.64 (m, 1H), 1.62 – 1.58 (m, 1H), 1.57 – 1.55 (m, 1H), 1.49 – 1.40 (m, 1H), 1.32 – 1.18 (m, 3H), 1.15 – 1.03 (m, 1H). **^{13}C{^{1}H} NMR (100 MHz, Chloroform-*d*)** δ 154.2 (dd, J_{CF} = 290.5, 286.2 Hz), 137.4, 130.3 (t, J_{CF} = 3.9 Hz), 128.7 (t, J_{CF} = 3.6 Hz), 126.5, 110.5, 90.5 (dd, J_{CF} = 21.9, 12.5 Hz), 64.9, 64.8, 42.2, 34.6, 28.6, 26.0, 24.5, 23.9, 15.8. **^{19}F NMR (377 MHz, Chloroform-*d*)** δ –90.33 (d, J_{FF} = 43.5 Hz), –90.75 (d, J_{FF} = 43.6 Hz). **HRMS (APCI)** *m/z*: [M+H]$^+$ Calcd for $C_{18}H_{23}F_2O_2S$ 341.1381; Found 341.1378.

6-(2-(4-Chlorophenyl)-3,3-difluoroallyl)-1,4-dioxaspiro[4.5]decane (Scheme 2, product 9c)

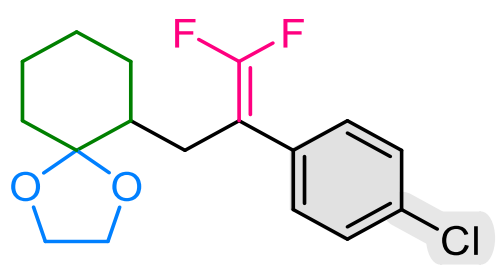

Colorless oily liquid (84%, 27.6 mg) was obtained. EtOAc: Hexane = 1:20, R$_f$ = 0.30. **^{1}H NMR (400 MHz, Chloroform-*d*)** δ 7.32 (d, *J* = 8.7 Hz, 2H), 7.28 (d, *J* = 8.7 Hz, 2H), 4.02 – 3.89 (m, 4H), 2.72 – 2.62 (m, 1H), 2.32 – 2.23 (m, 1H), 1.81 – 1.71 (m, 1H), 1.67 – 1.52 (m, 4H), 1.50 – 1.38 (m, 1H), 1.31 – 1.21 (m, 2H), 1.15 – 1.04 (m, 1H). **^{3}C{^{1}H} NMR (100 MHz, Chloroform-*d*)** δ 154.2 (dd, J_{CF} = 290.9, 286.9 Hz), 133.0, 132.1 (t, J_{CF} = 3.8 Hz), 129.7 (t, J_{CF} = 3.6 Hz), 128.8, 110.4, 90.3 (dd, J_{CF} = 22.2, 12.7 Hz), 64.9, 64.8, 42.3, 34.5, 28.7, 26.1, 24.5, 23.9. **^{19}F NMR (377 MHz, Chloroform-*d*)** δ –89.89 (d, J_{FF} = 42.2 Hz), –90.19 (d, J_{FF} = 41.9 Hz). **HRMS (APCI)** *m/z*: [M+H]$^+$ Calcd for $C_{17}H_{20}ClF_2O_2$ 329.1114; Found 329.1111.

6-(2-(4-(Benzyloxy)phenyl)-3,3-difluoroallyl)-1,4-dioxaspiro[4.5]decane (Scheme 2, product 9d)

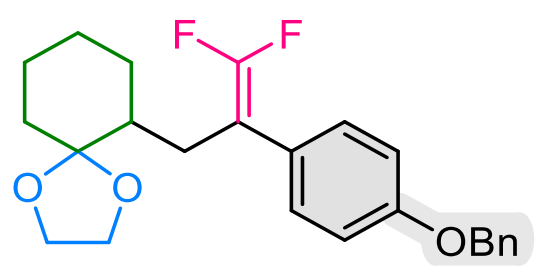

Colorless oily liquid (84%, 33.6 mg) was obtained. EtOAc: Hexane = 1:20, R$_f$ = 0.20. **^{1}H NMR (400 MHz, Chloroform-*d*)** δ 7.50 – 7.31 (m, 5H), 7.29 (d, *J* = 8.4 Hz, 2H), 6.97 (d, *J* = 8.7 Hz, 2H), 5.07 (s, 2H), 4.16 – 3.72 (m, 4H), 2.78 – 2.57 (m, 1H), 2.37 – 2.21 (m, 1H), 1.81 – 1.66 (m, 2H), 1.67 – 1.52 (m, 3H), 1.55 – 1.40 (m, 1H), 1.37 – 1.21 (m, 2H), 1.19 – 1.01 (m, 1H). **^{13}C{^{1}H} NMR (100 MHz, Chloroform-*d*)** δ 157.9, 154.1 (dd, J_{CF} = 289.4, 285.3 Hz), 137.0, 129.5 (t, J_{CF} = 3.6 Hz), 128.7, 128.1, 127.6, 126.0 (t, J_{CF} = 3.8 Hz), 114.9, 110.6, 90.4 (dd, J_{CF} = 21.7, 12.8 Hz), 70.1, 64.9, 64.8, 42.2, 34.6, 28.6, 26.1, 24.5, 23.9. **^{19}F NMR (377 MHz, Chloroform-*d*)** δ –91.48 (d, J_{FF} = 46.1 Hz), –91.93 (d, J_{FF} = 46.2 Hz). **HRMS (ESI)** *m/z*: [M+Na]$^+$ Calcd for $C_{24}H_{26}F_2O_3Na$ 423.1742; Found 423.1739.

6-(3,3-Difluoro-2-(4-methoxyphenyl)allyl)-1,4-dioxaspiro[4.5]decane (Scheme 2, product 9e)

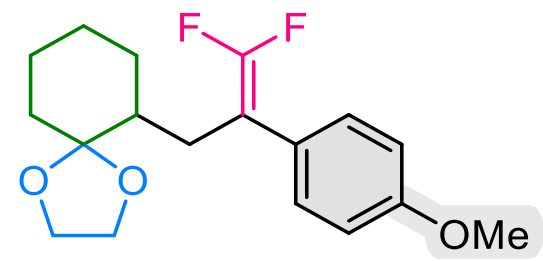

Light yellow oily liquid (82%, 26.6 mg) was obtained. EtOAc: Hexane = 1:5, R_f = 0.25. **^{1}H NMR (400 MHz, Chloroform-*d*)** δ 7.27 (d, *J* = 8.7 Hz, 2H), 6.89 (d, *J* = 8.6 Hz, 2H), 4.05 – 3.87 (m, 4H), 3.81 (s, 3H), 2.76 – 2.58 (m, 1H), 2.35 – 2.20 (m, 1H), 1.80 – 1.72 (m, 1H), 1.72 – 1.65 (m, 1H), 1.64 – 1.51 (m, 3H), 1.53 – 1.36 (m, 1H), 1.32 – 1.19 (m, 2H), 1.18 – 1.03 (m, 1H). **^{13}C{^{1}H} NMR (125 MHz, Chloroform-*d*)** δ 158.6, 154.1 (dd, J_{CF} = 289.3, 285.3 Hz), 129.4 (t, J_{CF} = 3.6 Hz), 125.8 (t, J_{CF} = 3.9 Hz), 114.0, 110.6, 90.4 (dd, J_{CF} = 21.7, 13.0 Hz), 64.9, 64.8, 55.3, 42.2, 34.6, 28.6, 26.2, 24.5, 23.9. **^{19}F NMR (471 MHz, Chloroform-*d*)** δ –91.65 (d, J_{FF} = 46.1 Hz), –92.10 (d, J_{FF} = 46.2 Hz). **HRMS (APCI)** *m/z*: [M+H]$^+$ Calcd for $C_{18}H_{23}F_2O_3$ 325.1610; Found 325.1601.

6-(3,3-Difluoro-2-(2-methoxyphenyl)allyl)-1,4-dioxaspiro[4.5]decane (Scheme 2, product 9f)

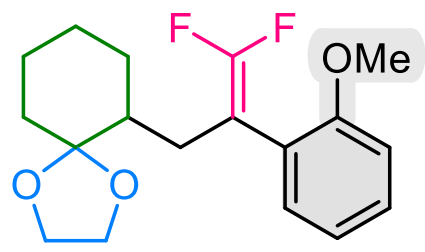

Colorless oily liquid (59%, 19.1 mg) was obtained. EtOAc: Hexane = 1:5, R_f = 0.25. **^{1}H NMR (400 MHz, Chloroform-*d*)** δ 7.31 – 7.21 (m, 1H), 7.16 – 7.09 (m, 1H), 6.98 – 6.84 (m, 2H), 4.04 – 3.85 (m, 4H), 3.81 (s, 3H), 2.73 – 2.52 (m, 1H), 2.35 – 2.16 (m, 1H), 1.88 – 1.77 (m, 1H), 1.76 – 1.67 (m, 1H), 1.66 – 1.54 (m, 2H), 1.53 – 1.39 (m, 2H), 1.31 – 1.20 (m, 2H), 1.19 – 1.03 (m, 1H). **^{13}C{^{1}H} NMR (100 MHz, Chloroform-*d*)** δ 157.5, 153.6 (dd, J_{CF} = 286.0, 285.5 Hz), 131.1 (t, J_{CF} = 2.3 Hz), 129.1, 122.8 (dd, J_{CF} = 5.0, 5.0 Hz), 120.6, 111.2, 110.6, 87.8 (dd, J_{CF} = 23.6, 16.6 Hz), 64.8(4), 64.8(0), 55.6, 42.4, 34.7, 28.7, 26.8, 24.5, 24.0. **^{19}F NMR (377 MHz, Chloroform-*d*)** δ –89.36 (d, J_{FF} = 44.0 Hz), –94.09 (d, J_{FF} = 44.2 Hz). **HRMS (ESI)** *m/z*: [M+Na]$^+$ Calcd for $C_{18}H_{22}F_2O_3Na$ 347.1429; Found 347.1426.

6-(3,3-Difluoro-2-(3-methoxyphenyl)allyl)-1,4-dioxaspiro[4.5]decane (Scheme 2, product 9g)

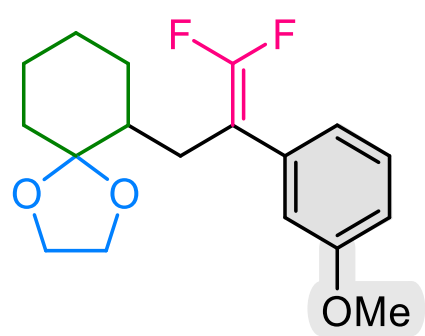

Colorless oily liquid (71%, 23.0 mg) was obtained. EtOAc: Hexane = 1:5, R_f = 0.25. **^{1}H NMR (500 MHz, Chloroform-*d*)** δ 7.32 – 7.25 (m, 1H), 6.98 – 6.93 (m, 1H), 6.91 (s, 1H), 6.85 – 6.79 (m, 1H), 4.10 – 3.88 (m, 4H), 3.82 (s, 3H), 2.81 – 2.60 (m, 1H), 2.40 – 2.20 (m, 1H), 1.82 – 1.74 (m, 1H), 1.73

– 1.67 (m, 1H), 1.65 – 1.56 (m, 3H), 1.54 – 1.42 (m, 1H), 1.35 – 1.22 (m, 2H), 1.18 – 1.06 (m, 1H). **^{13}C{^{1}H} NMR (125 MHz, Chloroform-*d*)** δ 159.6, 154.3 (dd, J_{CF} = 290.8, 286.1 Hz), 135.1 (t, J_{CF} = 4.0 Hz), 129.5, 120.8 (t, J_{CF} = 3.4 Hz), 114.3 (t, J_{CF} = 3.6 Hz), 112.6, 110.6, 91.0 (dd, J_{CF} = 22.0, 12.4 Hz), 64.9, 64.8, 55.3, 42.3, 34.6, 28.7, 26.2, 24.5, 23.9. **^{19}F NMR (471 MHz, Chloroform-*d*)** δ –89.86 (dd, J_{FF} = 42.6, 5.1 Hz), –90.66 (d, J_{FF} = 42.6 Hz). **HRMS (ESI)** *m/z*: [M+Na]$^+$ Calcd for $C_{18}H_{22}F_2O_3Na$ 347.1429; Found 347.1426.

3-(1,1-Difluoro-3-(1,4-dioxaspiro[4.5]decan-6-yl)prop-1-en-2-yl)aniline (Scheme 2, product 9h)

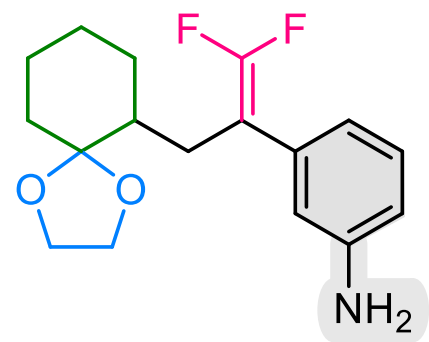

Yellow oily liquid (53%, 16.4 mg) was obtained. EtOAc: Hexane = 1:4, R$_f$ = 0.20. **^{1}H NMR (400 MHz, Chloroform-*d*)** δ 7.17 – 7.09 (m, 1H), 6.74 (d, *J* = 7.7 Hz, 1H), 6.67 (s, 1H), 6.59 (d, *J* = 7.9 Hz, 1H), 4.16 – 3.85 (m, 4H), 3.66 (s, 2H), 2.79 – 2.53 (m, 1H), 2.36 – 2.18 (m, 1H), 1.77 – 1.66 (m, 2H), 1.62 – 1.54 (m, 2H), 1.53 – 1.42 (m, 1H), 1.30 – 1.21 (m, 3H), 1.18 – 1.05 (m, 1H). **^{13}C{^{1}H} NMR (100 MHz, Chloroform-*d*)** δ 154.1 (dd, J_{CF} = 290.5, 285.6 Hz), 146.4, 134.7 (t, J_{CF} = 3.9 Hz), 129.4, 118.7 (t, J_{CF} = 3.4 Hz), 115.1 (t, J_{CF} = 3.5 Hz), 114.2, 110.6, 91.0 (dd, J_{CF} = 21.5, 12.6 Hz), 64.9, 64.8, 42.2, 34.6, 28.6, 26.1, 24.5, 23.9. **^{19}F NMR (377 MHz, Chloroform-*d*)** δ –90.18 (d, J_{FF} = 43.6 Hz), –91.11 (d, J_{FF} = 43.7 Hz). **HRMS (ESI)** *m/z*: [M+Na]$^+$ Calcd for $C_{17}H_{21}F_2NO_2Na$ 332.1433; Found 332.1430.

6-(3,3-Difluoro-2-(4-(methylsulfonyl)phenyl)allyl)-1,4-dioxaspiro[4.5]decane (Scheme 2, product 9i)

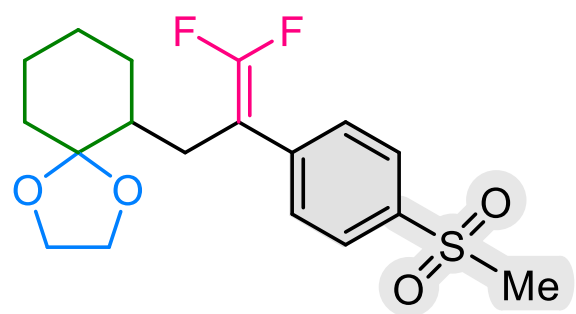

Colorless oily liquid (72%, 26.8 mg) was obtained. EtOAc: Hexane = 1:4, R$_f$ = 0.20. **1H NMR (400 MHz, Chloroform-*d*)** δ 7.91 (d, *J* = 8.2 Hz, 2H), 7.55 (d, *J* = 8.1 Hz, 2H), 4.01 – 3.88 (m, 4H), 3.07 (s, 3H), 2.81 – 2.66 (m, 1H), 2.37 – 2.27 (m, 1H), 1.79 – 1.72 (m, 1H), 1.68 – 1.56 (m, 3H), 1.53 – 1.41 (m, 2H), 1.33 – 1.17 (m, 2H), 1.15 – 1.04 (m, 1H). **^{13}C{^{1}H} NMR (100 MHz, Chloroform-*d*)** δ 154.6 (dd, J_{CF} = 293.6, 288.8 Hz), 139.7 (t, J_{CF} = 4.3 Hz), 138.9, 129.1 (t, J_{CF} = 3.8 Hz), 127.6, 110.2, 90.5 (dd, J_{CF} = 23.0, 11.7 Hz), 64.8, 64.7, 44.5, 42.3, 34.3, 28.7, 25.9, 24.4, 23.7. **^{19}F NMR (377 MHz, Chloroform-*d*)** δ –87.31 (d, J_{FF} = 35.9 Hz), –87.63 (d, J_{FF} = 35.8 Hz). **HRMS (ESI)** *m/z*: [M+Na]$^+$ Calcd for $C_{18}H_{22}F_2O_4SNa$ 395.1099; Found 395.1092.

6-(3,3-Difluoro-2-(naphthalen-2-yl)allyl)-1,4-dioxaspiro[4.5]decane (Scheme 2, product 9j)

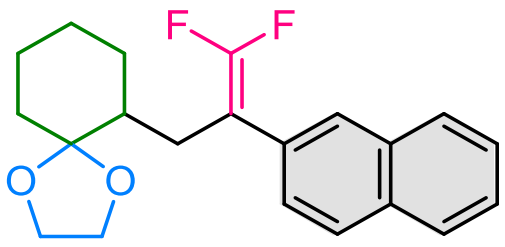

Colorless oily liquid (76%, 26.1 mg) was obtained. EtOAc: Hexane = 1:10, R_f = 0.25. **^{1}H NMR (400 MHz, Chloroform-*d*)** δ 7.89 – 7.74 (m, 4H), 7.56 – 7.43 (m, 3H), 4.06 – 3.80 (m, 4H), 2.94 – 2.76 (m, 1H), 2.53 – 2.31 (m, 1H), 1.81 – 1.72 (m, 2H), 1.66 – 1.57 (m, 2H), 1.52 – 1.43 (m, 1H), 1.39 – 1.19 (m, 3H), 1.16 – 1.04 (m, 1H). **^{13}C{^{1}H} NMR (125 MHz, Chloroform-*d*)** δ 154.4 (dd, J_{CF} = 288.9, 288.3 Hz), 133.4, 132.5, 131.1, 128.1(4), 128.1(2), 127.7, 127.5 (t, J_{CF} = 3.7 Hz), 126.3, 126.2 (d, J_{CF} = 3.4 Hz), 126.2, 110.6, 91.2 (dd, J_{CF} = 19.5, 14.9 Hz), 64.9, 64.8, 42.3, 34.5, 28.6, 26.2, 24.4, 23.9. **^{19}F NMR (377 MHz, Chloroform-*d*)** δ –90.37 (d, J_{FF} = 43.4 Hz), –90.49 (d, J_{FF} = 43.3 Hz). **HRMS (APCI)** *m/z*: $[M+H]^+$ Calcd for $C_{21}H_{23}F_2O_2$ 345.1661; Found 345.1658.

4-(1,1-Difluoro-3-(1,4-dioxaspiro[4.5]decan-6-yl)prop-1-en-2-yl)dibenzo[*b,d*]furan (Scheme 2, product 9k)

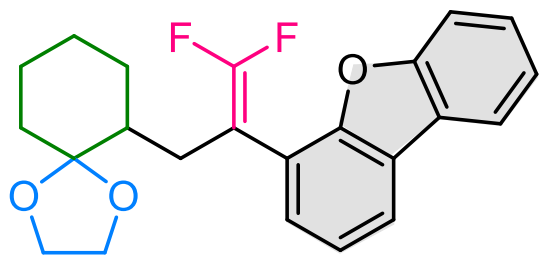

Colorless oily liquid (75%, 28.8 mg) was obtained. EtOAc: Hexane = 1:20, R_f = 0.20. **^{1}H NMR (400 MHz, Chloroform-*d*)** δ 7.99 – 7.94 (m, 1H), 7.93 – 7.84 (m, 1H), 7.63 – 7.54 (m, 1H), 7.52 – 7.42 (m, 1H), 7.42 – 7.29 (m, 3H), 4.06 – 3.60 (m, 4H), 3.01 – 2.79 (m, 1H), 2.54 – 2.35 (m, 1H), 1.91 – 1.82 (m, 1H), 1.77 – 1.67 (m, 1H), 1.65 – 1.50 (m, 3H), 1.50 – 1.40 (m, 1H), 1.34 (m, 1H), 1.26 – 1.18 (m, 1H), 1.15 – 1.03 (m, 1H). **^{13}C{^{1}H} NMR (100 MHz, Chloroform-*d*)** δ 156.2, 154.0 (dd, J_{CF} = 286.7, 281.4 Hz), 127.8 (t, J_{CF} = 2.5 Hz), 127.4, 124.7, 124.3, 122.9(0), 122.8(8), 120.8, 120.1, 118.5 (dd, J_{CF} = 5.1, 2.1 Hz), 111.9, 110.5, 86.9 (dd, J_{CF} = 24.8, 15.6 Hz), 64.9, 64.7, 42.5, 34.6, 28.7, 26.7 (d, J_{CF} = 1.5 Hz), 24.4, 23.9. **^{19}F NMR (377 MHz, Chloroform-*d*)** δ –86.85 (d, J_{FF} = 38.8 Hz), –91.44 (d, J_{FF} = 38.8 Hz). **HRMS (ESI)** *m/z*: $[M+Na]^+$ Calcd for $C_{23}H_{22}F_2O_3Na$ 407.1429; Found 407.1428.

6-(2-(Benzo[*b*]thiophen-2-yl)-3,3-difluoroallyl)-1,4-dioxaspiro[4.5]decane (Scheme 2, product 9l)

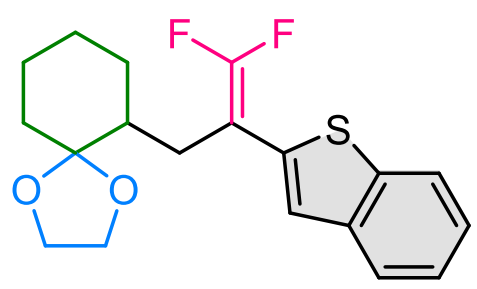

Colorless oily liquid (55%, 19.3 mg) was obtained. EtOAc: Hexane = 1:20, R_f = 0.30. **^{1}H NMR (500 MHz, Chloroform-*d*)** δ 7.79 (d, J = 7.9 Hz, 1H), 7.75 (d, J = 7.5 Hz, 1H), 7.36 – 7.27 (m, 3H), 4.21 – 3.76 (m, 4H), 2.97 – 2.67 (m, 1H), 2.42 – 2.33 (m, 1H), 1.98 – 1.89 (m, 1H), 1.86 – 1.80 (m, 1H), 1.80

– 1.72 (m, 1H), 1.68 – 1.61 (m, 2H), 1.54 – 1.44 (m, 1H), 1.41 – 1.30 (m, 2H), 1.21 – 1.10 (m, 1H). **$^{13}C\{^1H\}$ NMR (125 MHz, Chloroform-*d*)** *δ* 154.7 (dd, J_{CF} = 297.0, 288.8 Hz), 139.7, 139.5 (d, J_{CF} = 6.3 Hz), 136.4 (dd, J_{CF} = 7.7, 3.8 Hz), 124.5, 124.4, 123.5, 122.2 (dd, J_{CF} = 6.7, 4.4 Hz), 122.0, 110.6, 87.8 (dd, J_{CF} = 26.6, 11.7 Hz), 65.0, 64.9, 43.1, 34.7, 28.9, 26.4 (d, J_{CF} = 2.1 Hz), 24.7, 23.9. **^{19}F NMR (471 MHz, Chloroform-*d*)** *δ* –82.38 (d, J_{FF} = 30.0 Hz), –87.98 (d, J_{FF} = 30.2 Hz). **HRMS (APCI)** *m/z*: $[M+H]^+$ Calcd for $C_{19}H_{21}F_2O_2S$ 351.1225; Found 351.1220.

8-(1,1-Difluoro-3-(1,4-dioxaspiro[4.5]decan-6-yl)prop-1-en-2-yl)quinoline (Scheme 2, product 9m)

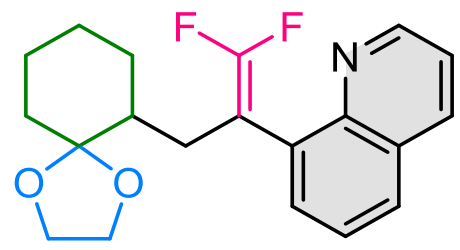

Yellow oily liquid (17%, 5.9 mg) was obtained. EtOAc: Hexane: MeOH = 100:20:1, R_f = 0.20. **^1H NMR (400 MHz, Chloroform-*d*)** *δ* 8.94 (d, *J* = 4.2 Hz, 1H), 8.15 (dd, *J* = 8.3, 1.8 Hz, 1H), 7.79 (dd, *J* = 8.0, 1.6 Hz, 1H), 7.58 (dd, *J* = 7.1, 1.6 Hz, 1H), 7.52 (t, *J* = 7.6 Hz, 1H), 7.40 (dd, *J* = 8.3, 4.2 Hz, 1H), 3.95 – 3.80 (m, 4H), 2.99 – 2.89 (m, 1H), 2.57 – 2.46 (m, 1H), 1.97 – 1.89 (m, 1H), 1.72 – 1.53 (m, 2H), 1.48 – 1.40 (m, 2H), 1.37 – 1.31 (m, 1H), 1.30 – 1.23 (m, 1H), 1.20 – 1.06 (m, 2H). **$^{13}C\{^1H\}$ NMR (100 MHz, Chloroform-*d*)** *δ* 150.9 (dd, J_{CF} = 288.5, 286.8 Hz), 150.0, 136.4, 131.1 (t, J_{CF} = 2.0 Hz), 128.7, 128.1, 126.1, 121.2, 110.5, 89.7 (dd, J_{CF} = 23.6, 16.2 Hz), 64.8, 64.7, 42.6 34.8, 28.8, 27.8, 24.6, 23.9. **^{19}F NMR (377 MHz, Chloroform-*d*)** δ -89.42 (d, J_{FF} = 44.7 Hz), -93.14 (d, J_{FF} = 44.8 Hz). **HRMS (ESI)** *m/z*: $[M+H]^+$ Calcd for $C_{20}H_{22}F_2NO_2$ 346.1613; Found 346.1610.

6-(2-([1,1'-Biphenyl]-4-yl)-3,3-difluoroallyl)-1,4-dioxaspiro[4.4]nonane (Scheme 2, product 9n)

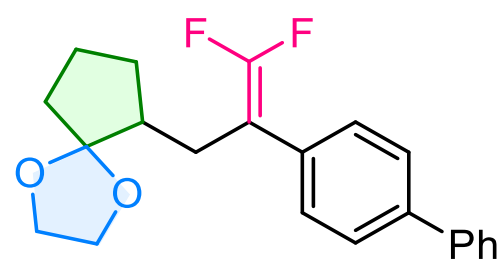

Colorless oily liquid (50%, 17.9 mg) was obtained. EtOAc: Hexane = 1:25, R_f = 0.30. **^1H NMR (400 MHz, Chloroform-*d*)** *δ* 7.63 – 7.57 (m, 4H), 7.48 – 7.33 (m, 5H), 3.99 – 3.82 (m, 4H), 2.68 – 2.55 (m, 1H), 2.49 – 2.38 (m, 1H), 2.05 – 1.94 (m, 1H), 1.83 – 1.51 (m, 5H), 1.45 – 1.34 (m, 1H). **$^{13}C\{^1H\}$ NMR (100 MHz, Chloroform-*d*)** *δ* 154.0 (dd, J_{CF} = 286.1, 285.9 Hz), 140.7, 140.1, 132.8, 128.9, 128.8 (t, J_{CF} = 3.3 Hz), 127.5, 127.2, 127.1, 117.9, 91.3 (dd, J_{CF} = 19.2, 15.5 Hz), 64.7, 64.6, 44.2, 35.6, 28.9, 26.7, 20.4. **^{19}F NMR (377 MHz, Chloroform-*d*)** *δ* –90.87 (d, J_{FF} = 43.2 Hz), –91.00 (d, J_{FF} = 43.2 Hz). **HRMS (APCI)** *m/z*: $[M+H]^+$ Calcd for $C_{22}H_{23}F_2O_2$ 357.1661; Found 357.1655.

6-(2-([1,1'-Biphenyl]-4-yl)-3,3-difluoroallyl)-1,4,9,12-tetraoxadispiro[4.2.4^8.2^5]tetradecane (Scheme 2, product 9o)

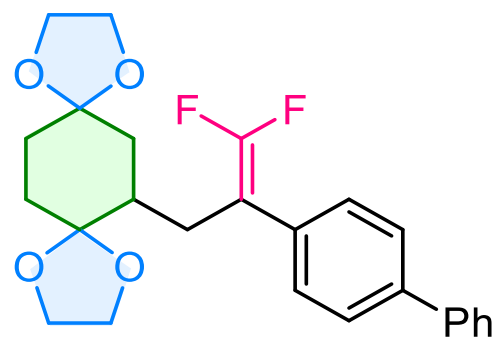

Colorless oily liquid (76%, 32.6 mg) was obtained. EtOAc: Hexane = 1:9, R$_f$ = 0.20. **^{1}H NMR (500 MHz, Chloroform-*d*)** δ 7.62 – 7.57 (m, 4H), 7.47 – 7.40 (m, 4H), 7.37 – 7.33 (m, 1H), 4.01 – 3.98 (m, 3H), 3.95 (s, 1H), 3.93 – 3.87 (m, 2H), 3.87 – 3.81 (m, 1H), 3.80 – 3.75 (m, 1H), 2.77 – 2.68 (m, 1H), 2.39 – 2.31 (m, 1H), 2.08 – 2.01 (m, 1H), 1.81 – 1.76 (m, 3H), 1.68 – 1.60 (m, 3H). **^{13}C{^{1}H} NMR (125 MHz, Chloroform-*d*)** δ 154.3 (dd, J_{CF} = 290.5, 287.3 Hz), 140.7, 140.0, 132.5 (t, J_{CF} = 3.9 Hz), 128.9, 128.7 (t, J_{CF} = 3.5 Hz), 127.5, 127.3, 127.1, 109.7, 108.2, 90.4 (dd, J_{CF} = 21.5, 12.8 Hz), 65.1, 65.0, 64.5, 64.2, 39.8, 36.9, 32.4, 31.8, 26.0. **^{19}F NMR (471 MHz, Chloroform-*d*)** δ –89.71 (dd, J_{FF} = 42.2, 4.7 Hz), –90.07 (d, J_{FF} = 42.0 Hz). **HRMS (ESI)** *m/z*: [M+Na]$^+$ Calcd for $C_{25}H_{26}F_2O_4Na$ 451.1691; Found 451.1686.

6-(2-([1,1'-Biphenyl]-4-yl)-3,3-difluoroallyl)-1,4,8-trioxaspiro[4.5]decane (Scheme 2, product 9p)

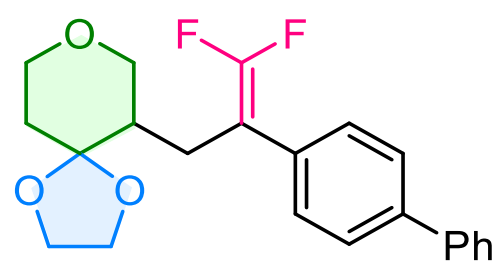

Yellow oily liquid (87%, 32.4 mg) was obtained. EtOAc: Hexane = 1:9, R$_f$ = 0.25. **^{1}H NMR (500 MHz, Chloroform-*d*)** δ 7.62 – 7.57 (m, 4H), 7.48 – 7.40 (m, 4H), 7.39 – 7.32 (m, 1H), 4.04 – 3.94 (m, 4H), 3.83 – 3.74 (m, 2H), 3.71 – 3.63 (m, 1H), 3.48 – 3.41 (m, 1H), 2.74 – 2.67 (m, 1H), 2.45 – 2.36 (m, 1H), 1.92 – 1.87 (m, 1H), 1.80 – 1.74 (m, 1H), 1.64 – 1.55 (m, 1H). **^{13}C{^{1}H} NMR (125 MHz, Chloroform-*d*)** δ 154.1 (dd, J_{CF} = 291.8, 287.3 Hz), 140.7, 140.3, 132.1 (t, J_{CF} = 3.6 Hz), 128.9, 128.7 (t, J_{CF} = 3.6 Hz), 127.5, 127.4, 127.2, 107.9, 90.2 (dd, J_{CF} = 21.4, 13.4 Hz), 69.2, 66.3, 64.9(1), 64.8(6), 42.4, 34.9, 22.9. **^{19}F NMR (471 MHz, Chloroform-*d*)** δ –89.55 (d, J_{FF} = 41.0 Hz), –89.77 (d, J_{FF} = 41.0 Hz). **HRMS (APCI)** *m/z*: [M+H]$^+$ Calcd for $C_{22}H_{23}F_2O_3$ 373.1610; Found 373.1606.

Methyl 4-(1,1-difluoro-3-(1,4,8-trioxaspiro[4.5]decan-6-yl)prop-1-en-2-yl)benzoate (Scheme 2, product 9q)

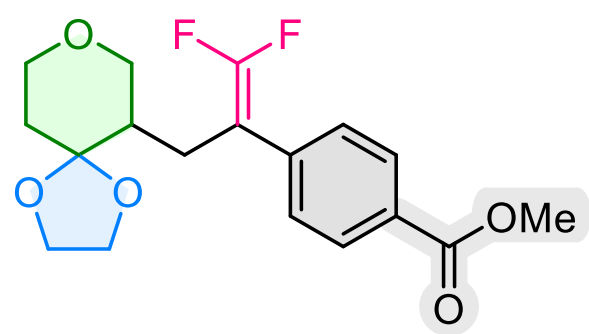

Colorless oily liquid (66%, 23.4 mg) was obtained. EtOAc: Hexane = 1:9, R_f = 0.25. **^{1}H NMR (400 MHz, Chloroform-*d*)** δ 8.02 (d, J = 8.5 Hz, 2H), 7.42 (d, J = 8.2 Hz, 2H), 4.01 – 3.96 (m, 2H), 3.96 – 3.92 (m, 2H), 3.92 (s, 3H), 3.80 – 3.63 (m, 3H), 3.45 – 3.35 (m, 1H), 2.74 – 2.64 (m, 1H), 2.45 – 2.33 (m, 1H), 1.82 – 1.70 (m, 2H), 1.60 – 1.52 (m, 1H). **^{13}C{^{1}H} NMR (100 MHz, Chloroform-*d*)** δ 166.7, 154.2 (dd, J_{CF} = 290.3, 289.9 Hz), 138.0, 129.8, 129.0, 128.2 (t, J_{CF} = 3.6 Hz), 107.6, 90.3 (dd, J_{CF} = 19.3, 15.5 Hz), 68.9, 66.1, 64.8, 64.7, 52.2, 42.3, 34.7, 22.7. **^{19}F NMR (377 MHz, Chloroform-*d*)** δ –87.84 (d, J_{FF} = 36.9 Hz), –87.95 (d, J_{FF} = 36.9 Hz). **HRMS (ESI)** *m/z*: [M+Na]$^+$ Calcd for $C_{18}H_{20}F_2O_5Na$ 377.1171; Found 377.1165.

2-(5-([1,1'-Biphenyl]-4-yl)-6,6-difluorohex-5-en-3-yl)-2-propyl-1,3-dioxolane (Scheme 2, product 9r)

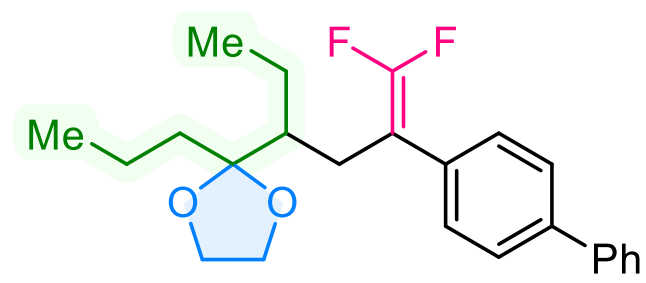

Colorless oily liquid (35%, 13.5 mg) was obtained. EtOAc: Hexane = 1:50, R_f = 0.20. **^{1}H NMR (400 MHz, Chloroform-*d*)** δ 7.65 – 7.57 (m, 4H), 7.49 – 7.39 (m, 4H), 7.39 – 7.31 (m, 1H), 3.95 – 3.68 (m, 4H), 2.76 – 2.65 (m, 1H), 2.44 – 2.33 (m, 1H), 1.67 – 1.48 (m, 4H), 1.38 – 1.24 (m, 3H), 0.98 – 0.81 (m, 6H). **^{13}C{^{1}H} NMR (100 MHz, Chloroform-*d*)** δ 153.7 (dd, J_{CF} = 290.1, 286.3 Hz), 140.7, 139.9, 132.6 (t, J_{CF} = 3.5 Hz), 128.9 (d, J_{CF} = 3.5 Hz), 127.5, 127.1(1), 127.0(9), 113.9, 91.9 (dd, J_{CF} = 21.0, 13.2 Hz), 65.1, 65.0, 43.7, 36.1, 27.7, 23.0, 16.3, 14.5, 12.8. **^{19}F NMR (377 MHz, Chloroform-*d*)** δ –90.97 (d, J_{FF} = 43.8 Hz), –91.20 (d, J_{FF} = 43.9 Hz). **HRMS (APCI)** *m/z*: [M+H]$^+$ Calcd for $C_{24}H_{29}F_2O_2$ 387.2130; Found 387.2123.

6-(2-([1,1'-Biphenyl]-4-yl)-3,3-difluoroallyl)-6-methyl-1,4-dioxaspiro[4.5]decane dioxolane (Scheme 2, product 9s)

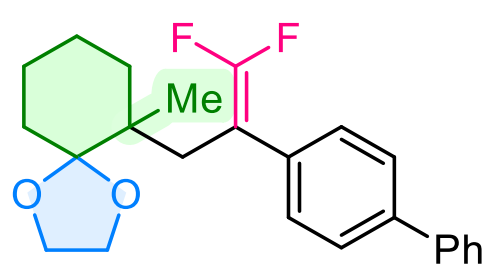

Colorless oily liquid (39%, 15.1 mg) was obtained. EtOAc: Hexane = 1:20, R_f = 0.25. **^{1}H NMR (500 MHz, Chloroform-*d*)** δ 7.73 – 7.48 (m, 4H), 7.50 – 7.36 (m, 4H), 7.37 – 7.30 (m, 1H), 4.06 – 3.78 (m, 4H), 2.71 – 2.54 (m, 1H), 2.14 – 1.90 (m, 1H), 1.63 – 1.42 (m, 4H), 1.38 – 1.12 (m, 4H), 0.86 (s, 3H). **^{13}C{^{1}H} NMR (125 MHz, Chloroform-*d*)** δ 154.6 (dd, J_{CF} = 290.7, 286.1 Hz), 140.7, 139.7, 135.0 (t, J_{CF} = 4.1 Hz), 128.9 (t, J_{CF} = 8.6 Hz), 127.5, 127.1, 127.0, 112.6, 90.3 (dd, J_{CF} = 14.8, 7.0 Hz), 65.0(3), 64.9(6), 43.5, 35.0, 32.2, 30.5, 23.6, 20.8, 20.0. **^{19}F NMR (471 MHz, Chloroform-*d*)** δ -88.69 (d, J_{FF}

= 39.8 Hz), -91.01 (d, J_{FF} = 39.8 Hz). **HRMS (ESI)** *m/z*: $[M+H]^+$ Calcd for $C_{24}H_{27}F_2O_2$ 385.1974; Found 385.1970.

2-(3-([1,1'-Biphenyl]-4-yl)-3-(2,2-difluorovinyl)-1-hexylpropyl)-4,4,7,7-tetramethyl-1,3-dioxepane (Scheme 2, product 10a)

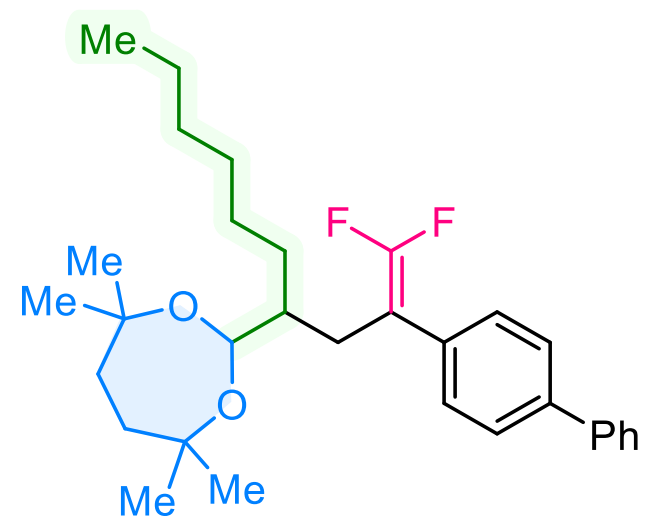

Colorless oily liquid (71%, 34.4 mg) was obtained. EtOAc: Hexane = 1:50, R_f = 0.20. **^{1}H NMR (400 MHz, Chloroform-*d*)** δ 7.65 – 7.54 (m, 4H), 7.50 – 7.41 (m, 4H), 7.40 – 7.32 (m, 1H), 4.60 (d, *J* = 5.5 Hz, 1H), 2.82 – 2.71 (m, 1H), 2.36 – 2.24 (m, 1H), 1.84 – 1.62 (m, 5H), 1.31 – 1.15 (m, 22H), 0.88 (t, *J* = 6.9 Hz, 3H). **^{13}C{^{1}H} NMR (100 MHz, Chloroform-*d*)** δ 153.9 (t, J_{CF} = 285.0, 284.0 Hz), 140.9, 139.9, 132.6 (t, J_{CF} = 2.8 Hz), 129.0 (t, J_{CF} = 3.4 Hz), 128.9, 127.4, 127.2, 127.1, 92.9, 91.6 (dd, J_{CF} = 20.1, 13.6 Hz), 74.6, 74.5, 41.6, 35.7(3), 35.6(7), 31.9, 30.3, 30.1, 29.9, 29.2, 27.2, 26.6, 26.1, 25.7, 22.8, 14.2. **^{19}F NMR (377 MHz, Chloroform-*d*)** δ –90.75 (d, J_{FF} = 44.2 Hz), –90.91 (d, J_{FF} = 44.1 Hz). **HRMS (ESI)** *m/z*: $[M+Na]^+$ Calcd for $C_{31}H_{42}F_2O_2Na$ 507.3045; Found 507.3041.

2-(5-([1,1'-Biphenyl]-4-yl)-6,6-difluoro-2-methylhex-5-en-3-yl)-4,4,7,7-tetramethyl-1,3-dioxepane (Scheme 2, product 10b)

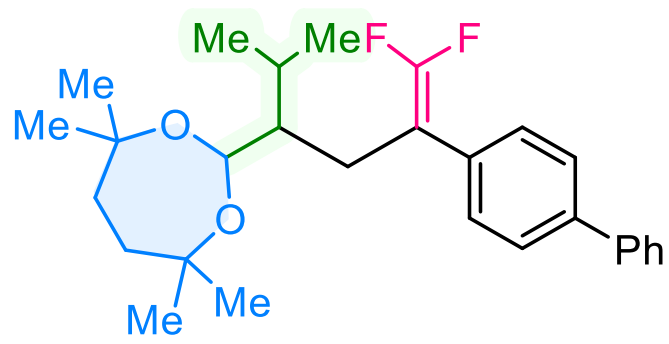

Colorless oily liquid (70%, 31.0 mg) was obtained. EtOAc: Hexane = 1:50, R_f = 0.30. **^{1}H NMR (400 MHz, Chloroform-*d*)** δ 7.63 – 7.53 (m, 4H), 7.48 – 7.40 (m, 4H), 7.38 – 7.32 (m, 1H), 4.64 (d, *J* = 6.0 Hz, 1H), 2.72 – 2.63 (m, 1H), 2.44 – 2.33 (m, 1H), 2.01 – 1.85 (m, 1H), 1.80 – 1.63 (m, 4H), 1.47 – 1.38 (m, 1H), 1.20 (m, 12H), 0.90 (m, 6H). **^{13}C{^{1}H} NMR (100 MHz, Chloroform-*d*)** δ 153.3 (dd, J_{CF} = 289.0, 285.5 Hz), 141.0, 139.8, 132.6 (t, J_{CF} = 3.9 Hz), 129.2 (t, J_{CF} = 3.2 Hz), 128.9, 127.3, 127.2, 127.0, 92.6, 92.5 (dd, J_{CF} = 21.3, 12.6 Hz), 74.7, 74.6, 46.4, 35.8, 35.7, 30.4, 30.3, 27.3, 26.0, 25.8, 24.1, 20.3, 19.3. **^{19}F NMR (377 MHz, Chloroform-*d*)** δ –91.10 (d, J_{FF} = 45.2 Hz), –91.75 (d, J_{FF} = 45.1 Hz). **HRMS (ESI)** *m/z*: $[M+Na]^+$ Calcd for $C_{28}H_{36}F_2O_2Na$ 465.2576; Found 465.2581.

2-(4-([1,1'-Biphenyl]-4-yl)-5,5-difluoropent-4-en-2-yl)-4,4,7,7-tetramethyl-1,3-dioxepane (Scheme 2, product 10c)

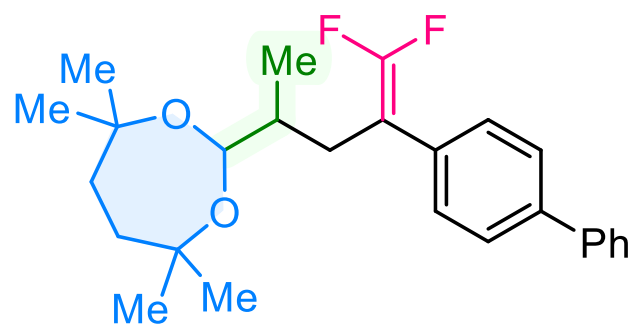

Colorless oily liquid (92%, 38.1 mg) was obtained. EtOAc: Hexane = 1:50, R_f = 0.40. **^{1}H NMR (400 MHz, Chloroform-*d*)** δ 7.62 – 7.52 (m, 4H), 7.48 – 7.39 (m, 4H), 7.39 – 7.30 (m, 1H), 4.43 (d, *J* = 6.5 Hz, 1H), 2.83 – 2.72 (m, 1H), 2.17 – 2.07 (m, 1H), 1.79 – 1.59 (m, 5H), 1.27 – 1.16 (m, 12H), 0.85 (d, *J* = 6.8 Hz, 3H). **^{13}C{^{1}H} NMR (125 MHz, Chloroform-*d*)** δ 154.9 (dd, J_{CF} = 287.5, 283.8 Hz), 140.9, 140.0, 132.5 (t, J_{CF} = 2.5 Hz), 128.9, 128.8 (t, J_{CF} = 3.6 Hz), 127.4, 127.2(1), 127.1(8), 94.7, 91.1 (dd, J_{CF} = 20.6, 13.3 Hz), 74.7, 37.0, 35.7(2), 35.7(0), 30.2, 30.1, 29.9, 26.0, 14.9. **^{19}F NMR (377 MHz, Chloroform-*d*)** δ –90.69 (d, J_{FF} = 43.6 Hz), –90.86 (d, J_{FF} = 43.7 Hz). **HRMS (ESI)** *m/z*: [M+Na]$^+$ Calcd for $C_{26}H_{32}F_2O_2Na$ 437.2263; Found 437.2258.

2-(4-([1,1'-Biphenyl]-4-yl)-5,5-difluoro-1-phenylpent-4-en-2-yl)-4,4,7,7-tetramethyl-1,3-dioxepane (Scheme 2, product 10d)

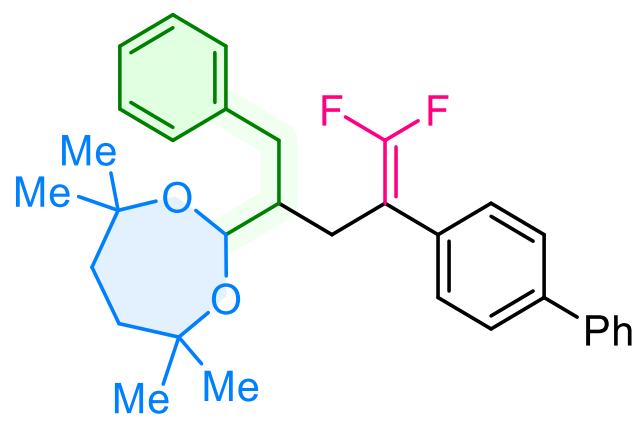

Colorless oily liquid (85%, 41.7 mg) was obtained. EtOAc: Hexane = 1:20, R_f = 0.50. **^{1}H NMR (400 MHz, Chloroform-*d*)** δ 7.65 – 7.59 (m, 2H), 7.54 – 7.43 (m, 4H), 7.41 – 7.33 (m, 1H), 7.27 – 7.13 (m, 5H), 7.11 – 7.05 (m, 2H), 4.61 (d, *J* = 4.2 Hz, 1H), 2.88 (m, 1H), 2.82 – 2.72 (m, 1H), 2.48 (m, 1H), 2.43 – 2.32 (m, 1H), 1.93 – 1.54 (m, 5H), 1.33 – 1.21 (m, 6H), 1.18 – 1.00 (m, 6H). **^{13}C{^{1}H} NMR (125 MHz, Chloroform-*d*)** δ 153.5 (dd, J_{CF} = 290.0, 285.8 Hz), 141.2, 140.9, 139.7, 132.3 (t, J_{CF} = 3.0 Hz), 129.2, 128.9 (t, J_{CF} = 2.6 Hz), 128.2, 127.4, 127.1, 126.9, 125.8, 92.2, 91.4 (dd, J_{CF} = 21.4, 12.5 Hz), 74.7, 74.6, 43.7, 36.3, 35.8, 35.6, 30.3, 30.1, 27.0, 25.9(1), 25.8(6). **^{19}F NMR (377 MHz, Chloroform-*d*)** δ –89.98 (d, J_{FF} = 43.3 Hz), –91.16 (d, J_{FF} = 43.1 Hz). **HRMS (ESI)** *m/z*: [M+ Na]$^+$ Calcd for $C_{32}H_{36}F_2O_2Na$ 513.2576; Found 513.2570.

2-(2-([1,1'-Biphenyl]-4-yl)-1,1-difluorohepta-1,6-dien-4-yl)-4,4,7,7-tetramethyl-1,3-dioxepane (Scheme 2, product 10e)

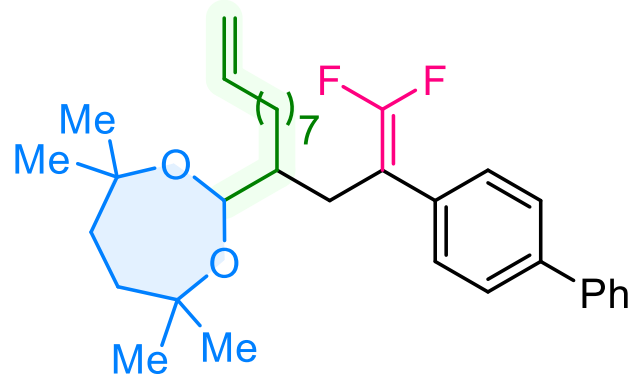

Colorless oily liquid (22%, 11.5 mg) was obtained. EtOAc: Hexane = 1:50, R_f = 0.20. **^{1}H NMR (400 MHz, Chloroform-*d*)** δ 7.62 – 7.53 (m, 4H), 7.49 – 7.40 (m,4H), 7.38 – 7.32 (m, 1H), 5.89 – 5.73 (m, 1H), 5.03 – 4.89 (m, 2H), 4.58 (d, *J* = 5.5 Hz, 1H), 2.79 – 2.69 (m, 1H), 2.33 – 2.19 (m, 1H), 2.07 – 1.98 (m, 2H), 1.82 – 1.63 (m, 4H), 1.30 – 1.12 (m, 25H). **^{13}C{^{1}H} NMR (100 MHz, Chloroform-*d*)** δ 153.9 (dd, J_{CF} = 289.5, 286.5 Hz), 140.9, 139.9, 139.4, 132.6 (t, J_{CF} = 2.7 Hz), 129.0 (t, J_{CF} = 3.5 Hz), 128.9, 127.4, 127.2, 127.1, 114.2, 92.9, 91.6 (dd, J_{CF} = 20.1, 13.6 Hz), 74.6, 74.5, 41.6, 35.7(3), 35.6(8), 34.0, 30.4, 30.1(2), 30.0(9), 29.5, 29.2(4), 29.2(0), 29.1, 27.2, 26.7, 26.2, 25.7. **^{19}F NMR (377 MHz, Chloroform-*d*)** δ –90.75 (d, J_{FF} = 44.1 Hz), –90.91 (d, J_{FF} = 43.8 Hz). **HRMS (ESI)** *m/z*: [M+ Na]$^+$ Calcd for $C_{34}H_{46}F_2O_2Na$ 547.3358; Found 547.3360.

7. General Procedures for Scale-up Synthesis of Product 10c

To a flame-dried tube equipped with a stir bar were added the Fe(bipy)Cl_3 (198.9 mg, 25 mol %), Zn (487.5 mg, 7.5 mmol), CF_3-alkene **7a** (620.6 mg, 2.5 mmol) and acetal-alkyl iodide **8t** (1.95 g, 6.25 mmol). The system was further evacuated and flushed with nitrogen for three cycles. DMA (37.5 mL) and TMSCl (635 μL) were added with stirring at room temperature for several minutes. The tube was then placed into a pre-heated oil bath (60 °C) and stirred for 18 h. After completion of reaction, the reaction tube was allowed to cool to room temperature. Ethyl acetate (~30 mL) and water (~30 mL) were added and the organic layer was subjected to GC analysis. The aqueous layer was extracted by ethyl acetate. The combined organic layer was concentrated in *vacuo*. The crude product **10c** (0.93 g, 90%) was purified by column chromatography on silica gel.

8. Derivatization of Products

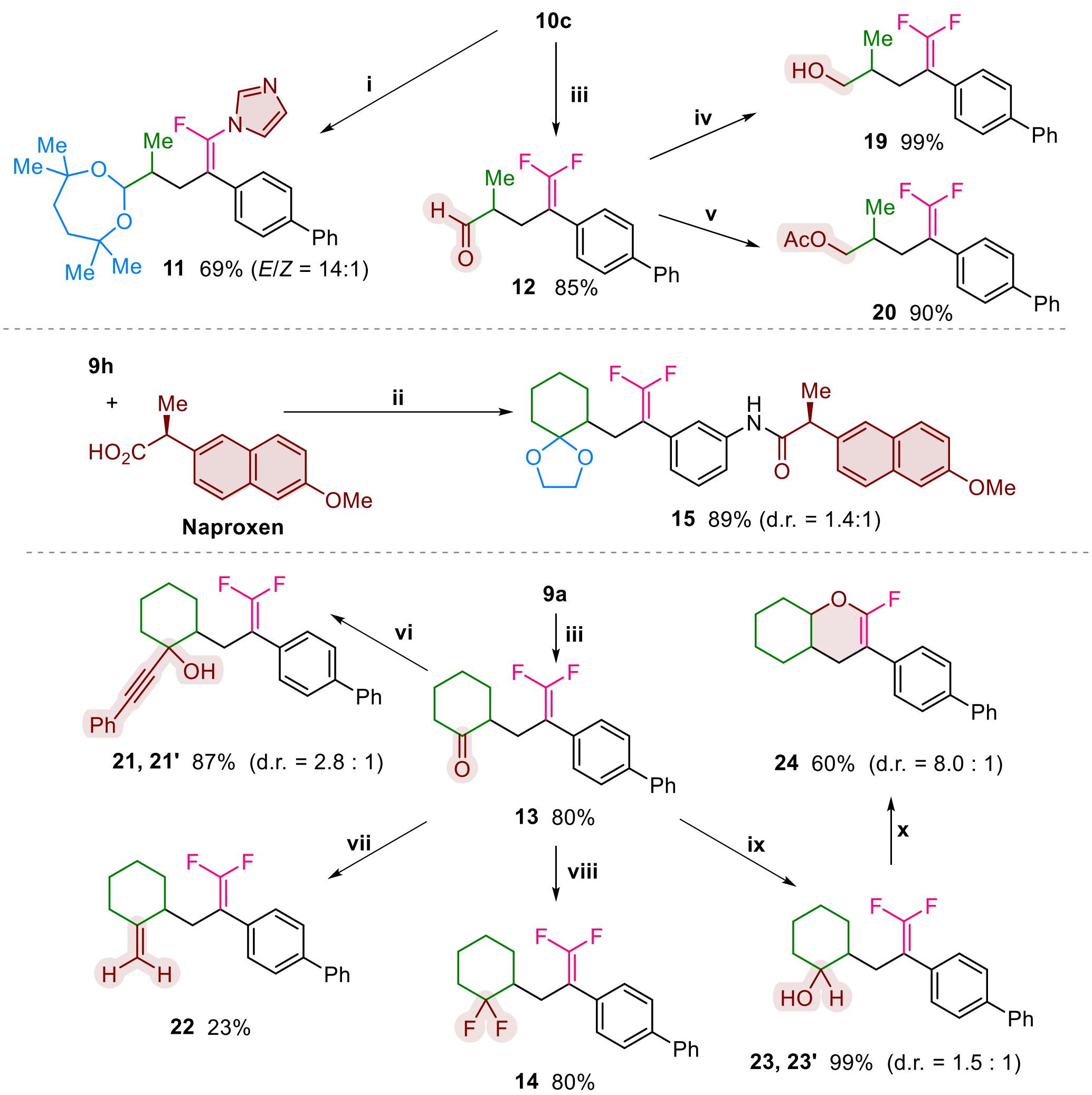

Figure S3. Derivatization of cyclic acetal-*gem*-difluoroalkene products. Reaction contitions: **(i)** imidazole, K_3PO_4, DMF; **(ii)** $(C(O)Cl)_2$, DMF/CH_2Cl_2, 0 °C to rt, 2 h, then Et_3N, CH_2Cl_2, 0 °C to rt, overnight; **(iii)** HCl (aq), acetone, rt; **(iv)** $NaBH_4$, MeOH; **(v)** $NaBH_4$, MeOH, 2 h; then AcCl, Et_3N, CH_2Cl_2; **(vi)** Phenylacetylene, ^{n}BuLi, THF, -78 °C – rt; **(vii)** $Ph_3P^+MeBr^-$, ^{n}BuLi, THF, -78 – 0 °C – rt; **(viii)** DAST, DME, 80 °C; **(ix)** $NaBH_4$, MeOH, rt; **(x)** NaH, DMF, rt.

8.1. Synthesis of compound 11

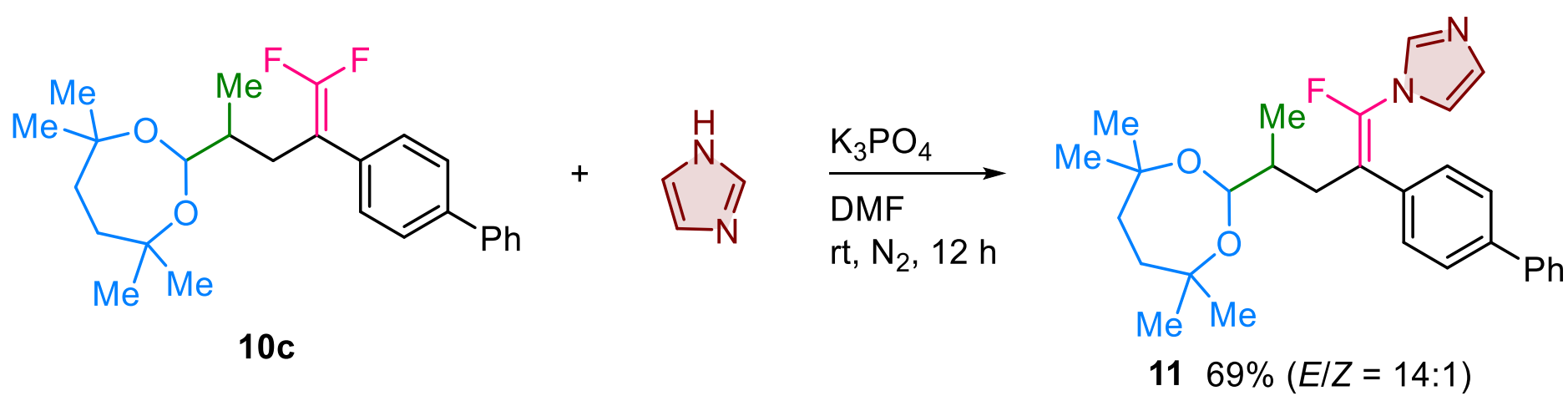

To a 10 mL Schlenk tube containing a magnetic stir bar under an atmosphere of argon were added **10c** (45.5 mg, 0.11 mmol, 1.1 equiv.), 1*H*-imidazole (6.8 mg, 0.1 mmol, 1.0 equiv.), K_3PO_4 (42.5 mg, 0.2 mmol, 2 equiv.) and DMF (2.0 mL). The resulting reaction mixture was stirred at room

temperature for 12 h. The mixture was then quenched with sat. NaCl and extracted with EtOAc (10 mL × 3). The organic layers were concentrated by evaporation. The residue was purified by preparative thin-layer chromatography (Hexane/EtOAc = 3:1) to afford product **11** (31.9 mg, 0.069 mmol, 69%) as a light yellow oil. The product was obtained as an inseparable mixture of isomers. The *Z/E* isomers ratio was determined by ^{19}F NMR analysis of the inseparable mixture of isomers (*E/Z* = 14:1).[10]

^{1}H NMR (400 MHz, Chloroform-*d*) δ 7.55 – 7.49 (m, 2H), 7.47 – 7.37 (m, 5H), 7.37 – 7.28 (m, 1H), 7.11 – 7.05 (m, 2H), 6.99 (s, 1H), 6.91 (s, 1H), 4.46 (d, *J* = 6.5 Hz, 1H), 2.95 – 2.83 (m, 1H), 2.53 – 2.42 (m, 1H), 1.82 – 1.61 (m, 5H), 1.27 – 1.15 (m, 12H), 0.92 (d, *J* = 6.8 Hz, 3H). (Only the ^{1}H NMR signals of major *E*-isomers were shown.) **^{13}C{^{1}H} NMR (100 MHz, Chloroform-*d*)** δ 142.7 (d, J_{CF} = 258.1 Hz), 140.6 (d, J_{CF} = 24.0 Hz), 137.6, 133.7 (d, J_{CF} = 4.4 Hz), 129.5, 128.9, 128.6 (d, J_{CF} = 3.1 Hz), 127.6, 127.1, 119.0, 114.5 (d, J_{CF} = 24.0 Hz), 94.6, 74.7, 36.7 (d, J_{CF} = 2.3 Hz), 35.6, 32.6, 30.1 (d, J_{CF} = 9.4 Hz), 26.0, 15.2. (Only the ^{13}C NMR signals of major *E*-isomers were shown.) **^{19}F NMR (377 MHz, Chloroform-*d*)** δ –88.73 (*Z*), –93.45 (*E*). **HRMS (ESI)** *m/z*: [M+H]$^+$ Calcd for $C_{29}H_{36}FN_2O_2$ 463.2755; Found 463.2756.

8.2. Acid-promoted hydrolysis of 10c

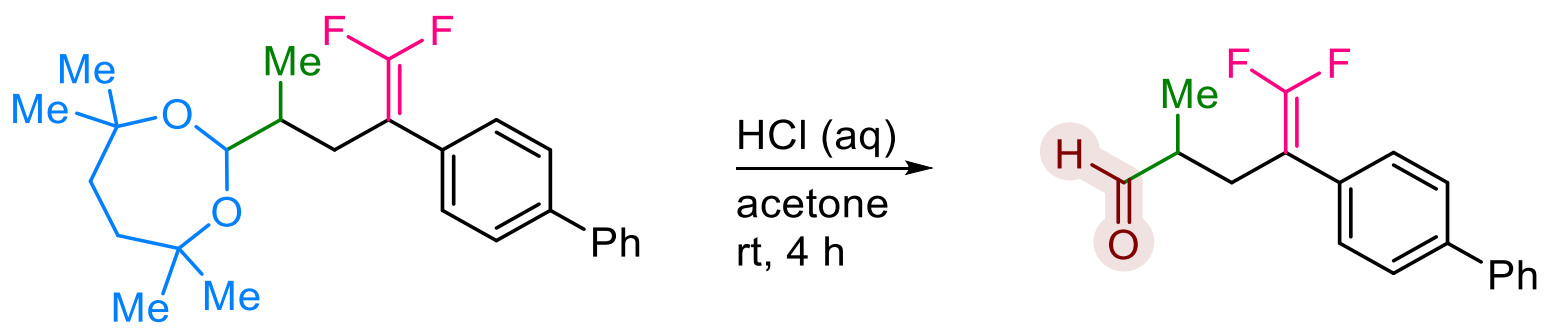

10c **12** 85%

To a 50 mL round-bottom flask equipped with a magnetic stir bar were added **10c** (400 mg, 1.0 mmol, 1.0 equiv.), acetone (10 mL) and concentrated HCl (excess) were successively added. The mixture was stirred at room temperature for 4 h. After completion of reaction, ethyl acetate (10 mL) and water (10 mL) were added and the organic layer was subjected to GC analysis. The aqueous layer was extracted by ethyl acetate. The combined organic layer was concentrated in *vacuo*. The crude product was purified by column chromatography on silica gel (Hexane/EtOAc = 20:1, Rf = 0.2) to afford the desired product **12** as light yellow oil (243 mg, 0.85 mmol, 85%).

^{1}H NMR (500 MHz, Chloroform-*d*) δ 9.64 (d, *J* = 1.4 Hz, 1H), 7.63 – 7.57 (m, 4H), 7.46 (t, *J* = 7.7 Hz, 2H), 7.41 – 7.34 (m, 3H), 2.93 – 2.86 (m, 1H), 2.53 – 2.39 (m, 2H), 1.13 (d, *J* = 7.0 Hz, 3H). **^{13}C{^{1}H} NMR (125 MHz, Chloroform-*d*)** δ 203.8, 154.2 (dd, J_{CF} = 291.0, 288.1 Hz), 140.7, 140.5, 131.7 (t, J_{CF} = 3.0 Hz), 129.0, 128.8 (t, J_{CF} = 3.1 Hz), 127.7, 127.5, 127.2, 89.8 (dd, J_{CF} = 21.0, 15.3 Hz), 44.6, 28.6

(d, J_{CF} = 1.7 Hz), 13.2. **^{19}F NMR (471 MHz, Chloroform-*d*)** δ –89.54 (d, J_{FF} = 40.5 Hz), –89.70 (d, J_{FF} = 40.6 Hz). **HRMS (ESI)** *m/z*: $[M+Na]^+$ Calcd for $C_{18}H_{16}F_2ONa$ 309.1061; Found 309.1060.

8.3. Acid-promoted hydrolysis of 9a

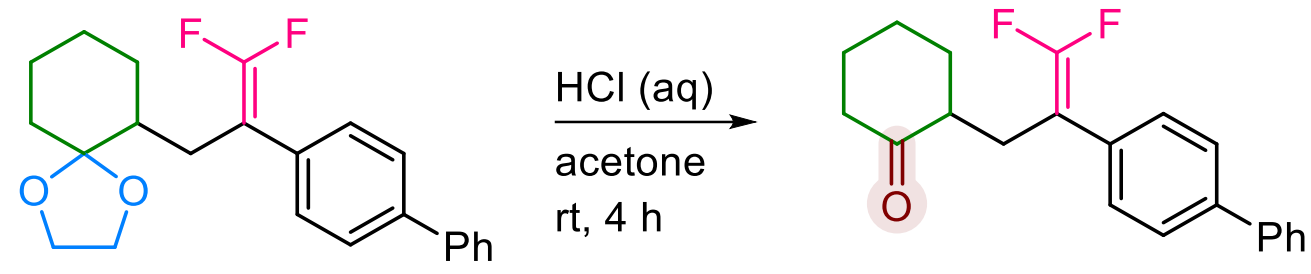

9a **13** 80%

To a 50 mL round-bottom flask equipped with a magnetic stir bar were added **9a** (600 mg, 1.6 mmol, 1.0 equiv.), acetone (10 mL) and concentrated HCl (excess). The mixture was stirred at room temperature for 4 h. After completion of reaction, ethyl acetate (~10 mL) and water (~10 mL) were added and the organic layer was subjected to GC analysis. The aqueous layer was extracted by ethyl acetate. The combined organic layer was concentrated in *vacuo*. The crude product was purified by column chromatography on silica gel (Hexane/EtOAc = 10:1, Rf = 0.5) to afford the desired product **13** as a white solid (422 mg, 1.3 mmol, 80%). Melting point 81.3 °C – 82.9 °C.

^{1}H NMR (400 MHz, Chloroform-*d*) δ 7.63 – 7.55 (m, 4H), 7.49 – 7.41 (m, 2H), 7.40 – 7.31 (m, 3H), 3.06 – 2.94 (m, 1H), 2.44 – 2.33 (m, 2H), 2.31 – 2.20 (m, 2H), 2.20 – 2.11 (m, 1H), 2.09 – 1.99 (m, 1H), 1.89 – 1.81 (m, 1H), 1.72 – 1.58 (m, 1H), 1.54 – 1.47 (m, 1H), 1.41 – 1.29 (m, 1H). **^{13}C{^{1}H} NMR (125 MHz, Chloroform-*d*)** δ 212.3, 154.2 (dd, J_{CF} = 289.9, 288.2 Hz), 140.6, 140.4, 132.1, 129.0, 128.7 (t, J_{CF} = 3.3 Hz), 127.6, 127.4, 127.1, 90.1 (dd, J_{CF} = 21.7, 13.8 Hz), 48.2, 42.1, 33.1, 27.9, 27.4, 25.0. **^{19}F NMR (377 MHz, Chloroform-*d*)** δ –89.80 (d, J_{FF} = 41.3 Hz), –90.09 (d, J_{FF} = 41.6 Hz). **HRMS (ESI)** *m/z*: $[M+H]^+$ Calcd for $C_{21}H_{21}F_2O$ 327.1555; Found 327.1555.

8.4. Synthesis of compound 14

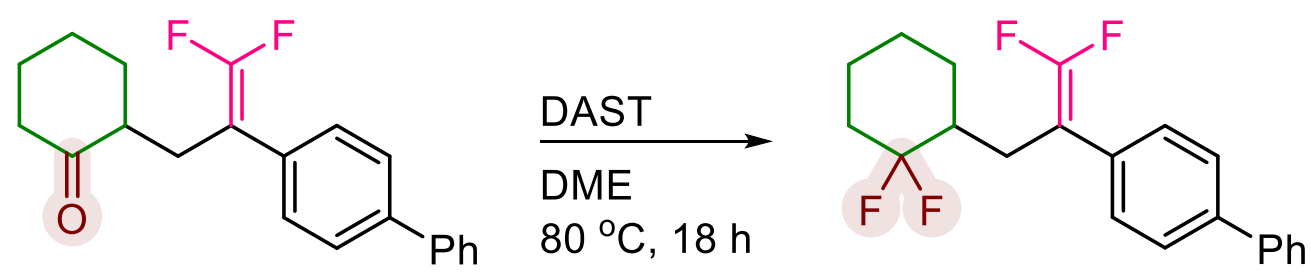

13 **14** 80%

To a 10 mL sealed tube were added **13** (32.6 mg, 0.1 mmol) and 1,2-dimethoxyethane (2 mL), followed by the addition of DAST (0.3 mL, 2.0 mmol, 20 equiv.) at -78 °C. The reaction mixture was slowly heated to 80 °C and stirred at this temperature until full conversion of **13** as indicated by TLC (about 18 h). After cooling down to room temperature, sat. $NaHCO_3$ (5 mL) was added into the reaction mixture followed by extraction with EtOAc (5 mL × 3). The combined organic phases were washed with aq. HCl (2.0 M, 2 mL), dried over Na_2SO_4, and concentrated under reduced pressure.

The residue was purified by preparative thin-layer chromatography by using PE as eluent to afford product **14** (27.9 mg, 0.080 mmol, 80%) as a colorless oil.

^{1}H NMR (500 MHz, Chloroform-*d*) δ 7.64 – 7.59 (m, 4H), 7.49 – 7.40 (m, 4H), 7.40 – 7.33 (m, 1H), 2.98 – 2.89 (m, 1H), 2.51 – 2.42 (m, 1H), 2.13 – 2.04 (m, 1H), 1.82 – 1.76 (m, 1H), 1.75 – 1.62 (m, 3H), 1.60 – 1.48 (m, 2H), 1.34 – 1.24 (m, 1H), 1.22 – 1.10 (m, 1H). **^{13}C{^{1}H} NMR (125 MHz, Chloroform-*d*)** δ 154.4 (dd, J_{CF} = 291.2, 287.5 Hz), 140.6, 140.3, 132.0 (t, J_{CF} = 3.8 Hz), 129.0, 128.7 (t, J_{CF} = 3.5 Hz), 127.6, 127.4, 127.1, 124.3 (t, J_{CF} = 244.5 Hz), 89.9 (dd, J_{CF} = 21.6, 13.8 Hz), 41.6 (dd, J_{CF} = 22.8, 20.0 Hz), 34.2 (dd, J_{CF} = 25.2, 22.3 Hz), 28.1 (d, J_{CF} = 6.7 Hz), 25.3, 24.1, 22.9 (d, J_{CF} = 9.2 Hz). **^{19}F NMR (471 MHz, Chloroform-*d*)** δ –89.59 (d, J_{FF} = 40.9 Hz), –89.90 (d, J_{FF} = 40.8 Hz), –96.59, –97.10. **HRMS (ESI)** *m/z*: [M+Na]$^+$ Calcd for $C_{21}H_{20}F_4Na$ 371.1393; Found 371.1394.

8.5. Synthesis of compound 15

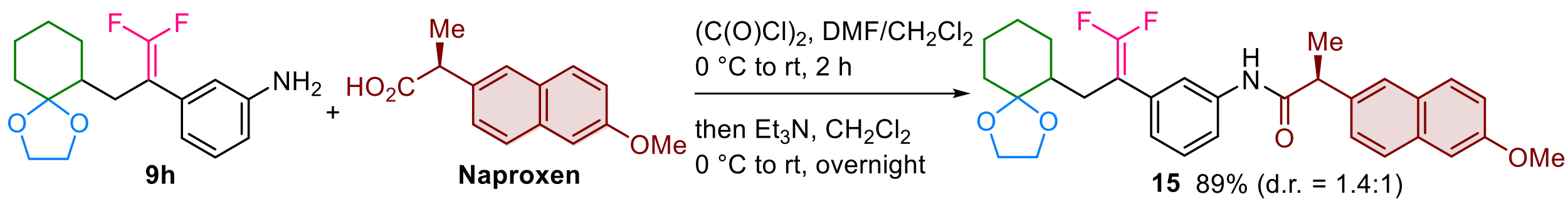

To a solution of the (*S*)-2-(6-methoxynaphthalen-2-yl)propanoic acid (Naproxen) (25.3 mg, 0.11 mmol, 1.1 equiv.) in anhydrous dichloromethane (CH_2Cl_2, 1.0 mL) was added oxalyl chloride ($(COCl)_2$, 14 µL, 0.16 mmol, 1.6 equiv.) dropwise at 0 °C under a nitrogen atmosphere, followed by a catalytic amount of anhydrous dimethylformamide (DMF, 1 drop). The reaction mixture was warmed to room temperature and stirred for 2 h until the starting material was completely consumed (monitored by TLC). The volatiles were removed under reduced pressure, and the residue was co-evaporated with anhydrous toluene to completely remove excess oxalyl chloride, yielding the crude acid chloride intermediate.

Subsequently, the crude acyl chloride was re-dissolved in anhydrous CH_2Cl_2 (1.0 mL). A solution of the corresponding amine **9h** (0.10 mmol, 1.0 equiv.) and triethylamine (Et_3N, 42 µL, 0.30 mmol, 3.0 equiv) in CH_2Cl_2 (1.0 mL) was added dropwise to the mixture at 0 °C. The resulting mixture was stirred at room temperature for 12 h. Upon completion, the reaction was quenched with water (10 mL) and extracted with CH_2Cl_2 (10 mL × 3). The combined organic layers were washed with 1 M HCl, saturated $NaHCO_3$ solution, and brine, dried over anhydrous Na_2SO_4, filtered, and concentrated under vacuum. The residue was purified by preparative thin-layer chromatography (Hexane/EtOAc = 4:1) to afford product **15** (46.2 mg, 0.089 mmol, 89%) as a colorless oily liquid. The product was obtained as an inseparable mixture of isomers. The diastereomeric ratio was determined by ^{19}F NMR analysis of the inseparable mixture of isomers (d.r. = 1.4:1).

^{1}H NMR (400 MHz, Chloroform-*d*) δ 7.80 – 7.64 (m, 3H), 7.51 – 7.40 (m, 2H), 7.34 – 7.27 (m, 1H), 7.26 – 7.21 (m, 2H), 7.20 – 7.12 (m, 2H), 7.06 – 7.00 (m, 1H), 3.92 (s, 3H), 3.91 – 3.80 (m, 5H), 2.67 – 2.57 (m, 1H), 2.28 – 2.16 (m, 1H), 1.74 – 1.62 (m, 5H), 1.60 – 1.51 (m, 3H), 1.29 – 1.17 (m, 3H), 1.13 – 1.02 (m, 1H). **^{13}C{^{1}H} NMR (100 MHz, Chloroform-*d*)** δ 172.6, 158.0, 154.1 (dd, J_{CF} = 286.5, 286.2 Hz), 138.1, 136.1 (d, J_{CF} = 3.3 Hz), 134.4, 134.0, 129.4, 129.1(2), 129.0(6) (d, J_{CF} = 2.6 Hz), 128.0, 126.5, 126.3, 124.2, 119.6, 119.4, 118.8, 110.4, 105.8, 90.8 (dd, J_{CF} = 22.8, 12.6 Hz), 64.7 (d, J_{CF} = 10.3 Hz), 55.4, 48.2, 42.2, 34.5 (d, J_{CF} = 5.0 Hz), 28.6, 26.1, 24.4, 23.8, 18.7. **^{19}F NMR (377 MHz, Chloroform-*d*)** δ –89.89 (d, J_{FF} = 18.7 Hz), –90.00 (d, J_{FF} = 18.7 Hz), –90.61 (d, J_{FF} = 15.0 Hz), –90.72 (d, J_{FF} = 15.1 Hz). **HRMS (ESI)** *m/z*: [M-H]$^{+}$ Calcd for $C_{31}H_{32}F_2NO_4$ 520.2305; Found 520.2303.

8.6. Synthesis of compound 19

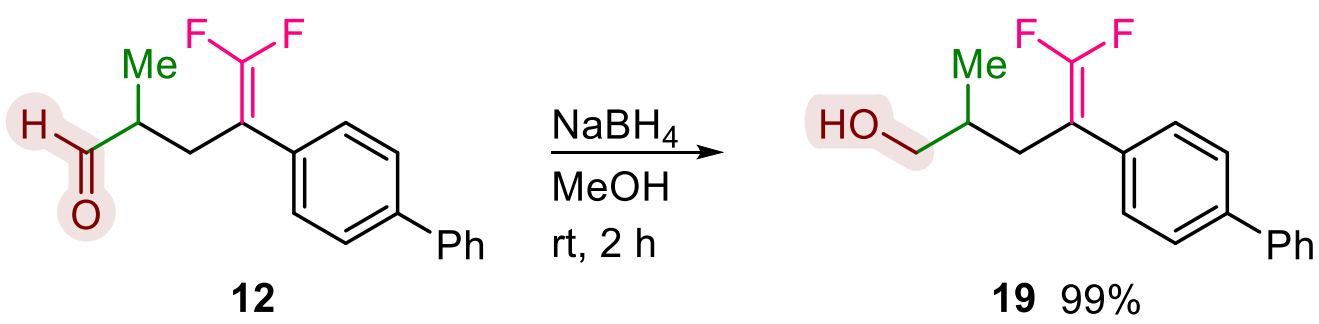

To a 10 mL vial were added **12** (28.6 mg, 0.1 mmol) and anhydrous MeOH (1.0 mL), followed by the addition of $NaBH_4$ (5.7 mg, 0.15 mmol, 1.5 equiv.) in one portion at room temperature. The resulting mixture was stirred at the same temperature until the complete consumption of **12** as indicated by TLC (about 2 h). After quenching with sat. NH_4Cl (5 mL), the solution was extracted with CH_2Cl_2 (10 mL × 3). The combined organic layers were dried over Na_2SO_4 and concentrated under reduced pressure. The residue was purified by preparative thin-layer chromatography (Hexane/EtOAc = 4:1) afforded product **19** (28.5 mg, 0.099 mmol, 99%) as a yellow oily liquid.

^{1}H NMR (400 MHz, Chloroform-*d*) δ 7.65 – 7.56 (m, 4H), 7.50 – 7.39 (m, 4H), 7.39 – 7.33 (m, 1H), 3.50 (d, *J* = 6.0 Hz, 2H), 2.65 – 2.56 (m, 1H), 2.35 – 2.25 (m, 1H), 1.77 – 1.66 (m, 2H), 0.94 (d, *J* = 6.8 Hz, 3H). **^{13}C{^{1}H} NMR (100 MHz, Chloroform-*d*)** δ 154.2 (dd, J_{CF} = 288.6, 288.2 Hz), 140.6, 140.2, 132.6 (d, J_{CF} = 2.0 Hz), 128.9, 128.7 (t, J_{CF} = 3.3 Hz), 127.5, 127.3, 127.1, 90.8 (dd, J_{CF} = 19.2, 15.7 Hz), 67.6, 34.2, 31.1, 16.3. **^{19}F NMR (471 MHz, Chloroform-*d*)** δ –90.55 (s, 2F). **HRMS (ESI)** *m/z*: [M+Na]$^{+}$ Calcd for $C_{18}H_{18}F_2ONa$ 311.1218; Found 311.1216.

8.7. Synthesis of compound 20

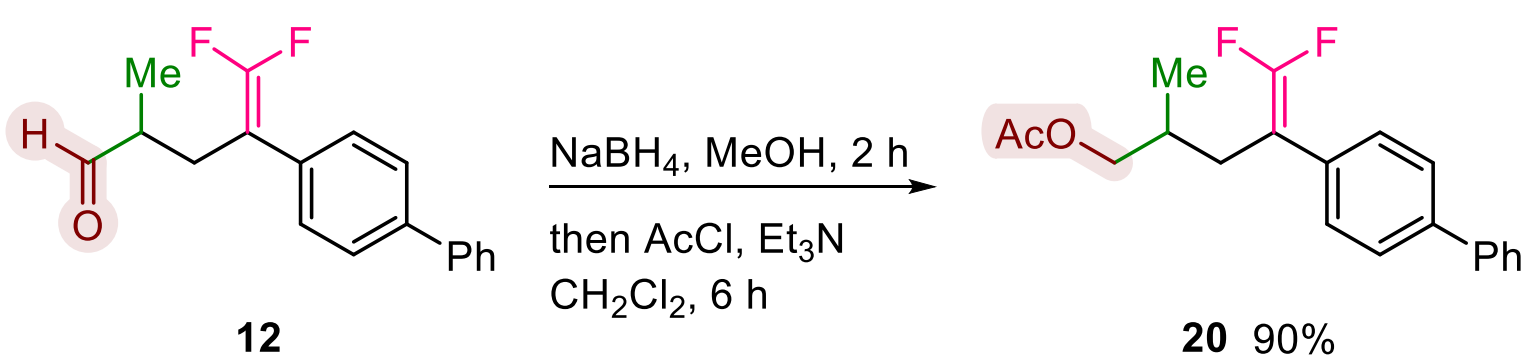

To a 10 mL vial were added **12** (28.6 mg, 0.1 mmol) and anhydrous MeOH (1.0 mL), followed by the addition of $NaBH_4$ (5.7 mg, 0.15 mmol, 1.5 equiv.) in one portion at room temperature. The resulting mixture was stirred at the same temperature until the complete consumption of **12** as indicated by TLC (about 2 h). After quenching with sat. NH_4Cl (5 mL), the solution was extracted with CH_2Cl_2 (10 mL × 3). The combined organic layers were dried over Na_2SO_4 and concentrated under reduced pressure. Then added acetyl chloride (15.8 mg, 0.20 mmol, 2.0 equiv.), triethylamine (20.2 mg, 0.20 mmol, 2.0 equiv.), and DCM (5 mL), and the mixture was stirred for 6 hours at room temperature. The mixture was quenched by sat. aqueous $NaHCO_3$ successively and diluted by CH_2Cl_2. The organic phase was collected, and the aqueous phase was extracted with CH_2Cl_2. The combined organic layers were dried over anhydrous Na_2SO_4, filtered, and concentrated under reduced pressure. The product **20** was isolated by preparative thin-layer chromatography (Hexane/EtOAc = 10:1) as a colorless oil (29.7 mg, 90%).

^{1}H NMR (400 MHz, Chloroform-*d*) δ 7.63 – 7.57 (m, 4H), 7.50 – 7.32 (m, 5H), 3.95 (d, J = 5.9 Hz, 2H), 2.62 – 2.52 (m, 1H), 2.40 – 2.29 (m, 1H), 2.06 (s, 3H), 1.95 – 1.79 (m, 1H), 0.97 (d, J = 6.8 Hz, 3H). **^{13}C{^{1}H} NMR (100 MHz, Chloroform-*d*)** δ 171.3, 154.2 (t, J_{CF} = 289.0 Hz), 140.6, 140.3, 132.4, 128.9, 128.7 (t, J_{CF} = 3.3 Hz), 127.6, 127.3, 127.1, 90.5 (dd, J_{CF} = 18.2, 16.8 Hz), 68.5, 31.3, 31.1 (t, J_{CF} = 2.5 Hz), 21.0, 16.7. **^{19}F NMR (377 MHz, Chloroform-*d*)** δ –90.27 (s, 2F). **HRMS (ESI)** *m/z*: $[M+H]^+$ Calcd for $C_{20}H_{21}F_2O_2$ 331.1504; Found 331.1501.

8.8. Synthesis of compound 21 and 21’

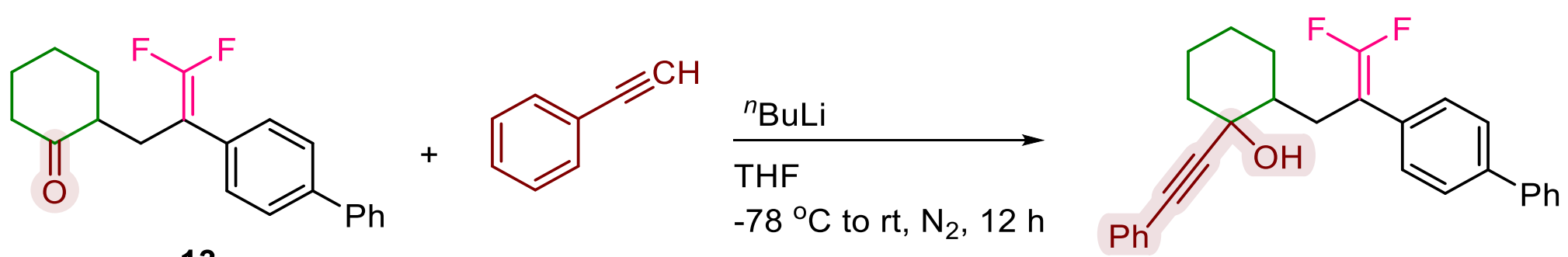

Ethynylbenzene (13.2 µL, 0.12 mmol, 1.2 equiv) was dissolved in anhydrous THF (2 mL) at -78 °C. Then, ^{n}BuLi (2.4 M in hexanes, 84 µL, 0.2 mmol, 2 equiv.) was added dropwise and the reaction mixture was stirred for 1 h before adding **13** (32.6 mg, 0.1 mmol, 1.0 equiv.). The reaction mixture was allowed to stir at room temperature overnight. The reaction was quenched with sat. NH_4Cl (5 mL) and extracted with EtOAc (10 mL ×3). The combined organic phases were dried over Na_2SO_4, filtered and concentrated under reduced pressure to afford the crude compound which was purified by preparative thin-layer chromatography (Hexane/EtOAc = 10:1, Rf_1 = 0.4, Rf_2 = 0.3) to afford products **21** (27.4 mg, 0.064 mmol, 64%), **21’** (9.86 mg, 0.023 mmol, 23%) as the colorless oil.

^{1}H NMR (400 MHz, Chloroform-*d*) (21) δ 7.63 – 7.53 (m, 4H), 7.53 – 7.41 (m, 6H), 7.40 – 7.32 (m, 4H), 3.19 – 3.12 (m, 1H), 2.60 – 2.50 (m, 1H), 2.12 – 2.04 (m, 1H), 1.85 – 1.54 (m, 7H), 1.46 – 1.33 (m, 1H), 1.25 – 1.12 (m, 1H). **^{13}C{^{1}H} NMR (100 MHz, Chloroform-*d*) (21)** δ 154.4 (dd, J_{CF} = 288.4, 287.0 Hz), 140.6, 140.0, 132.5, 131.7, 128.9, 128.7 (t, J_{CF} = 3.5 Hz), 128.5, 127.5, 127.2, 127.1, 122.8, 93.5, 90.8 (dd, J_{CF} = 19.5, 14.8 Hz), 83.9, 70.2, 43.9, 39.9, 28.7, 25.9, 24.9, 21.2. **^{19}F NMR (377 MHz, Chloroform-*d*) (21)** δ –89.72 (d, J_{FF} = 41.7 Hz), –89.84 (d, J_{FF} = 41.7 Hz).

^{1}H NMR (400 MHz, Chloroform-*d*) (21') δ 7.65 – 7.57 (m, 4H), 7.52 – 7.41 (m, 6H), 7.40 – 7.32 (m, 4H), 3.16 – 3.06 (m, 1H), 2.58 – 2.47 (m, 1H), 2.13 – 2.08 (m, 1H), 1.82 – 1.48 (m, 7H), 1.37 – 1.23 (m, 1H), 1.19 – 1.03 (m, 1H). **^{13}C{^{1}H}NMR (100 MHz, Chloroform-*d*) (21')** δ 154.4 (dd, J_{CF} = 291.5, 286.4 Hz), 140.7, 140.0, 132.6 (t, J_{CF} = 3.7 Hz), 131.9, 128.9, 128.7 (t, J_{CF} = 3.6 Hz), 128.6, 128.5, 127.5, 127.3, 127.1, 122.8, 89.9, 87.1, 73.1, 45.9 (d, J_{CF} = 2.9 Hz), 41.5, 28.9, 28.5, 25.3, 24.3. **^{19}F NMR (377 MHz, Chloroform-*d*) (21')** δ –89.62 (d, J_{FF} = 41.7 Hz), –89.92 (d, J_{FF} = 41.6 Hz). **HRMS (ESI)** *m/z*: $[M+Na]^+$ Calcd for $C_{29}H_{26}F_2ONa$ 451.1844; Found 451.1844.

8.9. Synthesis of compound 22

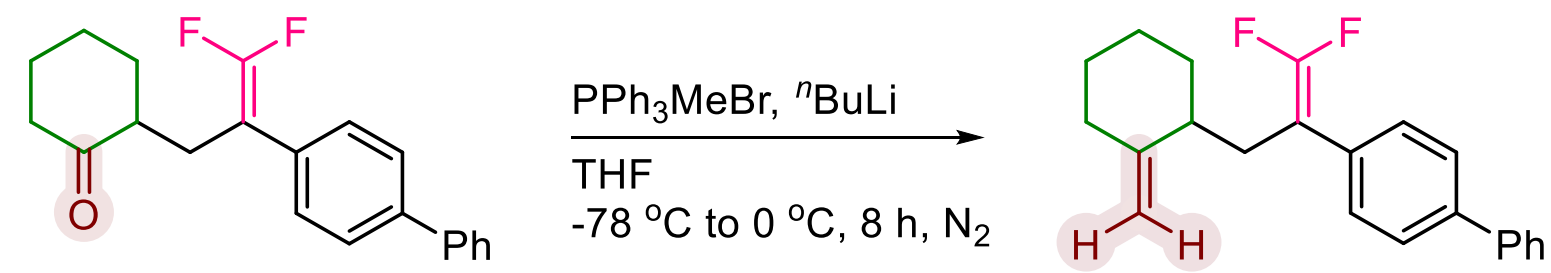

13 **22** 23%

In a 10 mL reaction tube were added methyltriphenylphosphonium bromide (53.6 mg, 0.15 mmol, 1.5 equiv.), THF (1.6 mL) at -78 °C. ^{n}BuLi (2.5 M in hexanes, 60 μL, 1.15 mmol, 1.5 equiv.) was added dropwise, and the resulting mixture was stirred for 15 min. A solution of **13** (32.6 mg, 0.1 mmol, 1.0 equiv.) in THF (0.4 mL) was then added dropwise to the reaction system. The mixture was warmed to 0 °C and stirred for 8 h. After completion, the reaction was quenched with water and extracted with CH_2Cl_2 (5mL × 3). The combined organic layers were dried over Na_2SO_4, filtered, and concentrated under reduced pressure. The residue was purified by preparative thin-layer chromatography by using PE as eluent to afford product **22** (7.5 mg, 0.023 mmol, 23%) as a colorless oil.

^{1}H NMR (500 MHz, Chloroform-*d*) δ 7.64 – 7.57 (m, 4H), 7.49 – 7.32 (m, 5H), 4.70 (s, 1H), 4.58 (s, 1H), 2.77 – 2.70 (m, 1H), 2.52 – 2.44 (m, 1H), 2.30 – 2.22 (m, 1H), 2.09 – 2.04 (m, 1H), 2.04 – 1.95 (m, 1H), 1.78 – 1.64 (m, 1H), 1.62 – 1.56 (m, 2H), 1.54 – 1.45 (m, 1H), 1.43 – 1.34 (m, 1H), 1.31 – 1.21 (m, 1H). **^{13}C{^{1}H} NMR (100 MHz, Chloroform-*d*)** δ 154.2 (dd, J_{CF} = 290.5, 286.4 Hz), 152.2, 140.7, 140.1, 132.7 (t, J_{CF} = 3.7 Hz), 128.9, 128.8 (t, J_{CF} = 3.3 Hz), 127.5, 127.2, 127.1, 106.2, 91.0 (dd, J_{CF} = 21.9, 13.0 Hz), 40.5, 34.8, 32.8, 30.5 (d, J_{CF} = 1.5 Hz), 28.7, 24.2. **^{19}F NMR (471 MHz,**

Chloroform-*d*) δ –90.48 (d, J_{FF} = 43.3 Hz), –91.01 (d, J_{FF} = 42.9 Hz). **HRMS (ESI)** *m/z*: $[M]^+$ Calcd for $C_{22}H_{22}F_2$ 324.1684; Found 324.1683.

8.10. Synthesis of compound 23, 23' and 24

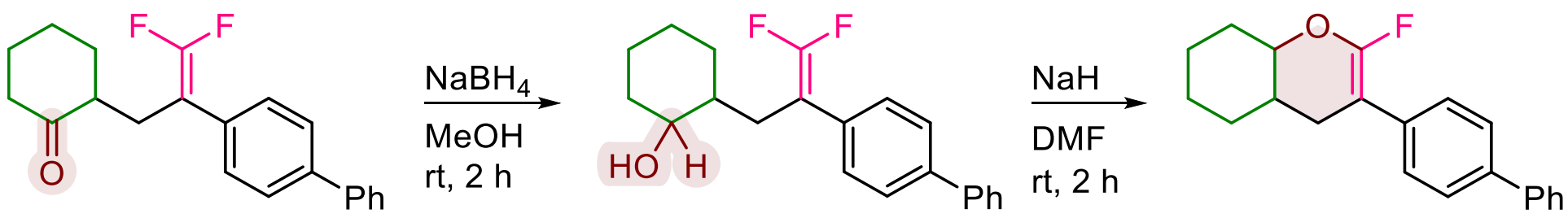

13 **23, 23'** 99% (d.r. = 1.5:1) **24** 60% (d.r. = 8.0:1)

To a 10 mL vial were added **13** (32.6 mg, 0.1 mmol) and anhydrous MeOH (1.0 mL), followed by the addition of $NaBH_4$ (5.7 mg, 0.15 mmol, 1.5 equiv.) in one portion at room temperature. The resulting mixture was stirred at the same temperature until the complete consumption of **13** as indicated by TLC (about 2 h). After quenching with sat. NH_4Cl (5 mL), the solution was extracted with CH_2Cl_2 (10 mL × 3). The combined organic layers were dried over Na_2SO_4 and concentrated under reduced pressure. The residue was purified by preparative thin-layer chromatography (Hexane/EtOAc = 10:1) afforded product **23** (19.4 mg, 0.059 mmol, 59%) and **23'** (13.0 mg, 0.040 mmol, 40%) as a colorless oil.

^{1}H NMR (500 MHz, Chloroform-*d*) (23) δ 7.64 – 7.56 (m, 4H), 7.47 – 7.39 (m, 4H), 7.38 – 7.33 (m, 1H), 3.88 – 3.82 (m, 1H), 2.60 – 2.39 (m, 2H), 1.79 – 1.73 (m, 1H), 1.70 – 1.62 (m, 1H), 1.61 – 1.54 (m, 1H), 1.49 – 1.37 (m, 5H), 1.31 – 1.23 (m, 1H), 1.23 – 1.15 (m, 1H). **^{13}C{^{1}H} NMR (125 MHz, Chloroform-*d*) (23)** δ 154.2 (dd, J_{CF} = 290.9, 286.8 Hz), 140.6, 140.2, 132.9 (t, J_{CF} = 3.6 Hz), 129.0, 128.7 (t, J_{CF} = 3.3 Hz), 127.6, 127.3, 127.1, 90.8 (dd, J_{CF} = 21.7, 12.9 Hz), 68.9, 39.6, 33.1, 29.9, 26.3, 25.0, 20.4. **^{19}F NMR (471 MHz, Chloroform-*d*) (23)** δ –90.36 (d, J_{FF} = 42.9 Hz), –90.57 (d, J_{FF} = 43.0 Hz).

^{1}H NMR (500 MHz, Chloroform-*d*) (23') δ 7.68 – 7.53 (m, 4H), 7.50 – 7.31 (m, 5H), 3.32 – 3.20 (m, 1H), 3.10 – 2.97 (m, 1H), 2.31 – 2.18 (m, 1H), 2.00 – 1.88 (m, 1H), 1.85 – 1.76 (m, 1H), 1.76 – 1.66 (m, 1H), 1.66 – 1.57 (m, 1H), 1.45 – 1.33 (m, 1H), 1.32 – 1.15 (m, 3H), 1.12 – 1.01 (m, 1H), 1.01 – 0.89 (m, 1H). **^{13}C{^{1}H} NMR (125 MHz, Chloroform-*d*) (23')** δ 154.3 (dd, J_{CF} = 286.5, 285.8 Hz), 140.7, 140.0, 132.8, 128.9, 128.7, 127.5, 127.3, 127.1, 90.7 (dd, J_{CF} = 21.7, 13.0 Hz), 74.8, 43.5, 35.9, 30.4, 29.9, 25.4, 25.0. **^{19}F NMR (471 MHz, Chloroform-*d*) (23')** δ –90.34 (d, J_{FF} = 42.9 Hz), –90.54 (d, J_{FF} = 43.0 Hz). **HRMS (ESI)** *m/z*: $[M+Na]^+$ Calcd for $C_{21}H_{22}F_2ONa$ 351.1531; Found 351.1531.

A flask charged with NaH (60% dispersion in mineral oil, 8.0 mg, 0.2 mmol, 2.0 equiv.) in dry DMF (2.0 mL) was cooled to 0 °C, before *gem*-difluoroalkene **23** and **23'** (32.4 mg, 0.099 mmol, 1.0 equiv.) were added. The reaction was allowed to warm up and stirred at room temperature for 2 h. After

completion, the reaction was quenched with a minimal amount of water. The resultant crude product was purified by preparative thin-layer chromatography (Hexane/EtOAc = 20:1) to afford product **24** (18.3 mg, 0.059 mmol, 60%) as a white solid. The product was obtained as an inseparable mixture of isomers. The diastereomeric ratio was determined by ^{19}F NMR analysis of the inseparable mixture of isomers (d.r. = 8.0:1). Melting point 163.6 °C – 168.5 °C.

^{1}H NMR (400 MHz, Chloroform-*d*) δ 7.65 – 7.54 (m, 4H), 7.51 – 7.41 (m, 4H), 7.38 – 7.30 (m, 1H), 3.89 – 3.76 (m, 1H), 2.52 – 2.43 (m, 1H), 2.27 – 2.14 (m, 1H), 2.07 – 1.97 (m, 1H), 1.94 – 1.85 (m, 1H), 1.79 – 1.69 (m, 2H), 1.59 – 1.48 (m, 2H), 1.39 – 1.29 (m, 2H), 1.16 – 1.02 (m, 1H). **^{13}C{^{1}H} NMR (100 MHz, Chloroform-*d*)** δ 155.2 (d, J_{CF} = 259.1 Hz), 141.0, 138.2, 135.7 (d, J_{CF} = 4.5 Hz), 128.9, 127.1 (d, J_{CF} = 6.8 Hz), 127.0(4), 126.9(7) (d, J_{CF} = 7.7 Hz), 84.5 (d, J_{CF} = 13.7 Hz), 81.8, 37.7, 31.5, 31.3, 31.2(7), 31.2(3) (d, J_{CF} = 2.2 Hz), 25.3, 24.3. **^{19}F NMR (471 MHz, Chloroform-*d*)** δ –87.91. **HRMS (ESI)** *m/z*: $[M+Na]^+$ Calcd for $C_{21}H_{21}FONa$ 308.1571; Found 308.1570.

9. ^{1}H, ^{13}C, and ^{19}F NMR Spectra

6-(2-([1,1'-Biphenyl]-4-yl)-3,3-difluoroallyl)-1,4-dioxaspiro[4.5]decane (Scheme 2, product 9a)

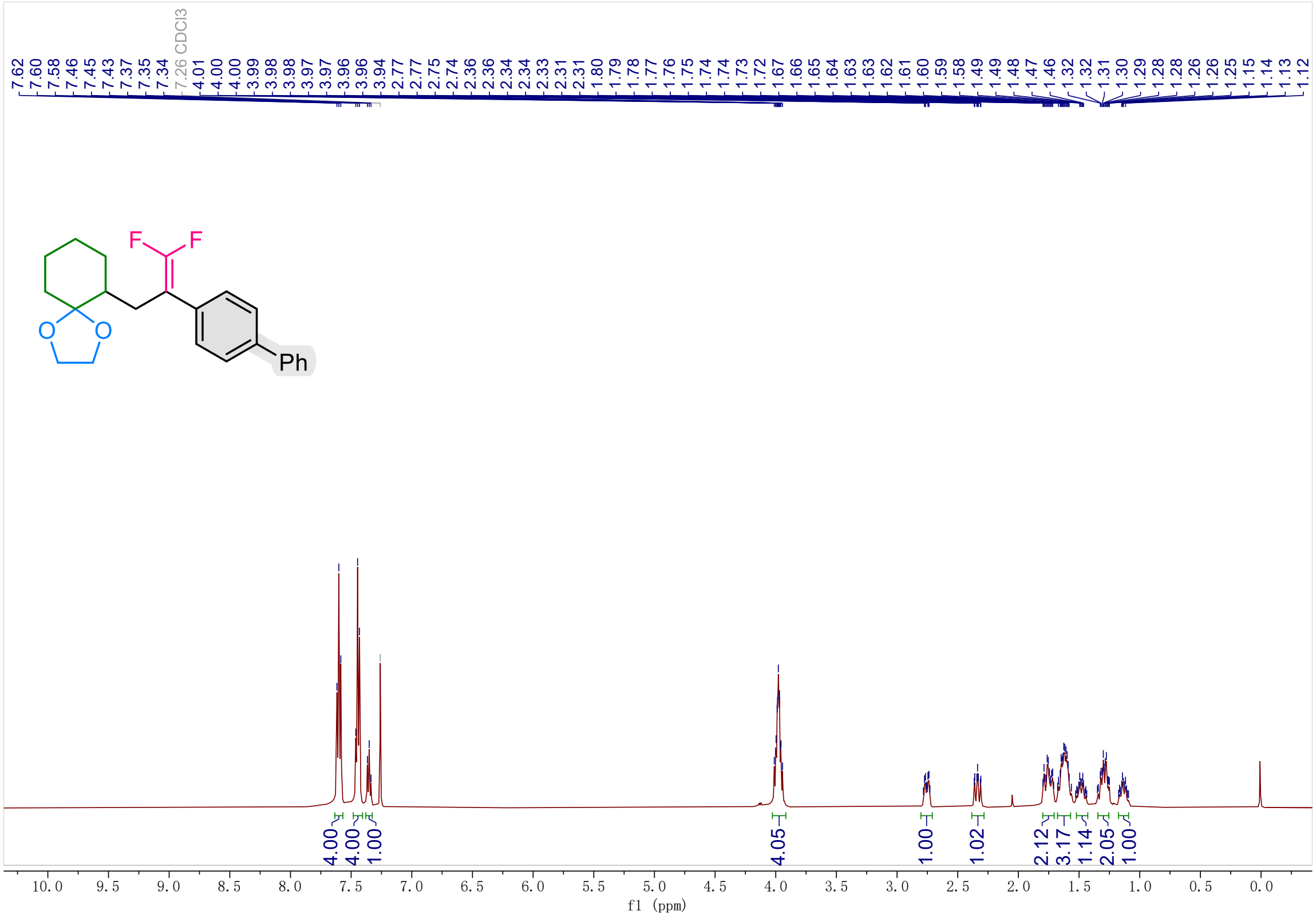

^{1}H NMR spectrum (500 MHz, Chloroform-*d*) of **9a**

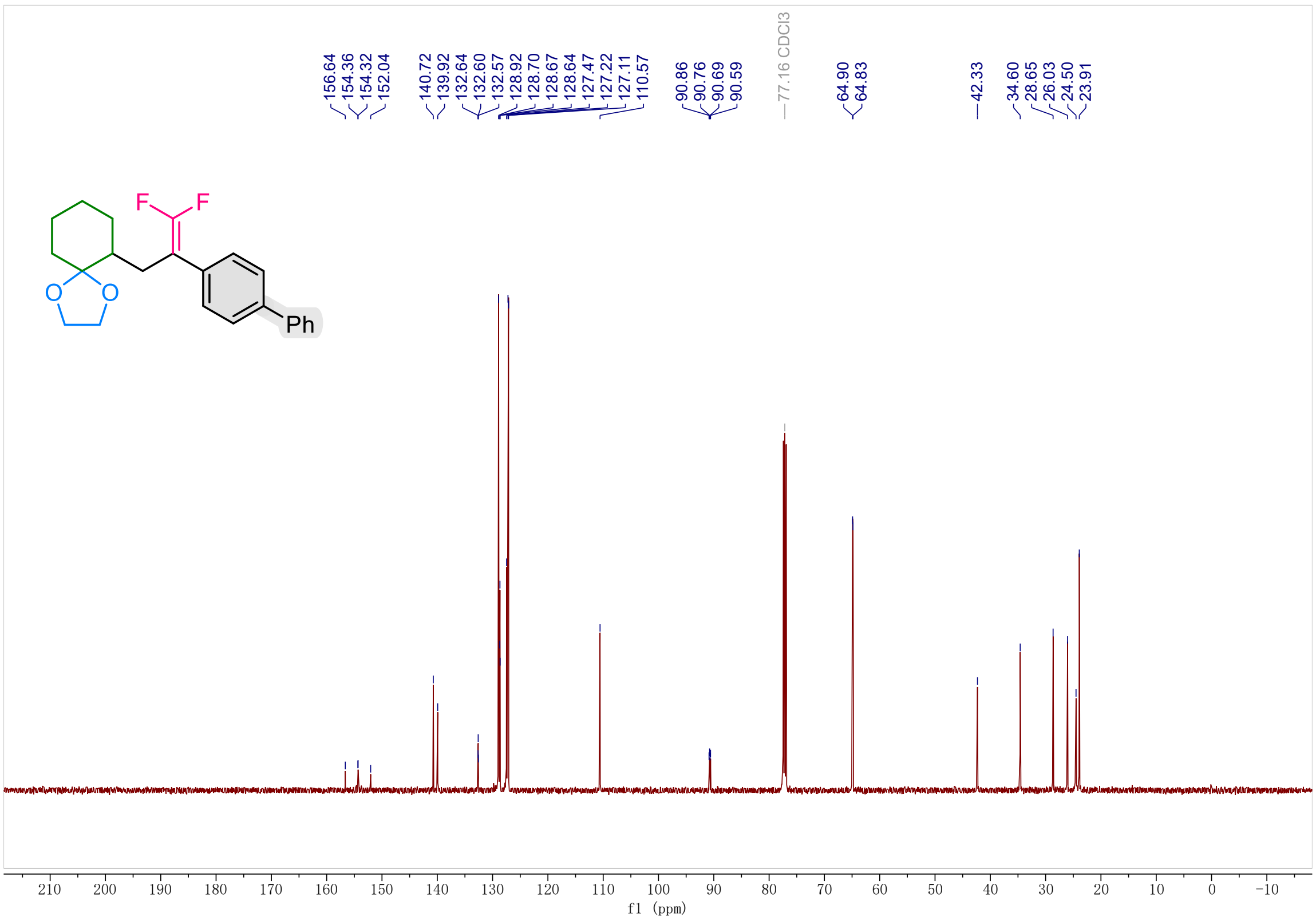

^{13}C NMR spectrum (125 MHz, Chloroform-*d*) of **9a**

6-(2-([1,1'-Biphenyl]-4-yl)-3,3-difluoroallyl)-1,4-dioxaspiro[4.5]decane (Scheme 2, product 9a)

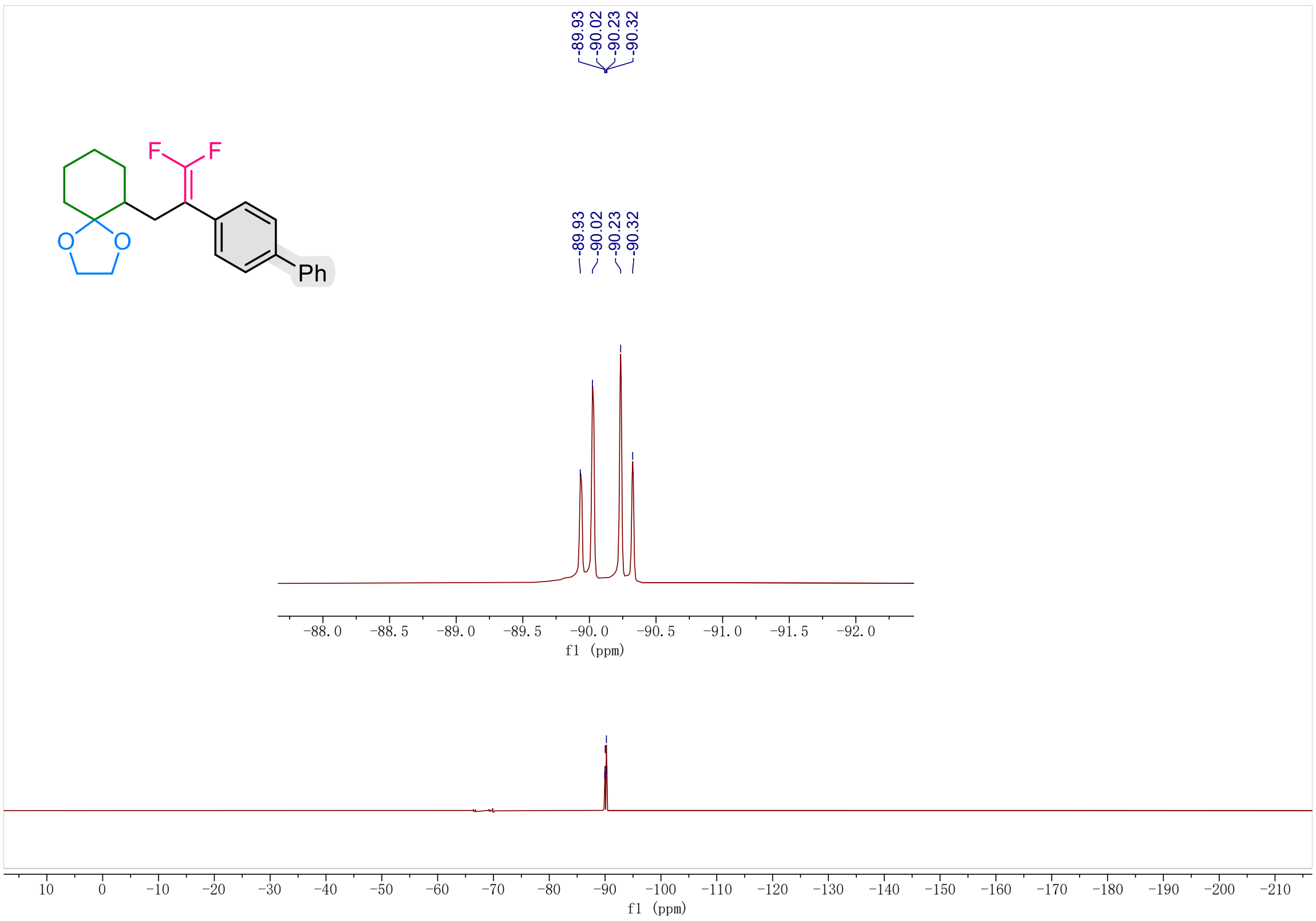

^{19}F NMR spectrum (471 MHz, Chloroform-*d*) of **9a**

6-(3,3-Difluoro-2-(4-(methylthio)phenyl)allyl)-1,4-dioxaspiro[4.5]decane (Scheme 2, product 9b)

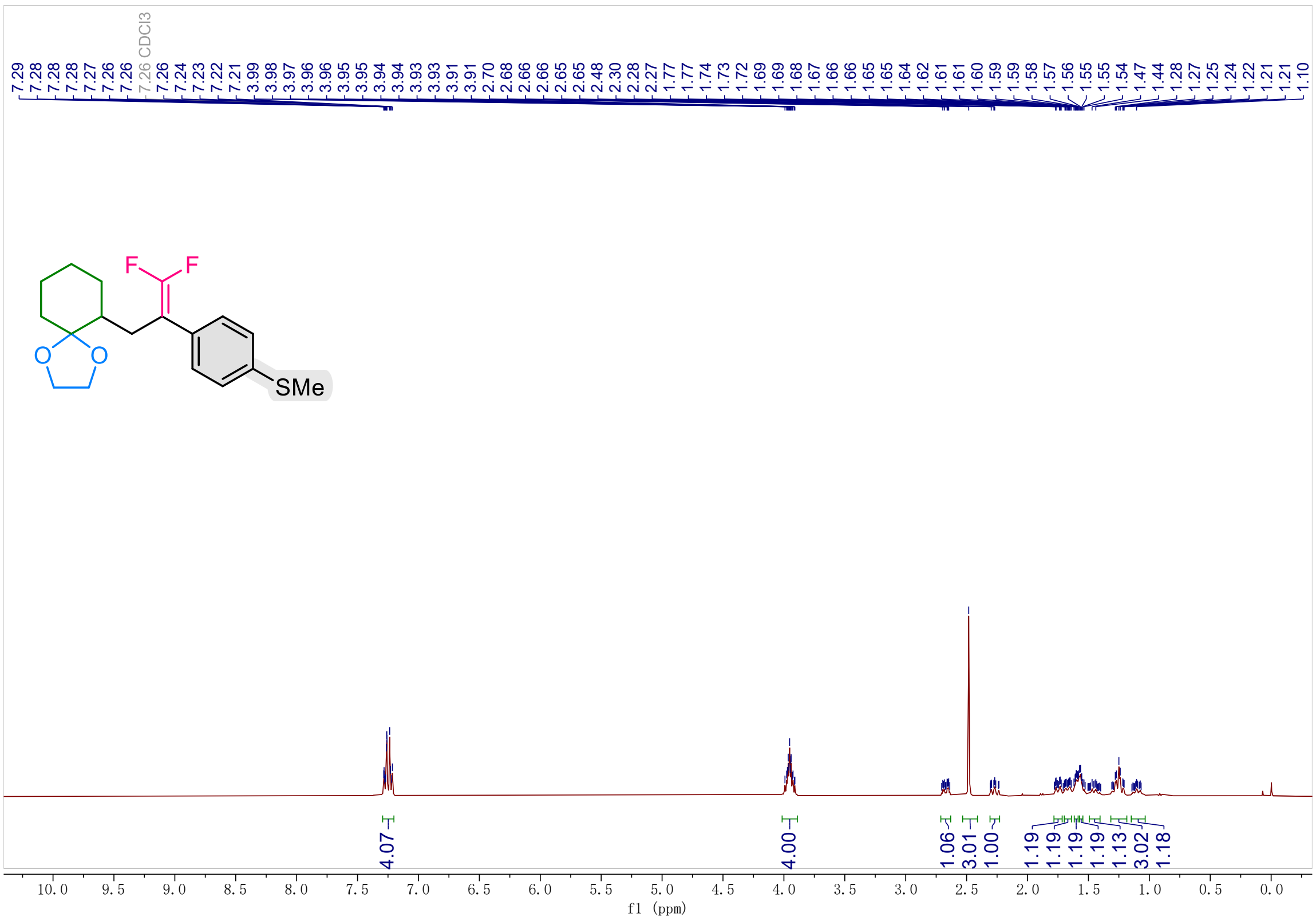

^{1}H NMR spectrum (400 MHz, Chloroform-*d*) of **9b**

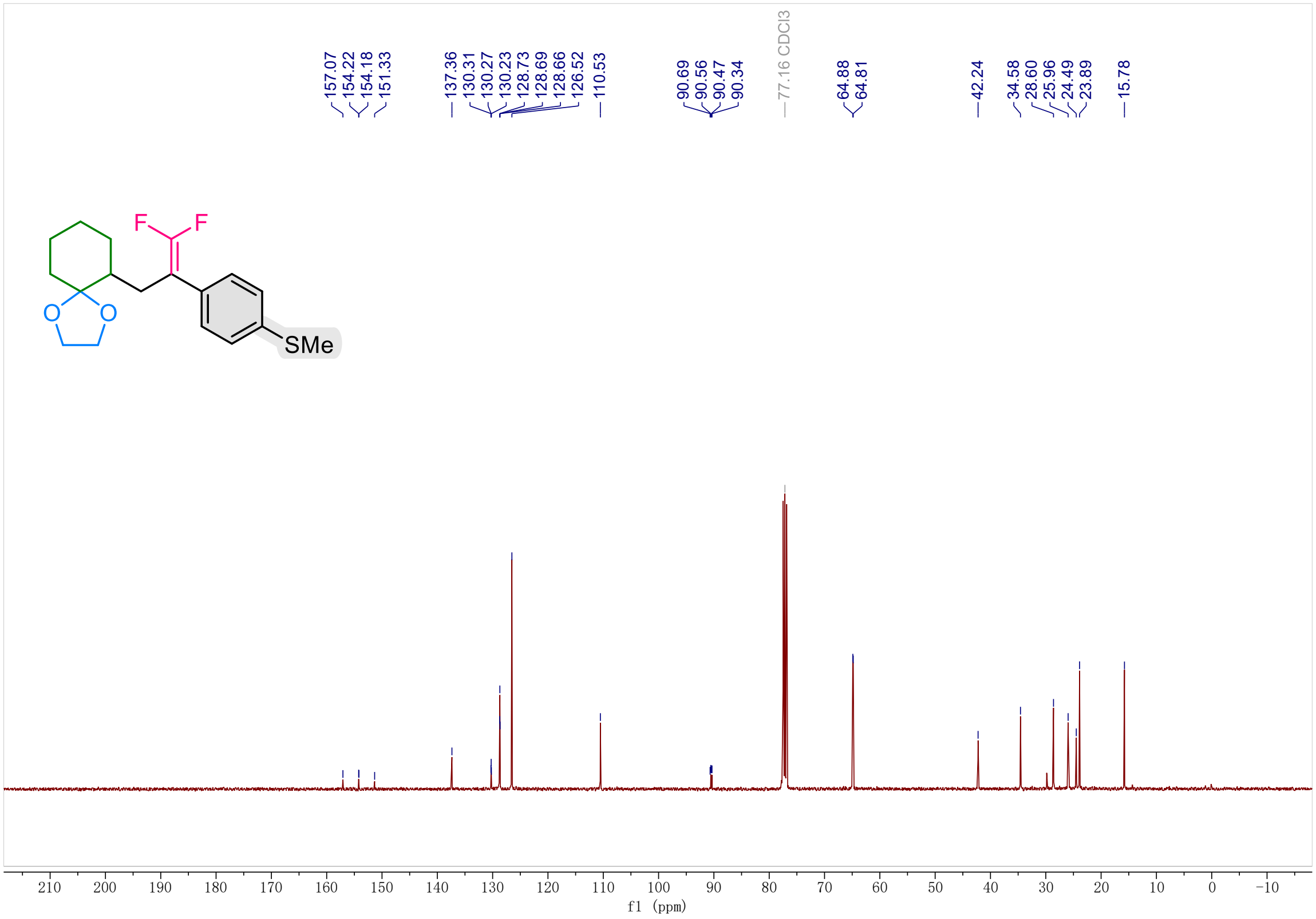

^{13}C NMR spectrum (100 MHz, Chloroform-*d*) of **9b**

6-(3,3-Difluoro-2-(4-(methylthio)phenyl)allyl)-1,4-dioxaspiro[4.5]decane (Scheme 2, product 9b)

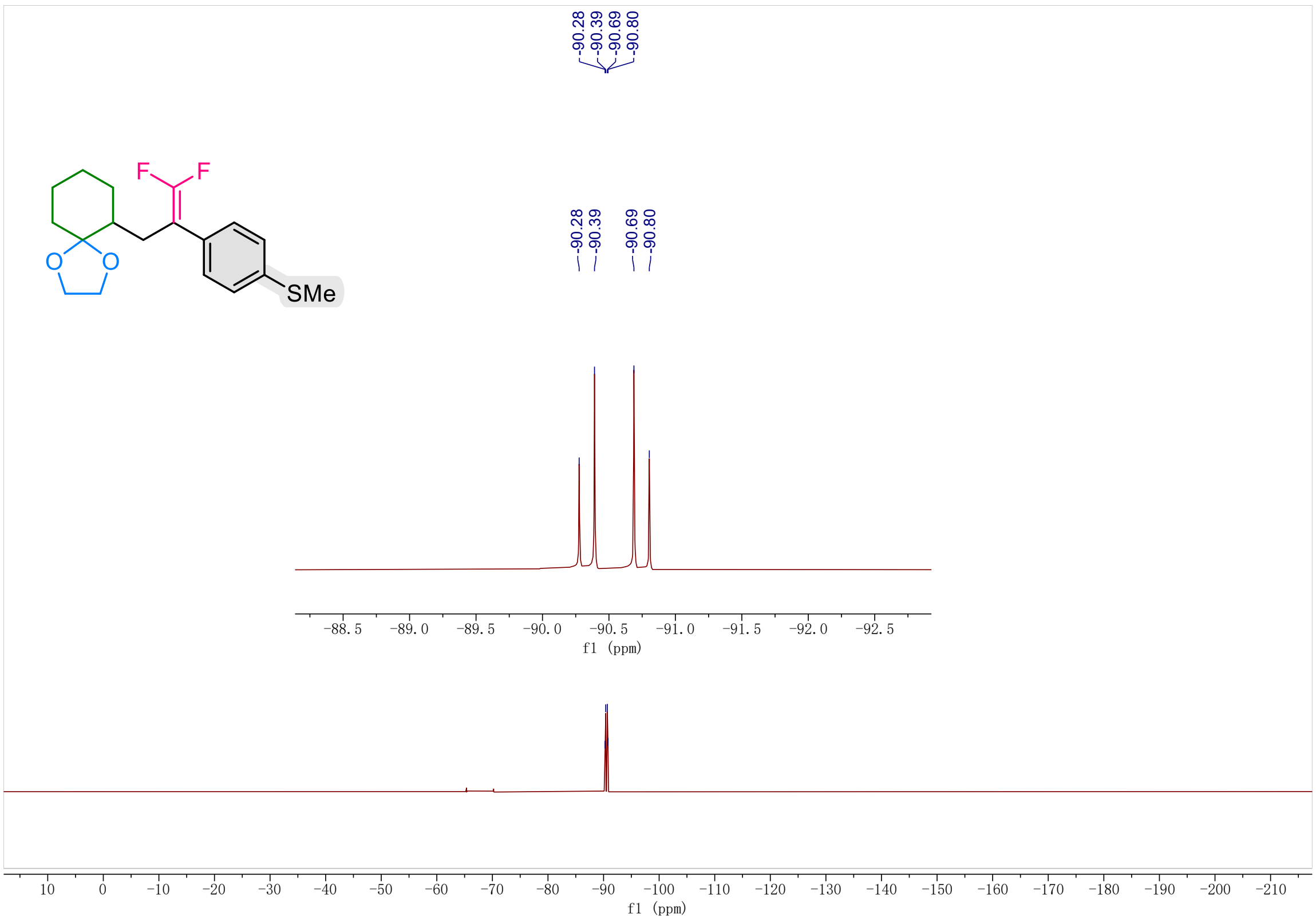

^{19}F NMR spectrum (377 MHz, Chloroform-*d*) of **9b**

6-(2-(4-Chlorophenyl)-3,3-difluoroallyl)-1,4-dioxaspiro[4.5]decane (Scheme 2, product 9c)

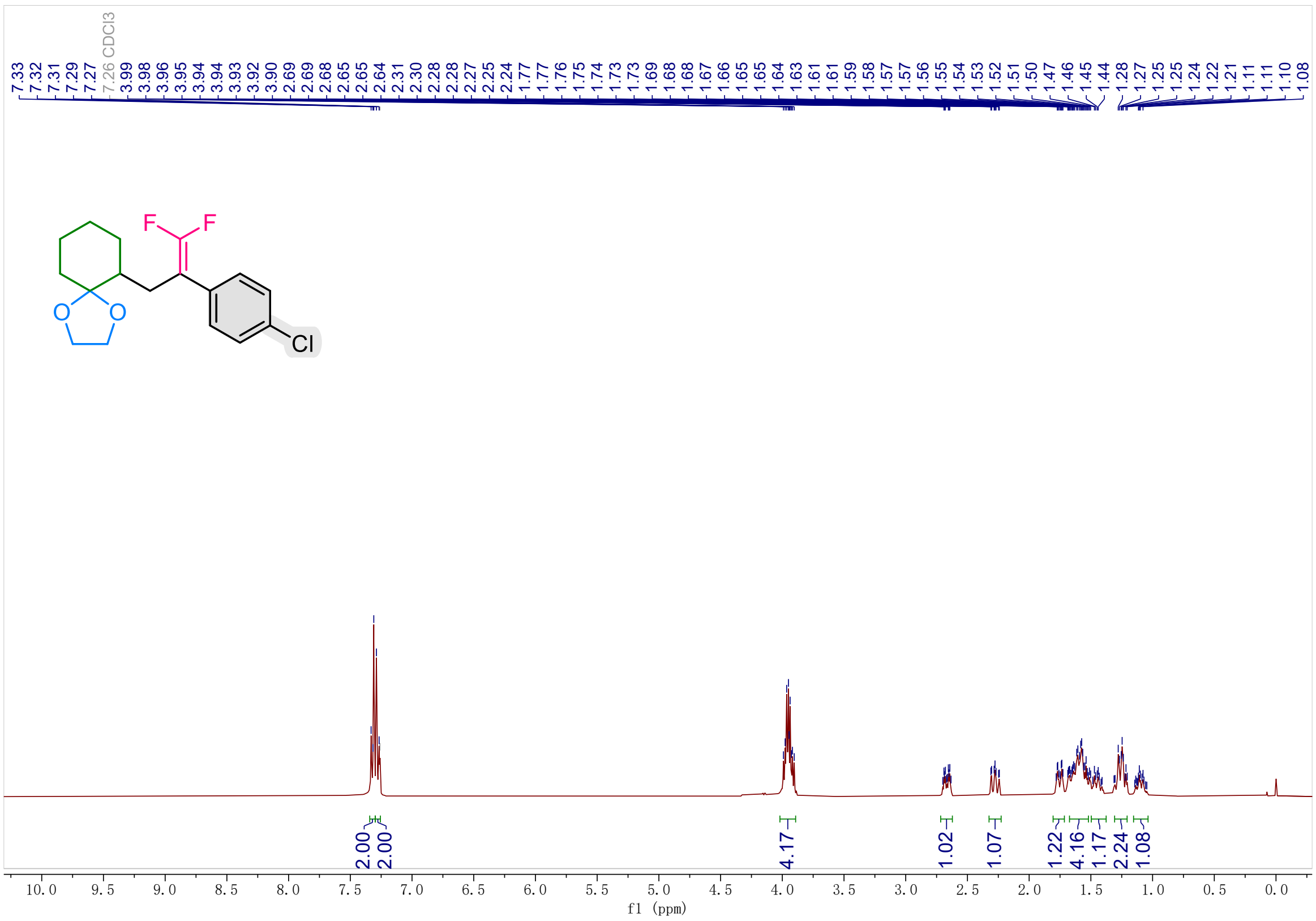

^{1}H NMR spectrum (400 MHz, Chloroform-*d*) of **9c**

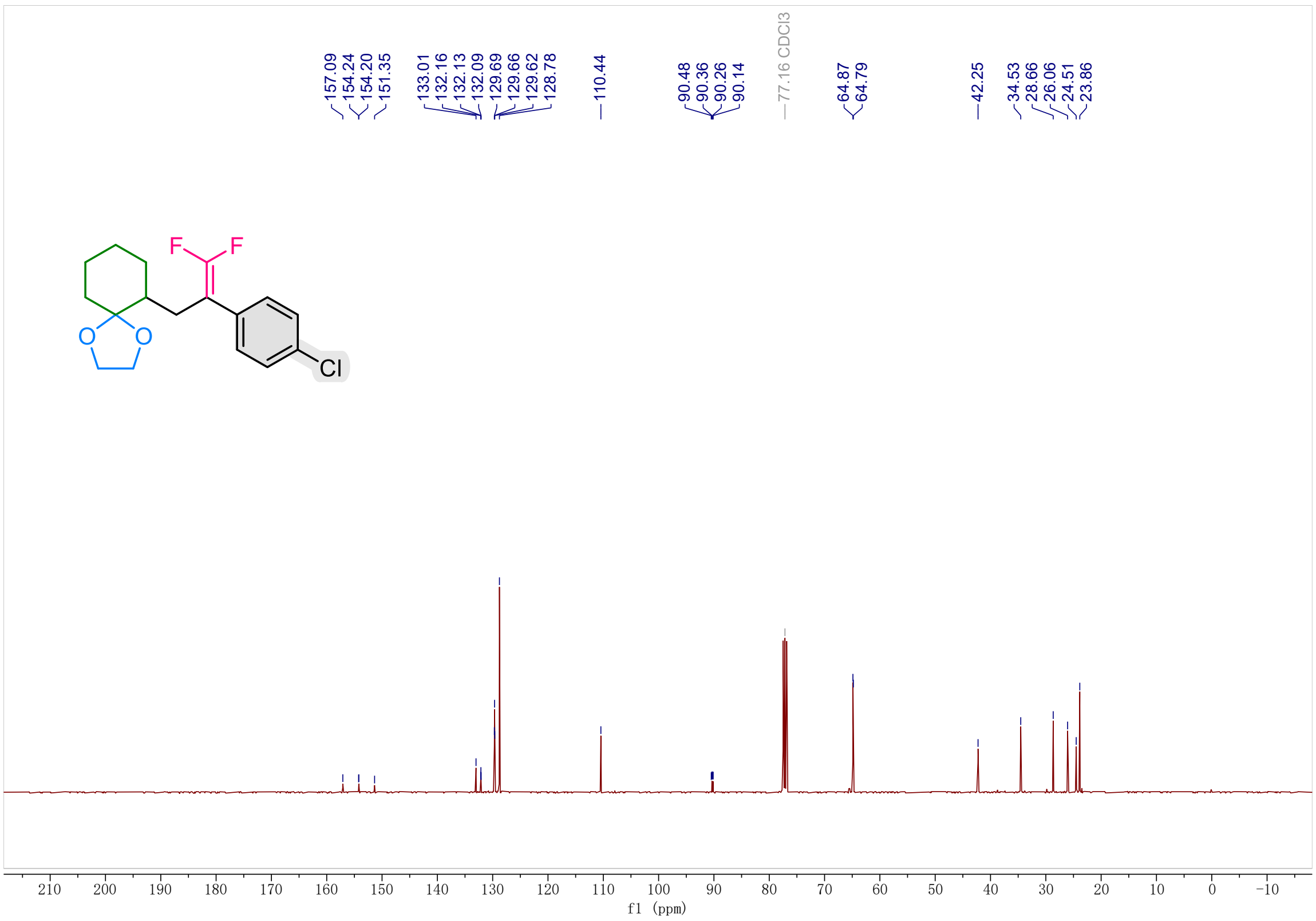

^{13}C NMR spectrum (100 MHz, Chloroform-*d*) of **9c**

6-(2-(4-Chlorophenyl)-3,3-difluoroallyl)-1,4-dioxaspiro[4.5]decane (Scheme 2, product 9c)

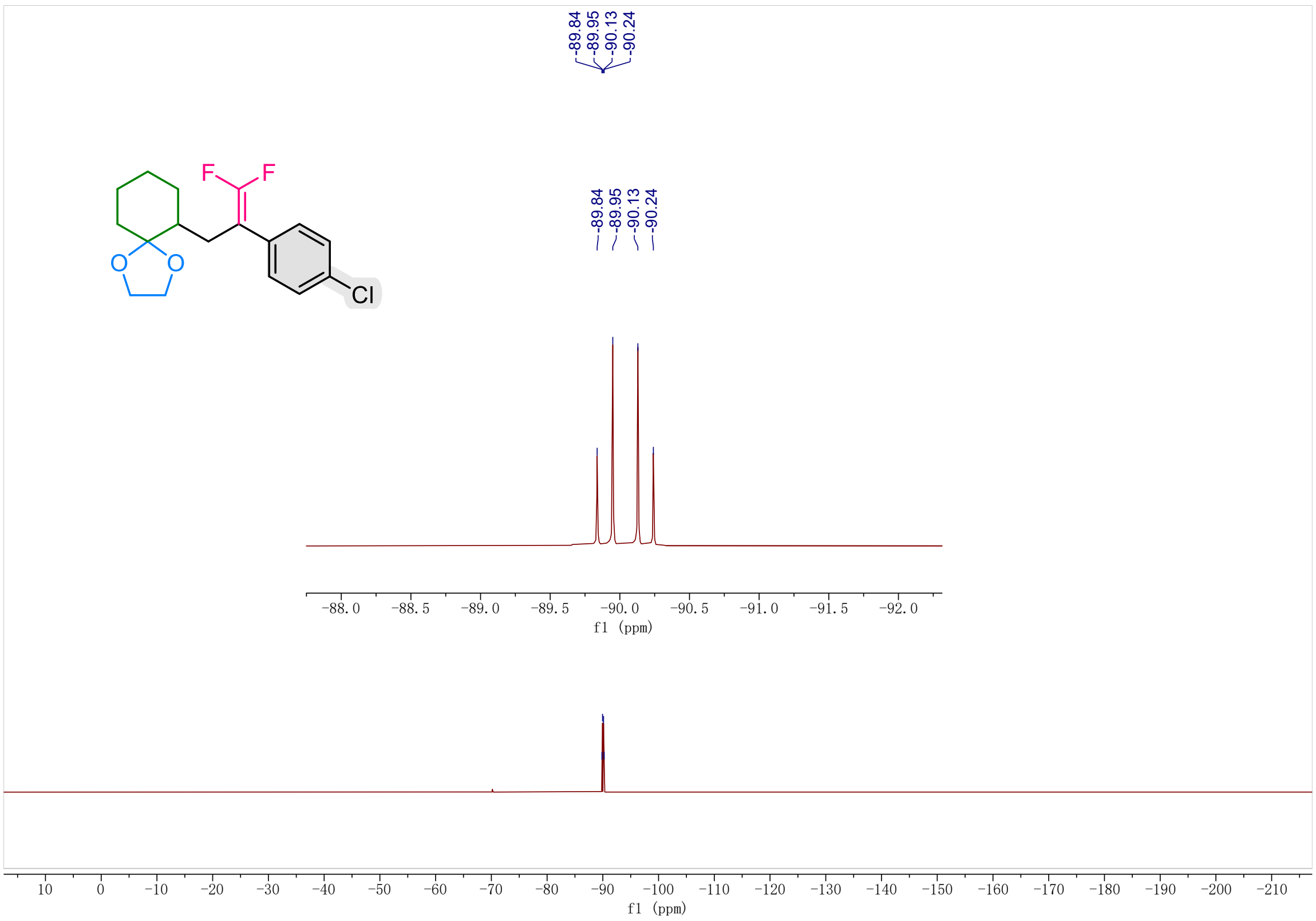

^{19}F NMR spectrum (377 MHz, Chloroform-*d*) of **9c**

6-(2-(4-(Benzyloxy)phenyl)-3,3-difluoroallyl)-1,4-dioxaspiro[4.5]decane (Scheme 2, product 9d)

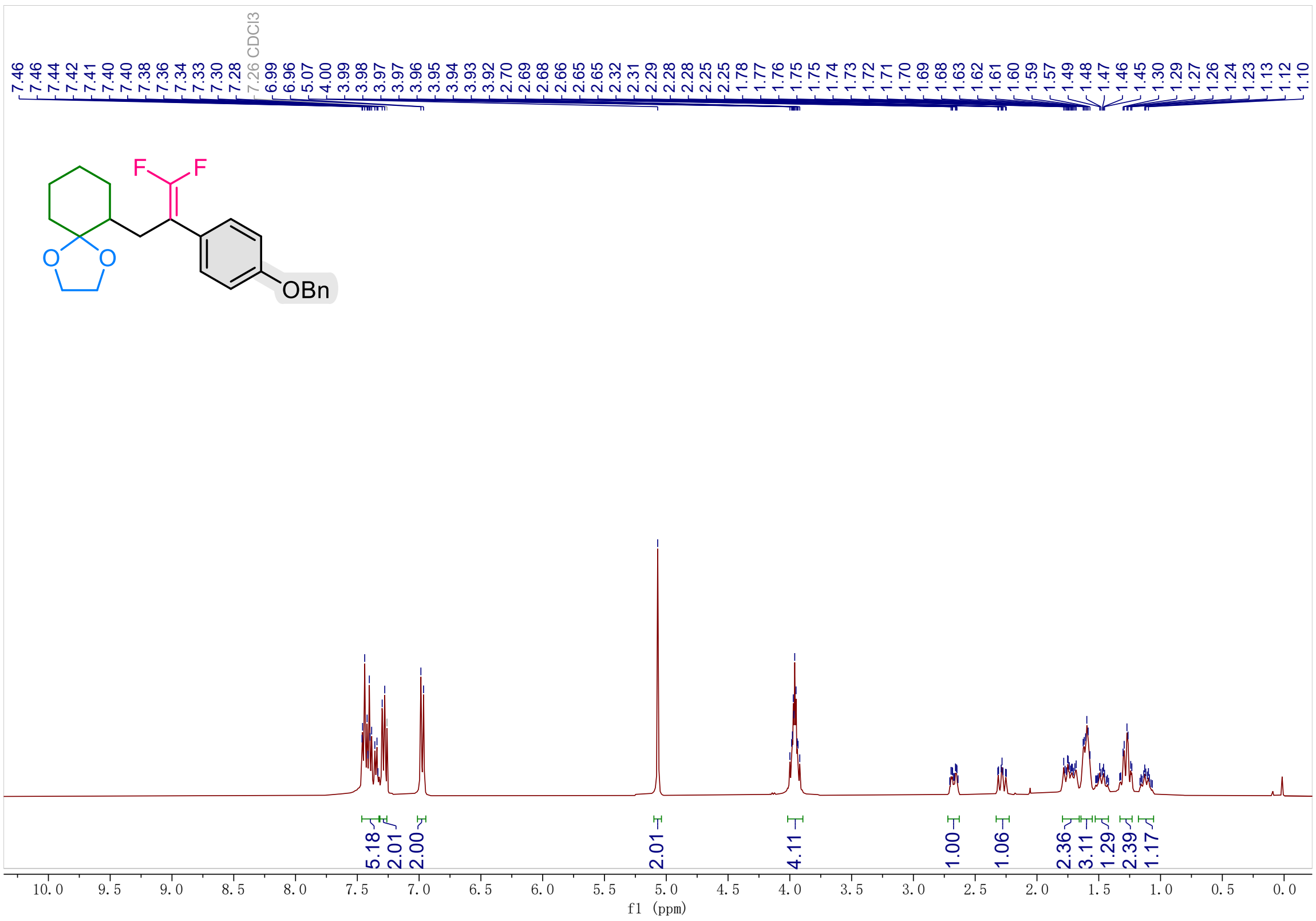

^{1}H NMR spectrum (400 MHz, Chloroform-*d*) of **9d**

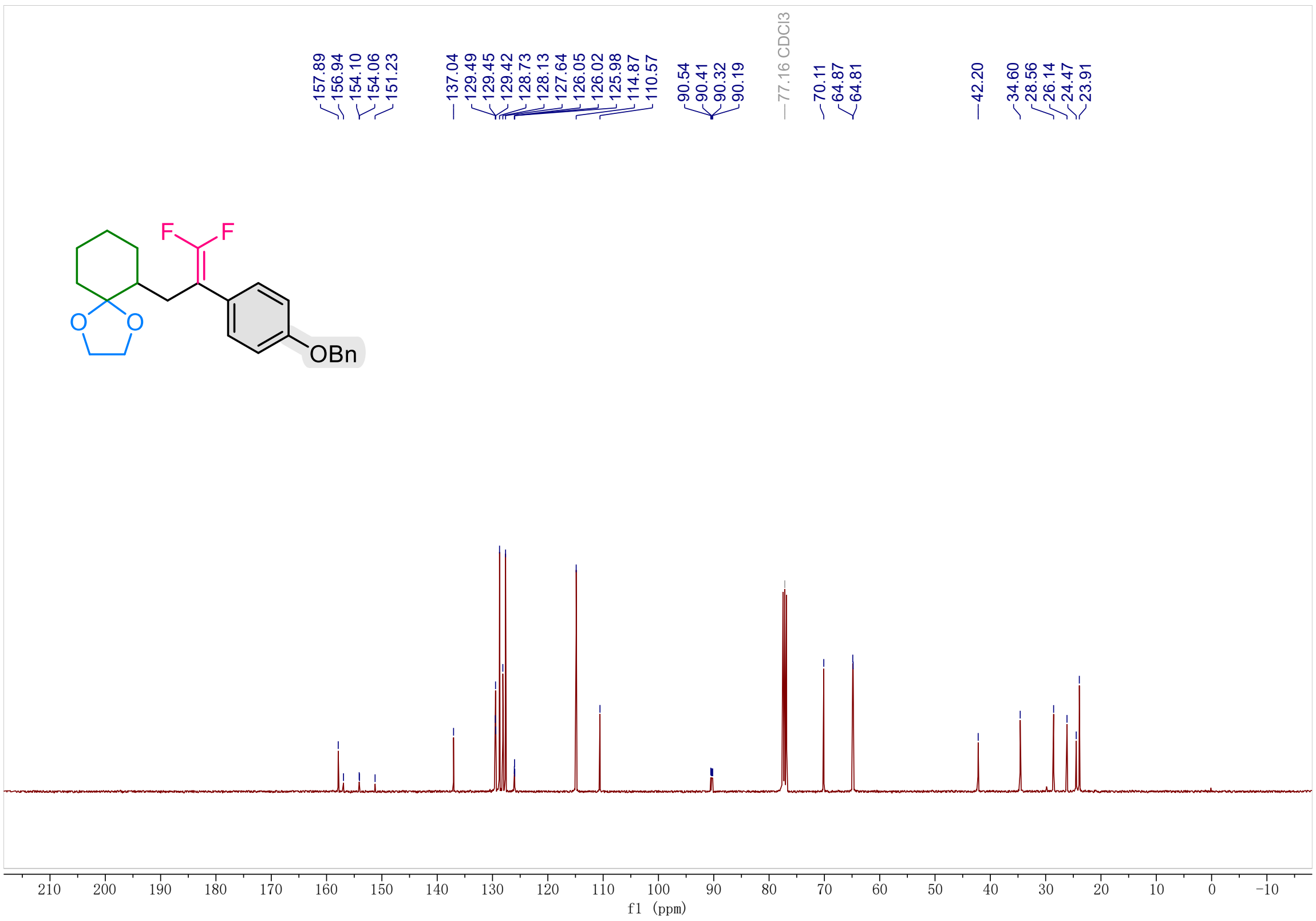

^{13}C NMR spectrum (100 MHz, Chloroform-*d*) of **9d**

6-(2-(4-(Benzyloxy)phenyl)-3,3-difluoroallyl)-1,4-dioxaspiro[4.5]decane (Scheme 2, product 9d)

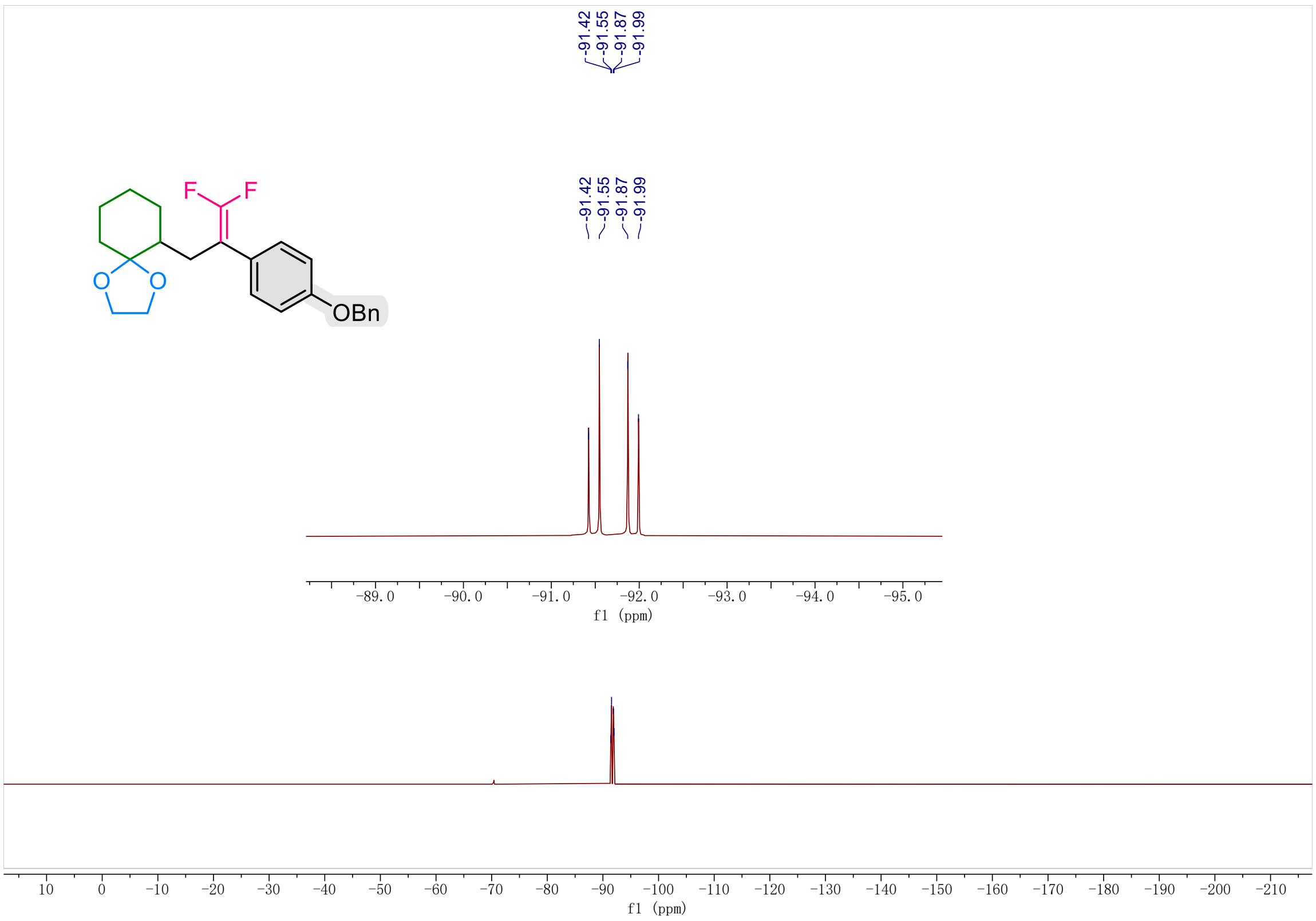

^{19}F NMR spectrum (377 MHz, Chloroform-*d*) of **9d**

6-(3,3-Difluoro-2-(4-methoxyphenyl)allyl)-1,4-dioxaspiro[4.5]decane (Scheme 2, product 9e)

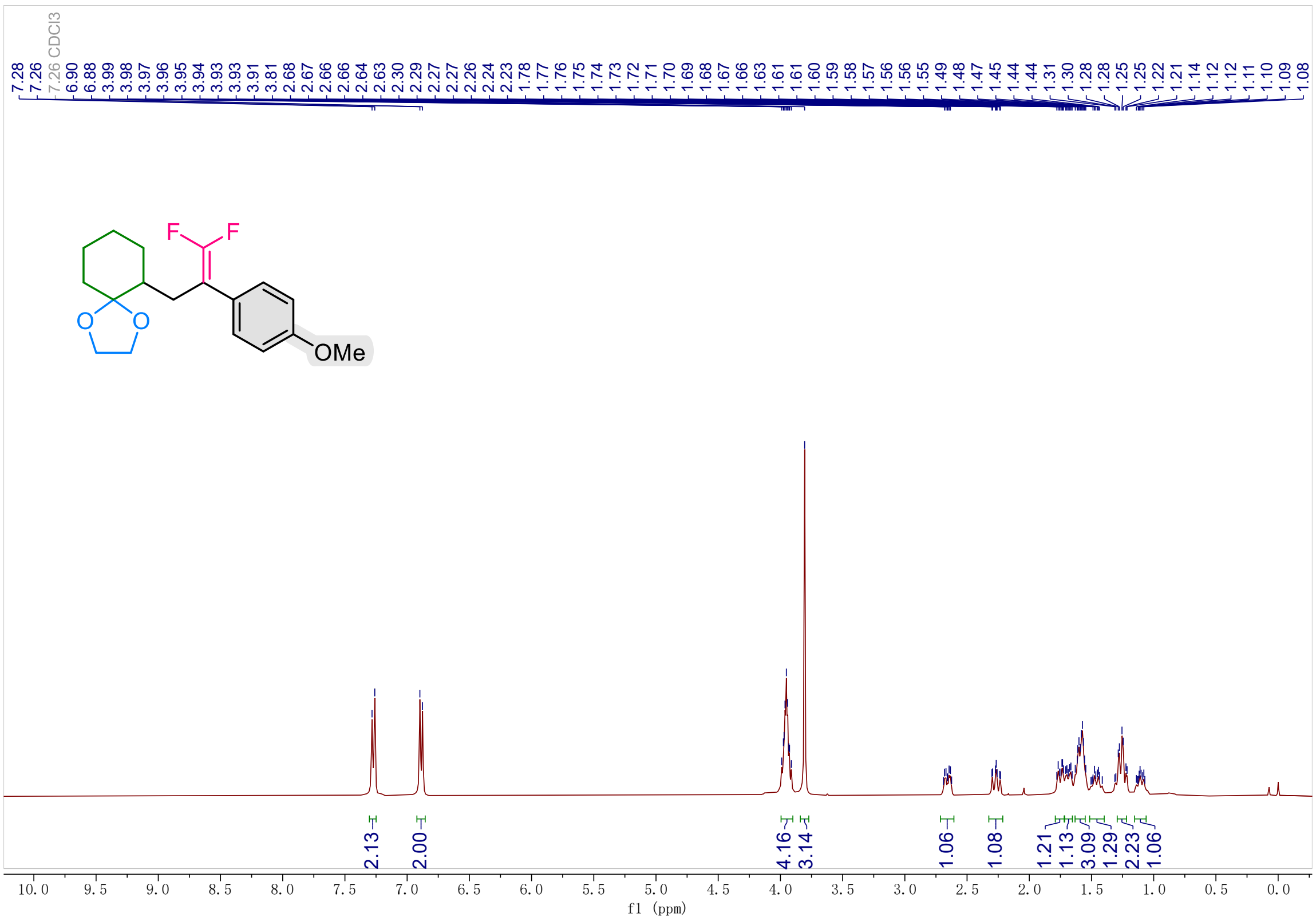

^{1}H NMR spectrum (400 MHz, Chloroform-*d*) of **9e**

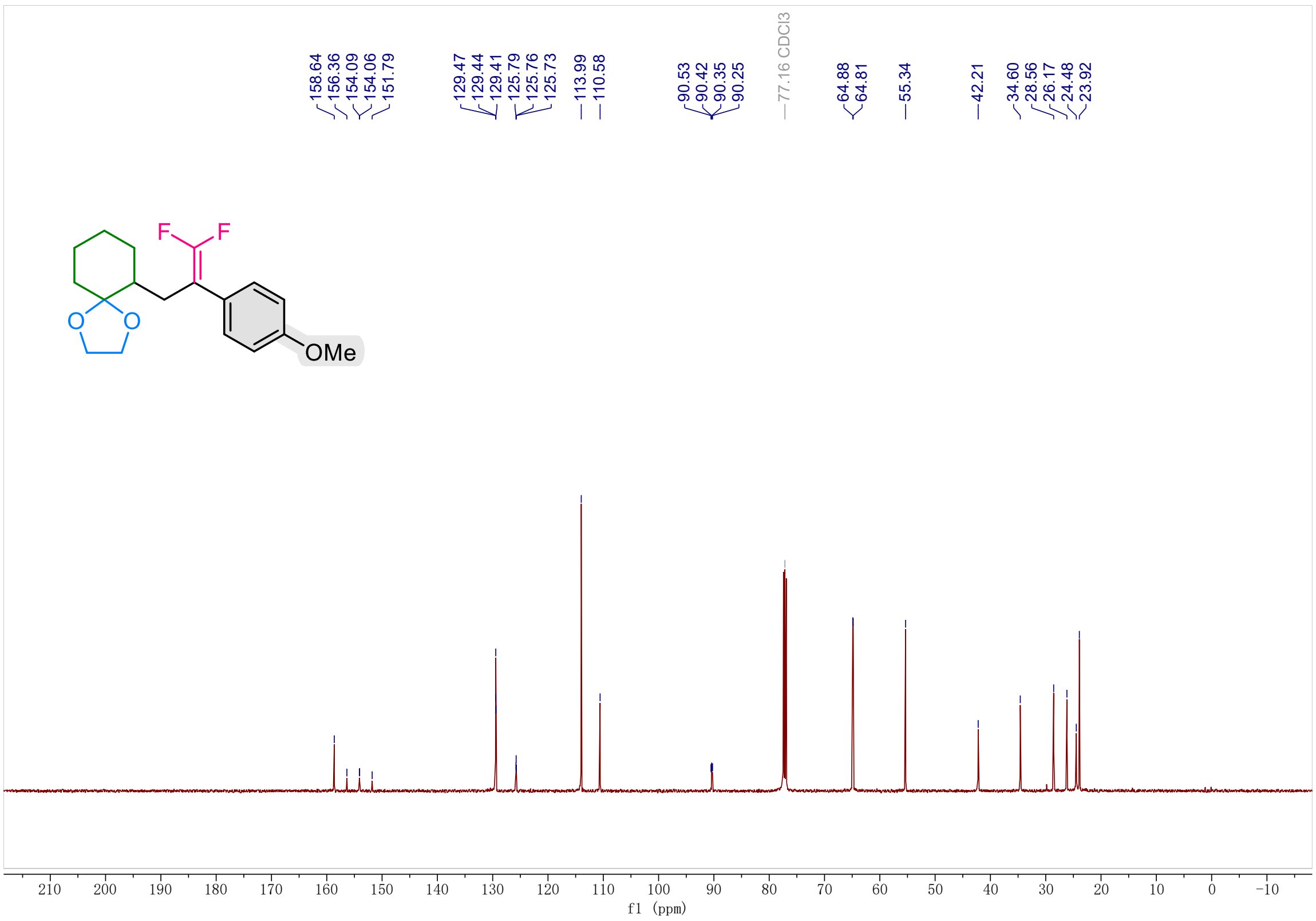

^{13}C NMR spectrum (125 MHz, Chloroform-*d*) of **9e**

6-(3,3-Difluoro-2-(4-methoxyphenyl)allyl)-1,4-dioxaspiro[4.5]decane (Scheme 2, product 9e)

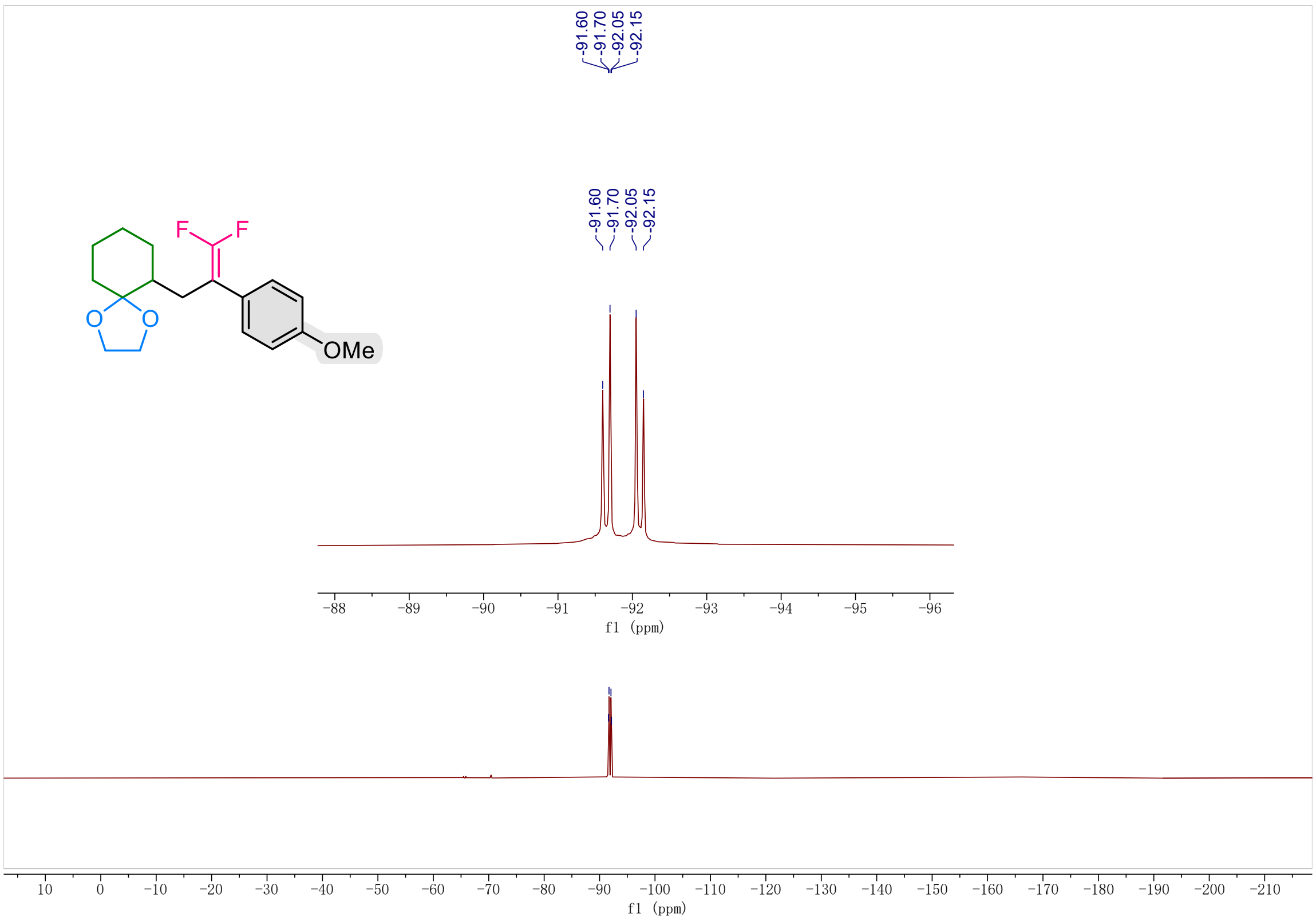

^{19}F NMR spectrum (471 MHz, Chloroform-*d*) of **9e**

6-(3,3-Difluoro-2-(2-methoxyphenyl)allyl)-1,4-dioxaspiro[4.5]decane (Scheme 2, product 9f)

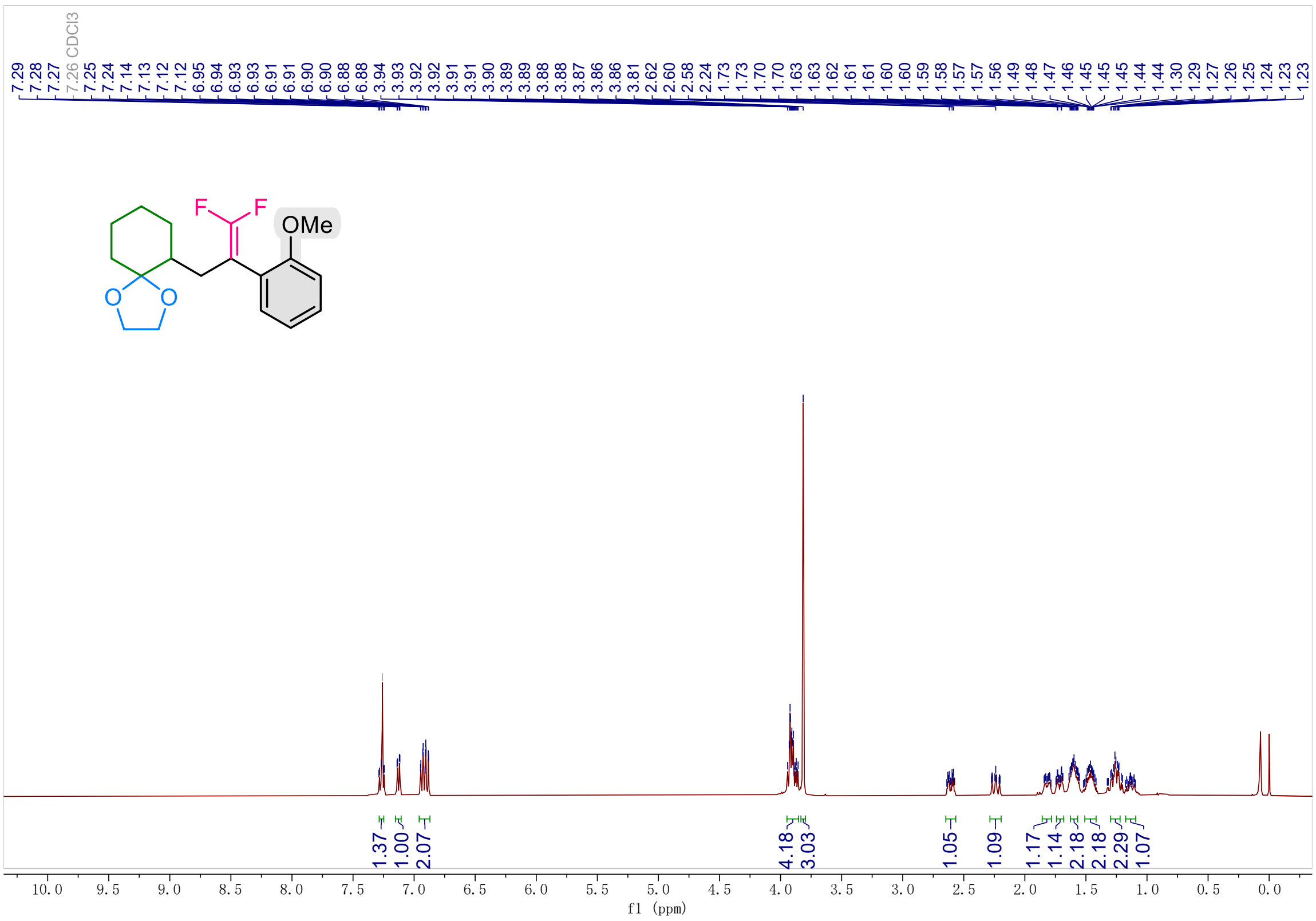

^{1}H NMR spectrum (400 MHz, Chloroform-*d*) of **9f**

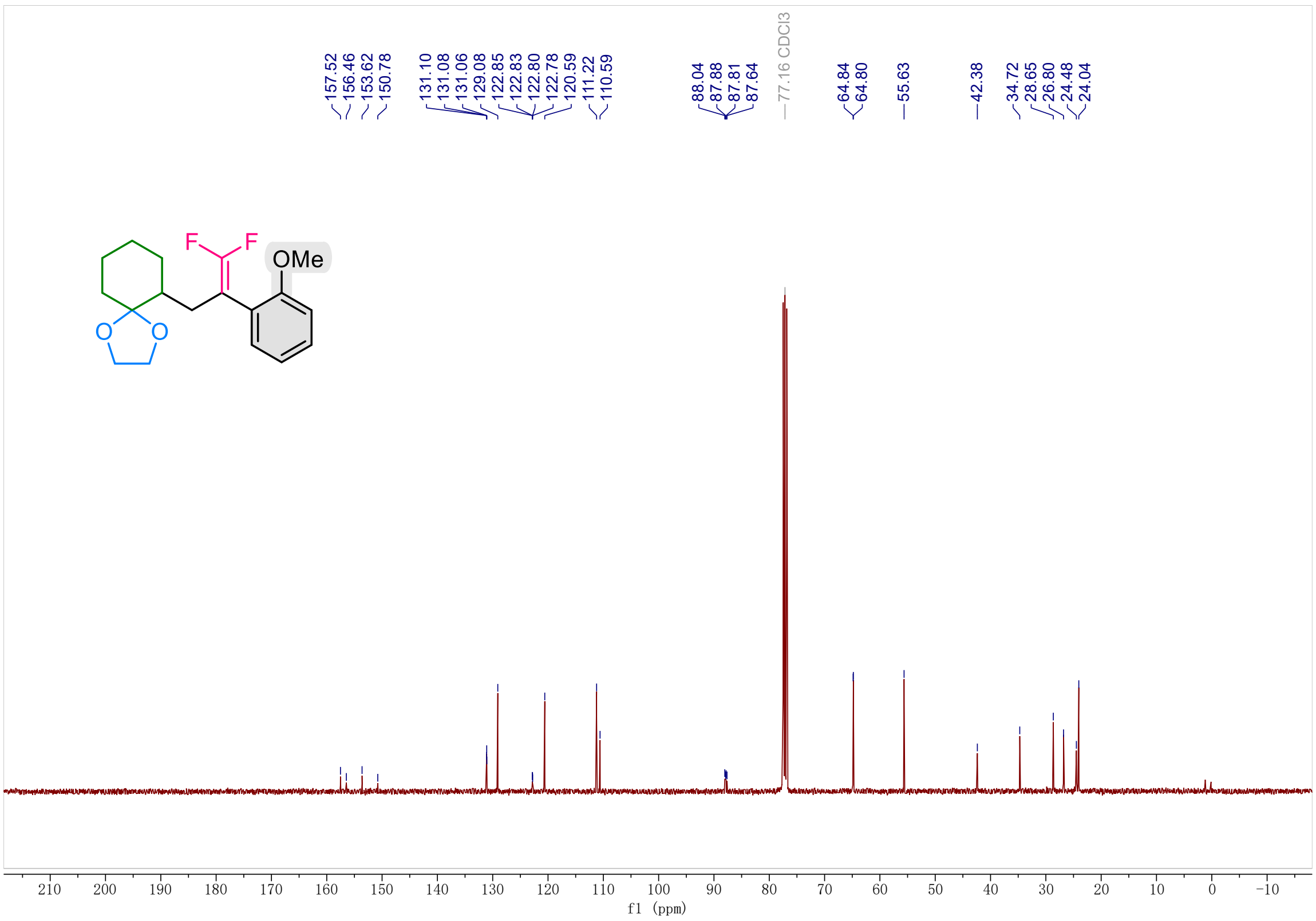

^{13}C NMR spectrum (100 MHz, Chloroform-*d*) of **9f**

6-(3,3-Difluoro-2-(2-methoxyphenyl)allyl)-1,4-dioxaspiro[4.5]decane (Scheme 2, product 9f)

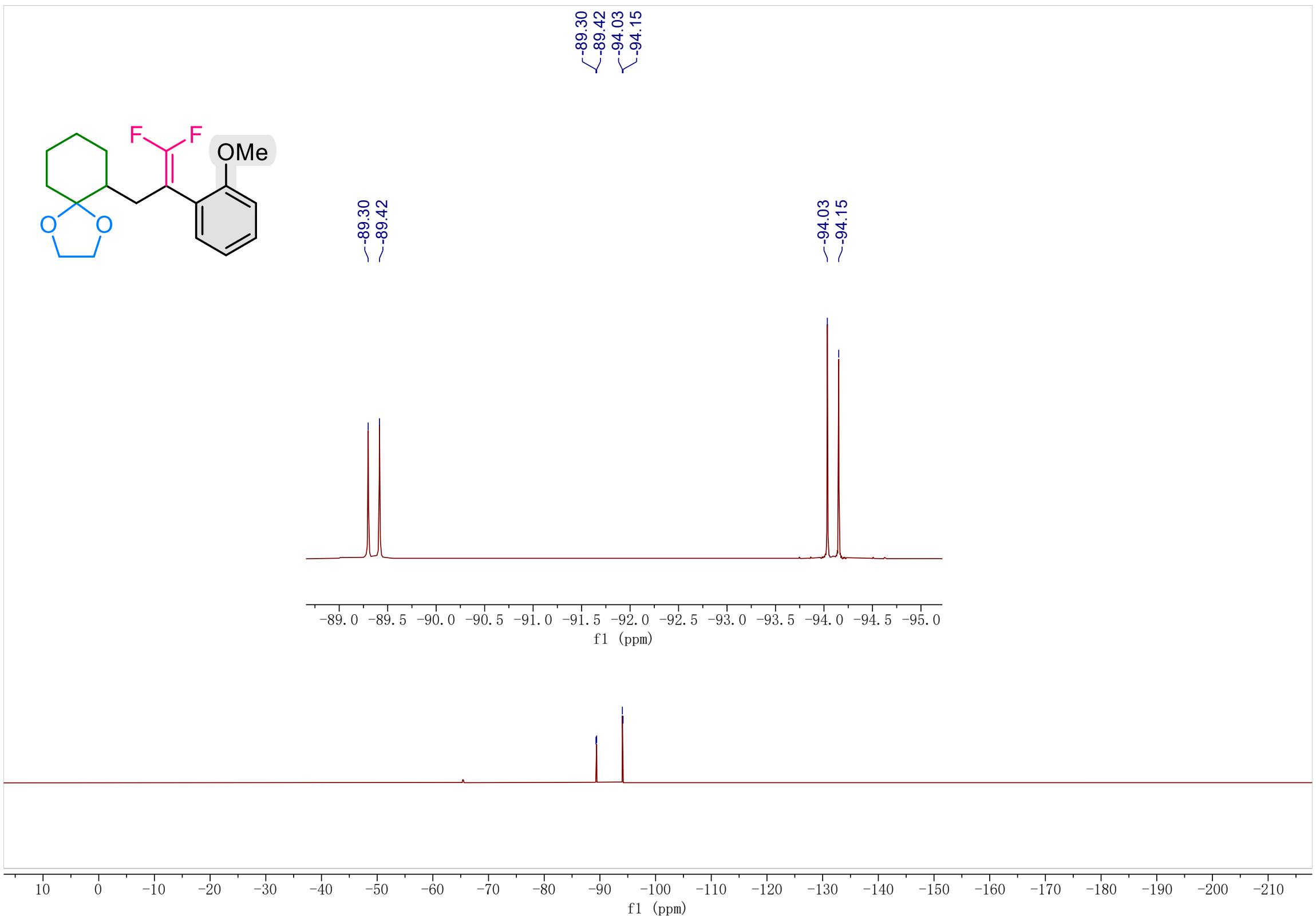

^{19}F NMR spectrum (377 MHz, Chloroform-*d*) of **9f**

6-(3,3-Difluoro-2-(3-methoxyphenyl)allyl)-1,4-dioxaspiro[4.5]decane (Scheme 2, product 9g)

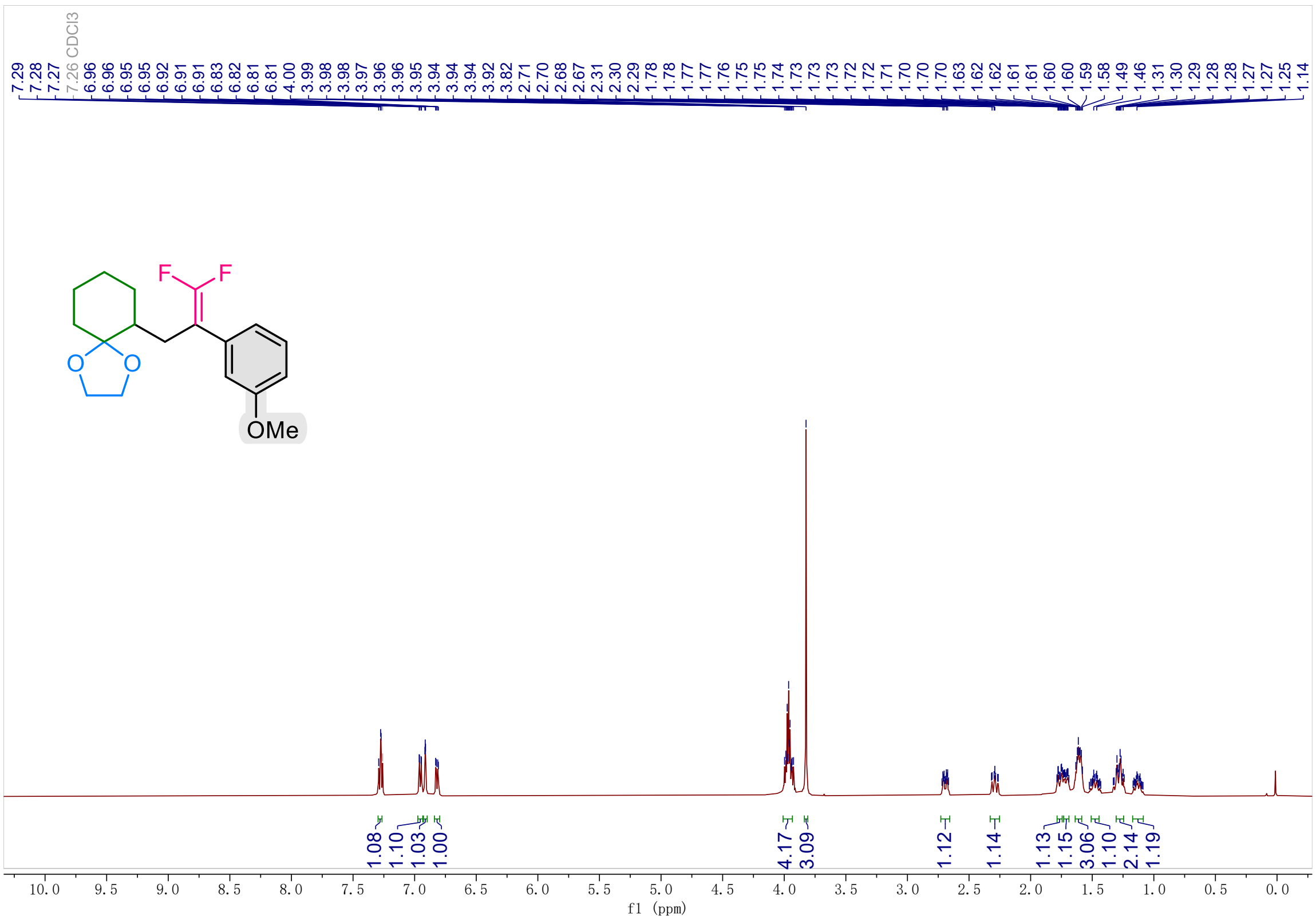

^{1}H NMR spectrum (500 MHz, Chloroform-*d*) of **9g**

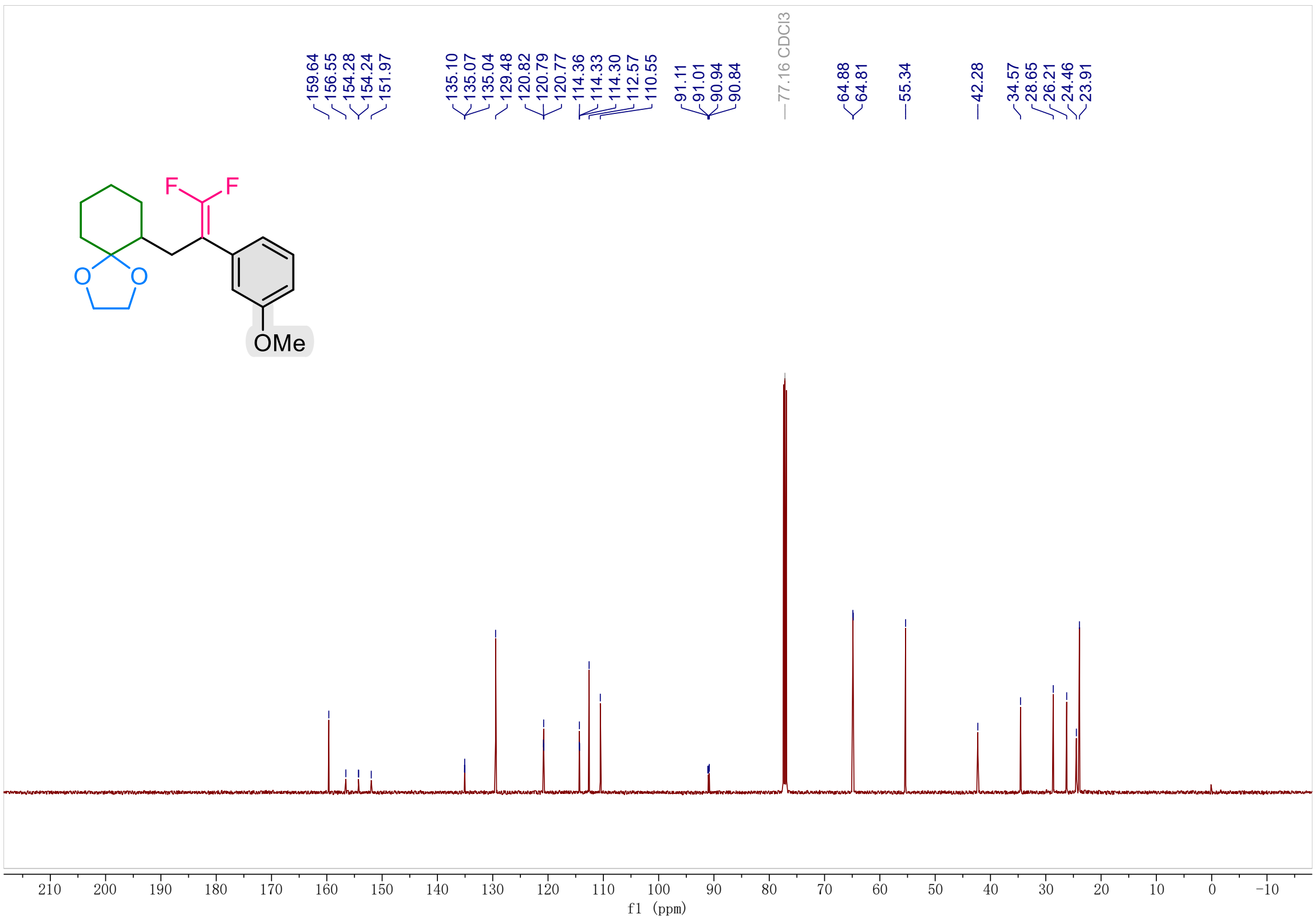

^{13}C NMR spectrum (125 MHz, Chloroform-*d*) of **9g**

6-(3,3-Difluoro-2-(3-methoxyphenyl)allyl)-1,4-dioxaspiro[4.5]decane (Scheme 2, product 9g)

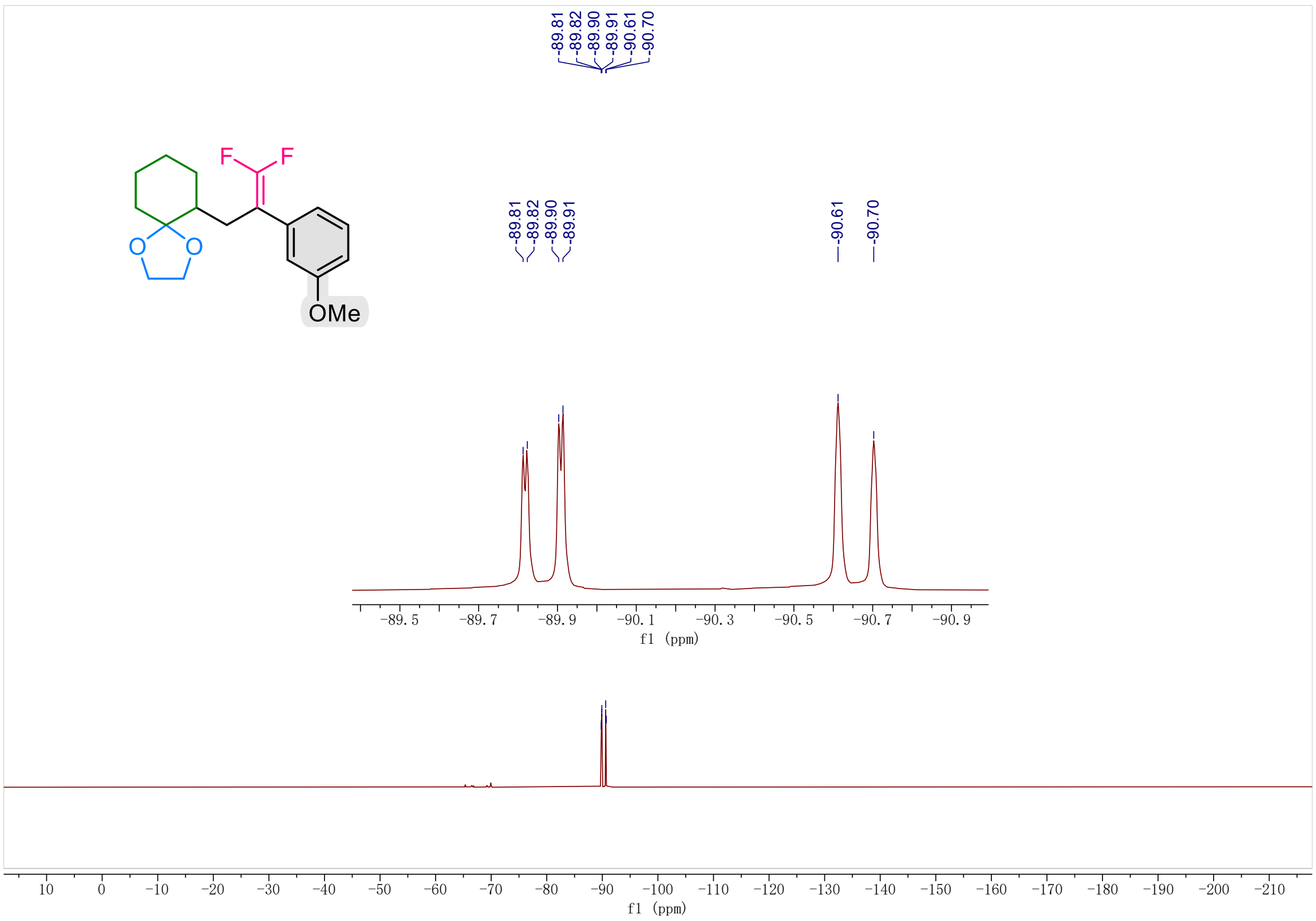

^{19}F NMR spectrum (471 MHz, Chloroform-*d*) of **9g**

3-(1,1-Difluoro-3-(1,4-dioxaspiro[4.5]decan-6-yl)prop-1-en-2-yl)aniline (Scheme 2, product 9h)

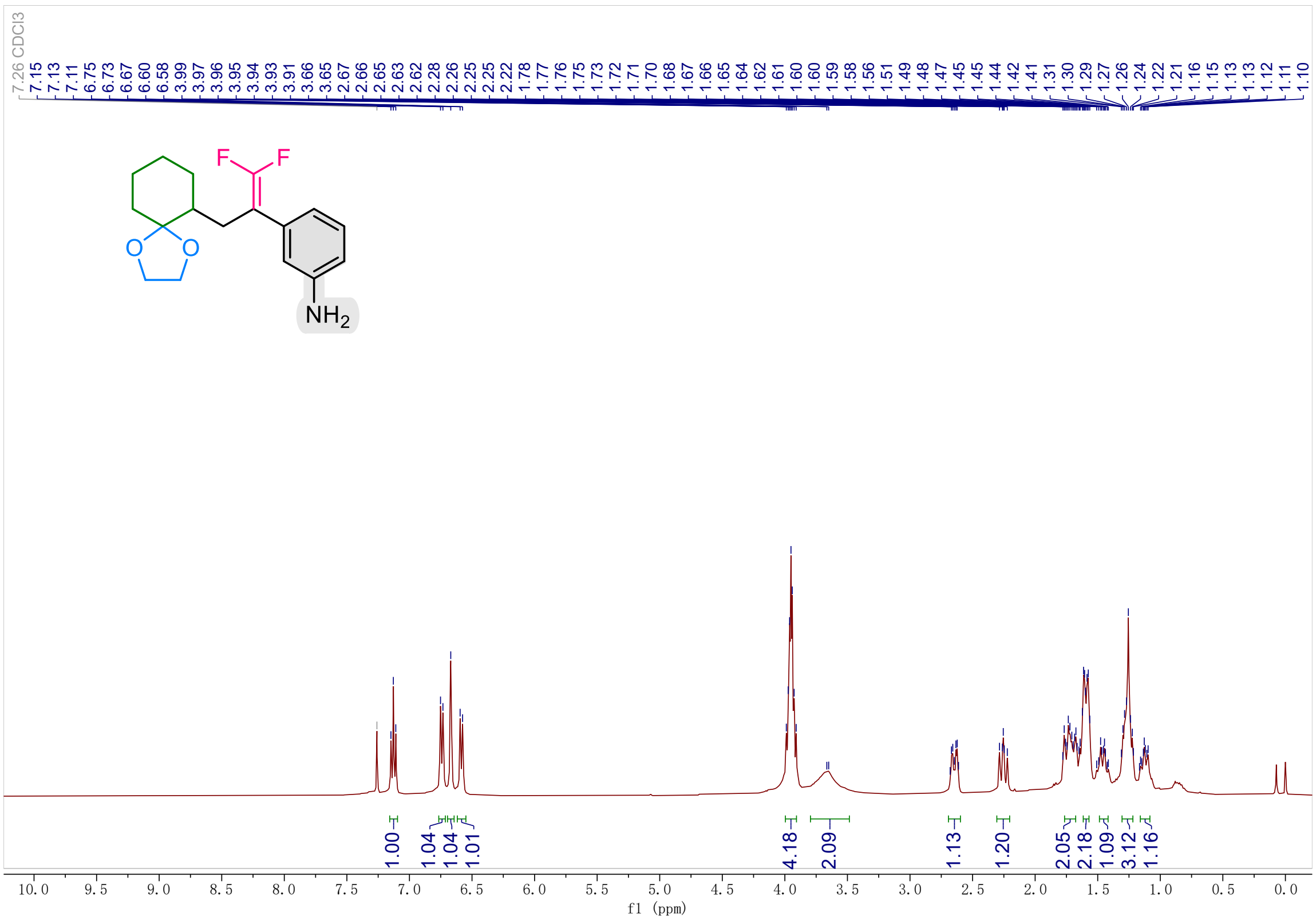

^{1}H NMR spectrum (400 MHz, Chloroform-*d*) of **9h**

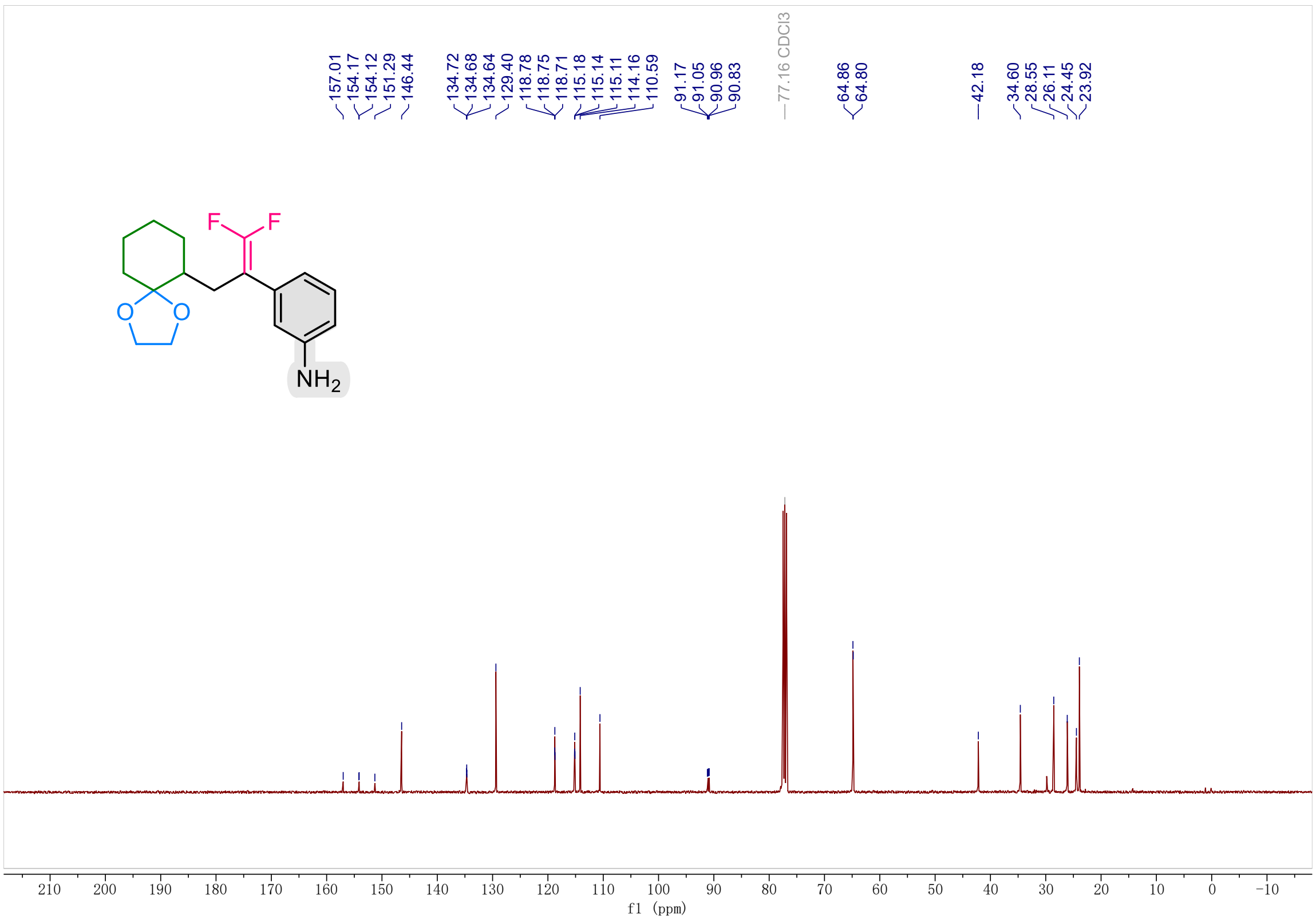

^{13}C NMR spectrum (100 MHz, Chloroform-*d*) of **9h**

3-(1,1-Difluoro-3-(1,4-dioxaspiro[4.5]decan-6-yl)prop-1-en-2-yl)aniline (Scheme 2, product 9h)

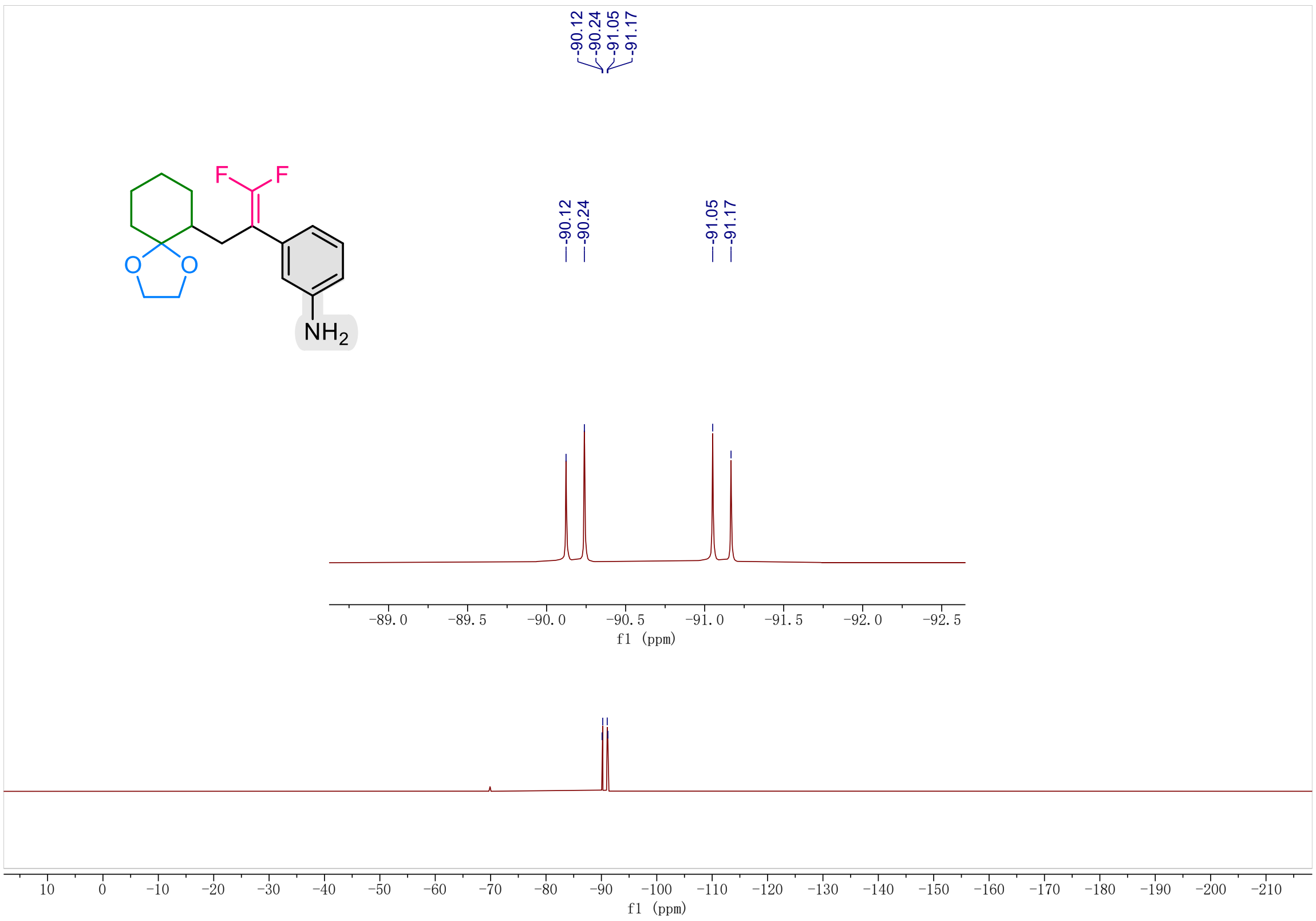

^{19}F NMR spectrum (377 MHz, Chloroform-*d*) of **9h**

6-(3,3-Difluoro-2-(4-(methylsulfonyl)phenyl)allyl)-1,4-dioxaspiro[4.5]decane (Scheme 2, product 9i)

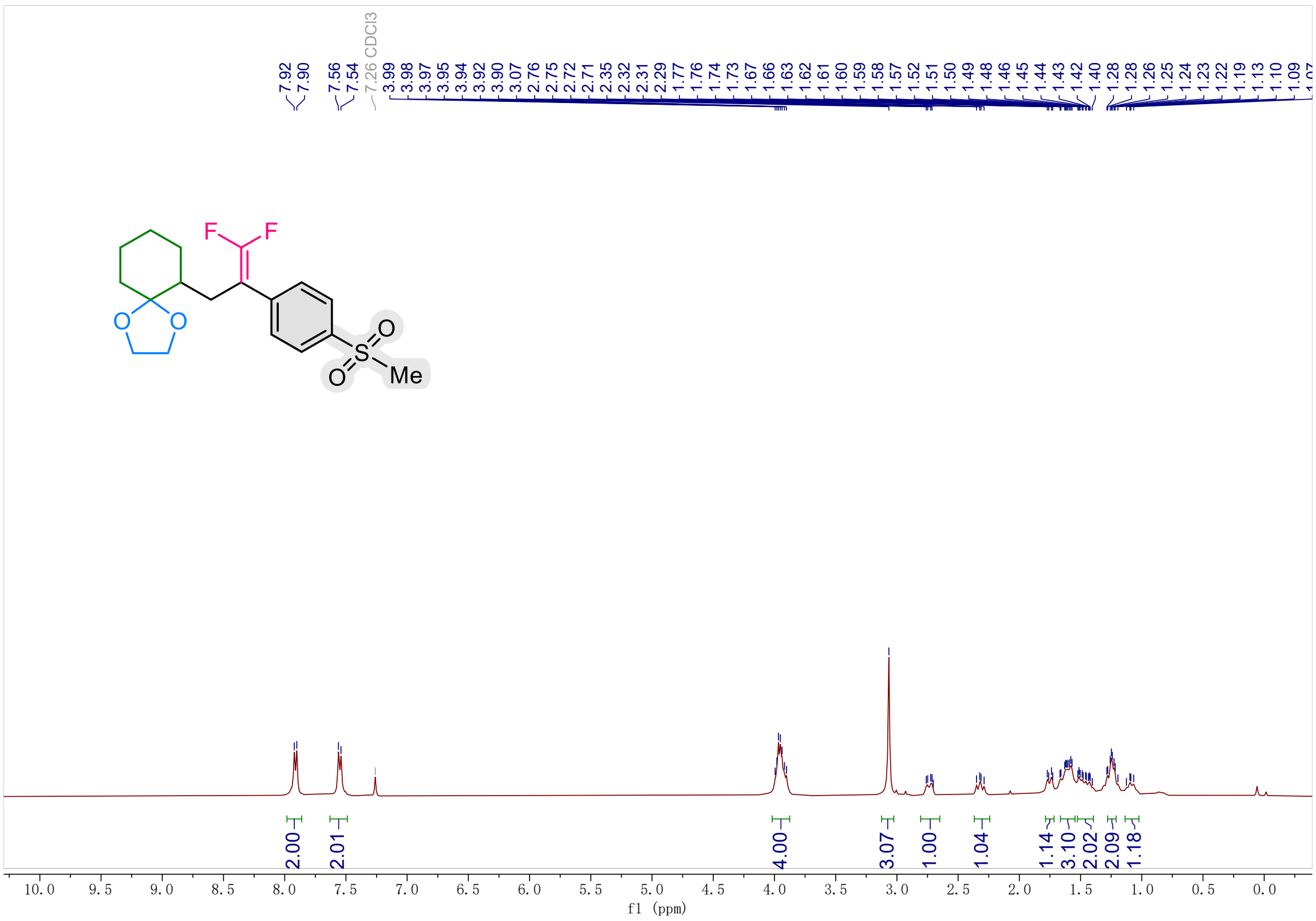

^{1}H NMR spectrum (400 MHz, Chloroform-*d*) of **9i**

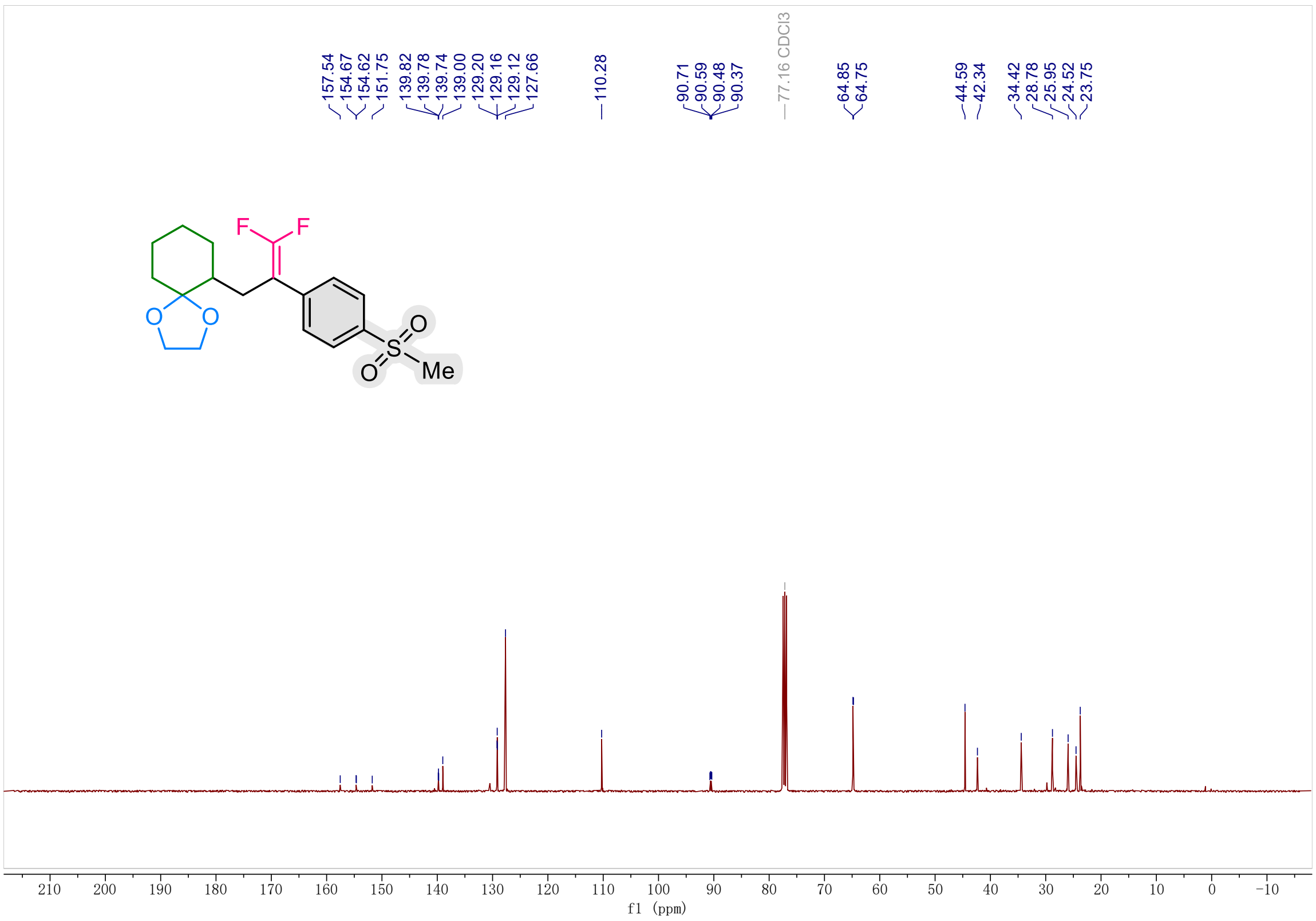

^{13}C NMR spectrum (100 MHz, Chloroform-*d*) of **9i**

6-(3,3-Difluoro-2-(4-(methylsulfonyl)phenyl)allyl)-1,4-dioxaspiro[4.5]decane (Scheme 2, product 9i)

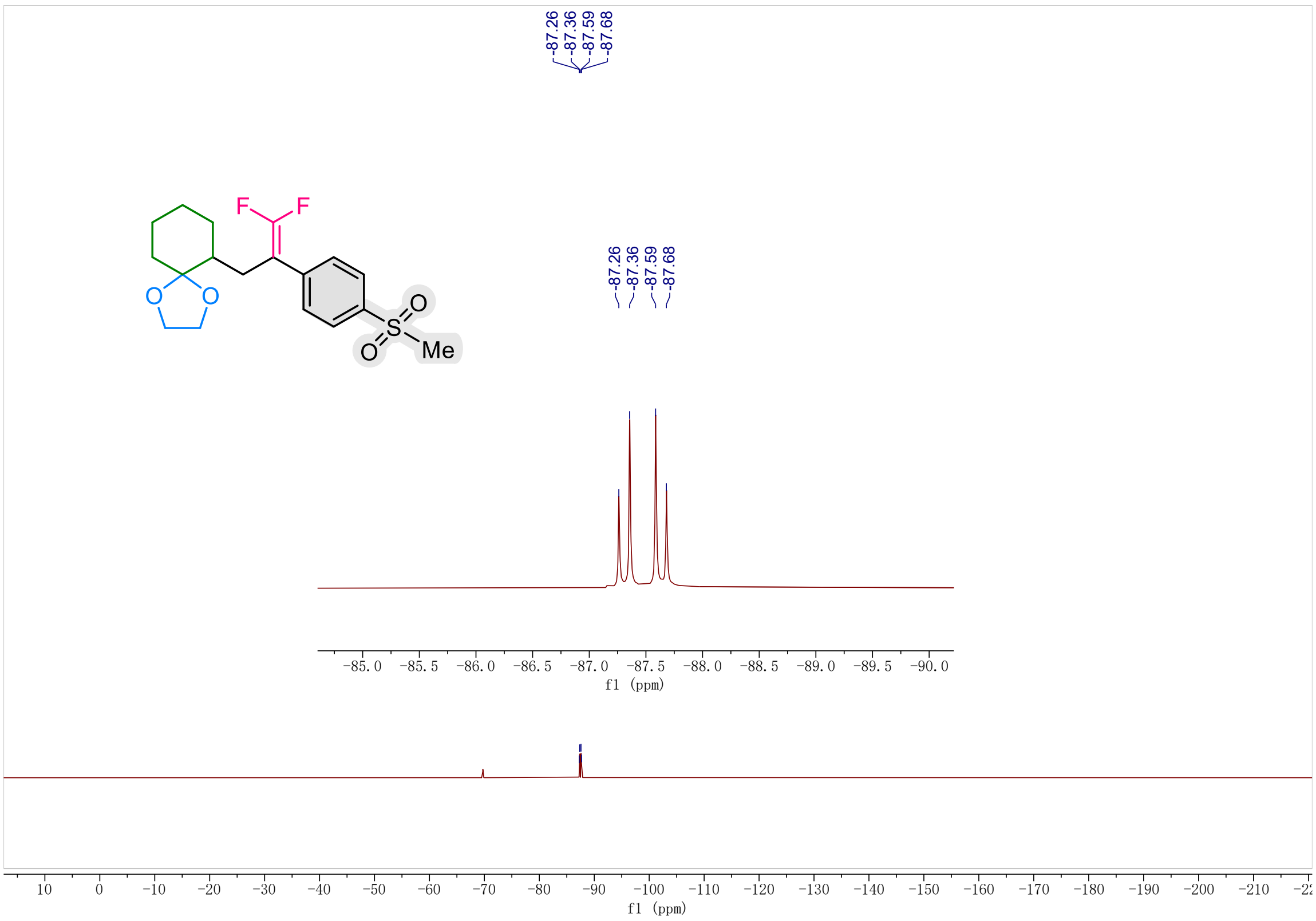

^{19}F NMR spectrum (377 MHz, Chloroform-*d*) of **9i**

6-(3,3-Difluoro-2-(naphthalen-2-yl)allyl)-1,4-dioxaspiro[4.5]decane (Scheme 2, product 9j)

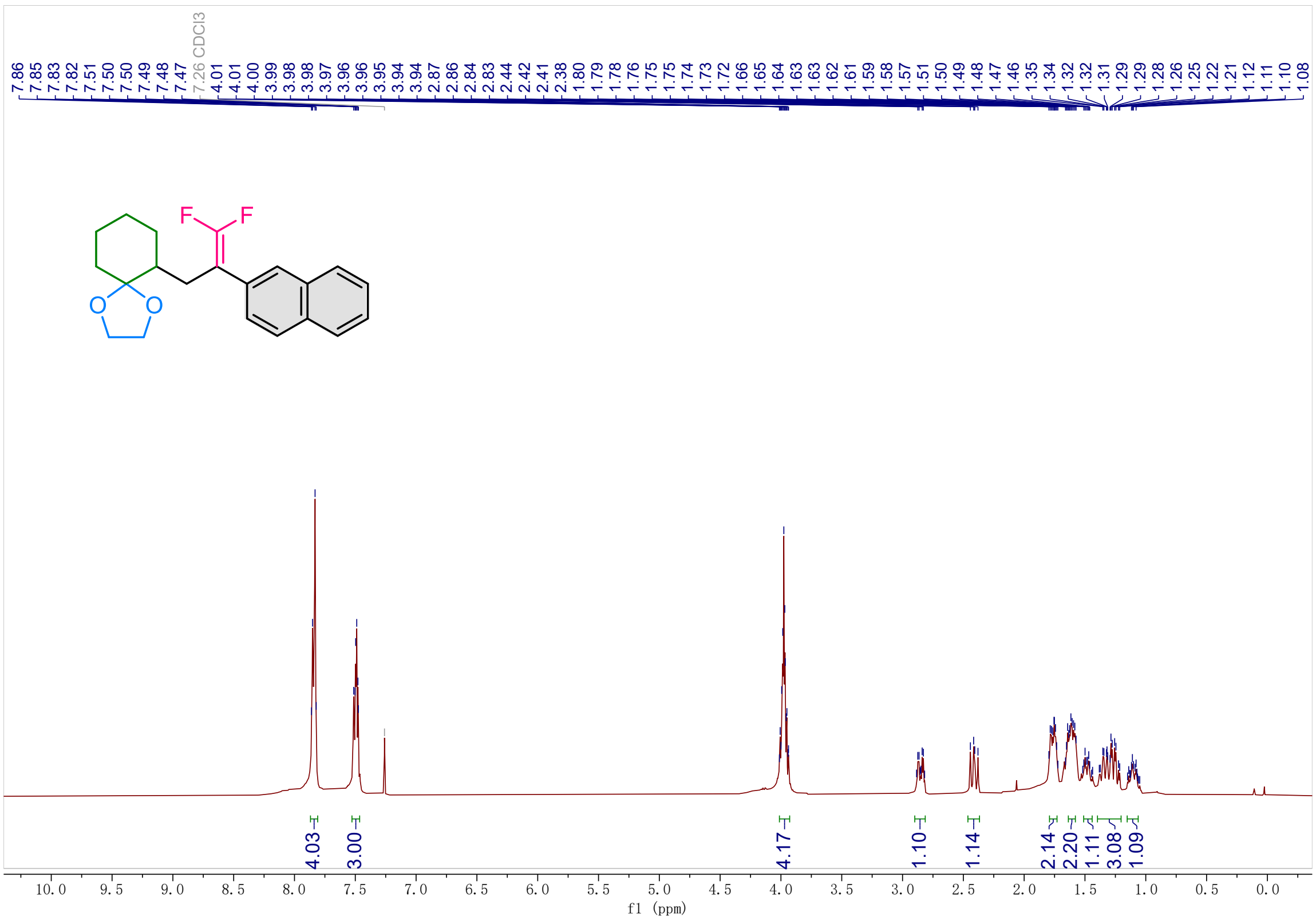

^{1}H NMR spectrum (400 MHz, Chloroform-*d*) of **9j**

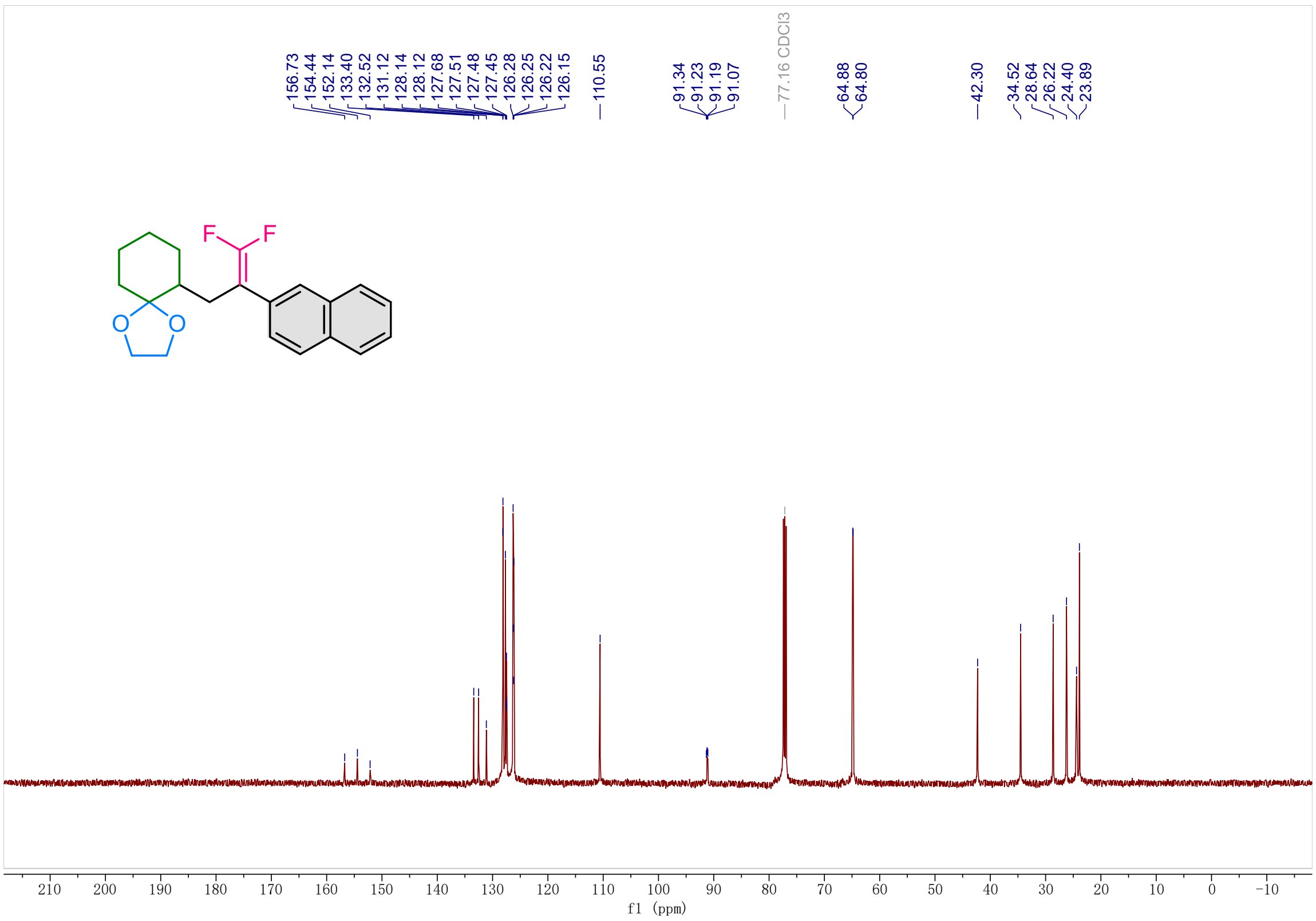

^{13}C NMR spectrum (125 MHz, Chloroform-*d*) of **9j**

6-(3,3-Difluoro-2-(naphthalen-2-yl)allyl)-1,4-dioxaspiro[4.5]decane (Scheme 2, product 9j)

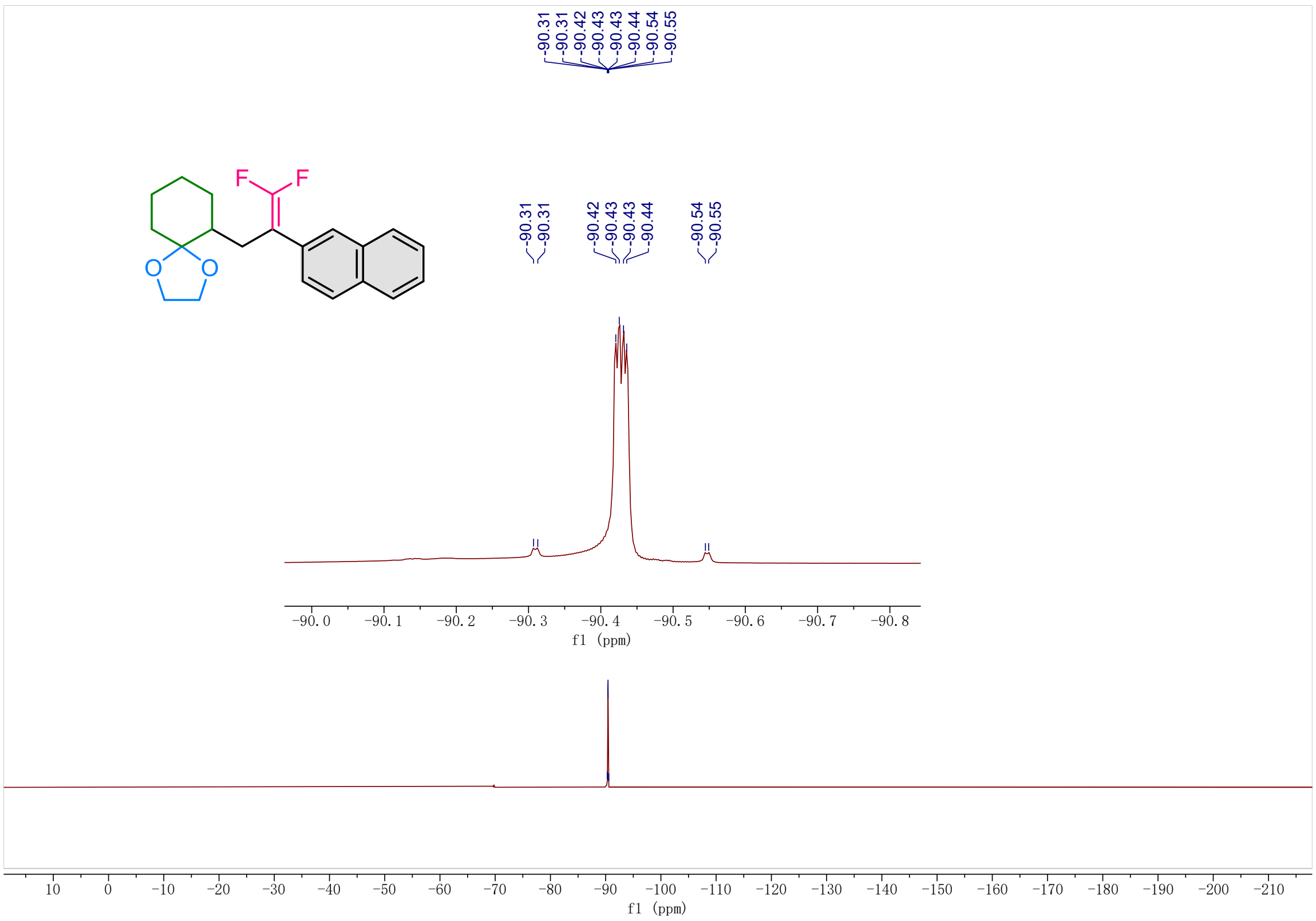

^{19}F NMR spectrum (377 MHz, Chloroform-*d*) of **9j**

4-(1,1-Difluoro-3-(1,4-dioxaspiro[4.5]decan-6-yl)prop-1-en-2-yl)dibenzo[*b,d*]furan (Scheme 2, product 9k)

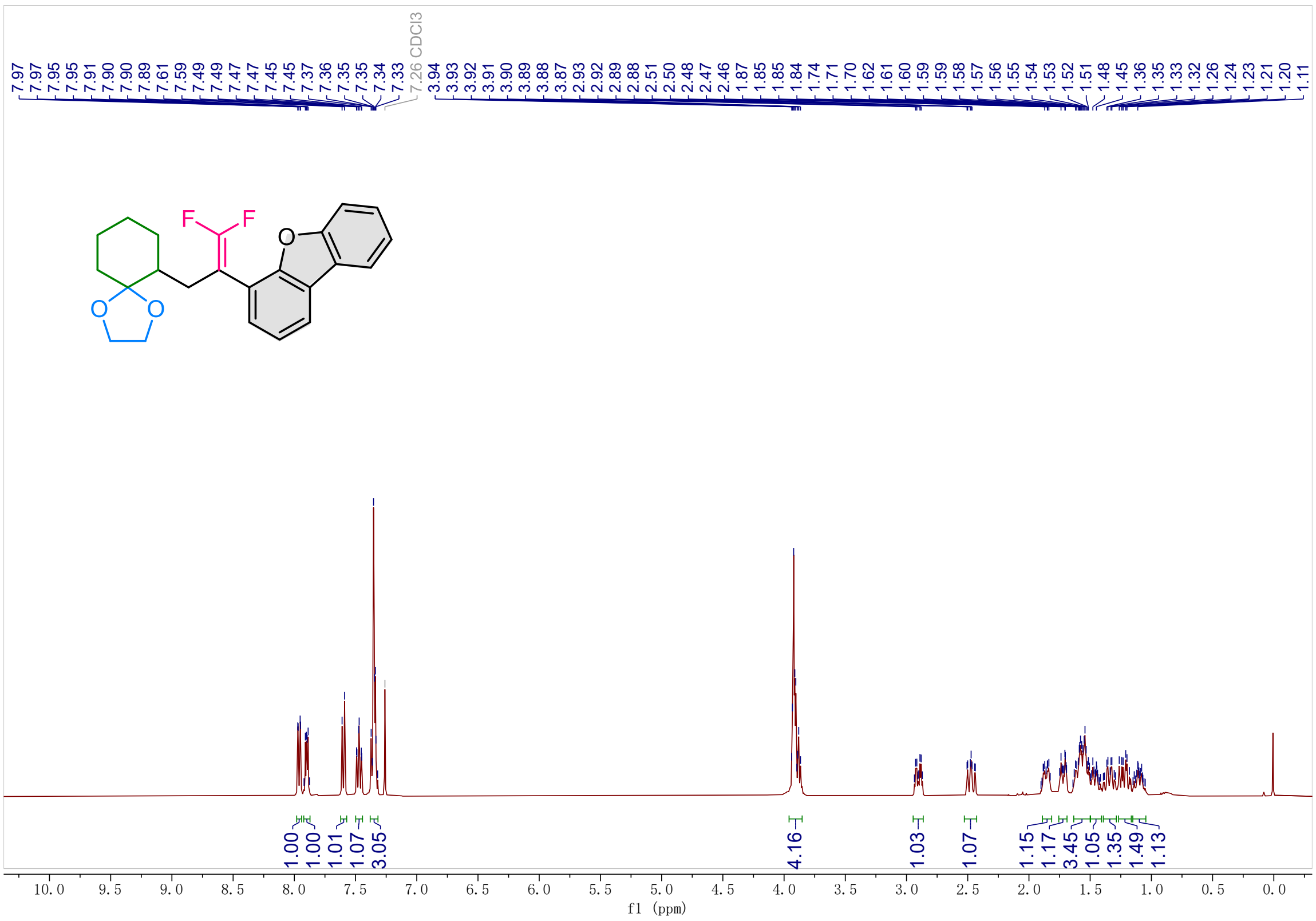

^{1}H NMR spectrum (400 MHz, Chloroform-*d*) of **9k**

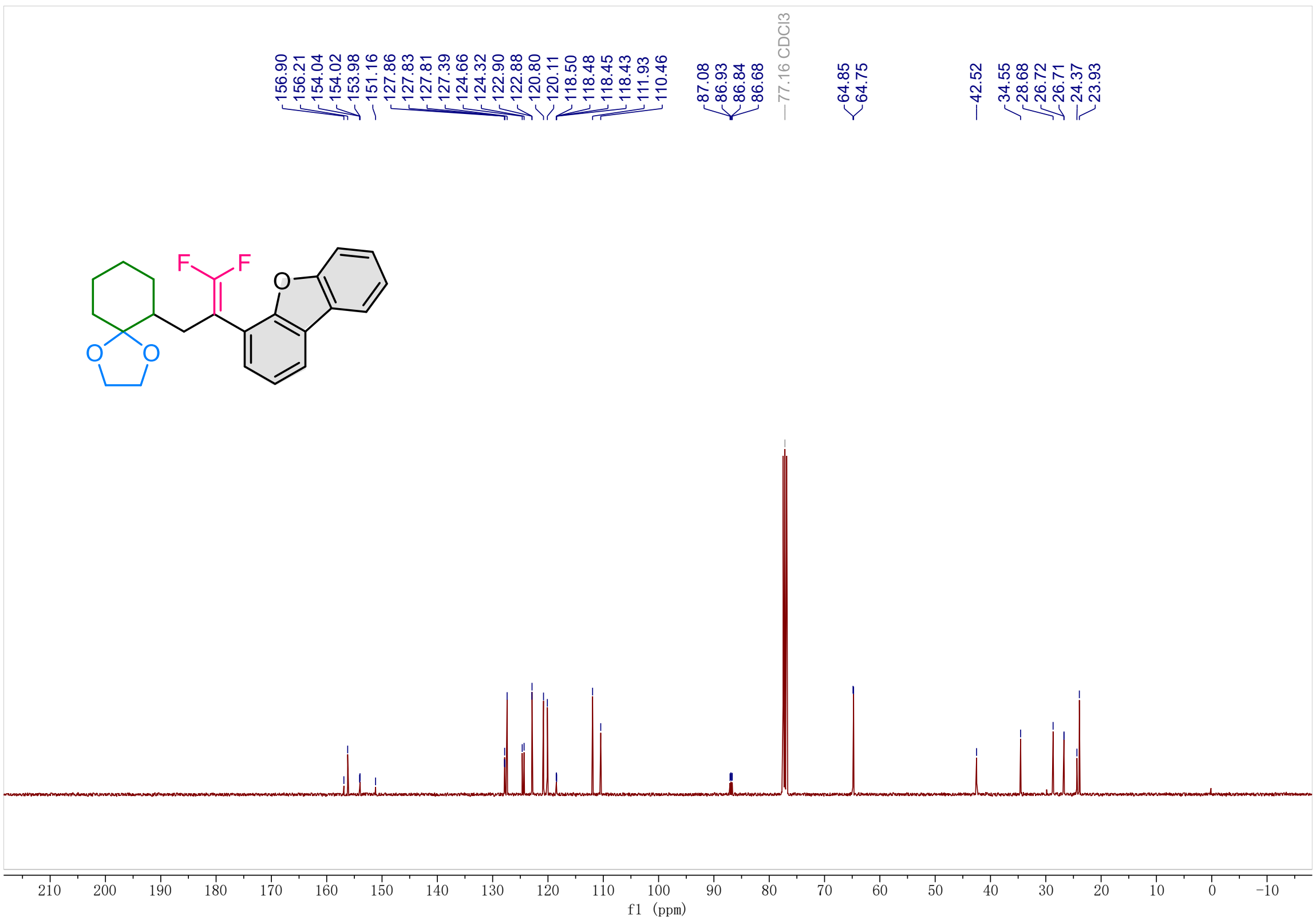

^{13}C NMR spectrum (100 MHz, Chloroform-*d*) of **9k**

4-(1,1-Difluoro-3-(1,4-dioxaspiro[4.5]decan-6-yl)prop-1-en-2-yl)dibenzo[*b,d*]furan (Scheme 2, product 9k)

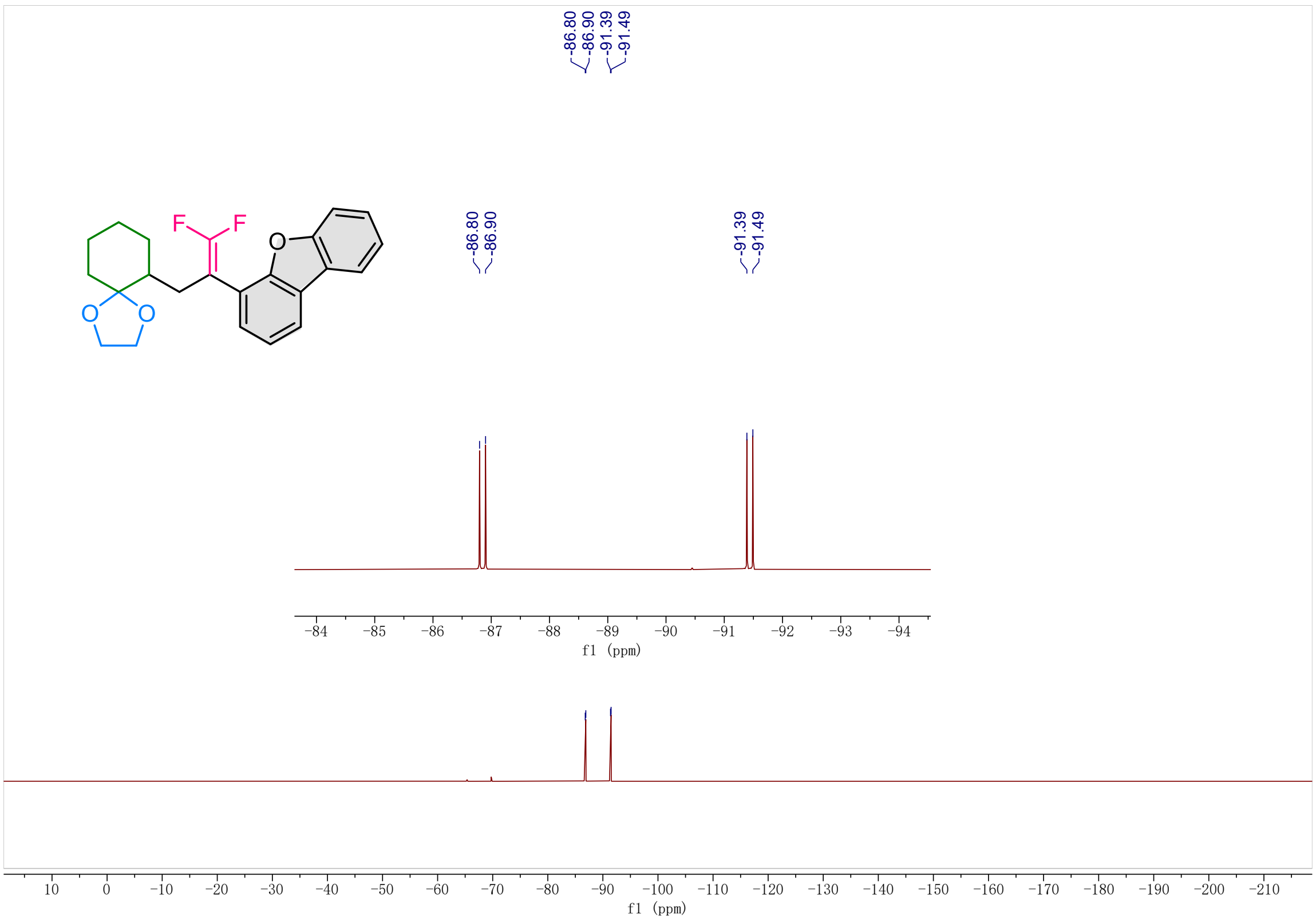

^{19}F NMR spectrum (377 MHz, Chloroform-*d*) of **9k**

6-(2-(Benzo[*b*]thiophen-2-yl)-3,3-difluoroallyl)-1,4-dioxaspiro[4.5]decane (Scheme 2, product 9l)

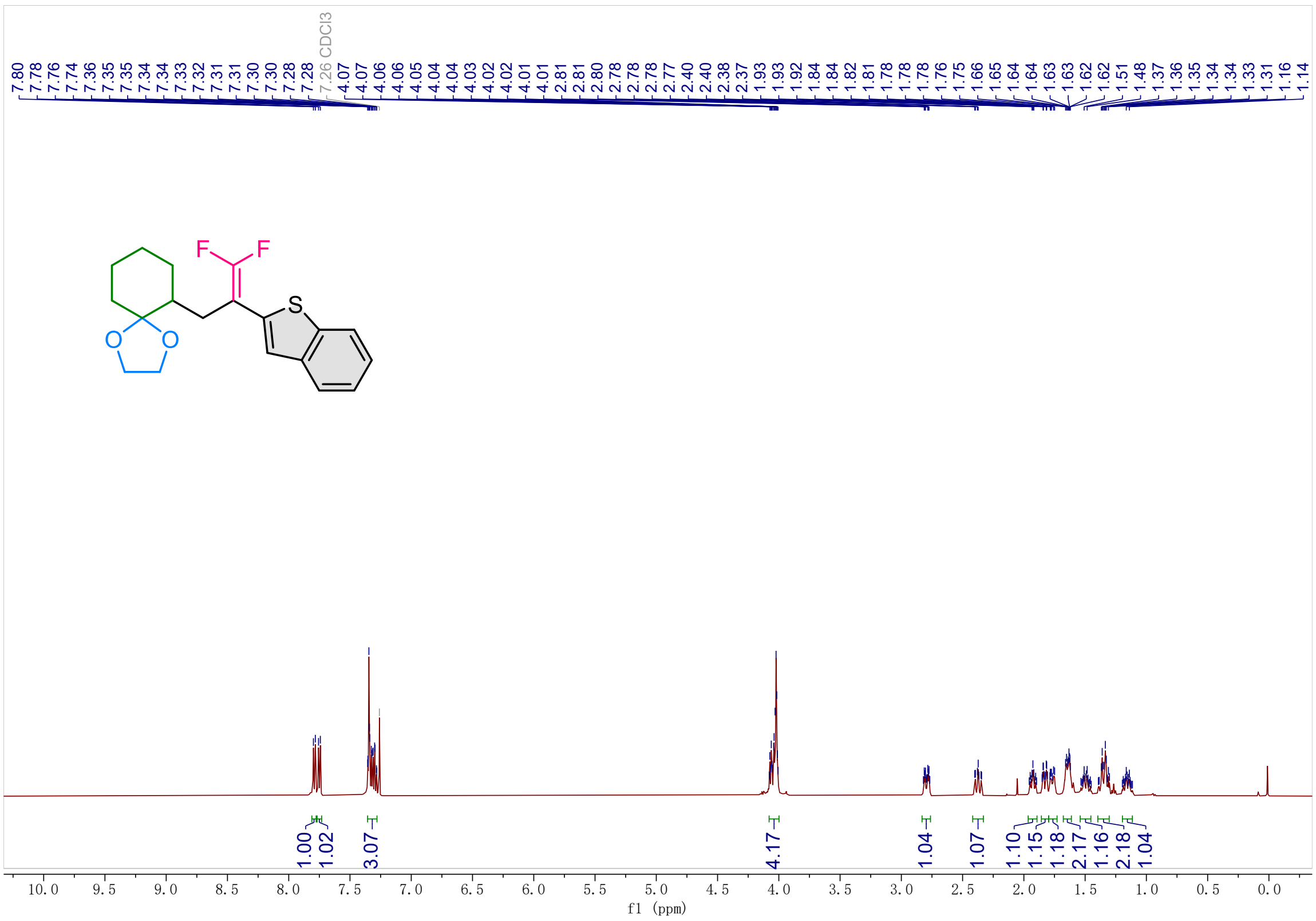

^{1}H NMR spectrum (500 MHz, Chloroform-*d*) of **9l**

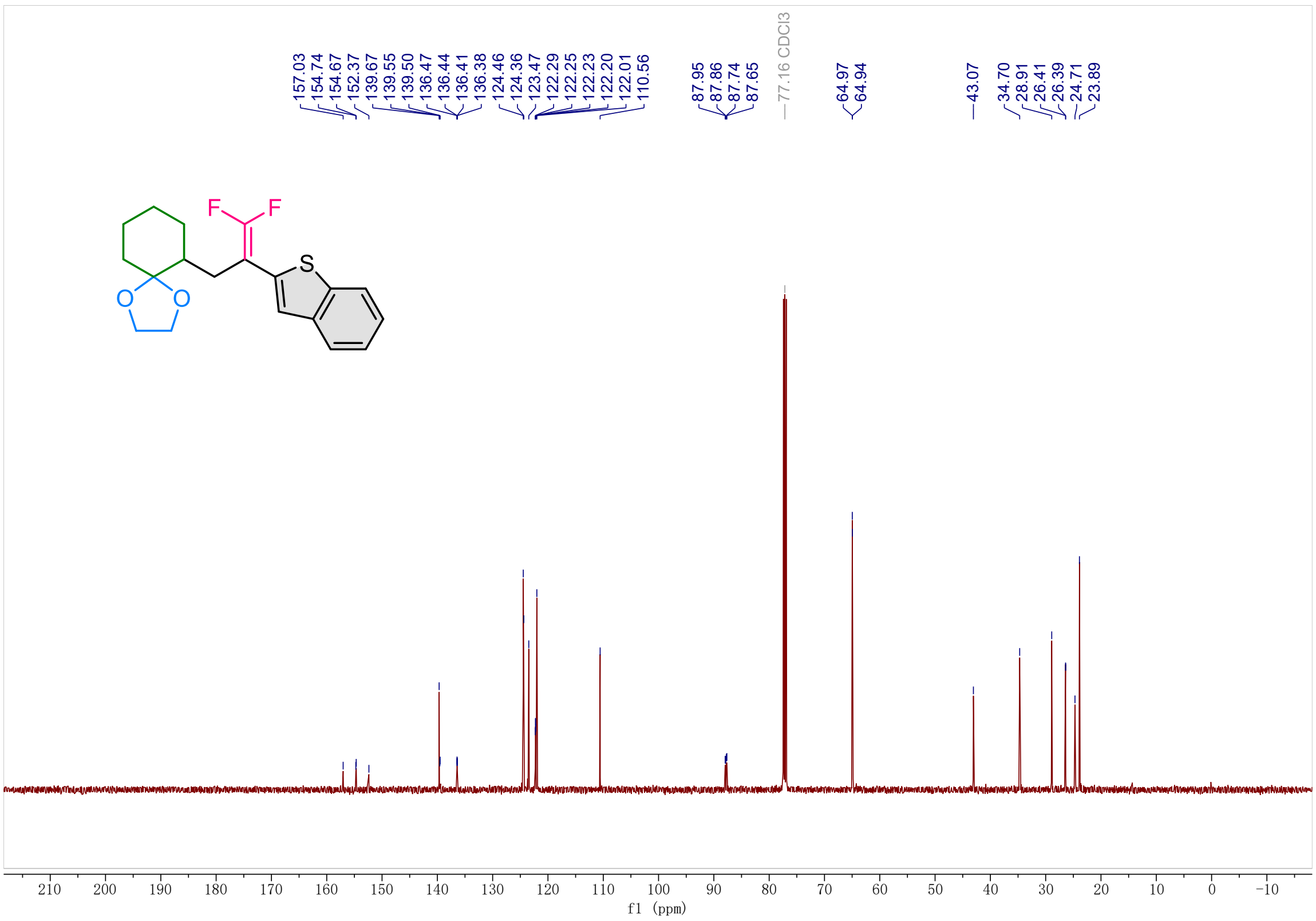

^{13}C NMR spectrum (125 MHz, Chloroform-*d*) of **9l**

6-(2-(Benzo[*b*]thiophen-2-yl)-3,3-difluoroallyl)-1,4-dioxaspiro[4.5]decane (Scheme 2, product 9l)

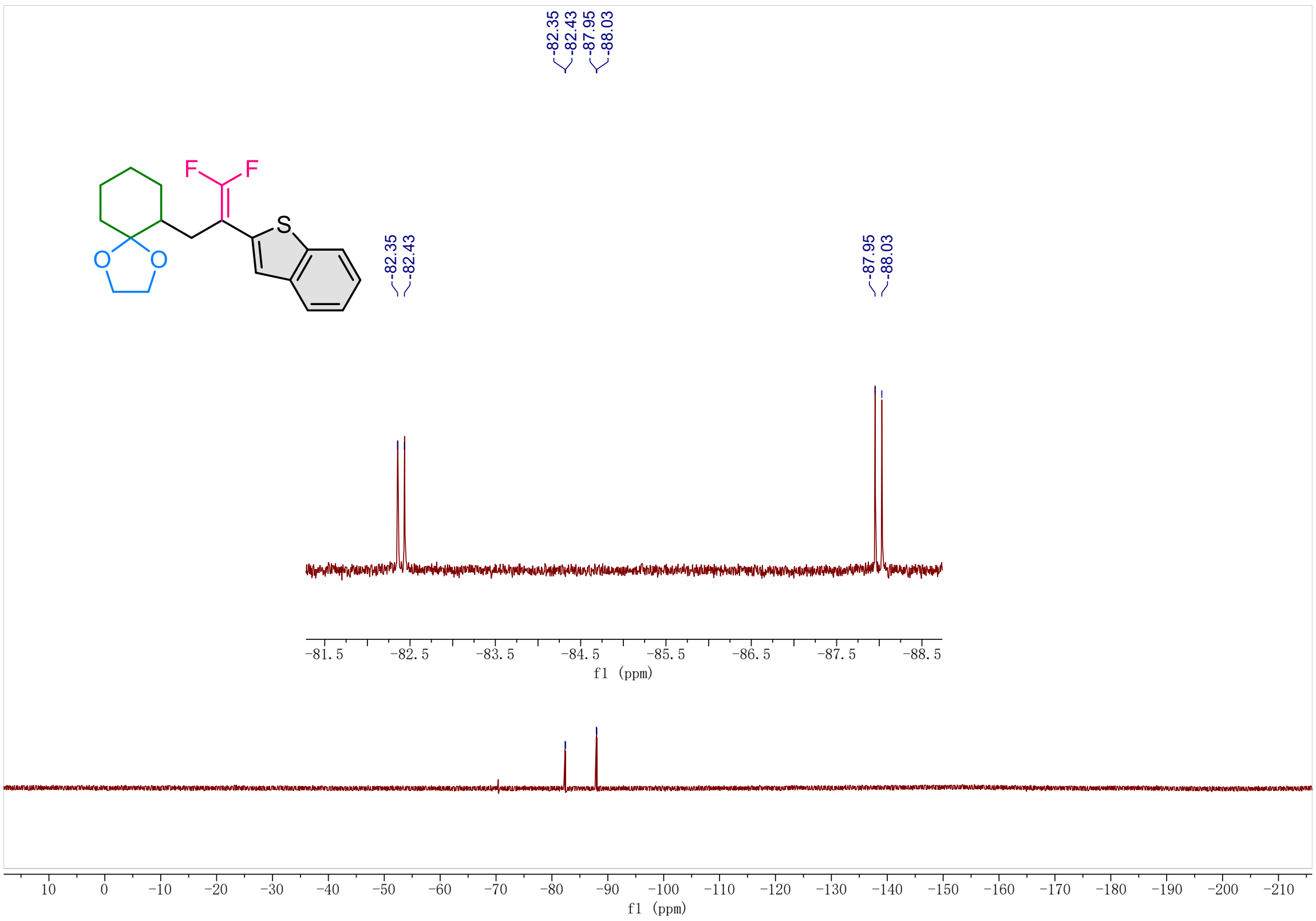

^{19}F NMR spectrum (471 MHz, Chloroform-*d*) of **9l**

8-(1,1-Difluoro-3-(1,4-dioxaspiro[4.5]decan-6-yl)prop-1-en-2-yl)quinoline (Scheme 2, product 9m)

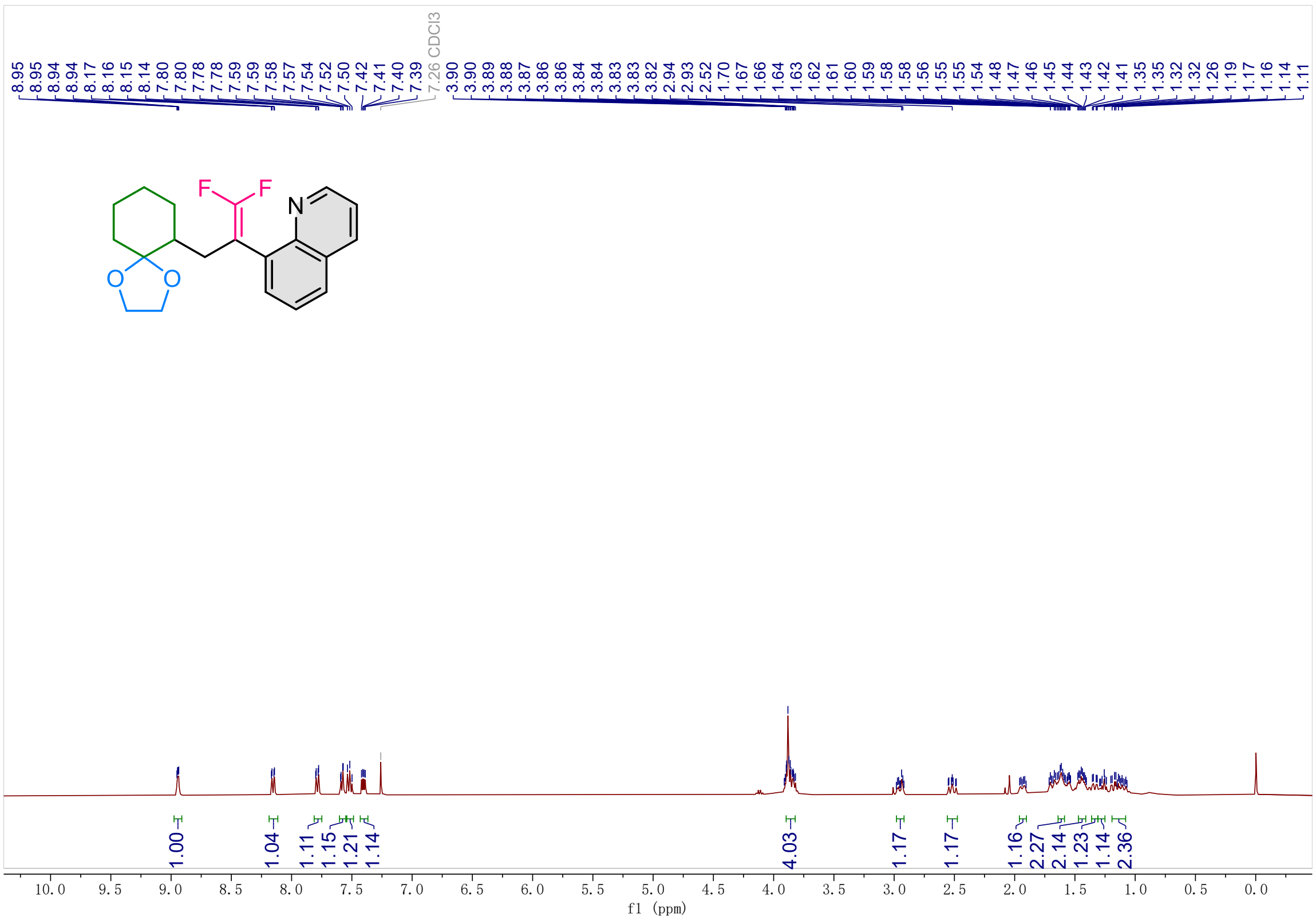

^{1}H NMR spectrum (400 MHz, Chloroform-*d*) of **9m**

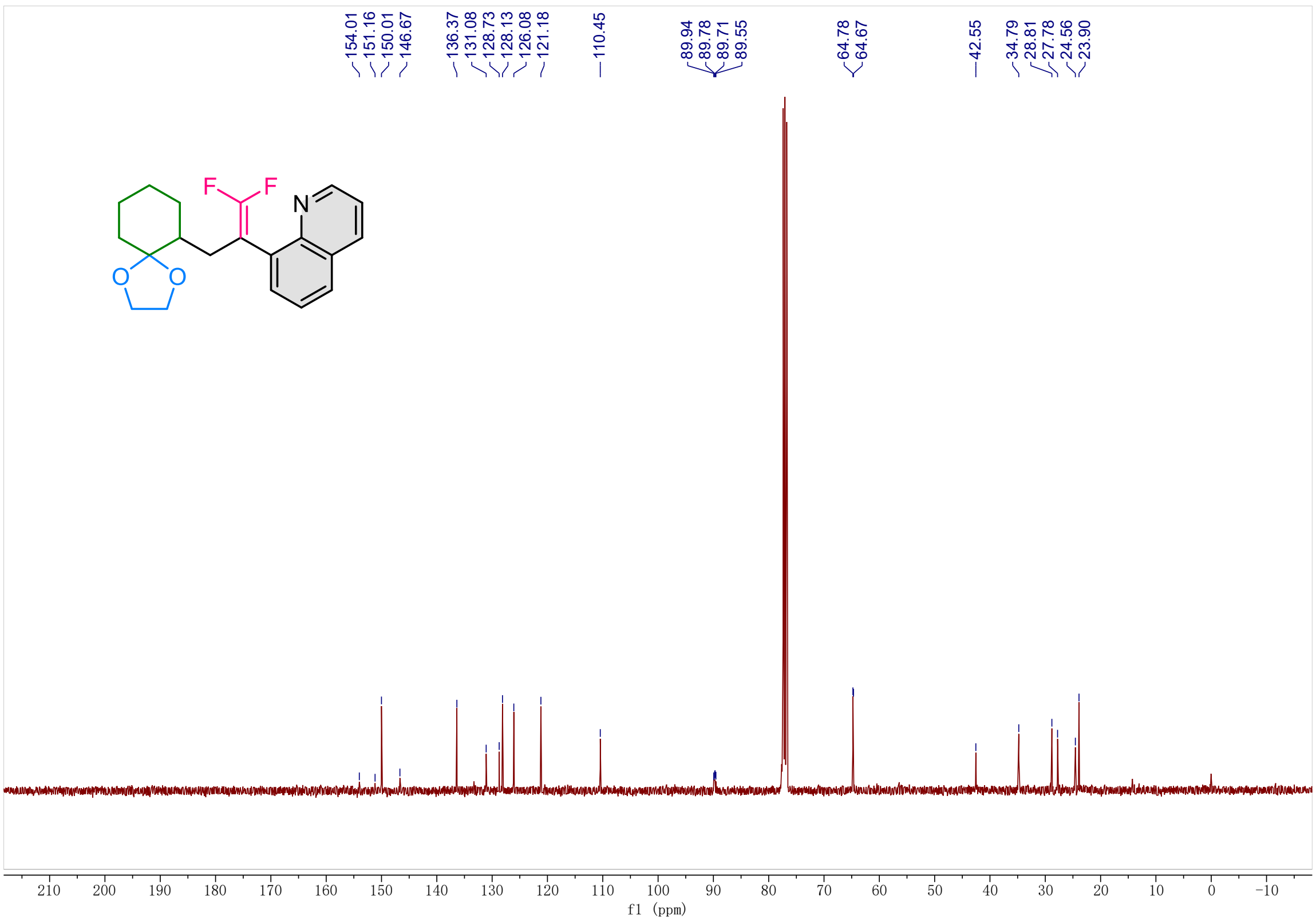

^{13}C NMR spectrum (100 MHz, Chloroform-*d*) of **9m**

8-(1,1-Difluoro-3-(1,4-dioxaspiro[4.5]decan-6-yl)prop-1-en-2-yl)quinoline (Scheme 2, product 9m)

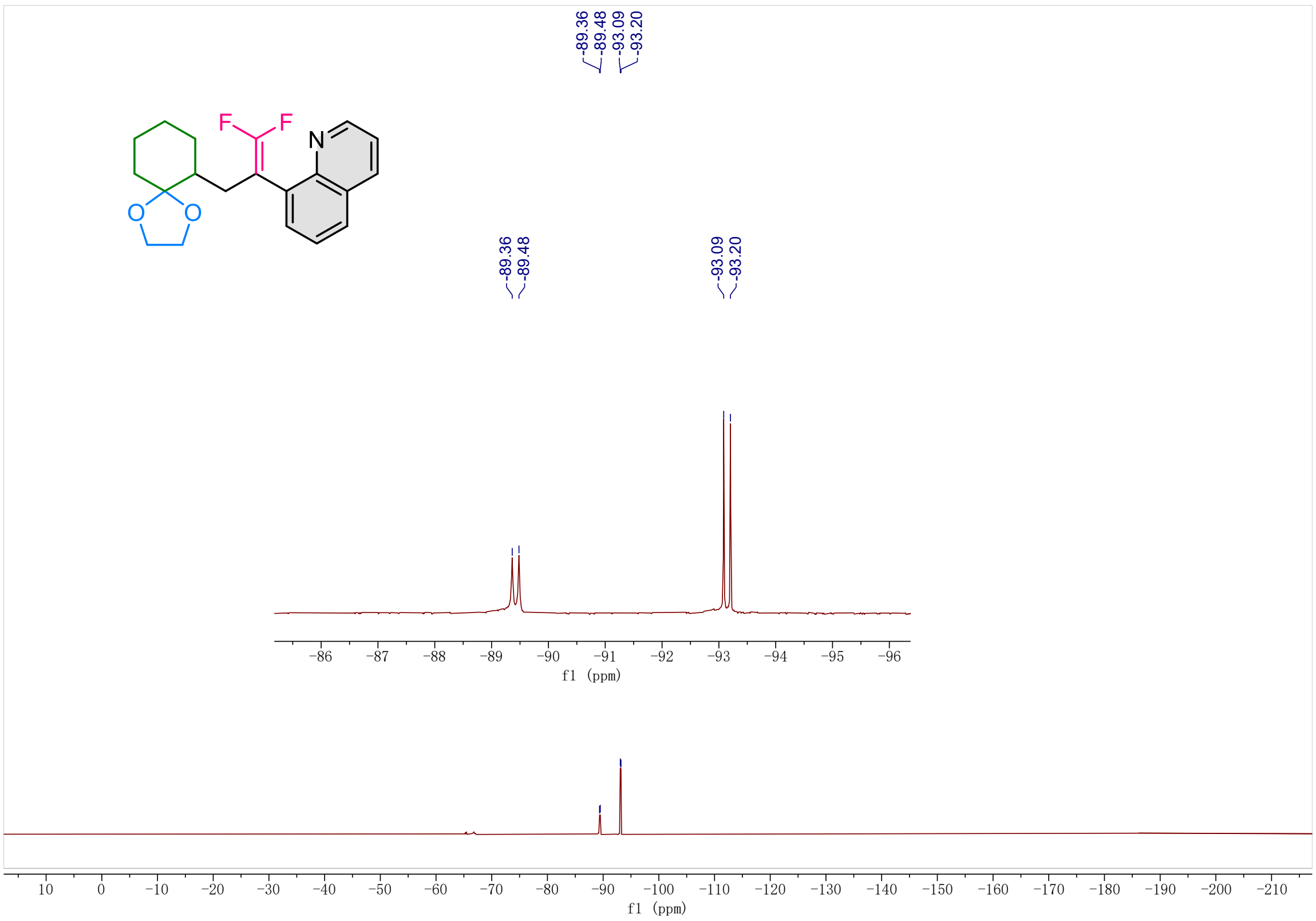

^{19}F NMR spectrum (377 MHz, Chloroform-*d*) of **9m**

6-(2-([1,1'-Biphenyl]-4-yl)-3,3-difluoroallyl)-1,4-dioxaspiro[4.4]nonane (Scheme 2, product 9n)

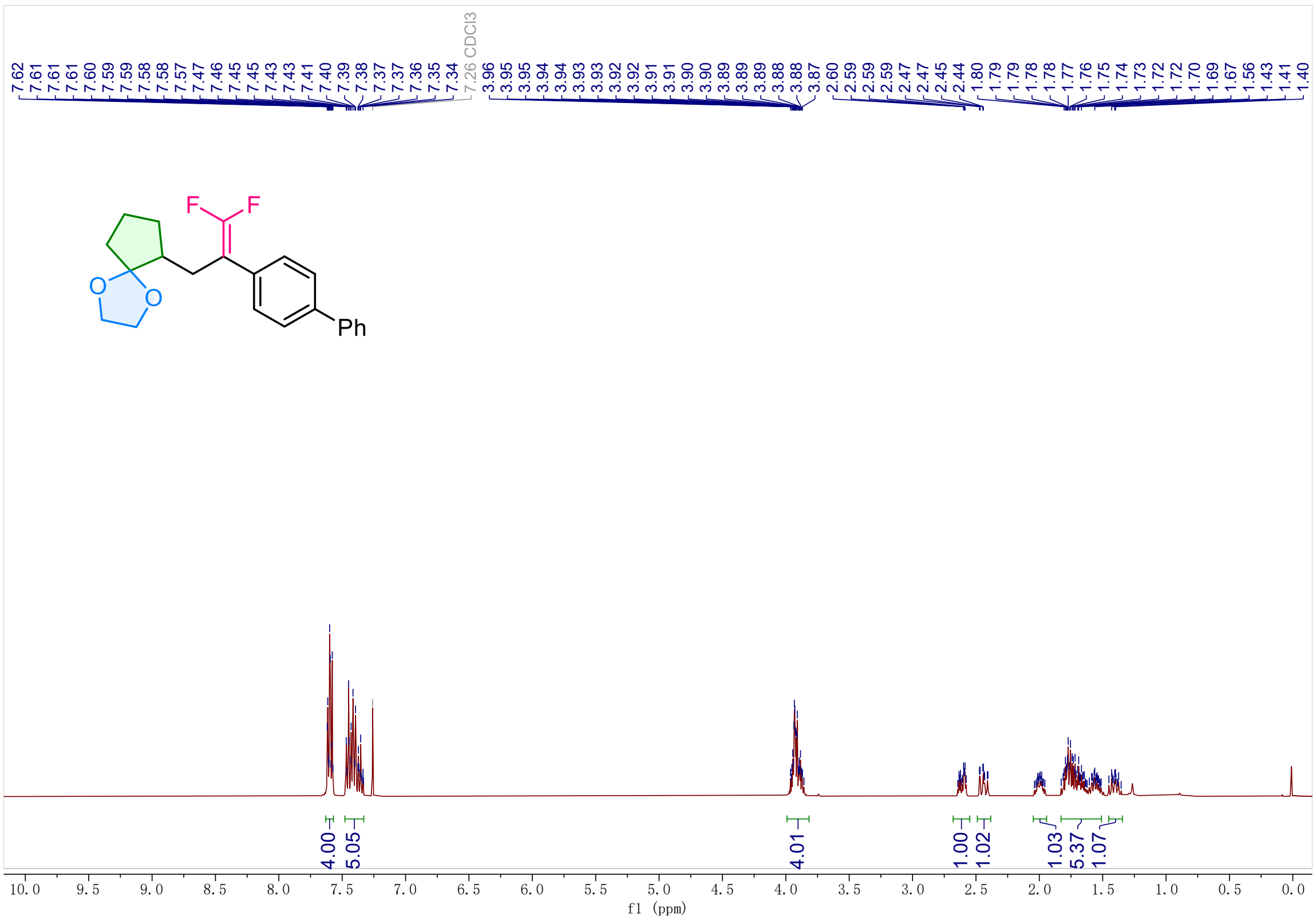

^{1}H NMR spectrum (400 MHz, Chloroform-*d*) of **9n**

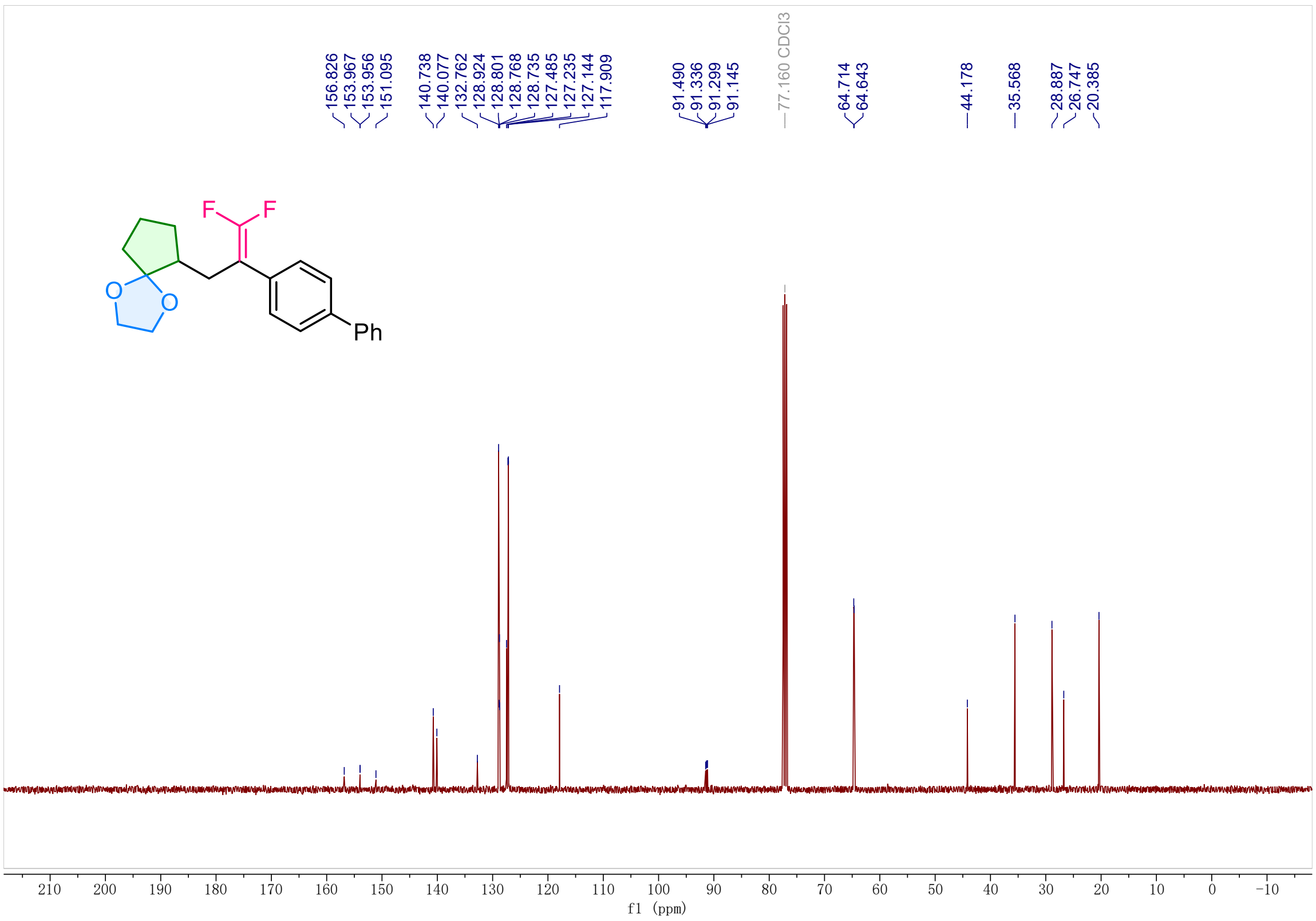

^{13}C NMR spectrum (100 MHz, Chloroform-*d*) of **9n**

6-(2-([1,1'-Biphenyl]-4-yl)-3,3-difluoroallyl)-1,4-dioxaspiro[4.4]nonane (Scheme 2, product 9n)

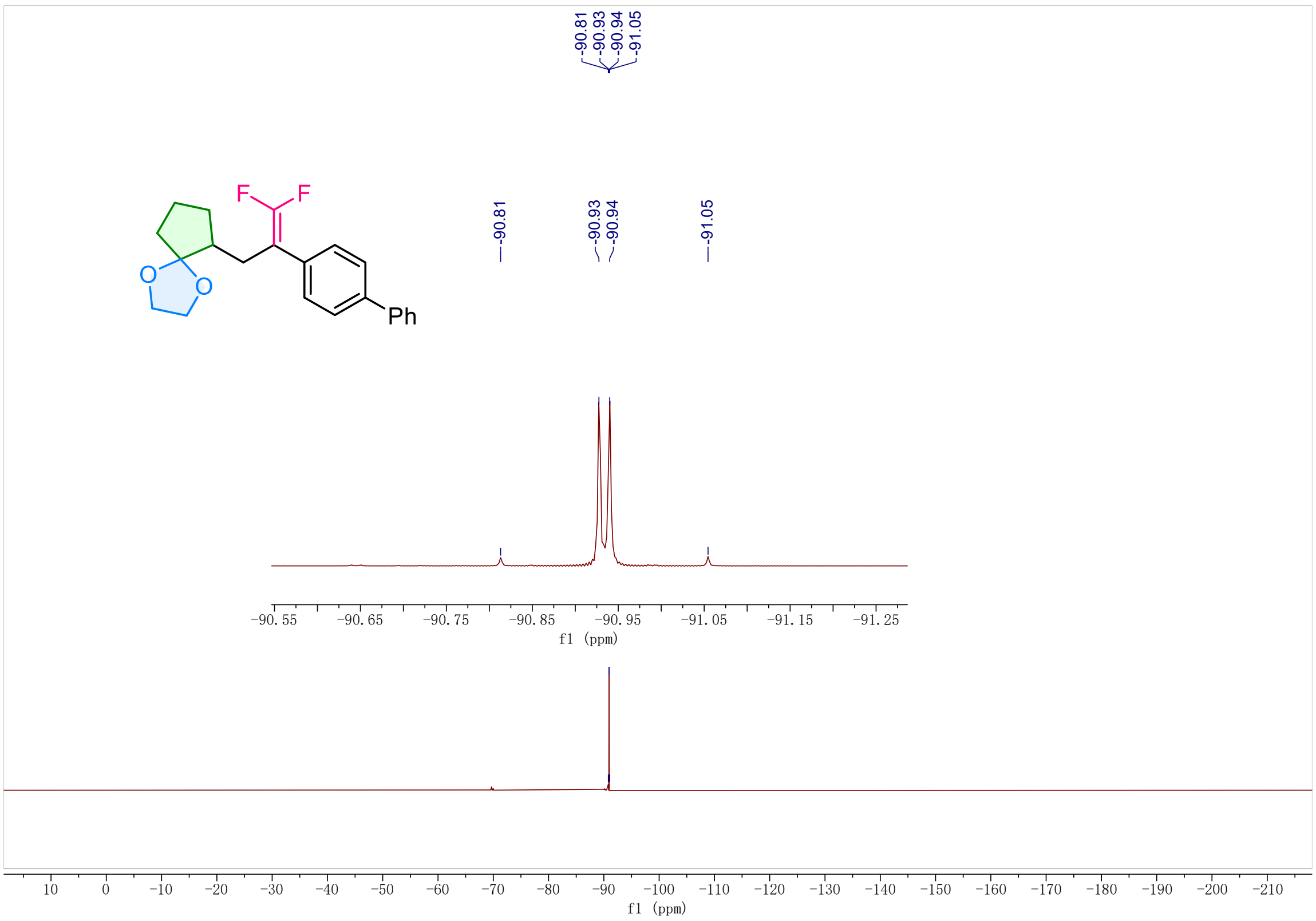

^{19}F NMR spectrum (377 MHz, Chloroform-*d*) of **9n**

6-(2-([1,1'-Biphenyl]-4-yl)-3,3-difluoroallyl)-1,4,9,12-tetraoxadispiro[4.2.4⁸.2⁵]tetradecane (Scheme 2, product 9o)

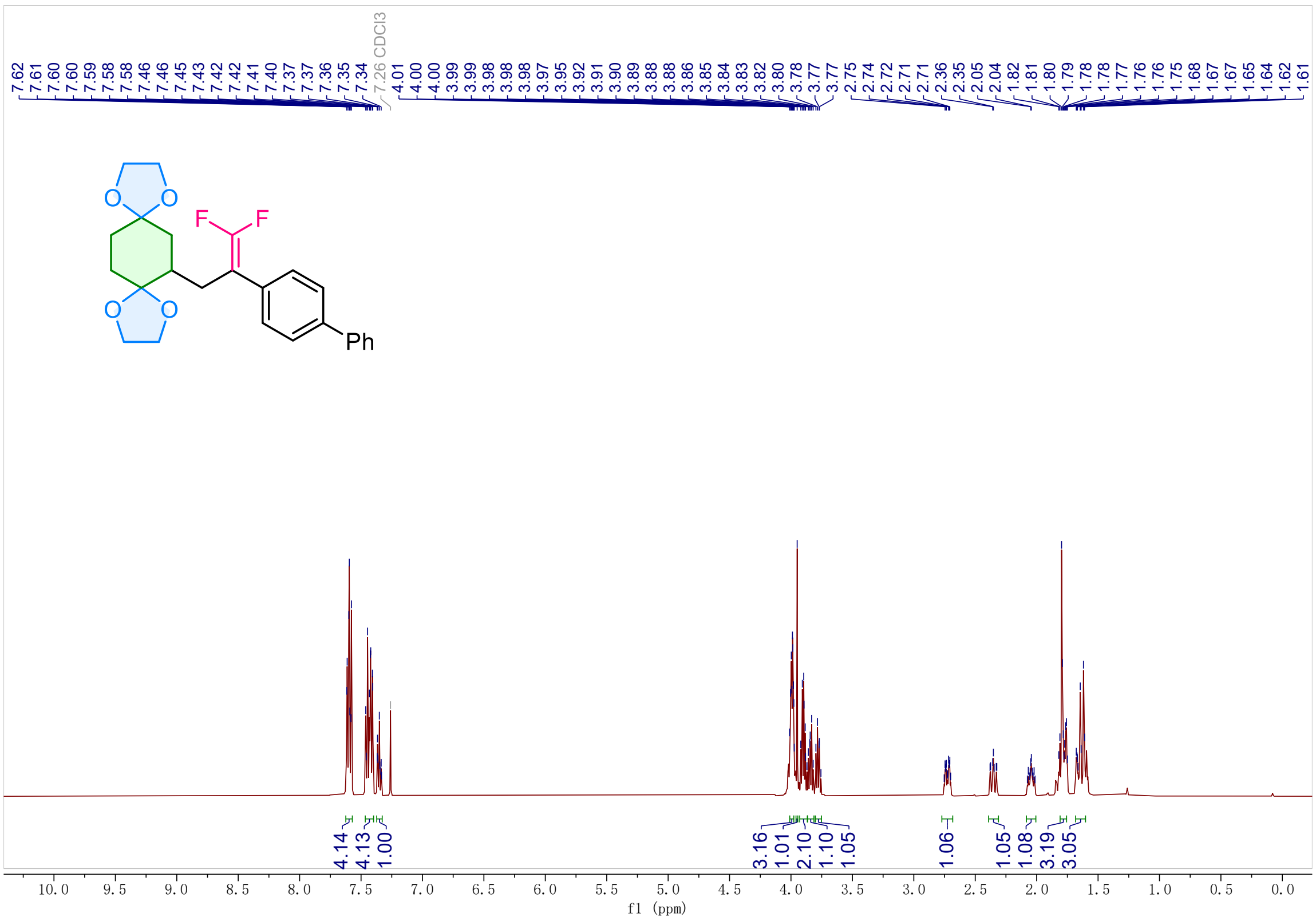

^{1}H NMR spectrum (500 MHz, Chloroform-*d*) of **9o**

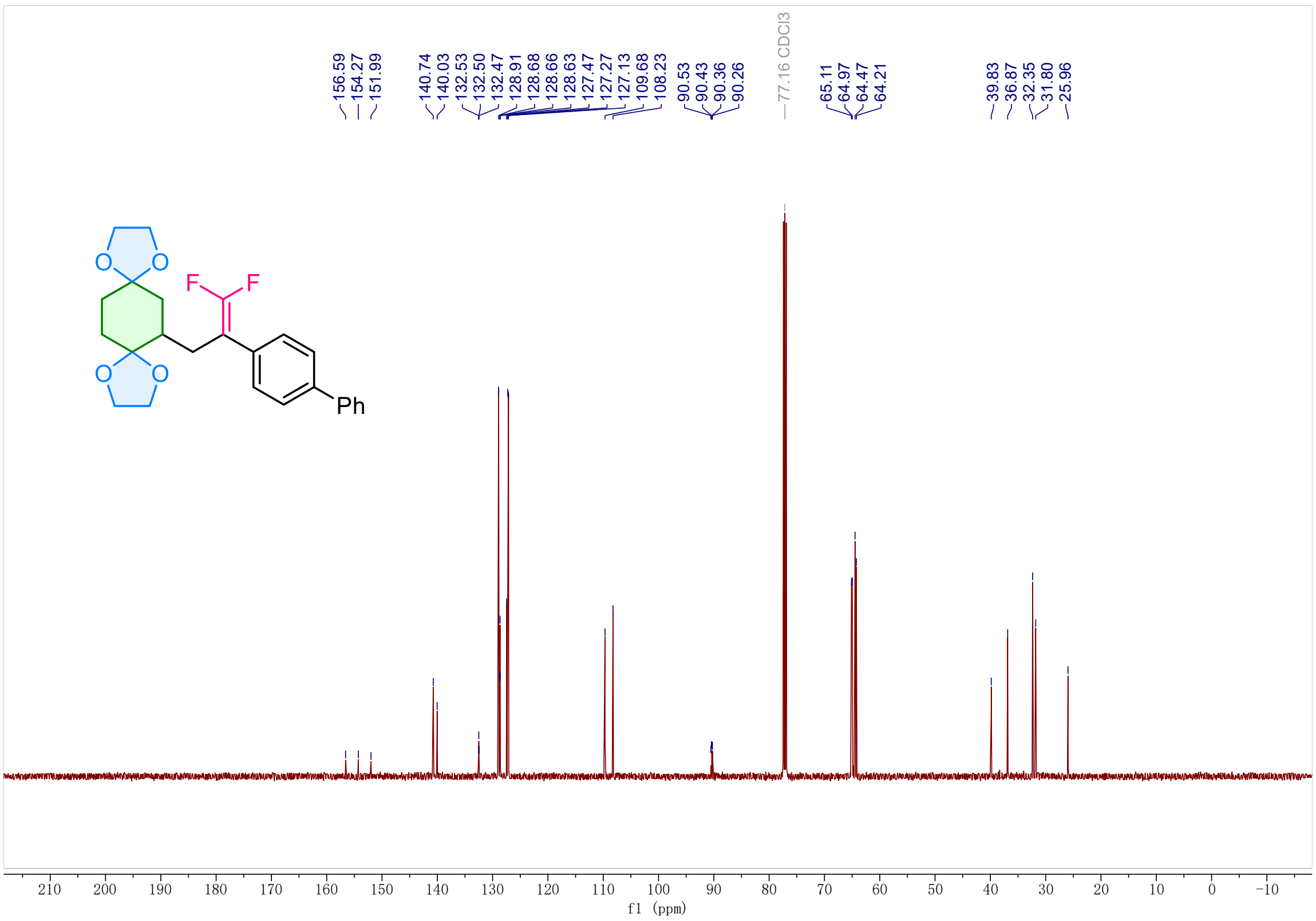

^{13}C NMR spectrum (125 MHz, Chloroform-*d*) of **9o**

6-(2-([1,1'-Biphenyl]-4-yl)-3,3-difluoroallyl)-1,4,9,12-tetraoxadispiro[4.2.4^{8}.2^{5}]tetradecane (Scheme 2, product 9o)

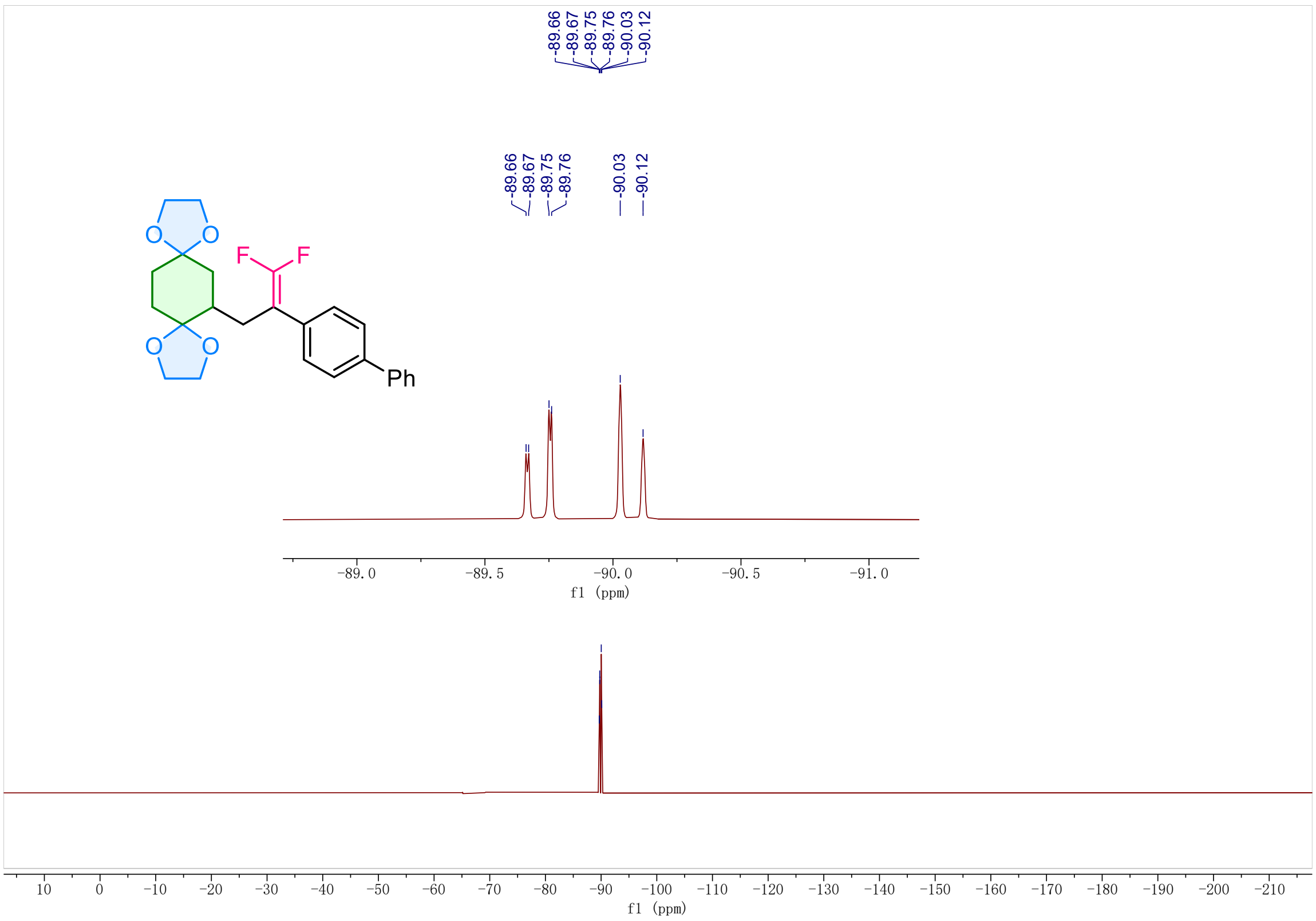

^{19}F NMR spectrum (471 MHz, Chloroform-*d*) of **9o**

6-(2-([1,1'-Biphenyl]-4-yl)-3,3-difluoroallyl)-1,4,8-trioxaspiro[4.5]decane (Scheme 2, product 9p)

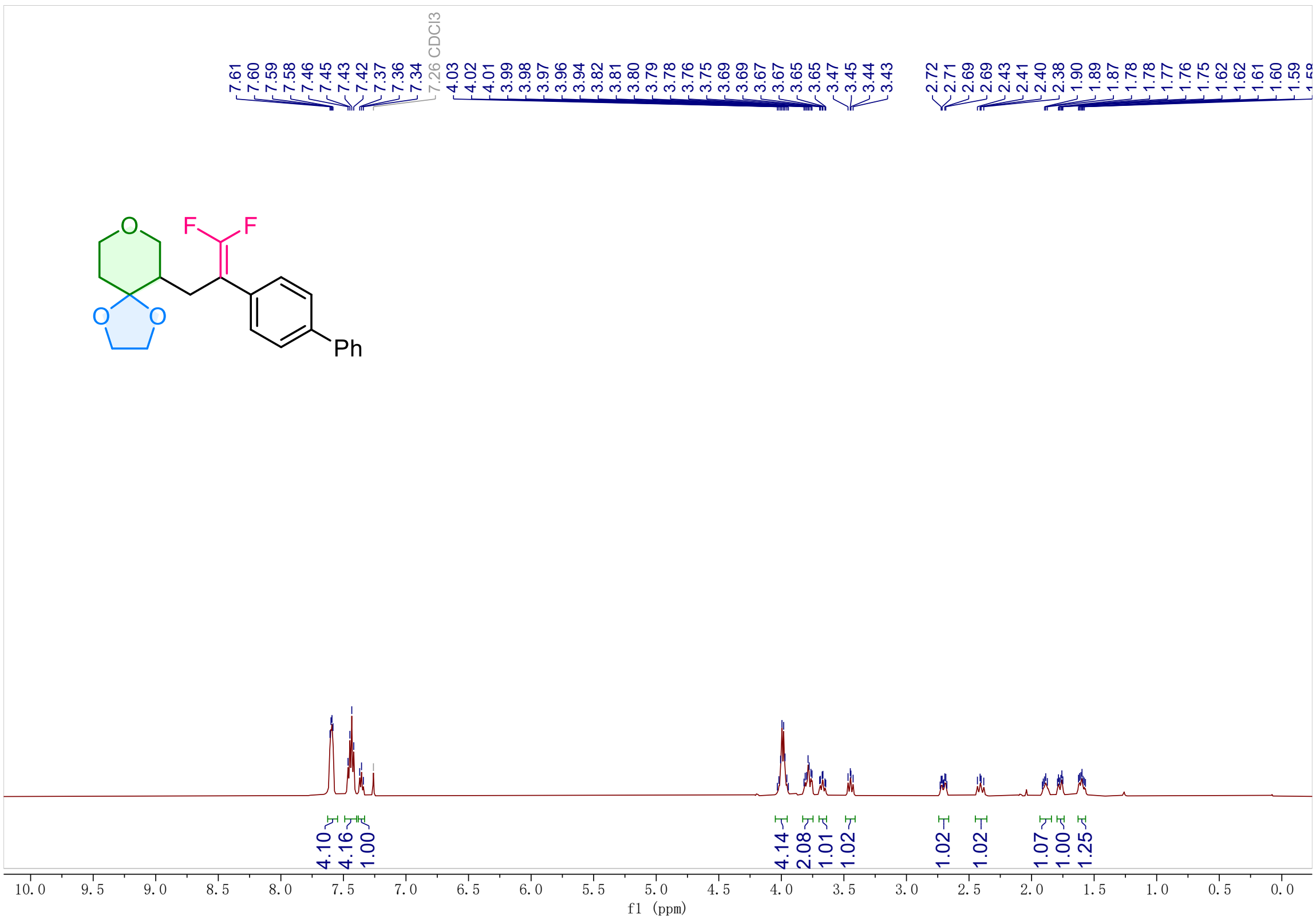

^{1}H NMR spectrum (500 MHz, Chloroform-*d*) of **9p**

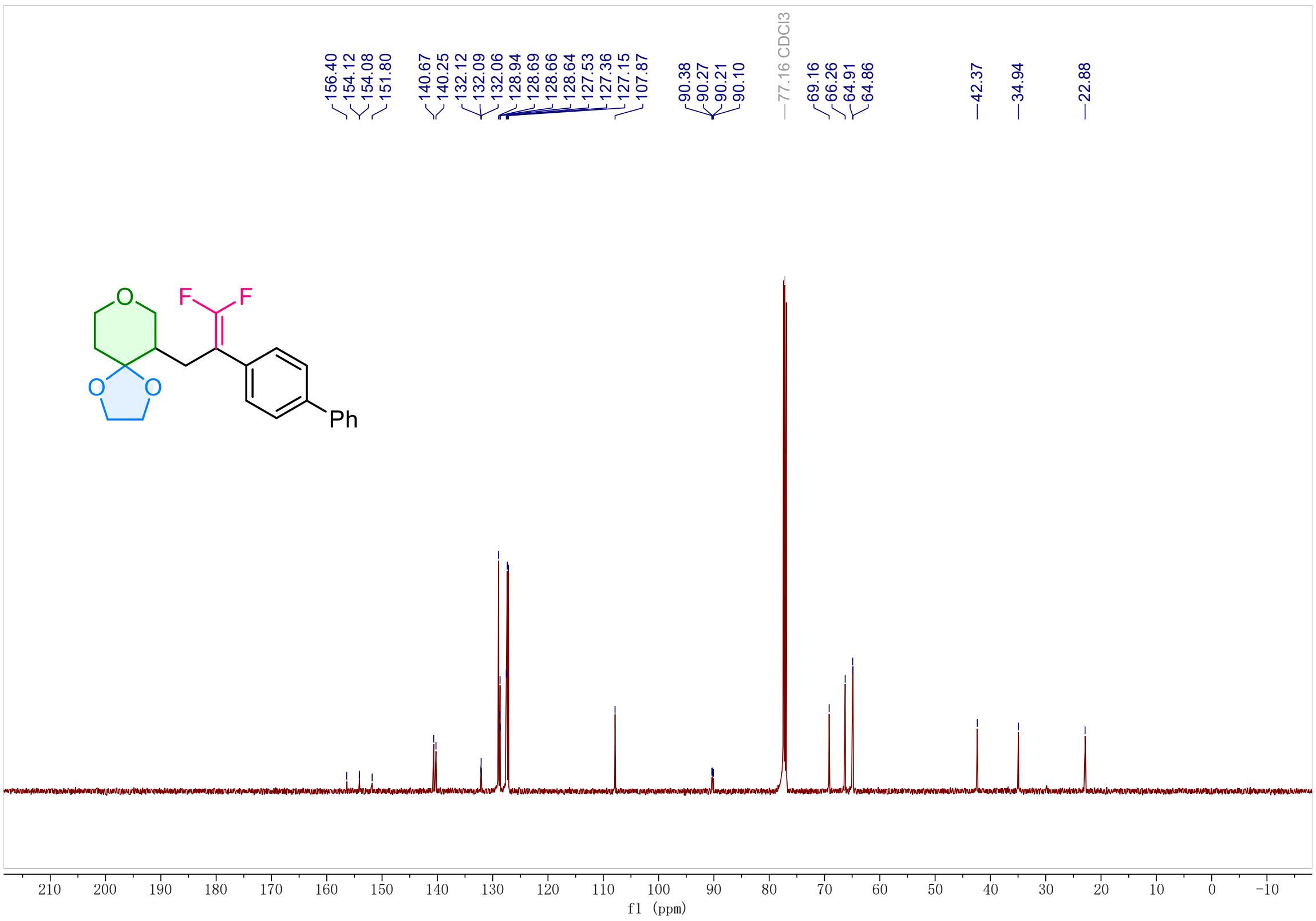

^{13}C NMR spectrum (125 MHz, Chloroform-*d*) of **9p**

6-(2-([1,1'-Biphenyl]-4-yl)-3,3-difluoroallyl)-1,4,8-trioxaspiro[4.5]decane (Scheme 2, product 9p)

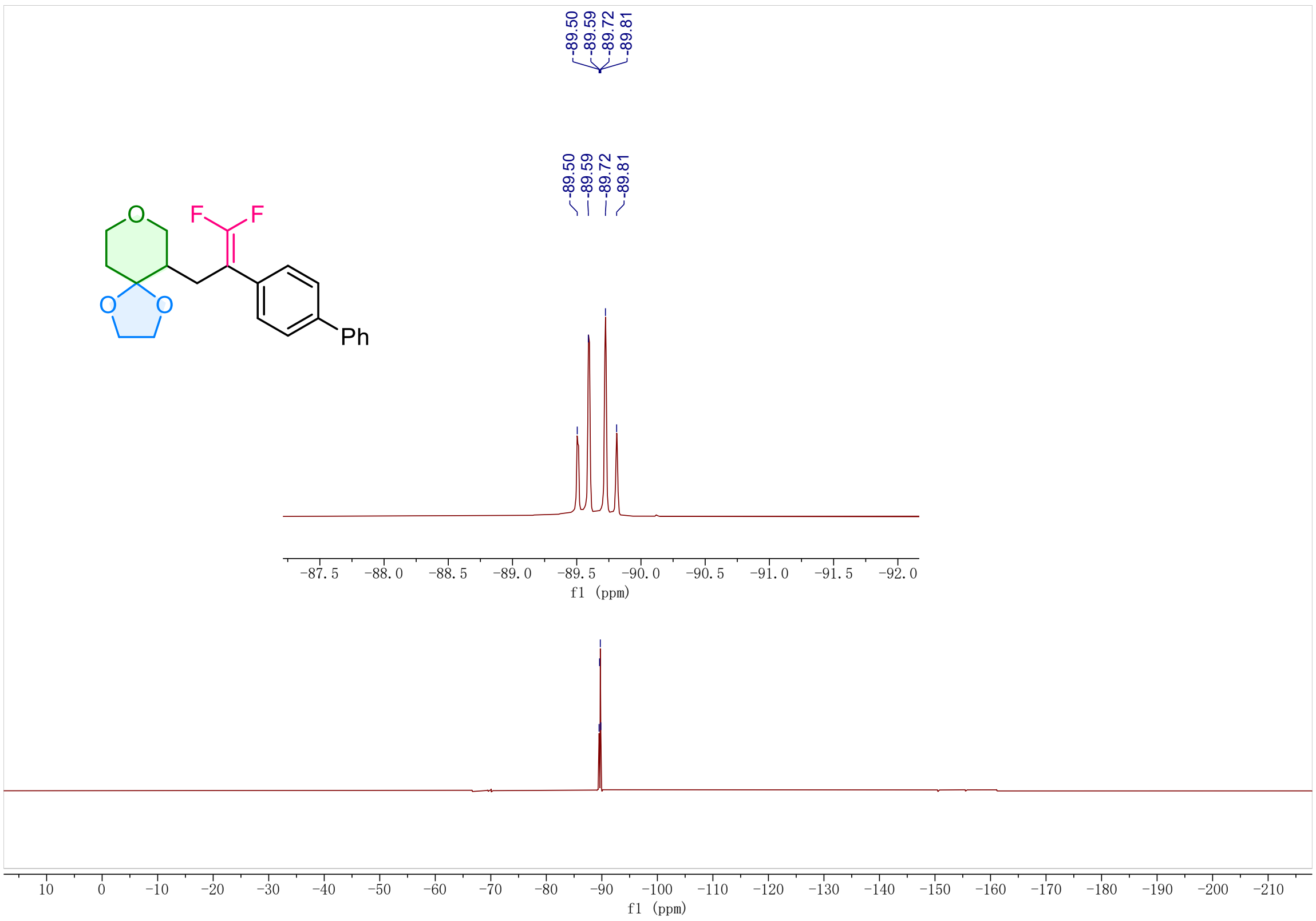

^{19}F NMR spectrum (471 MHz, Chloroform-*d*) of **9p**

Methyl 4-(1,1-difluoro-3-(1,4,8-trioxaspiro[4.5]decan-6-yl)prop-1-en-2-yl)benzoate (Scheme 2, product 9q)

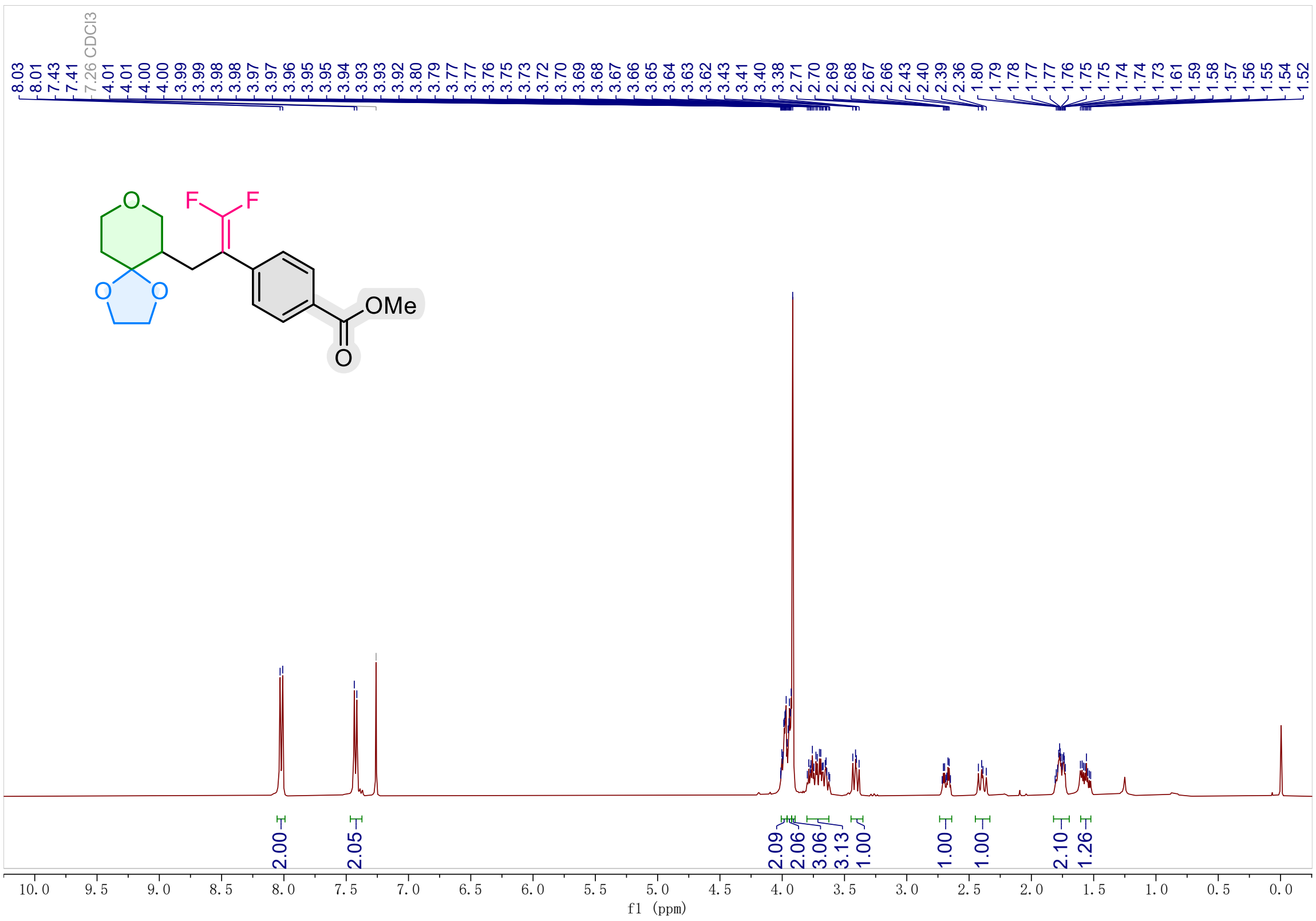

^{1}H NMR spectrum (400 MHz, Chloroform-*d*) of **9q**

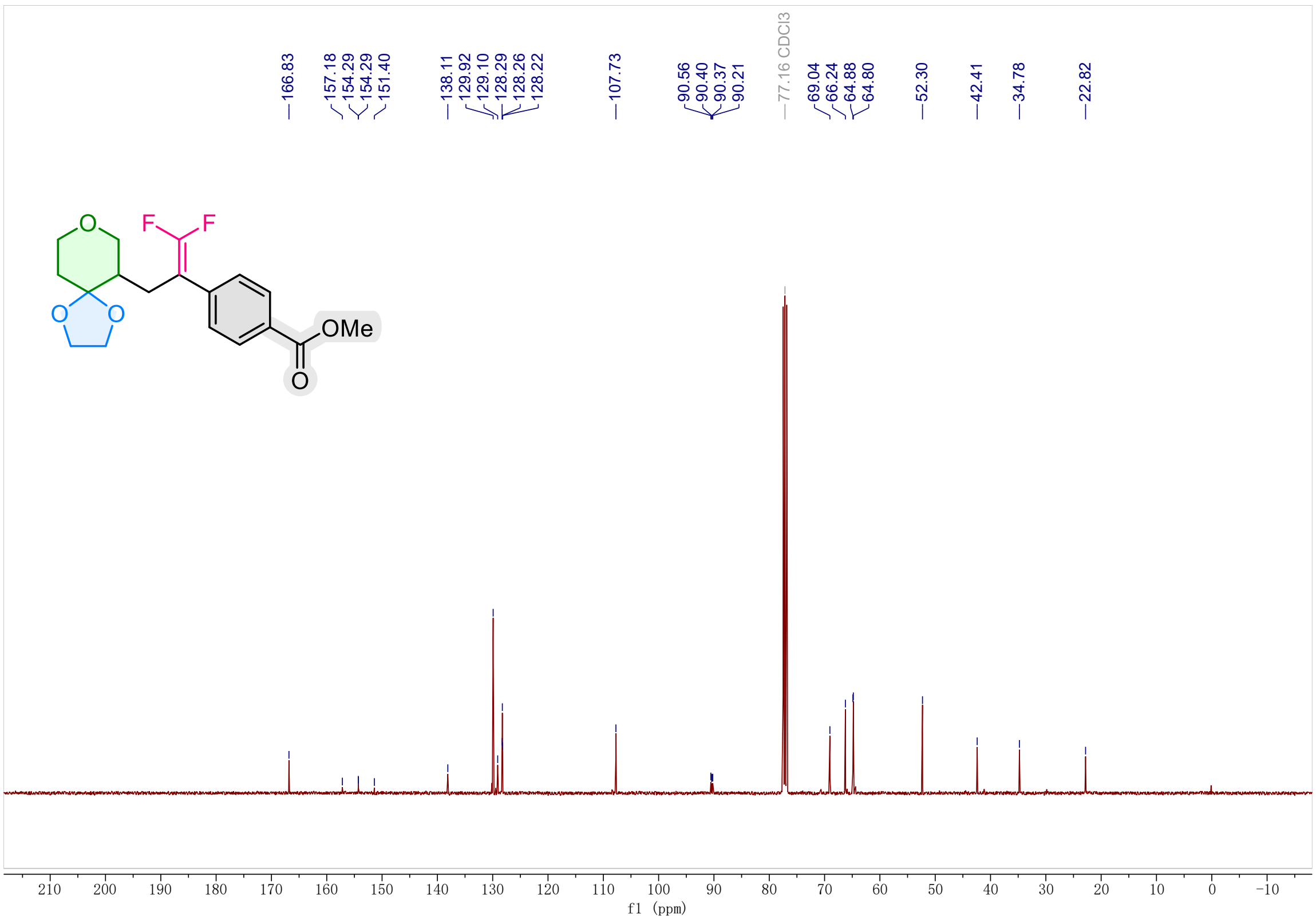

^{13}C NMR spectrum (100 MHz, Chloroform-*d*) of **9q**

Methyl 4-(1,1-difluoro-3-(1,4,8-trioxaspiro[4.5]decan-6-yl)prop-1-en-2-yl)benzoate (Scheme 2, product 9q)

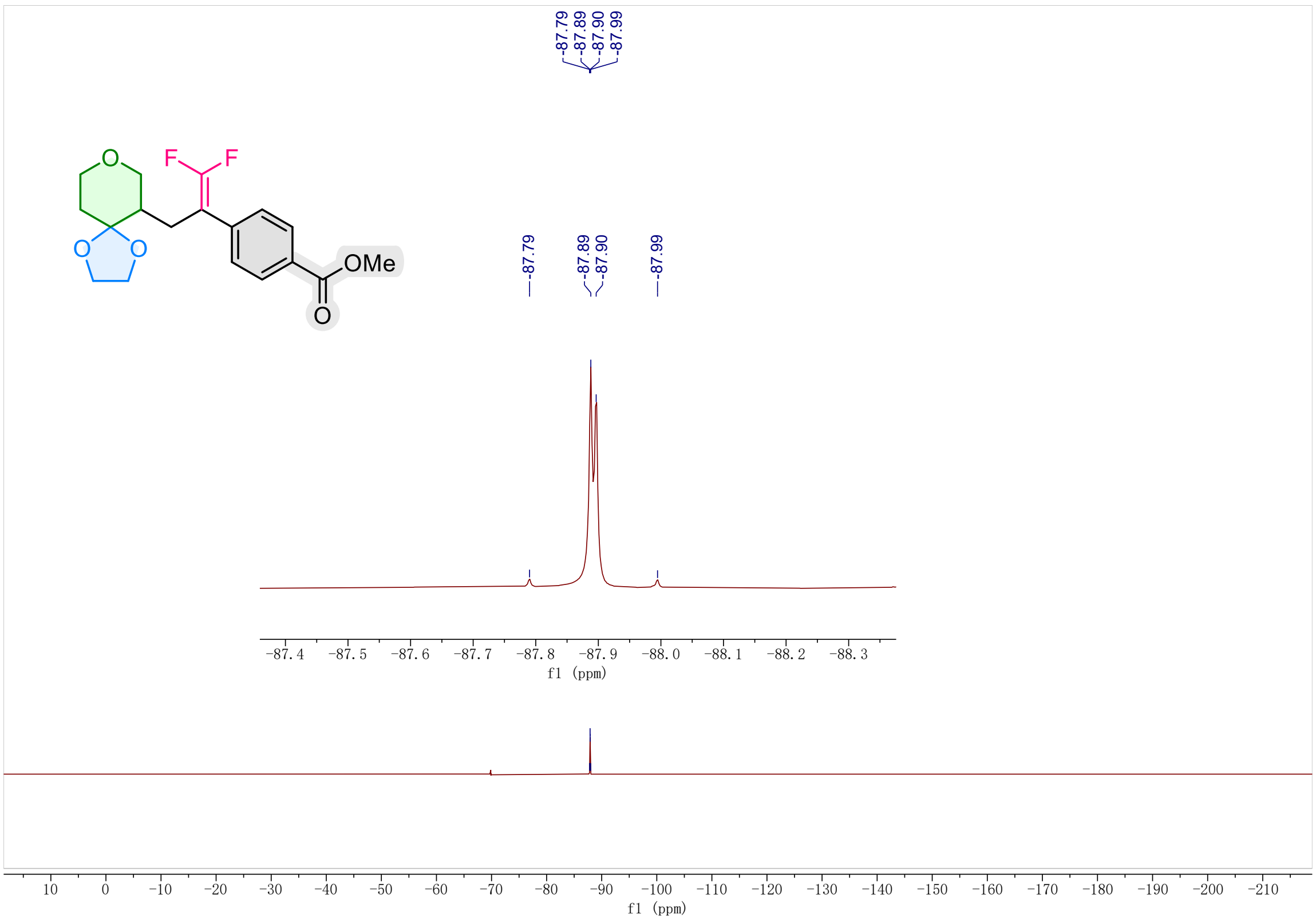

^{19}F NMR spectrum (377 MHz, Chloroform-*d*) of **9q**

2-(5-([1,1'-Biphenyl]-4-yl)-6,6-difluorohex-5-en-3-yl)-2-propyl-1,3-dioxolane (Scheme 2, product 9r)

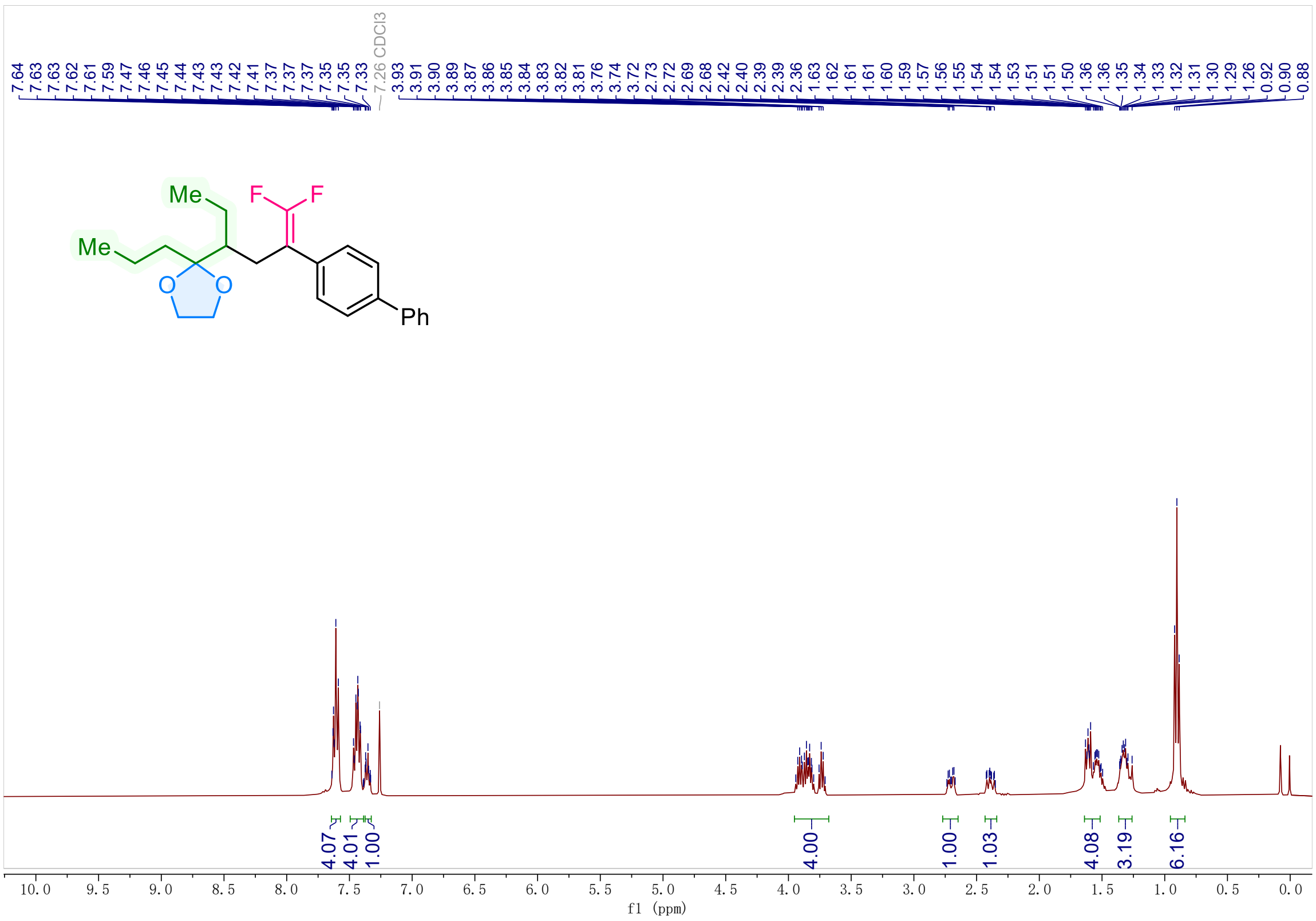

^{1}H NMR spectrum (400 MHz, Chloroform-*d*) of **9r**

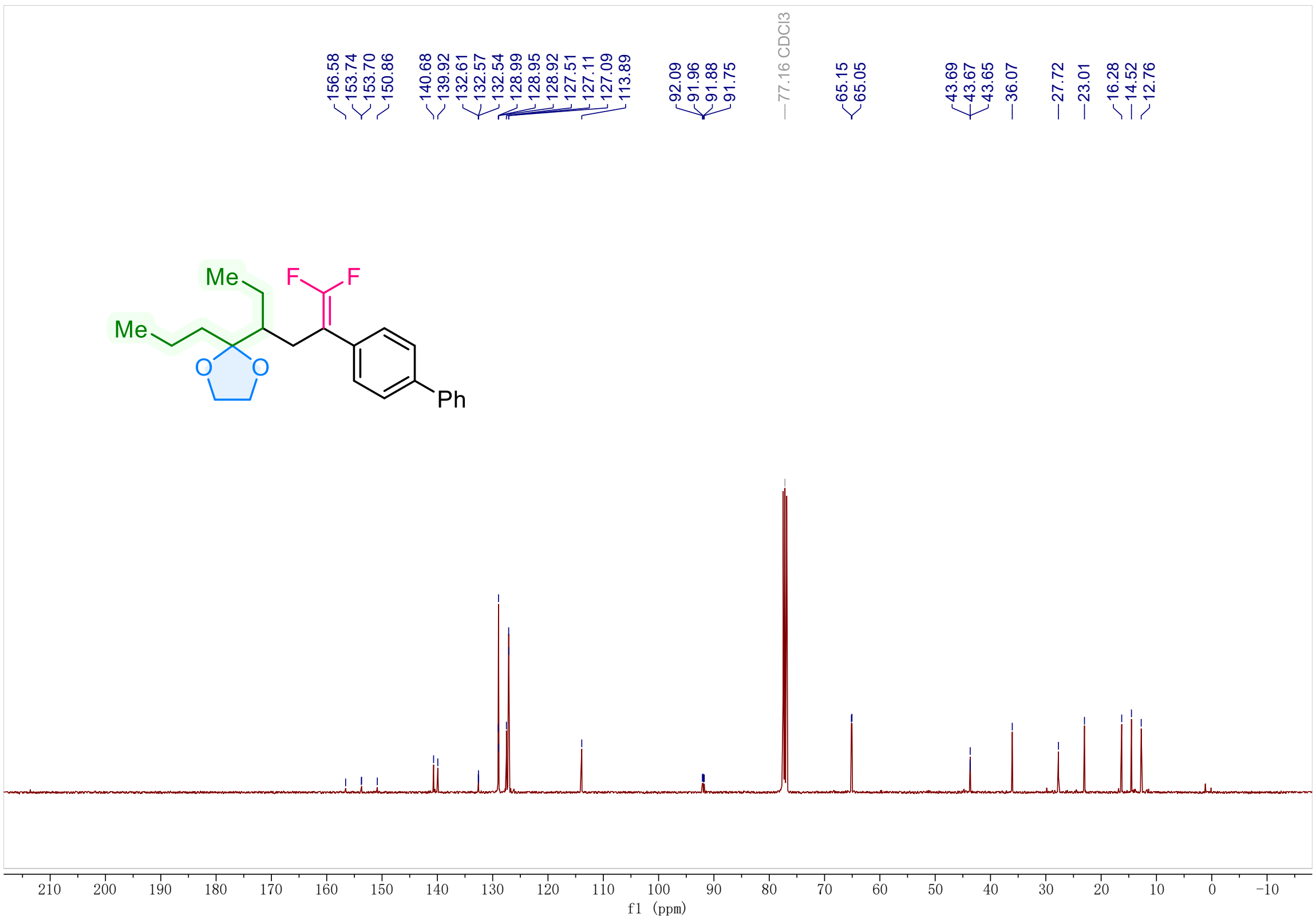

^{13}C NMR spectrum (100 MHz, Chloroform-*d*) of **9r**

2-(5-([1,1'-Biphenyl]-4-yl)-6,6-difluorohex-5-en-3-yl)-2-propyl-1,3-dioxolane (Scheme 2, product 9r)

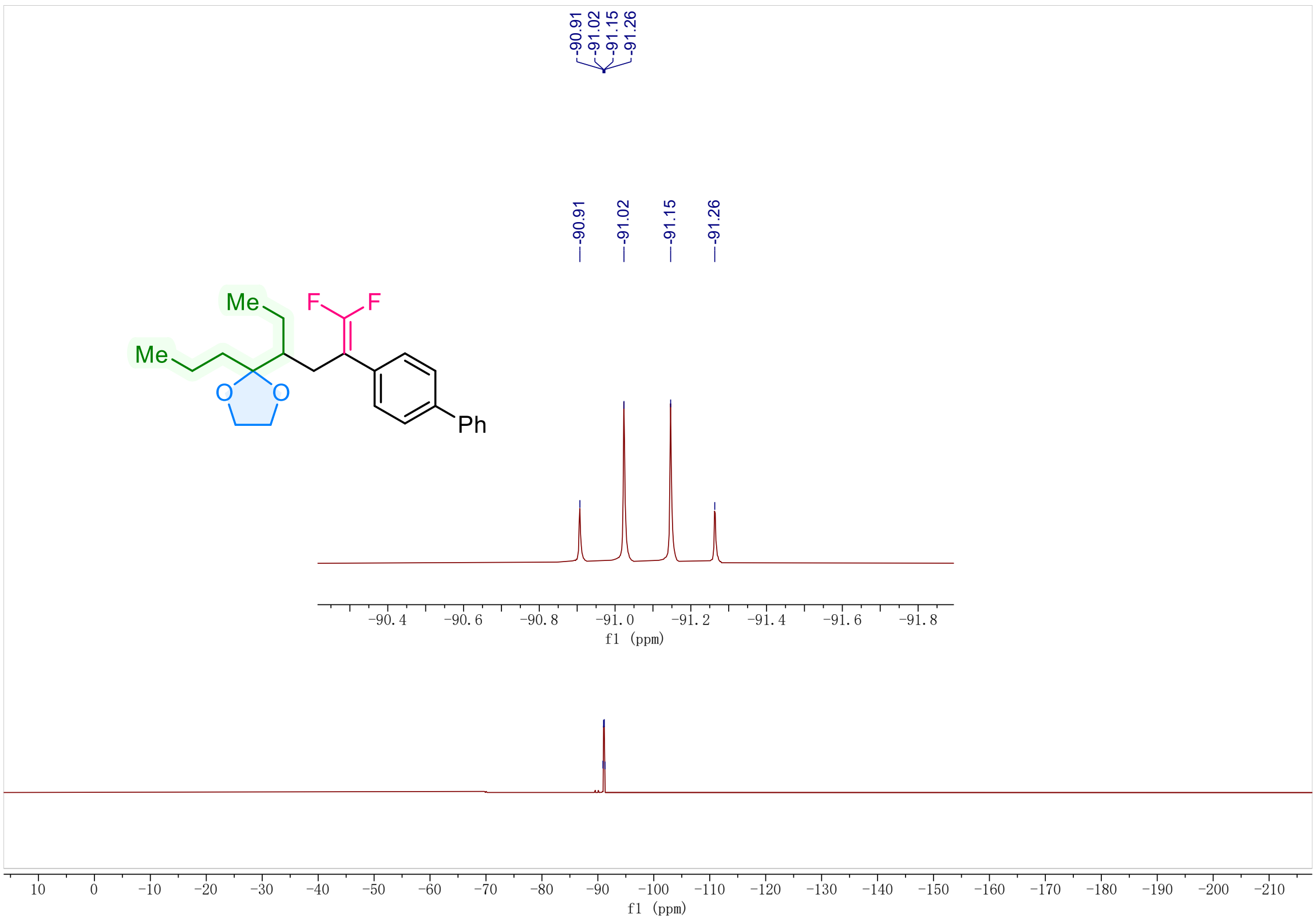

^{19}F NMR spectrum (377 MHz, Chloroform-*d*) of **9r**

6-(2-([1,1'-Biphenyl]-4-yl)-3,3-difluoroallyl)-6-methyl-1,4-dioxaspiro[4.5]decane dioxolane (Scheme 2, product 9s)

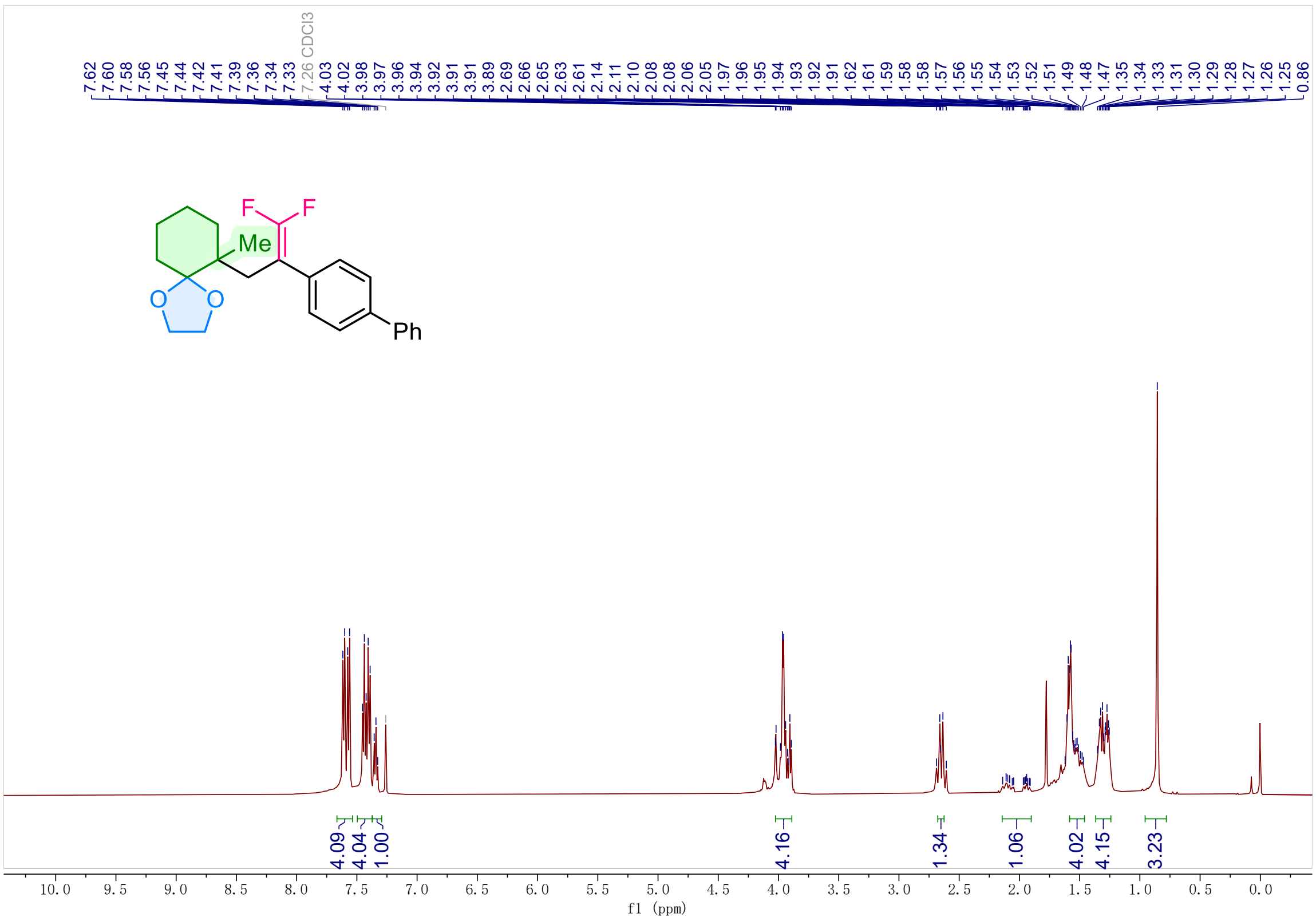

^{1}H NMR spectrum (500 MHz, Chloroform-*d*) of **9s**

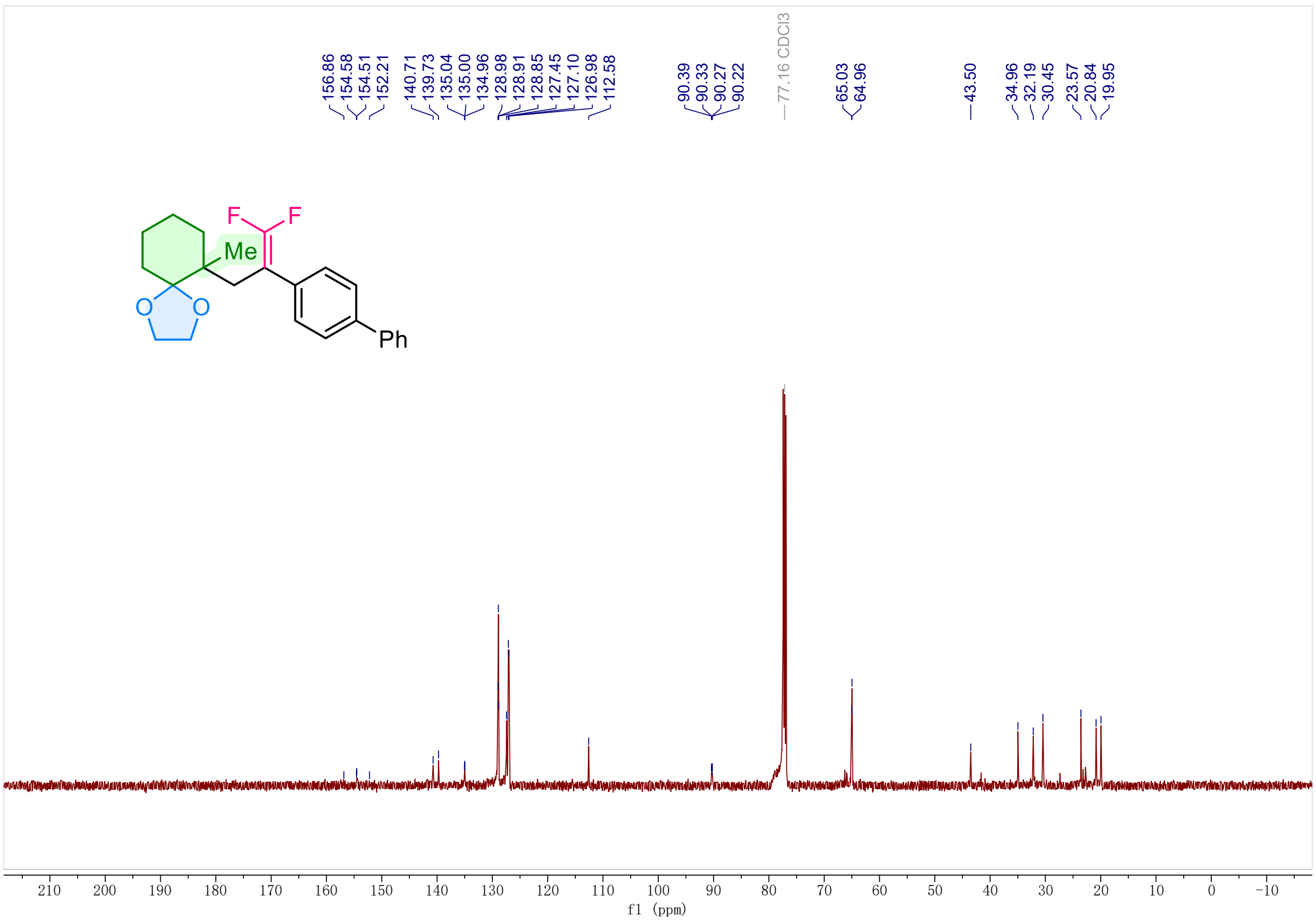

^{13}C NMR spectrum (125 MHz, Chloroform-*d*) of **9s**

6-(2-([1,1'-Biphenyl]-4-yl)-3,3-difluoroallyl)-6-methyl-1,4-dioxaspiro[4.5]decane dioxolane (Scheme 2, product 9s)

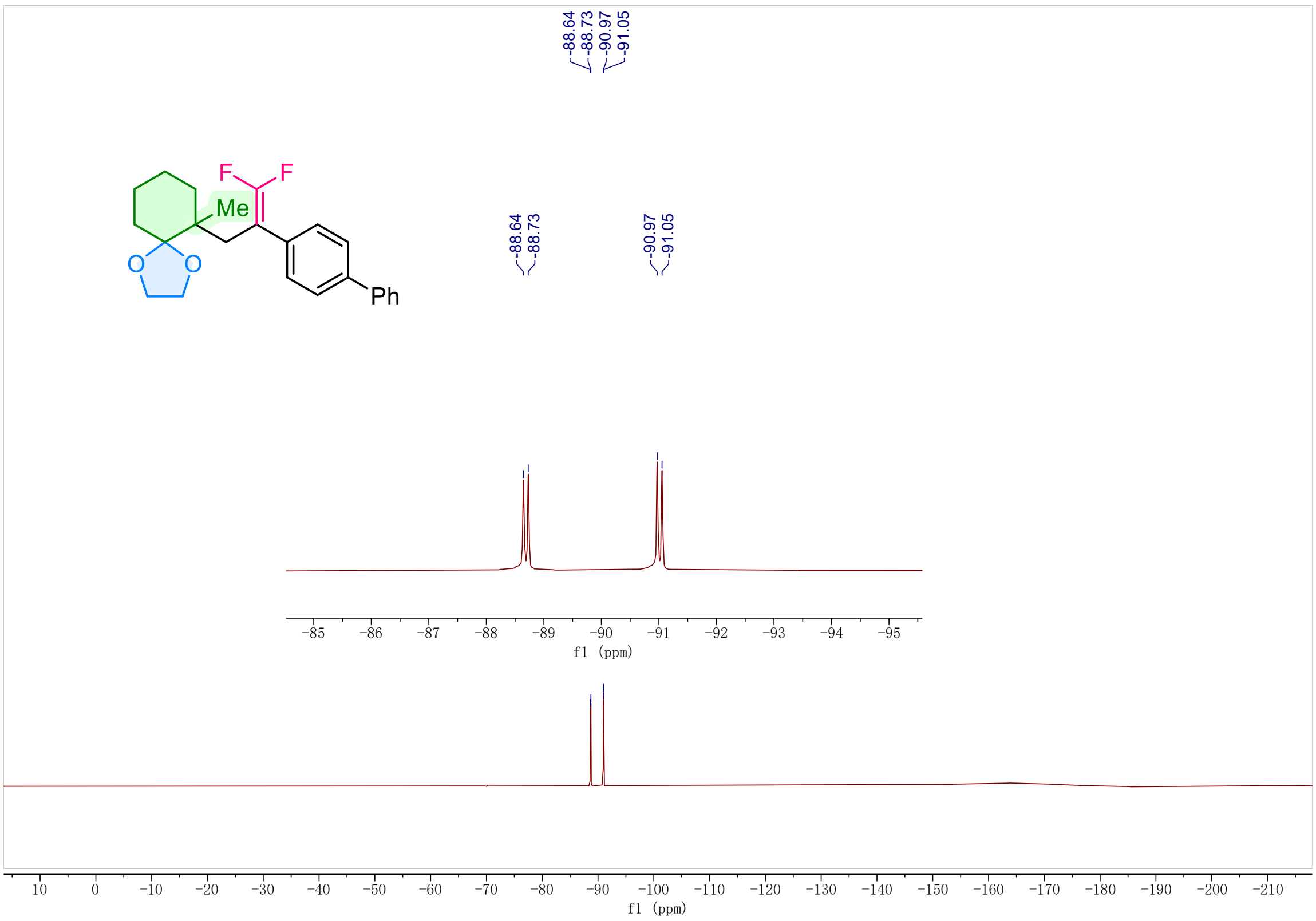

^{19}F NMR spectrum (471 MHz, Chloroform-*d*) of **9s**

2-(3-([1,1'-Biphenyl]-4-yl)-3-(2,2-difluorovinyl)-1-hexylpropyl)-4,4,7,7-tetramethyl-1,3-dioxepane (Scheme 2, product 10a)

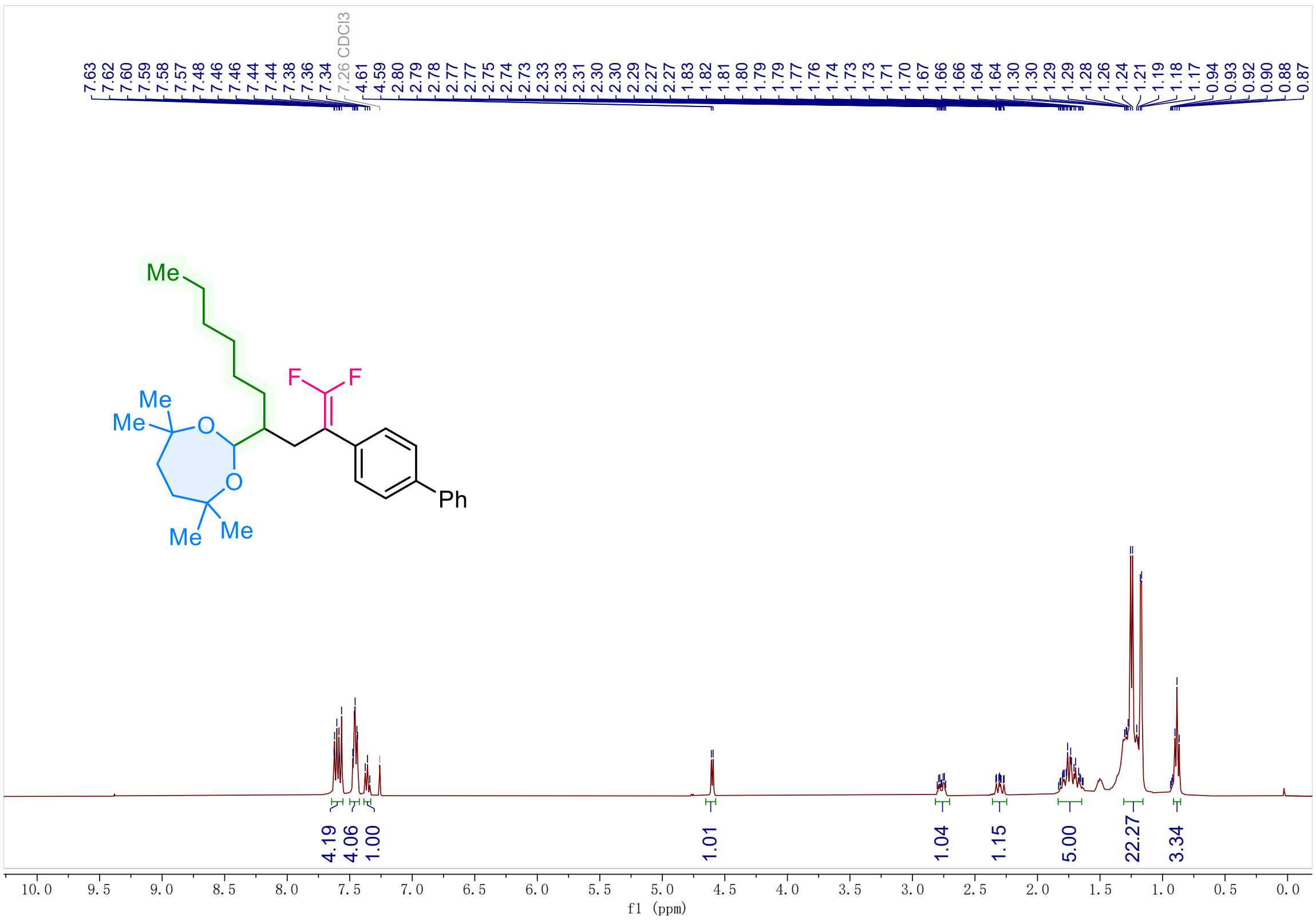

^{1}H NMR spectrum (400 MHz, Chloroform-*d*) of **10a**

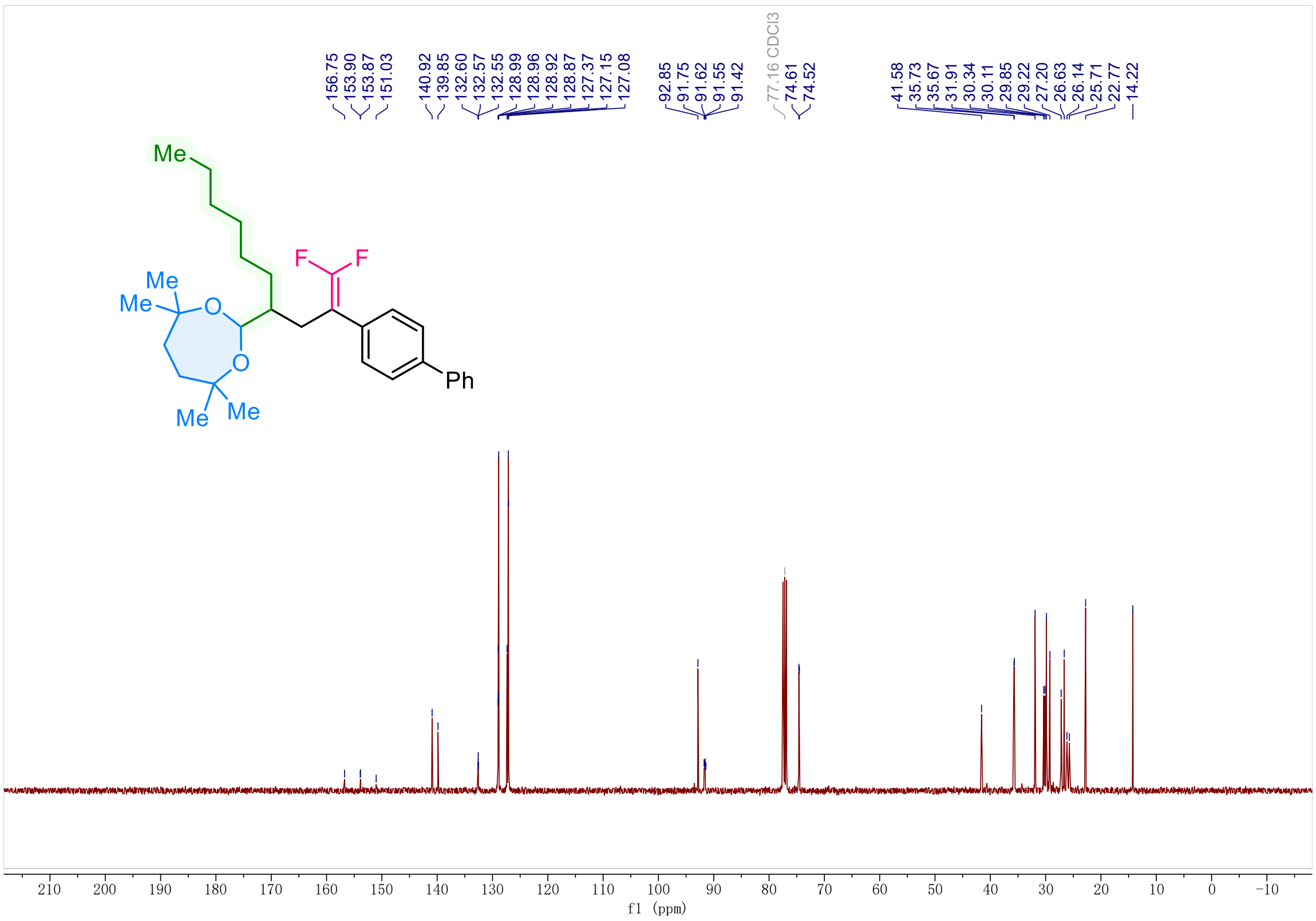

^{13}C NMR spectrum (100 MHz, Chloroform-*d*) of **10a**

2-(3-([1,1'-Biphenyl]-4-yl)-3-(2,2-difluorovinyl)-1-hexylpropyl)-4,4,7,7-tetramethyl-1,3-dioxepane (Scheme 2, product 10a)

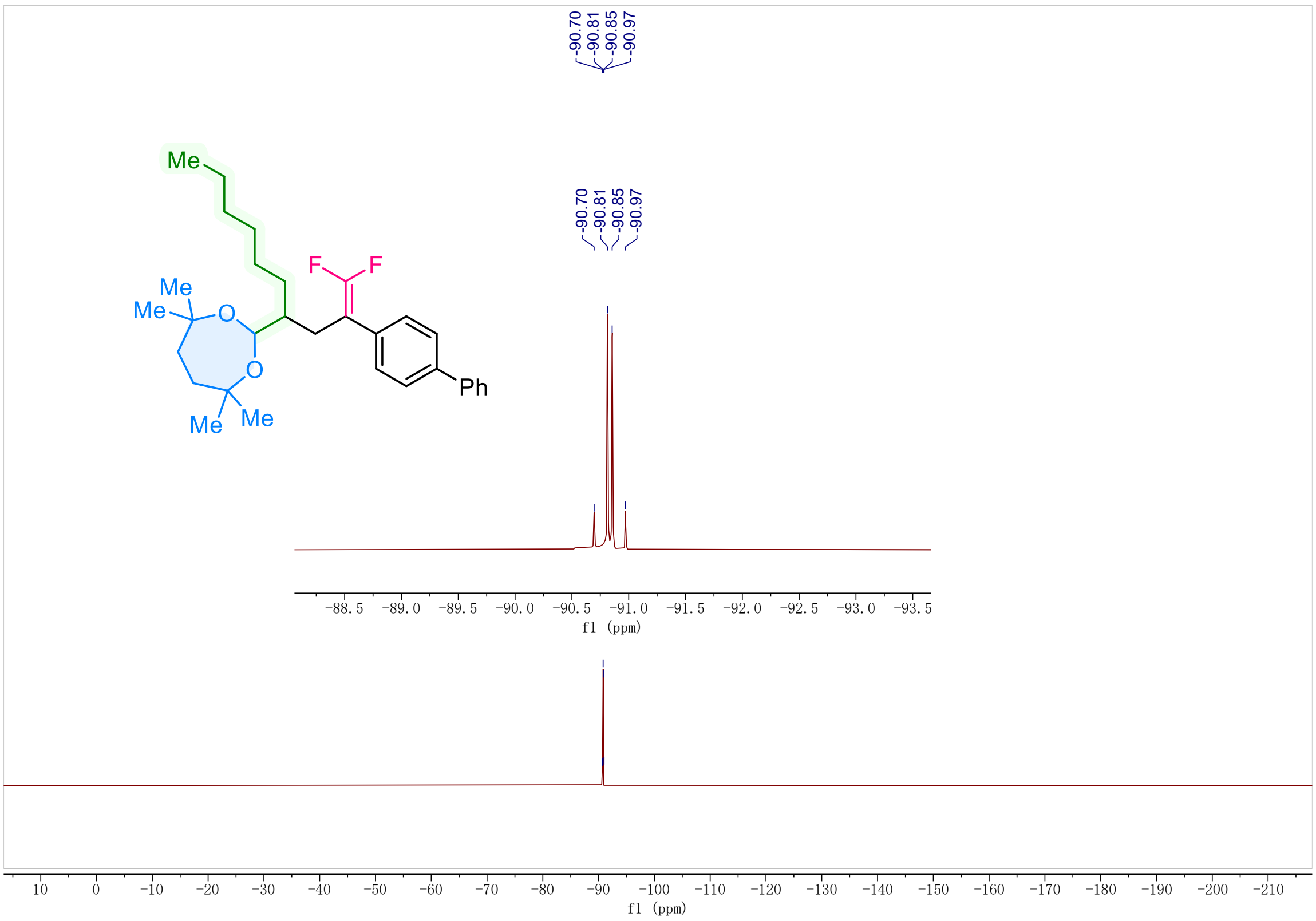

^{19}F NMR spectrum (377 MHz, Chloroform-*d*) of **10a**

2-(5-([1,1'-Biphenyl]-4-yl)-6,6-difluoro-2-methylhex-5-en-3-yl)-4,4,7,7-tetramethyl-1,3-dioxepane (Scheme 2, product 10b)

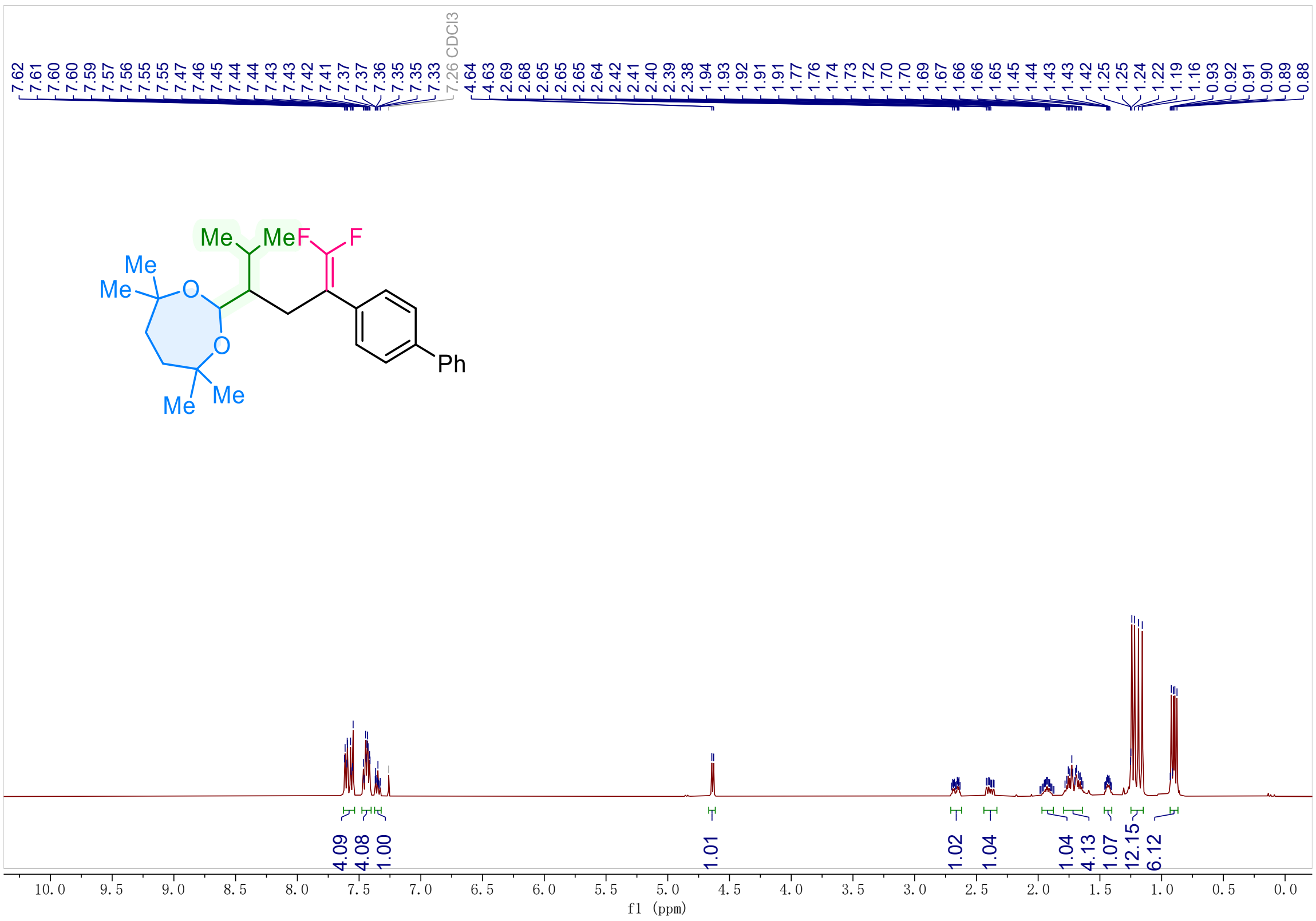

^{1}H NMR spectrum (400 MHz, Chloroform-*d*) of **10b**

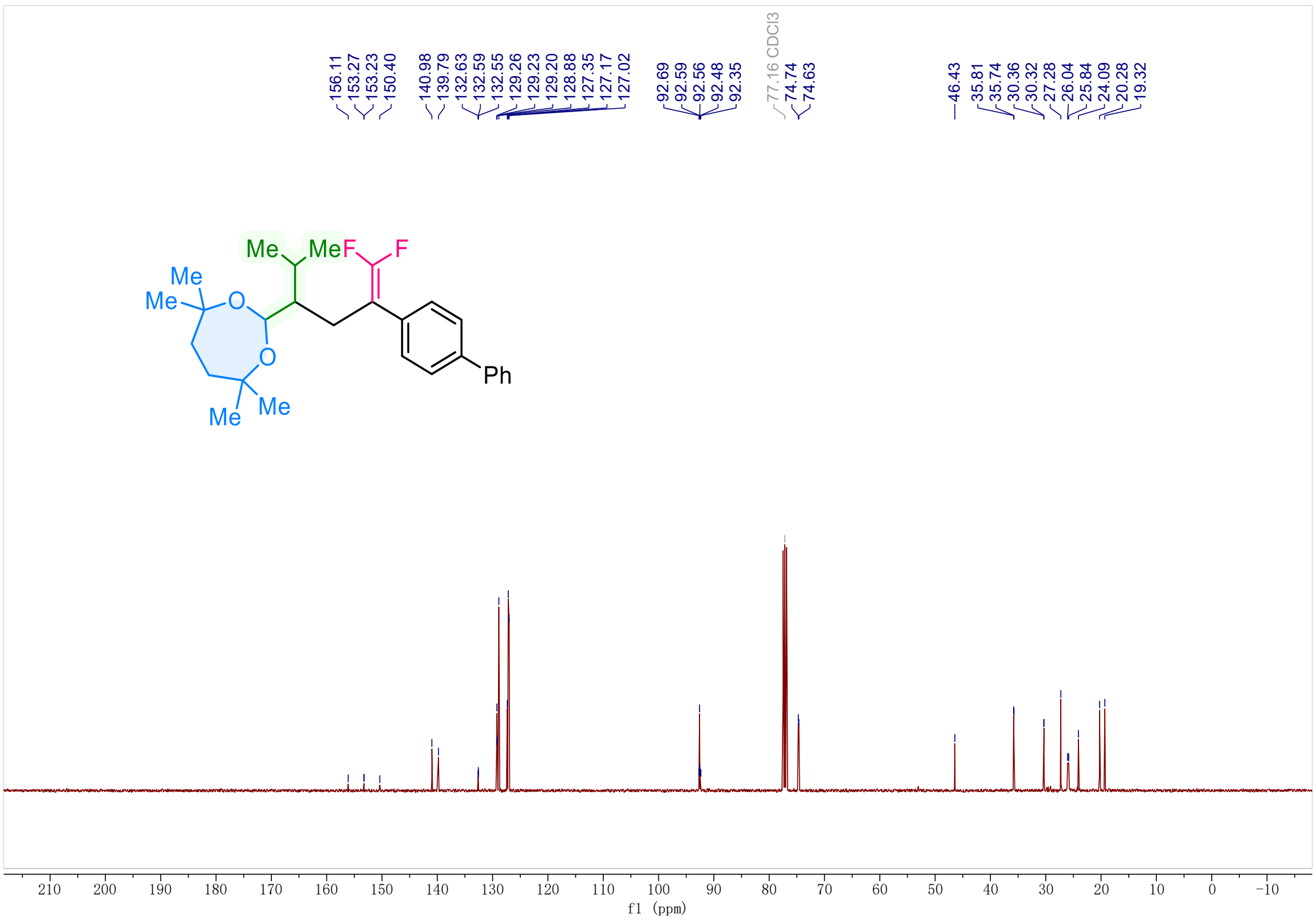

^{13}C NMR spectrum (100 MHz, Chloroform-*d*) of **10b**

2-(5-([1,1'-Biphenyl]-4-yl)-6,6-difluoro-2-methylhex-5-en-3-yl)-4,4,7,7-tetramethyl-1,3-dioxepane (Scheme 2, product 10b)

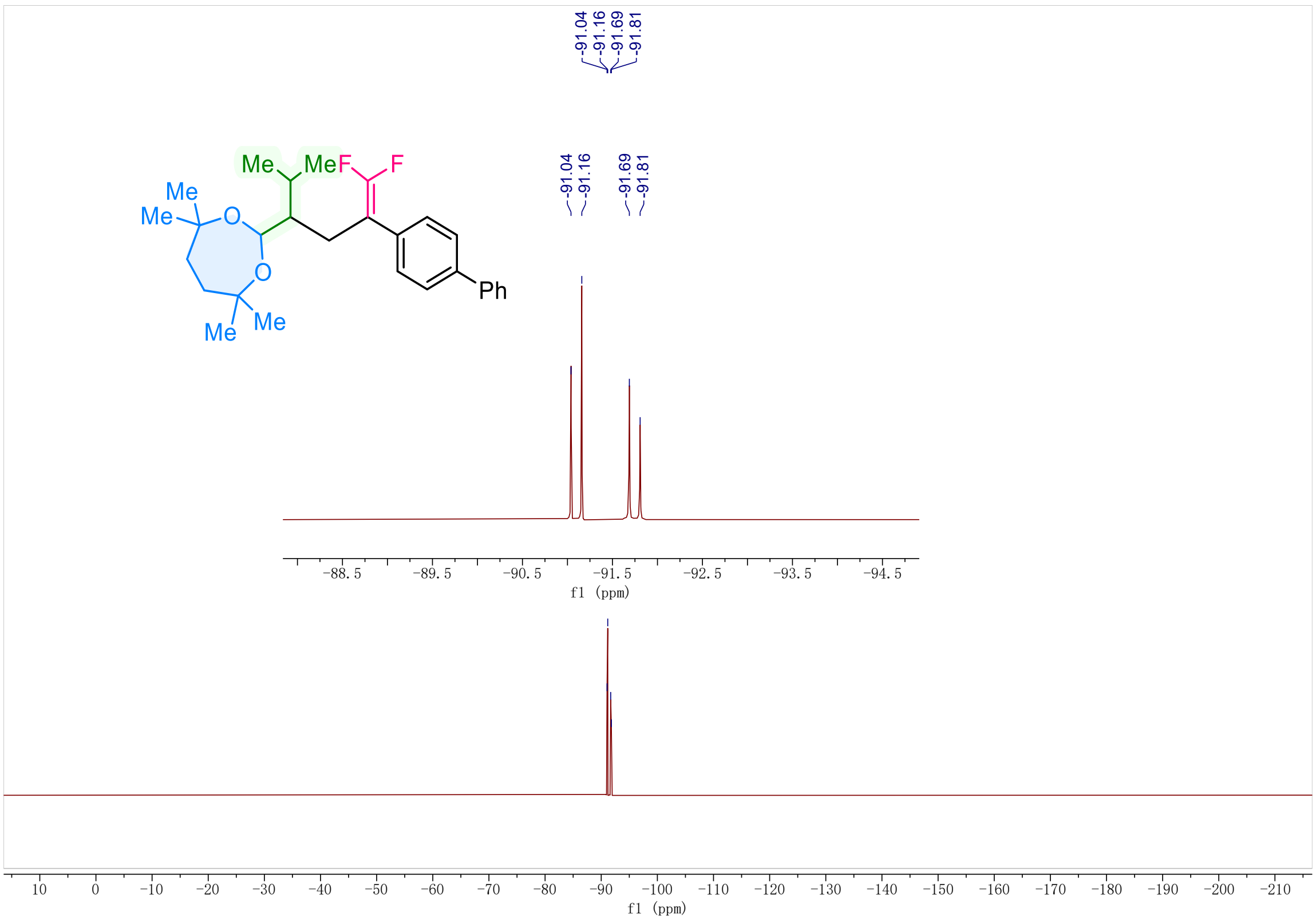

^{19}F NMR spectrum (377 MHz, Chloroform-*d*) of **10b**

2-(4-([1,1'-Biphenyl]-4-yl)-5,5-difluoropent-4-en-2-yl)-4,4,7,7-tetramethyl-1,3-dioxepane (Scheme 2, product 10c)

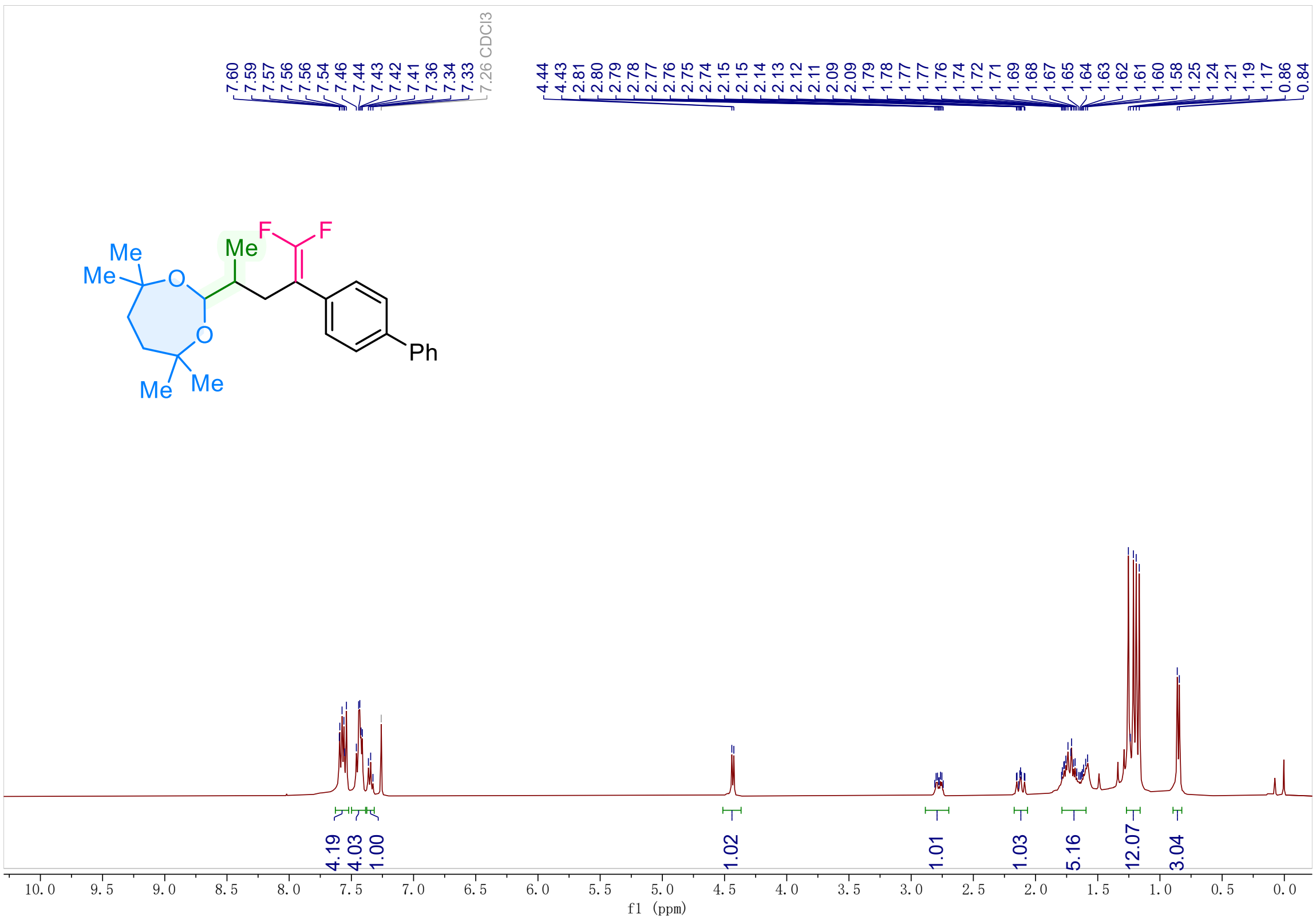

^{1}H NMR spectrum (400 MHz, Chloroform-*d*) of **10c**

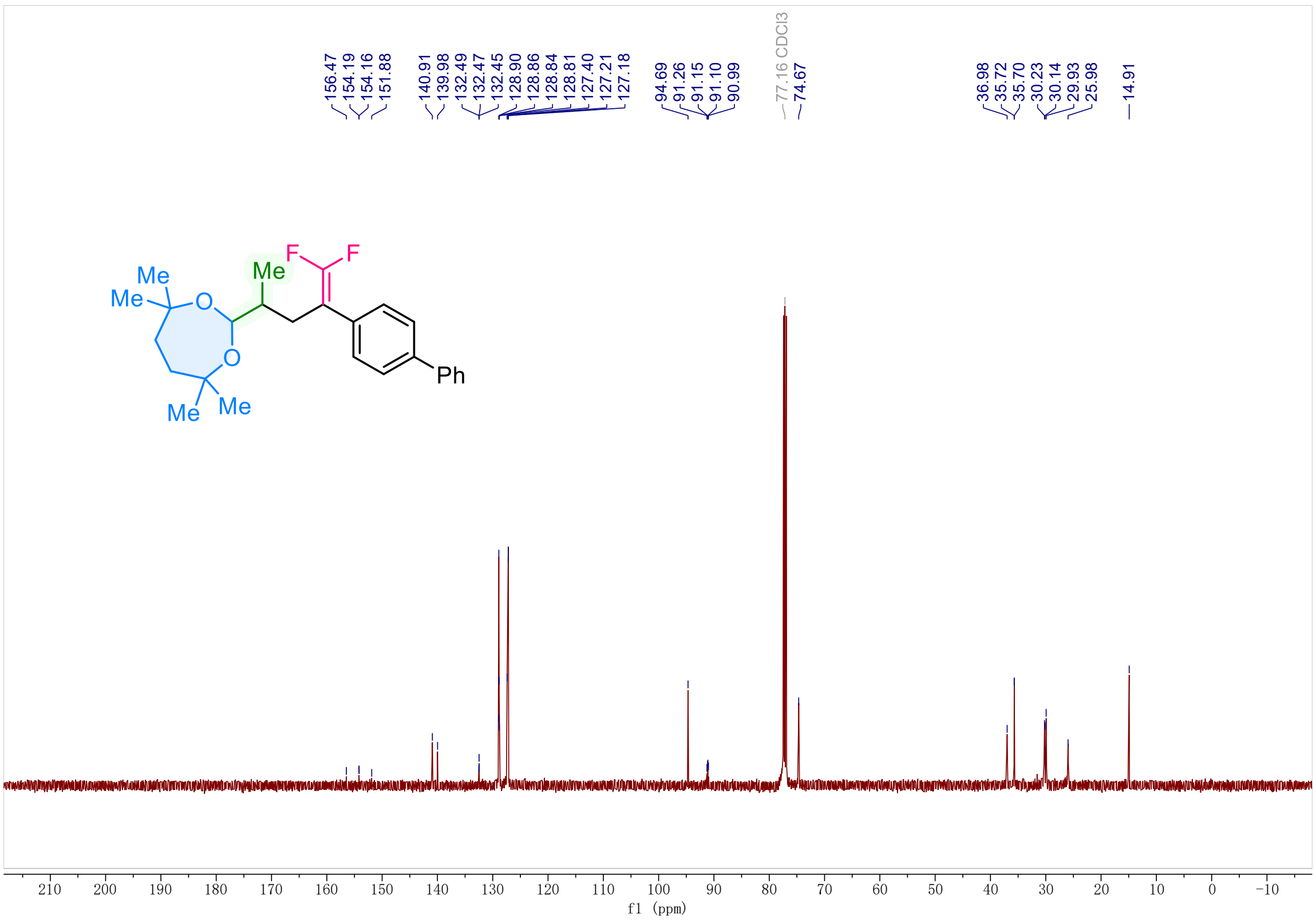

^{13}C NMR spectrum (125 MHz, Chloroform-*d*) of **10c**

2-(4-([1,1'-Biphenyl]-4-yl)-5,5-difluoropent-4-en-2-yl)-4,4,7,7-tetramethyl-1,3-dioxepane (Scheme 2, product 10c)

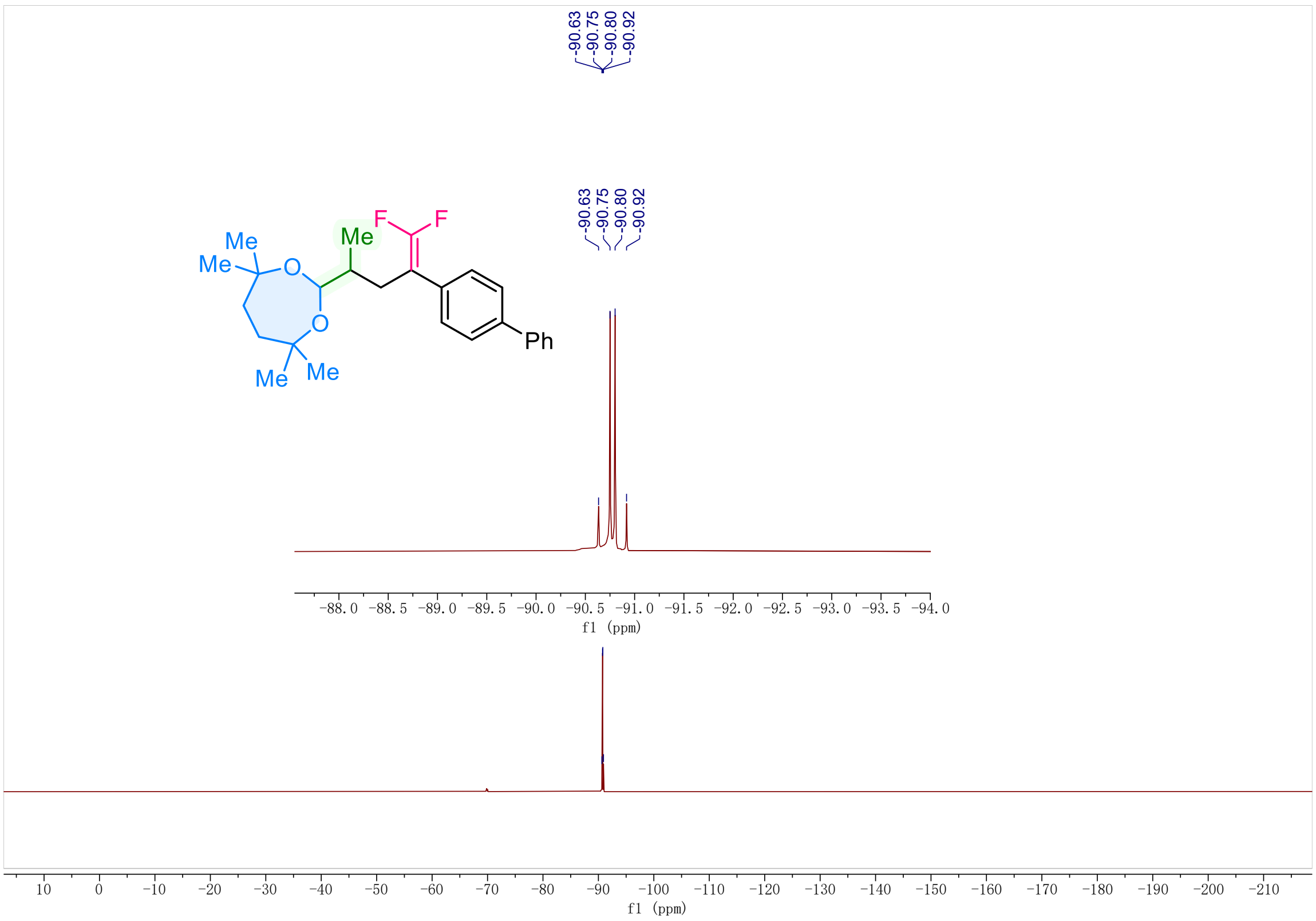

^{19}F NMR spectrum (377 MHz, Chloroform-*d*) of **10c**

2-(4-([1,1'-Biphenyl]-4-yl)-5,5-difluoro-1-phenylpent-4-en-2-yl)-4,4,7,7-tetramethyl-1,3-dioxepane (Scheme 2, product 10d)

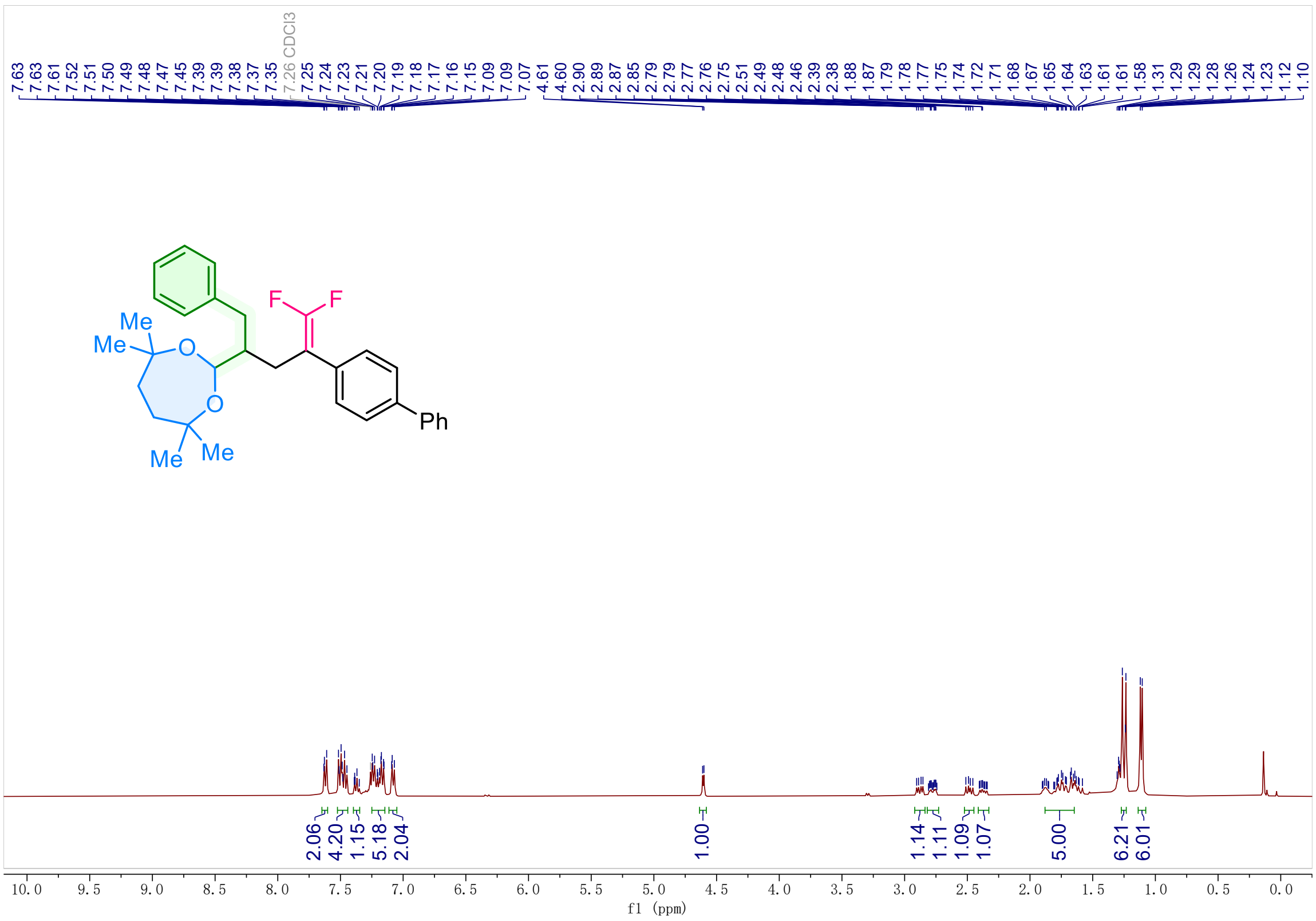

^{1}H NMR spectrum (400 MHz, Chloroform-*d*) of **10d**

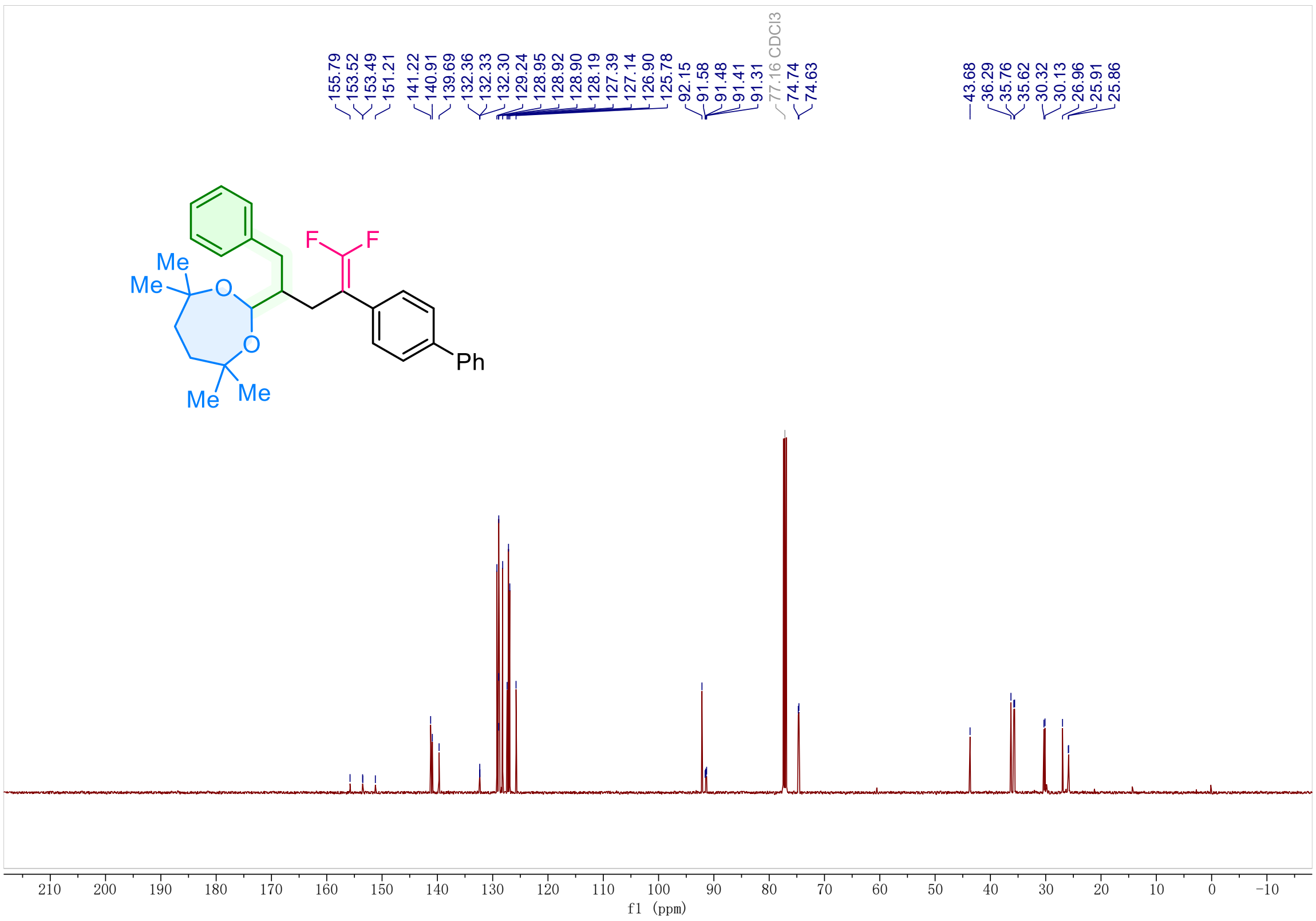

^{13}C NMR spectrum (125 MHz, Chloroform-*d*) of **10d**

2-(4-([1,1'-Biphenyl]-4-yl)-5,5-difluoro-1-phenylpent-4-en-2-yl)-4,4,7,7-tetramethyl-1,3-dioxepane (Scheme 2, product 10d)

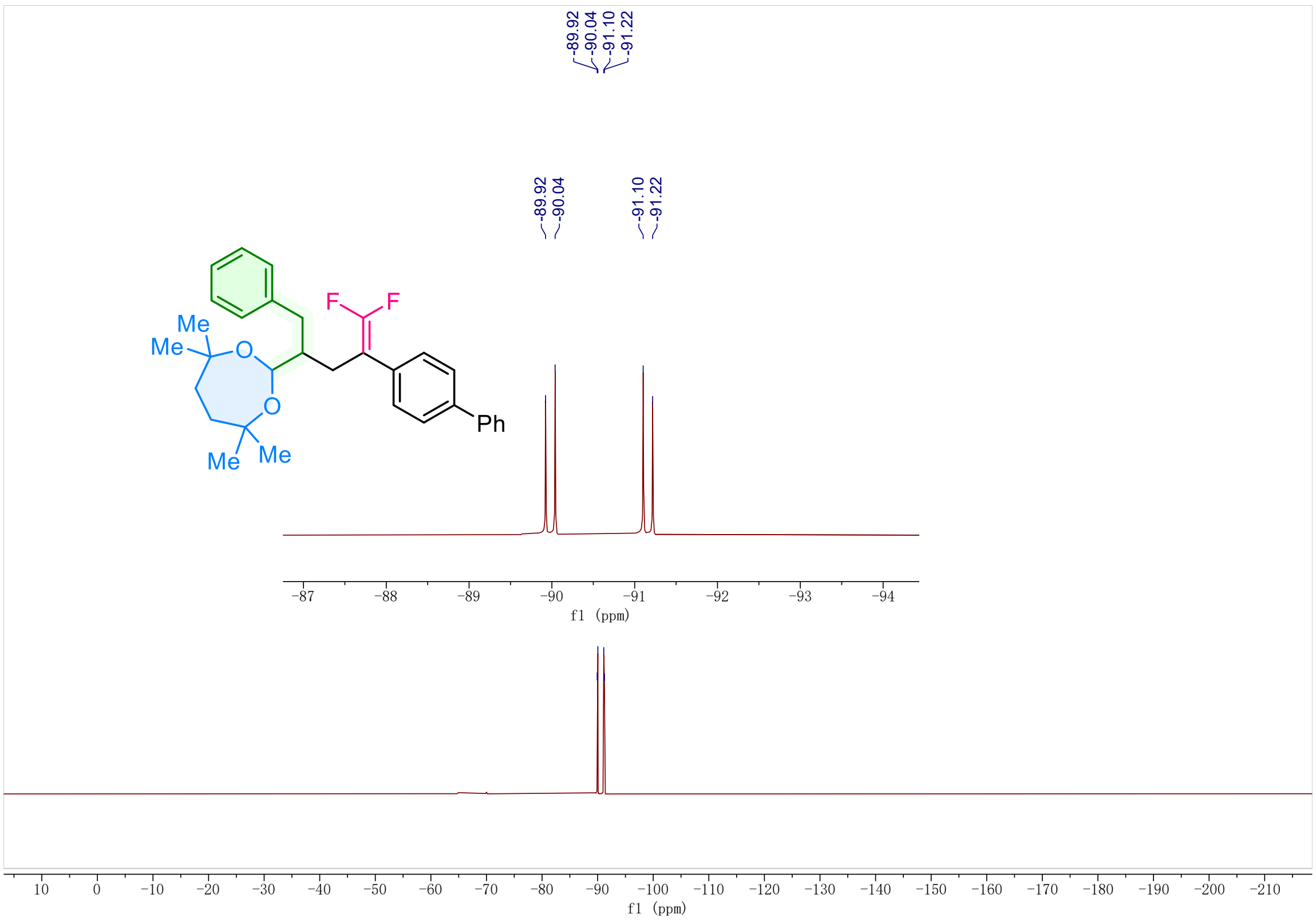

^{19}F NMR spectrum (377 MHz, Chloroform-*d*) of **10d**

2-(2-([1,1'-Biphenyl]-4-yl)-1,1-difluorohepta-1,6-dien-4-yl)-4,4,7,7-tetramethyl-1,3-dioxepane (Scheme 2, product 10e)

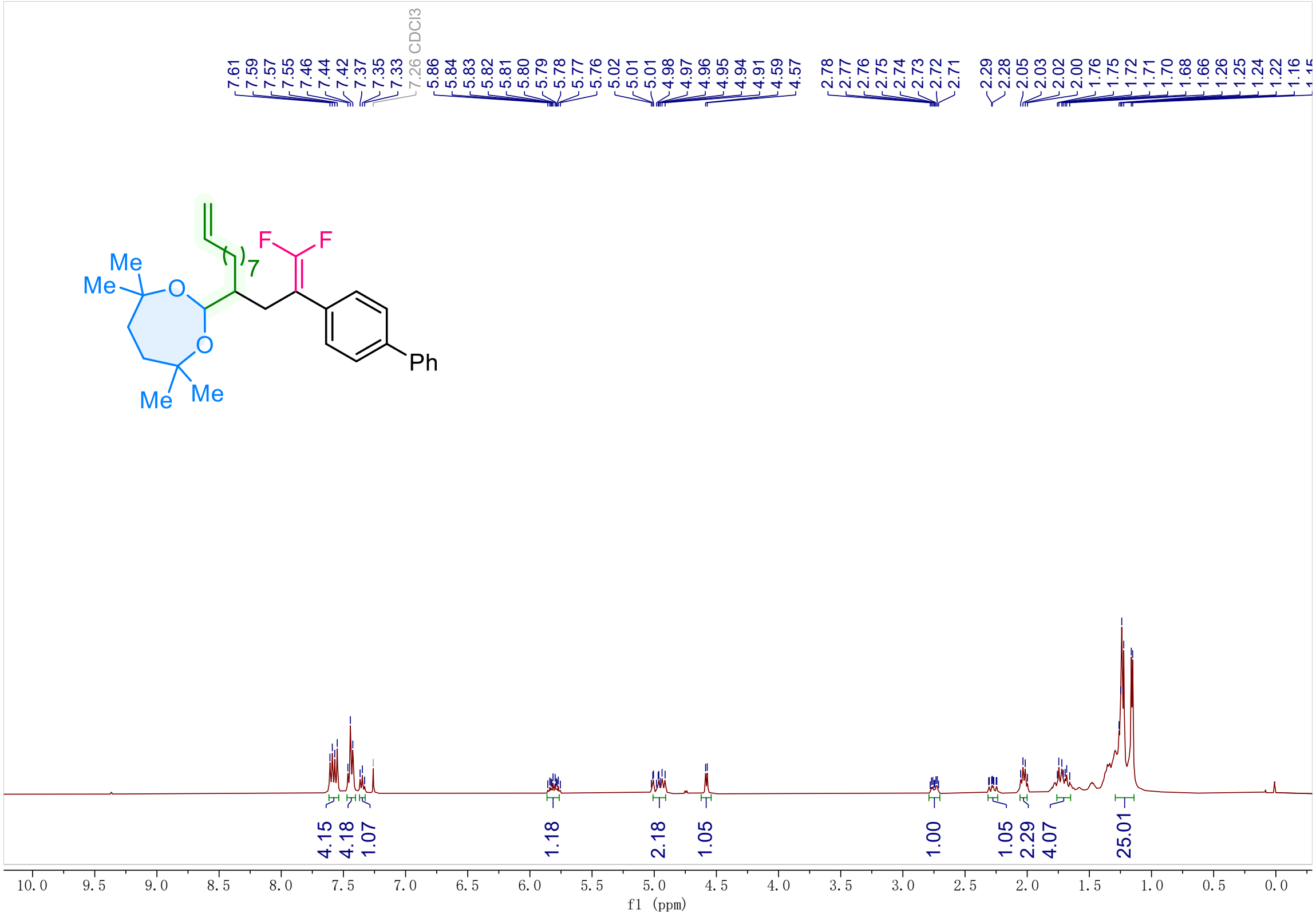

^{1}H NMR spectrum (400 MHz, Chloroform-*d*) of **10e**

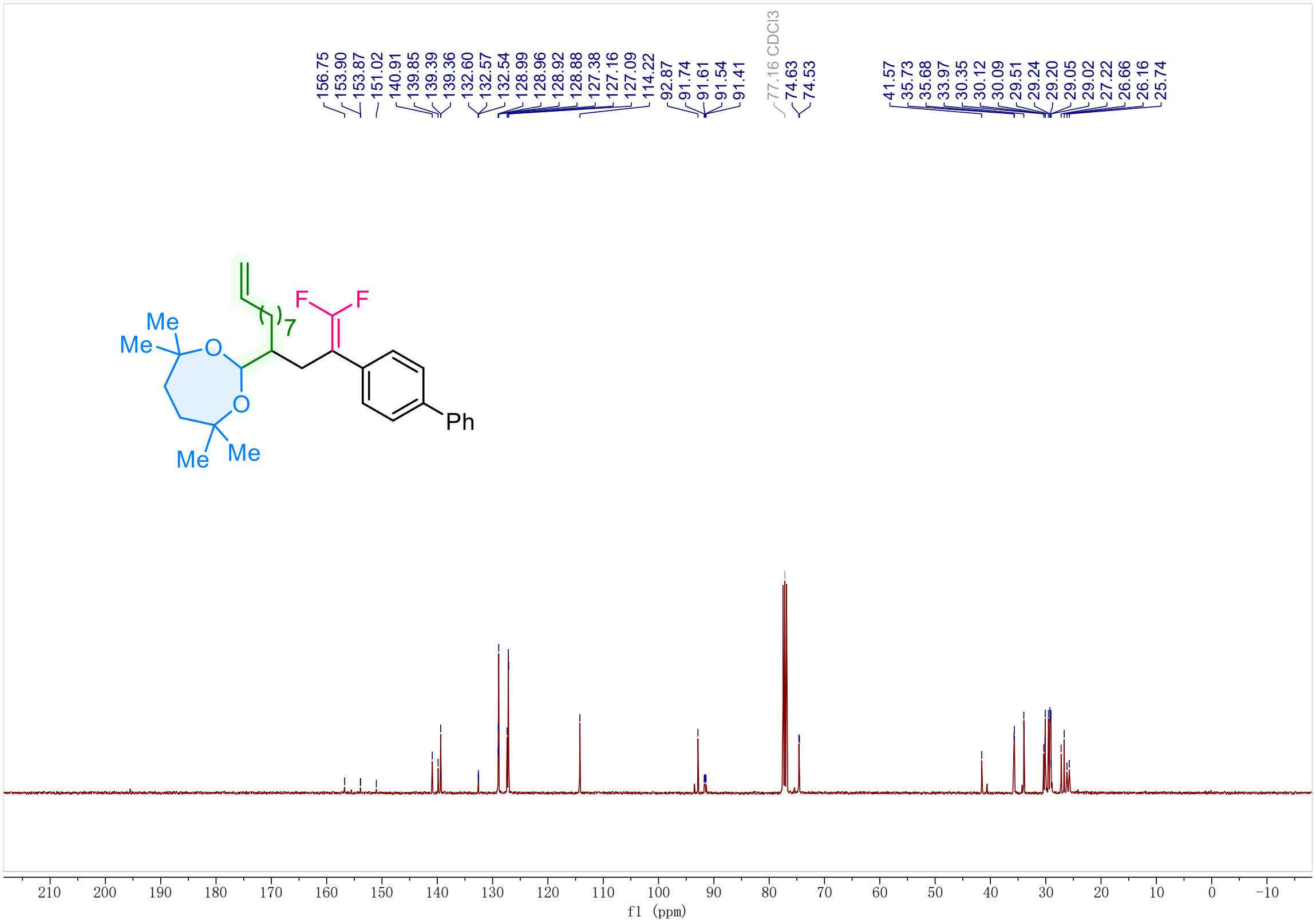

^{13}C NMR spectrum (100 MHz, Chloroform-*d*) of **10e**

2-(2-([1,1'-Biphenyl]-4-yl)-1,1-difluorohepta-1,6-dien-4-yl)-4,4,7,7-tetramethyl-1,3-dioxepane (Scheme 2, product 10e)

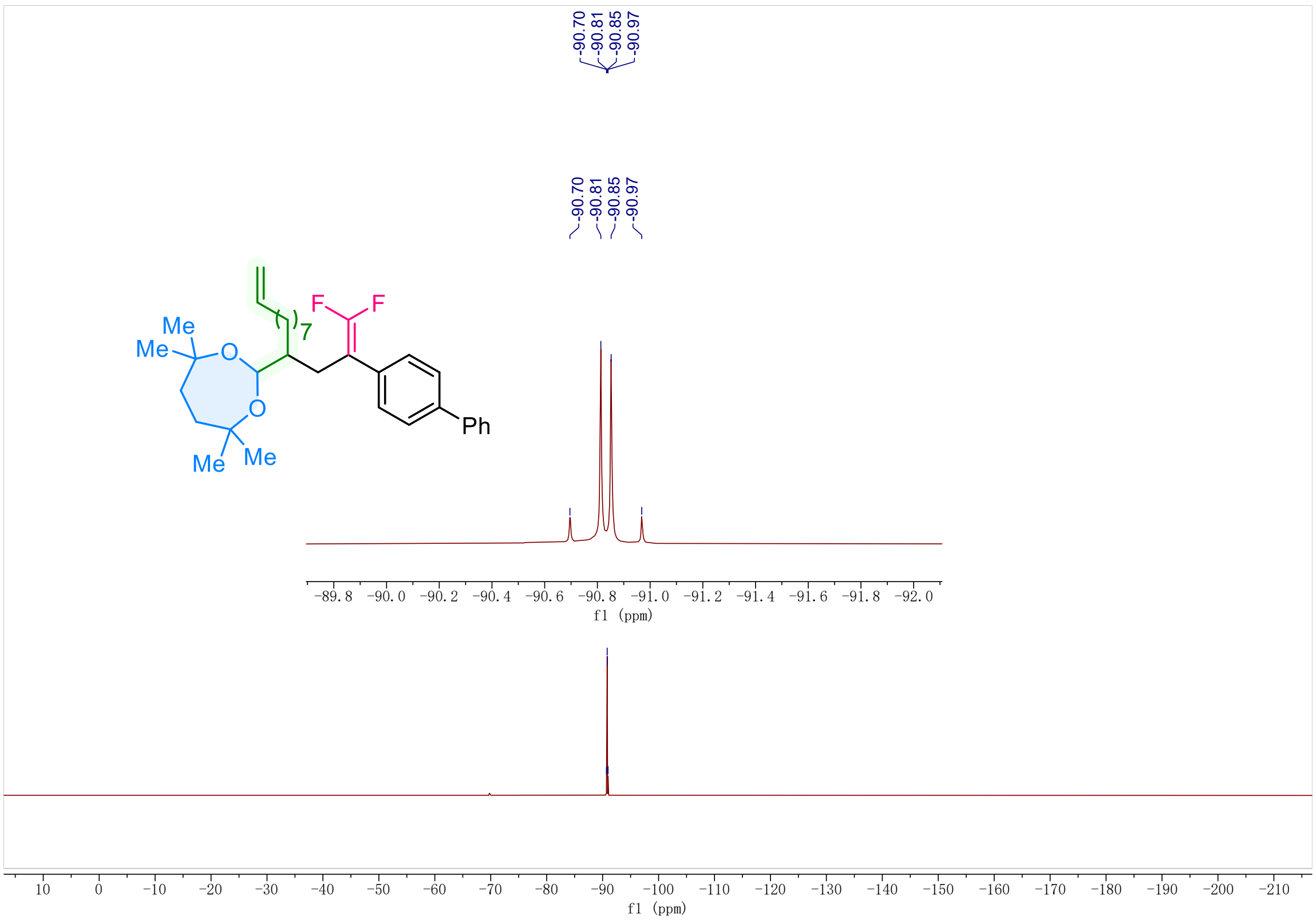

^{19}F NMR spectrum (377 MHz, Chloroform-*d*) of **10e**

1-(2-([1,1'-Biphenyl]-4-yl)-1-fluoro-4-(4,4,7,7-tetramethyl-1,3-dioxepan-2-yl)pent-1-en-1-yl)-1*H*-imidazole (Scheme 3, product 11)

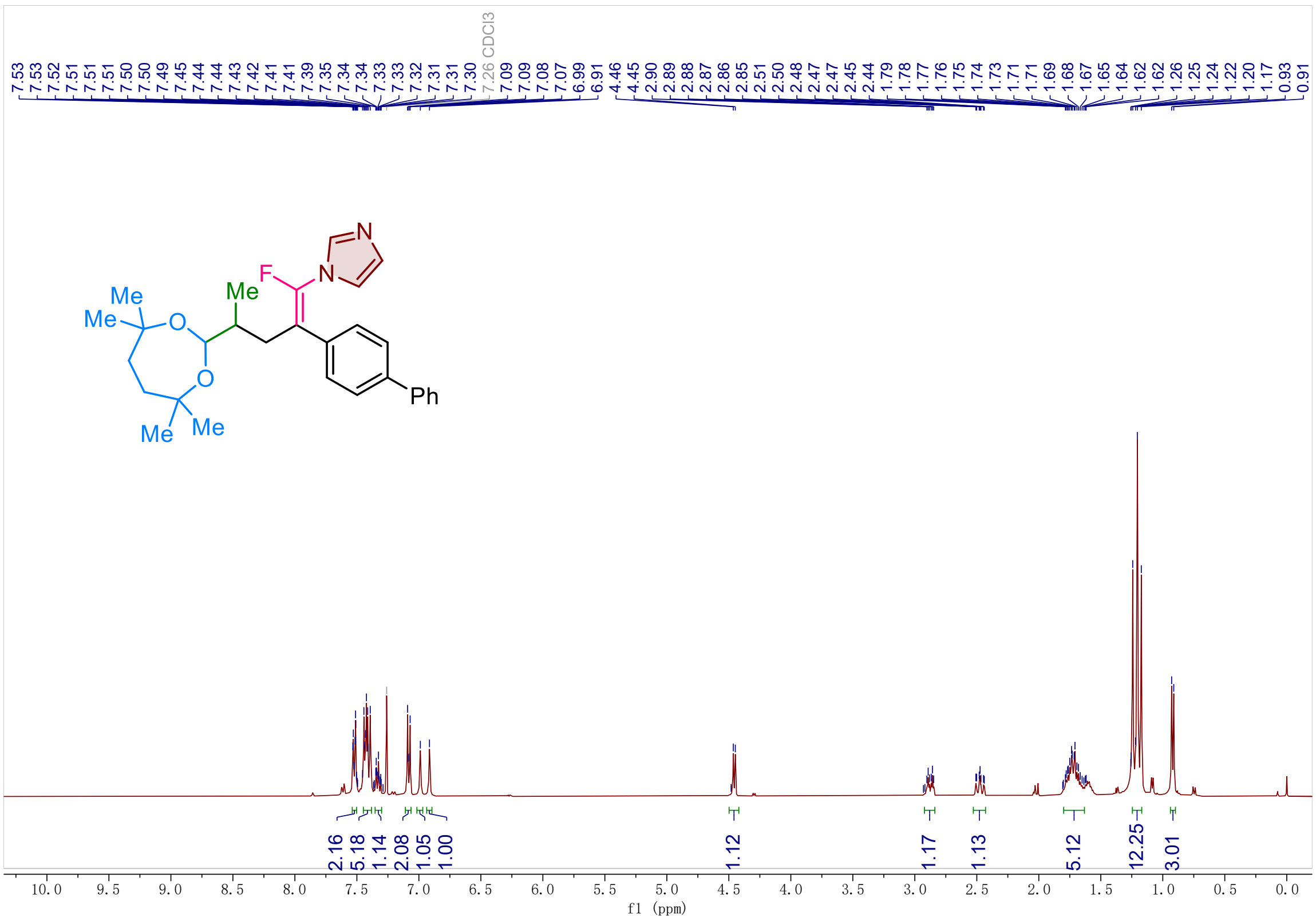

^{1}H NMR spectrum (400 MHz, Chloroform-*d*) of **11**

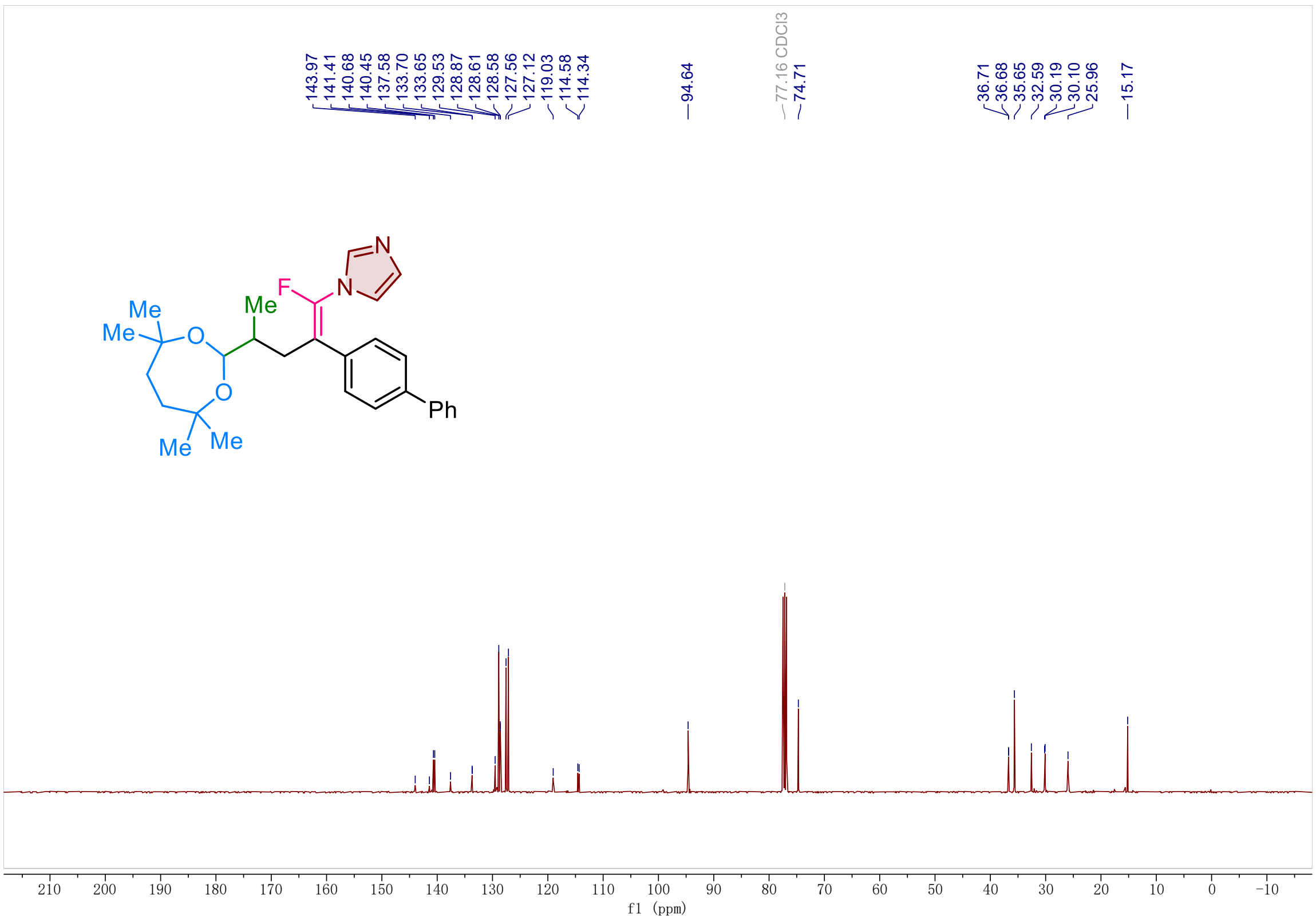

^{13}C NMR spectrum (100 MHz, Chloroform-*d*) of **11**

1-(2-([1,1'-Biphenyl]-4-yl)-1-fluoro-4-(4,4,7,7-tetramethyl-1,3-dioxepan-2-yl)pent-1-en-1-yl)-1*H*-imidazole (Scheme 3, product 11)

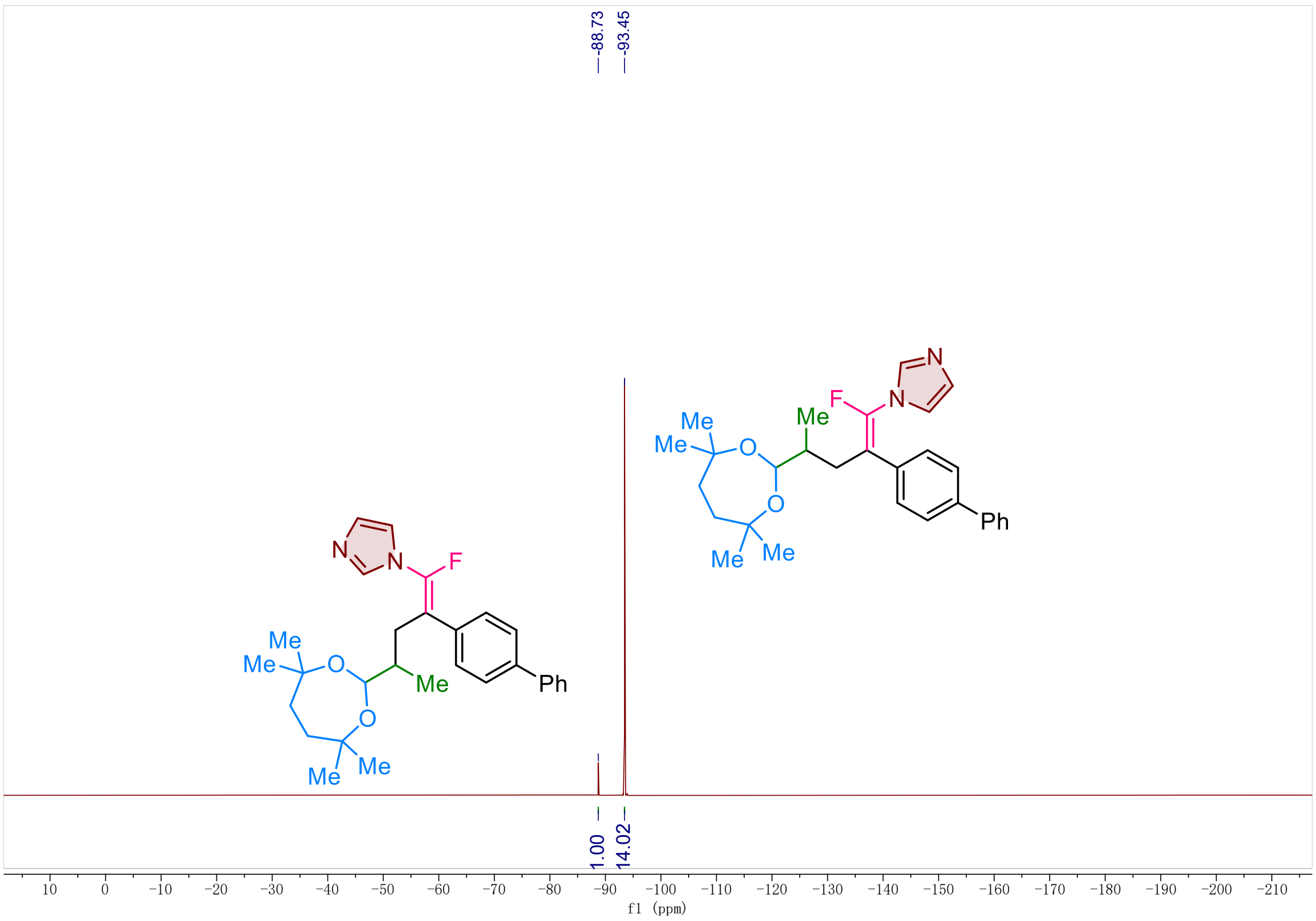

^{19}F NMR spectrum (377 MHz, Chloroform-*d*) of **11**

4-([1,1'-Biphenyl]-4-yl)-5,5-difluoro-2-methylpent-4-enal (Scheme 3, product 12)

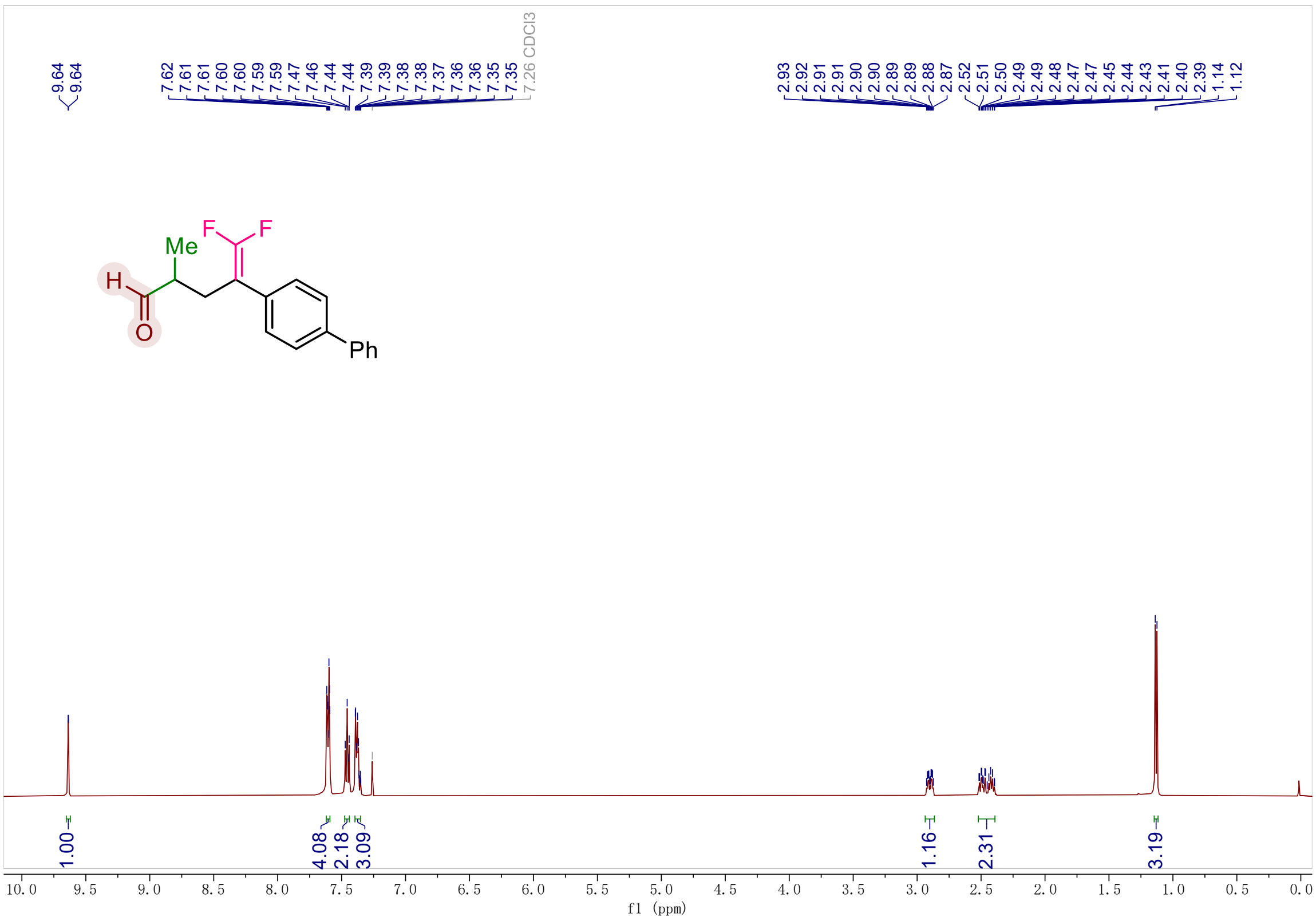

^{1}H NMR spectrum (500 MHz, Chloroform-*d*) of **12**

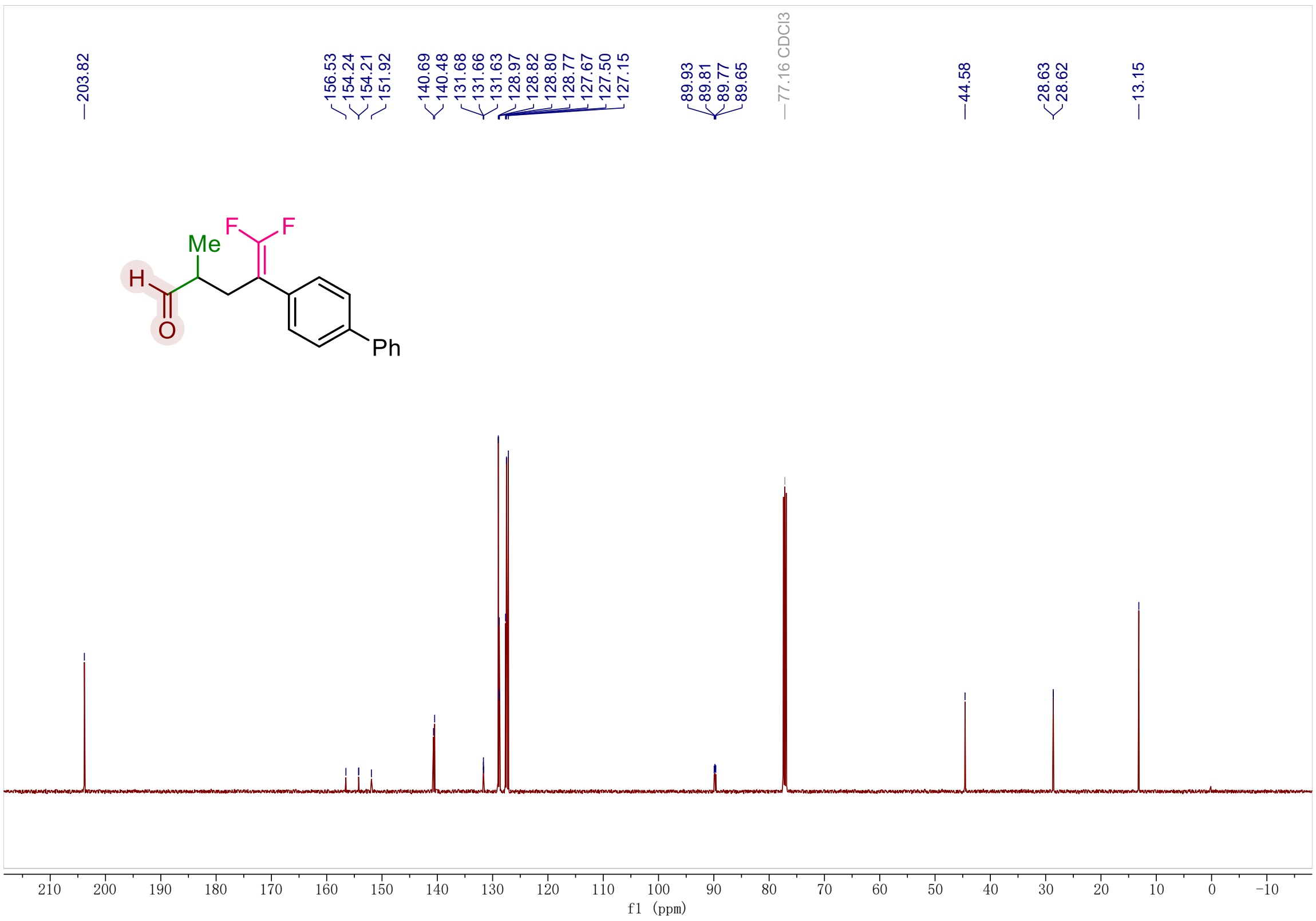

^{13}C NMR spectrum (125 MHz, Chloroform-*d*) of **12**

4-([1,1'-Biphenyl]-4-yl)-5,5-difluoro-2-methylpent-4-enal (Scheme 3, product 12)

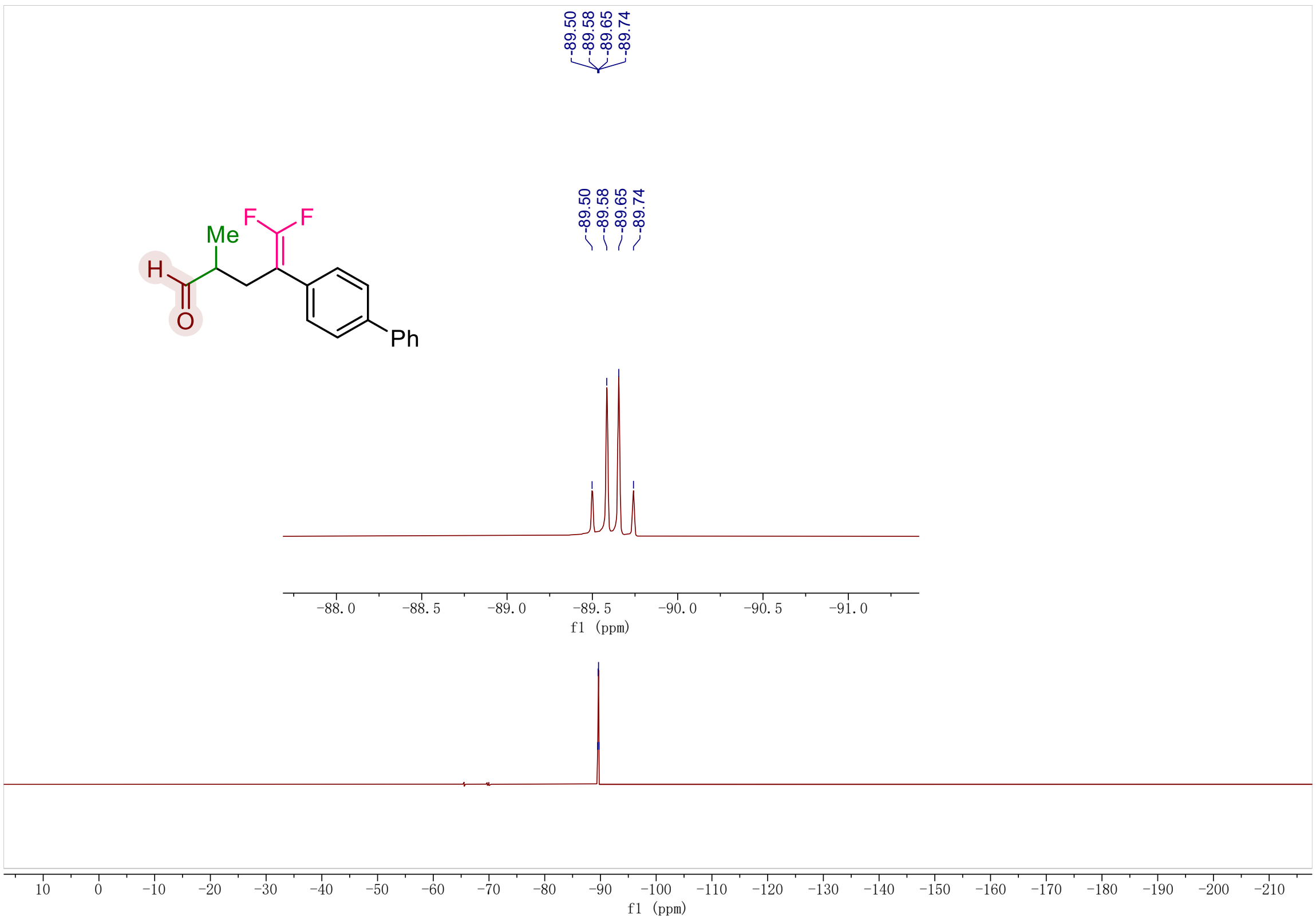

^{19}F NMR spectrum (471 MHz, Chloroform-*d*) of **12**

2-(2-([1,1'-Biphenyl]-4-yl)-3,3-difluoroallyl)cyclohexan-1-one (Scheme 3, product 13)

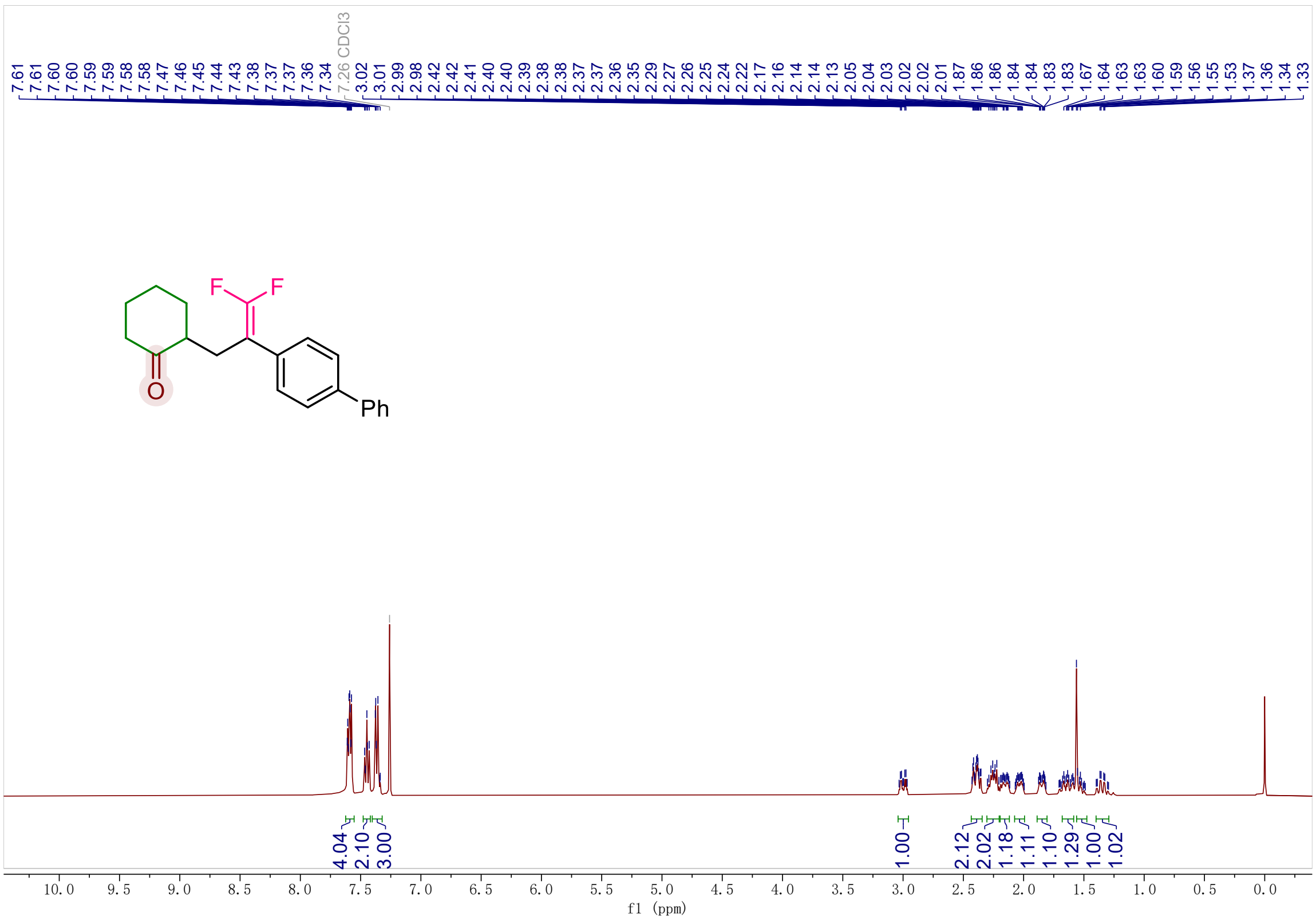

^{1}H NMR spectrum (400 MHz, Chloroform-*d*) of **13**

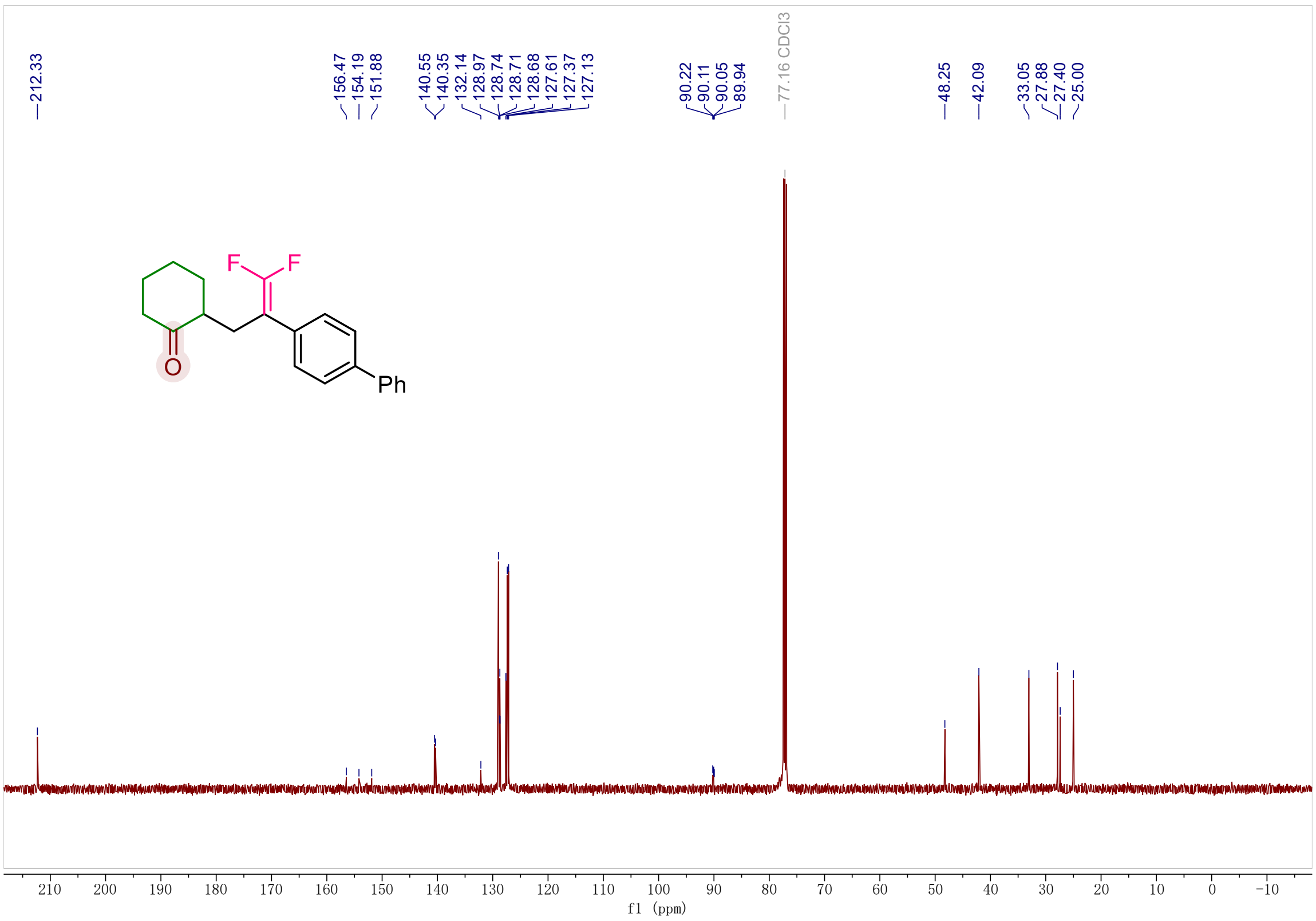

^{13}C NMR spectrum (125 MHz, Chloroform-*d*) of **13**

2-(2-([1,1'-Biphenyl]-4-yl)-3,3-difluoroallyl)cyclohexan-1-one (Scheme 3, product 13)

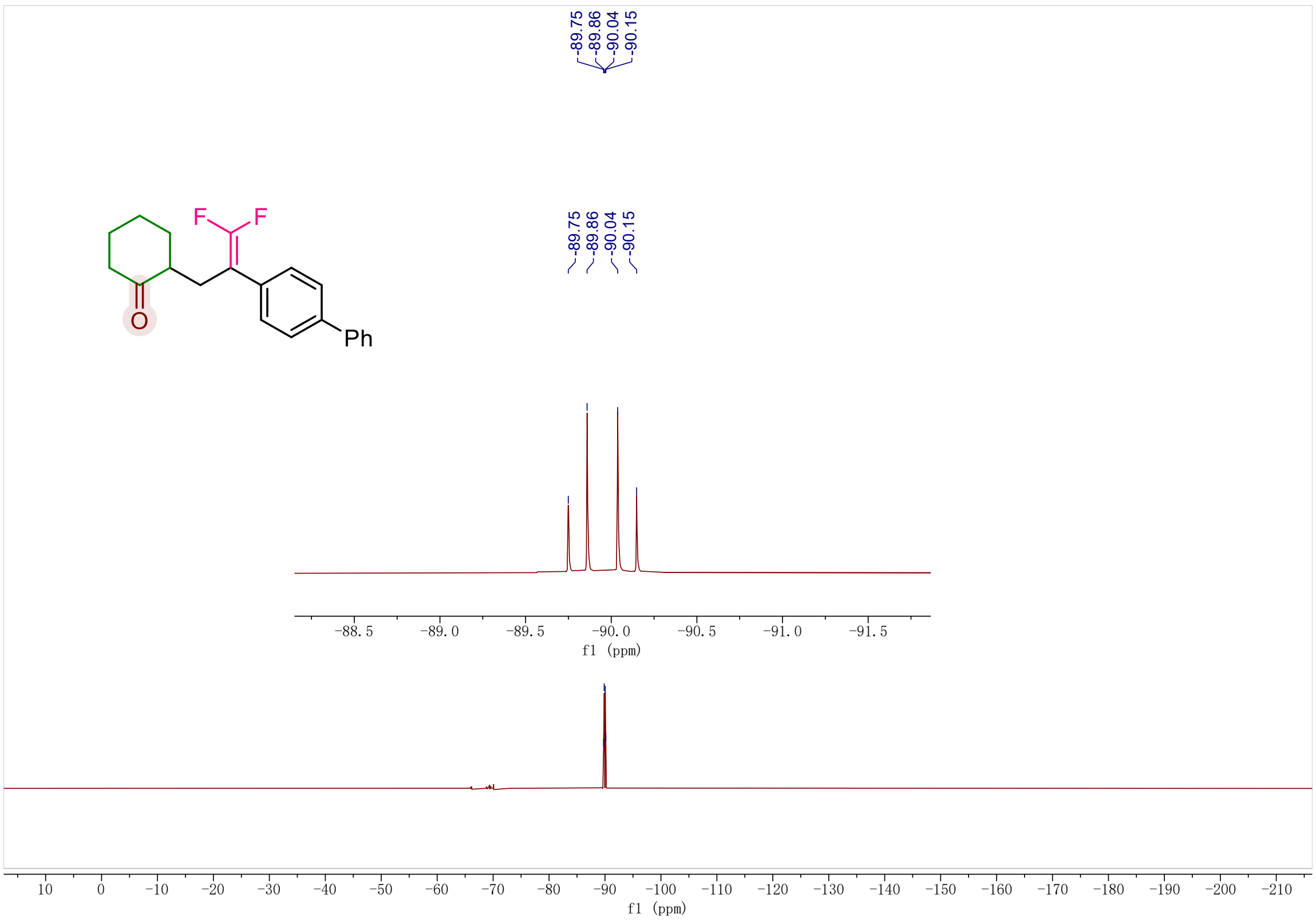

^{19}F NMR spectrum (377 MHz, Chloroform-*d*) of **13**

4-(3-(2,2-Difluorocyclohexyl)-1,1-difluoroprop-1-en-2-yl)-1,1'-biphenyl (Scheme 3, product 14)

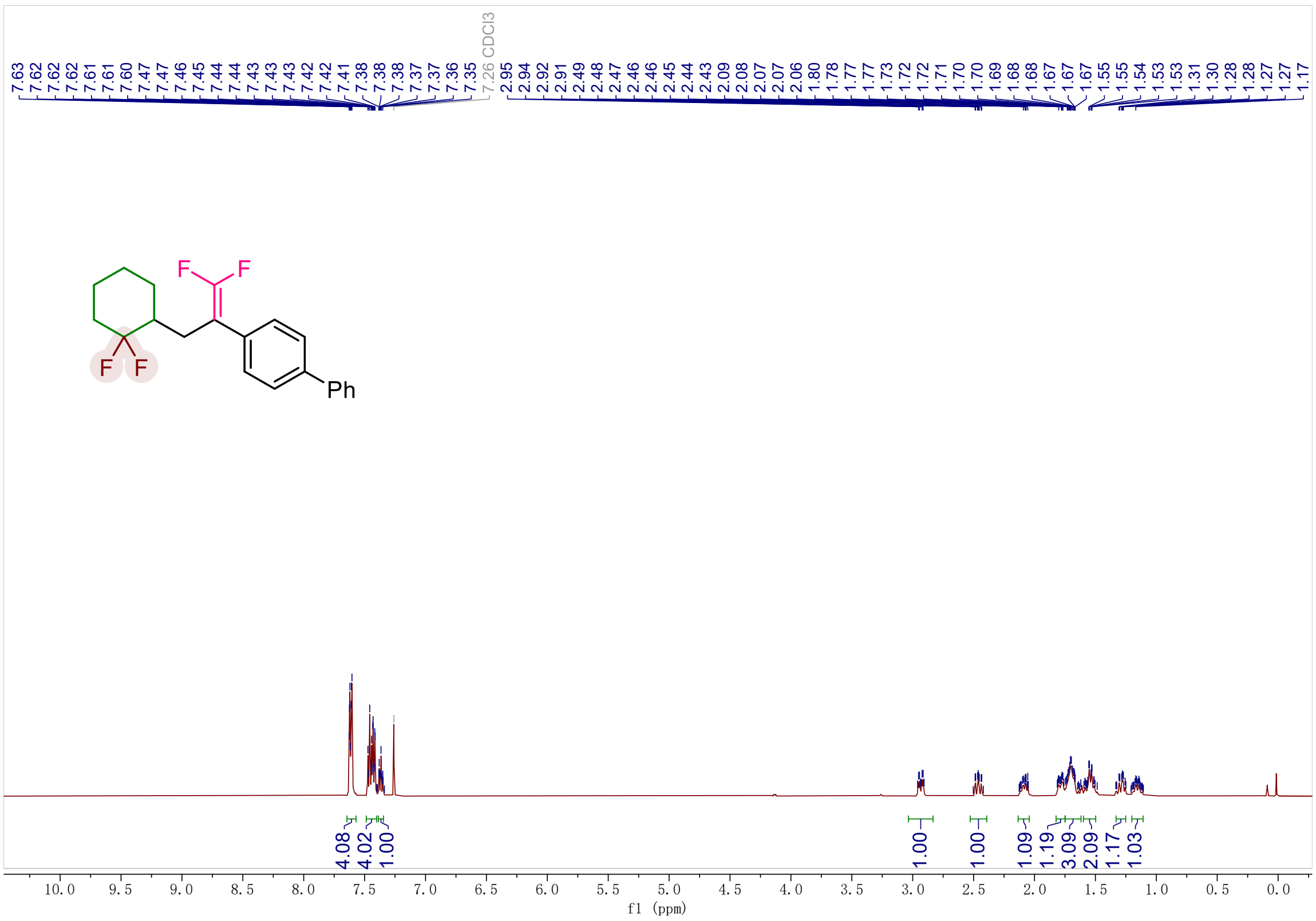

^{1}H NMR spectrum (500 MHz, Chloroform-*d*) of **14**

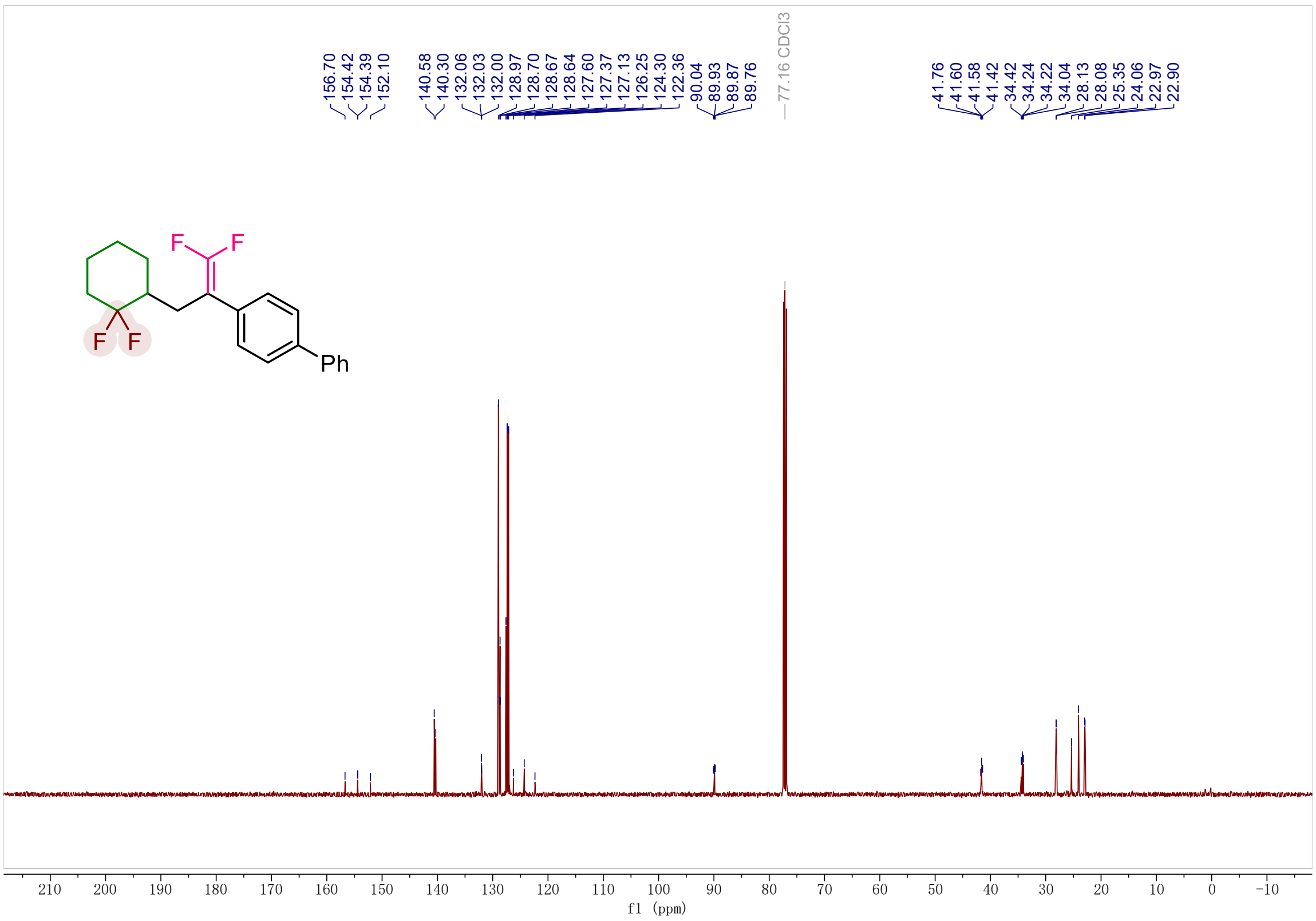

^{13}C NMR spectrum (125 MHz, Chloroform-*d*) of **14**

4-(3-(2,2-Difluorocyclohexyl)-1,1-difluoroprop-1-en-2-yl)-1,1'-biphenyl (Scheme 3, product 14)

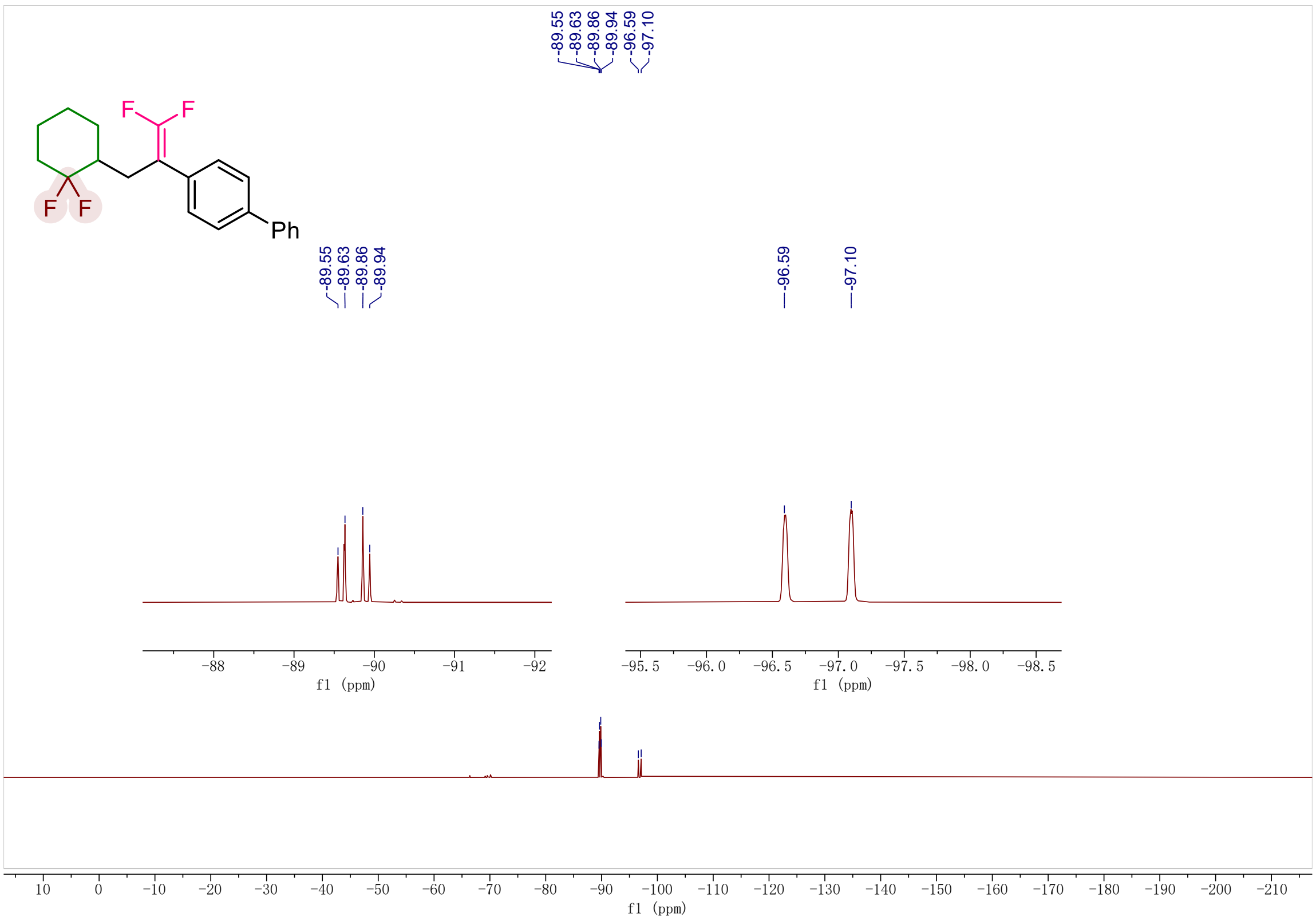

^{19}F NMR spectrum (471 MHz, Chloroform-*d*) of **14**

***N*-(4-(1,1-Difluoro-3-(1,4-dioxaspiro[4.5]decan-6-yl)prop-1-en-2-yl)phenyl)-2-(6-methoxynaphthalen-2-yl)propanamide (Scheme 3, product 15)**

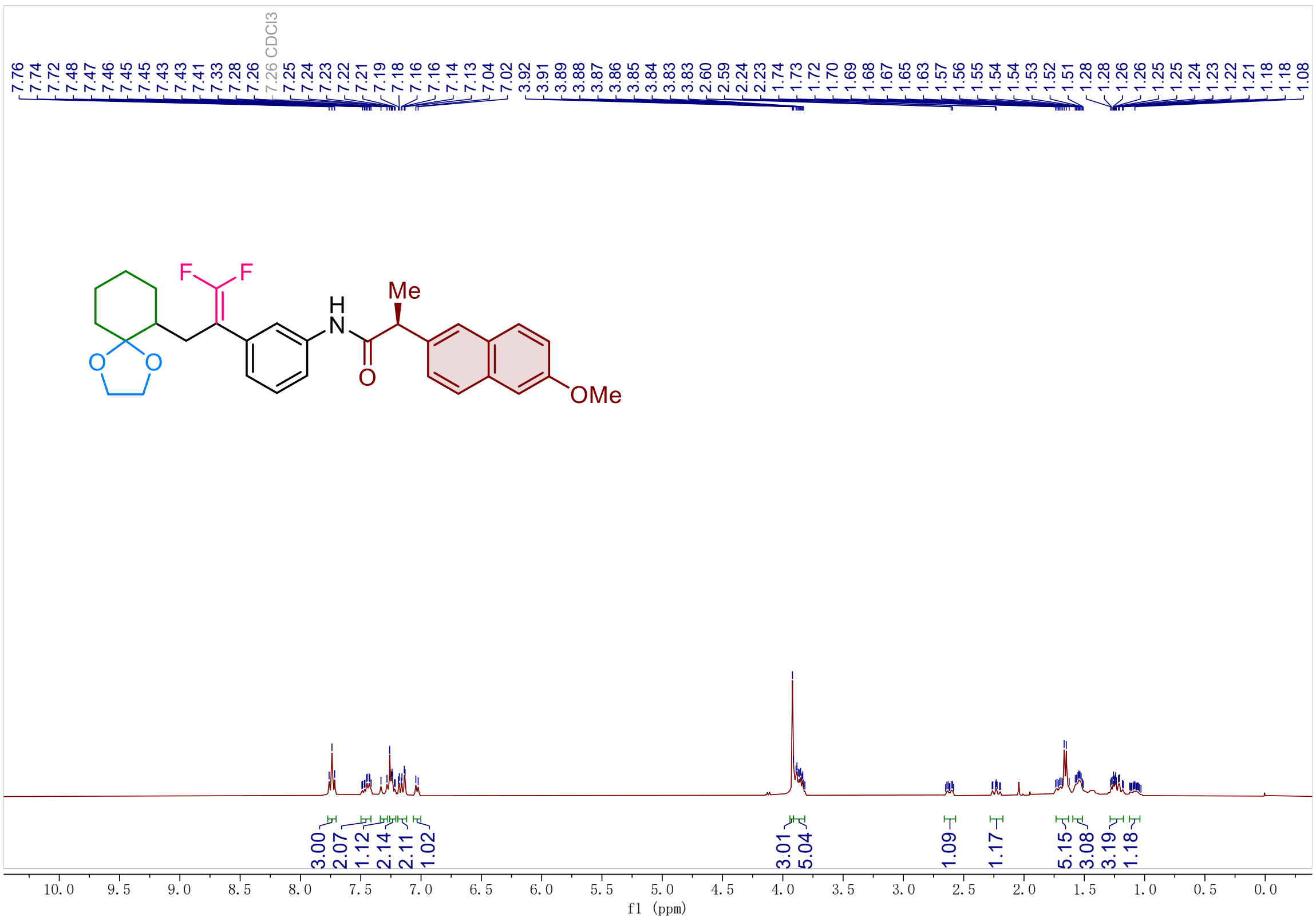

^{1}H NMR spectrum (400 MHz, Chloroform-*d*) of **15**

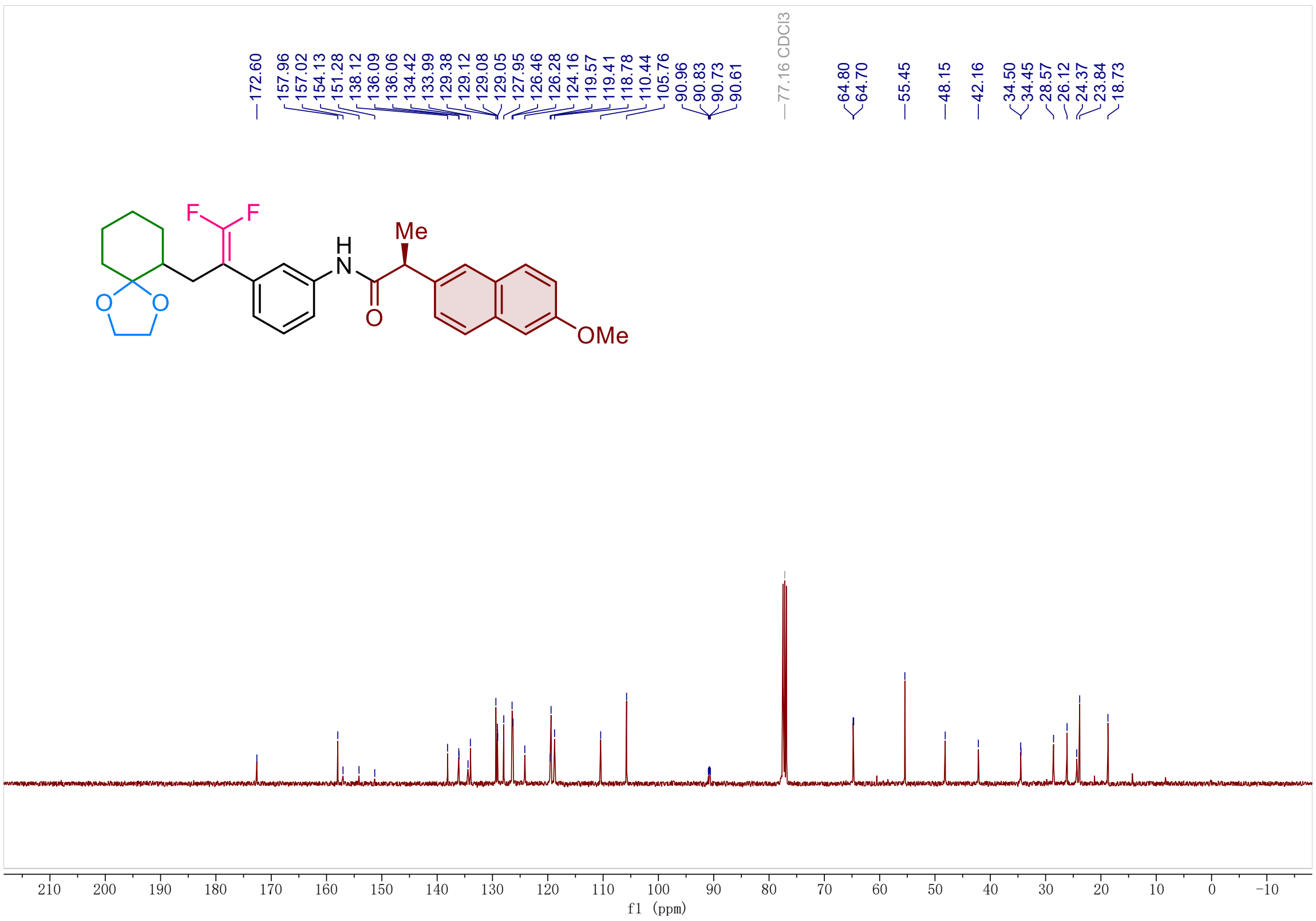

^{13}C NMR spectrum (100 MHz, Chloroform-*d*) of **15**

***N*-(4-(1,1-Difluoro-3-(1,4-dioxaspiro[4.5]decan-6-yl)prop-1-en-2-yl)phenyl)-2-(6-methoxynaphthalen-2-yl)propanamide (Scheme 3, product 15)**

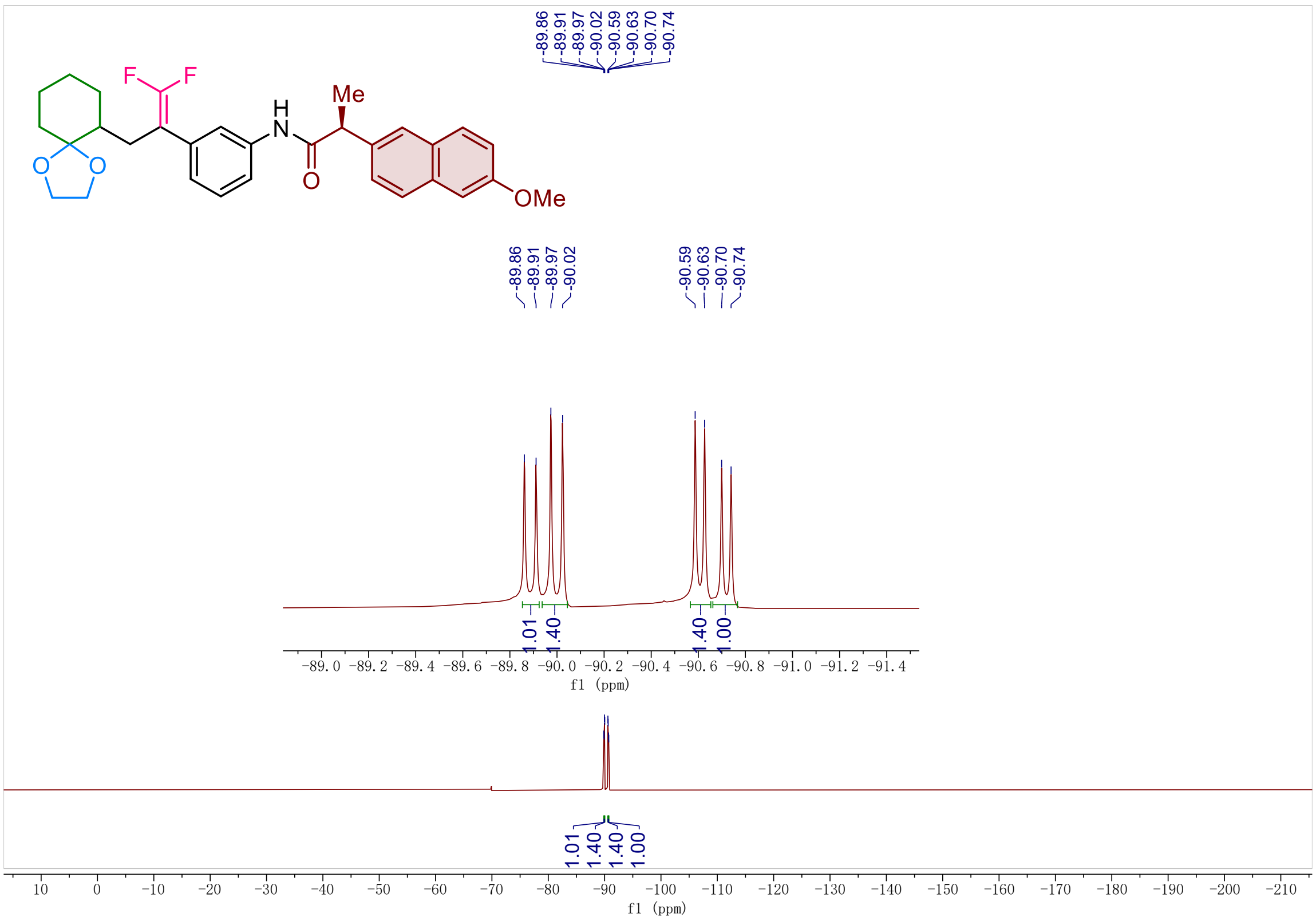

^{19}F NMR spectrum (377 MHz, Chloroform-*d*) of **15**

4-([1,1'-Biphenyl]-4-yl)-5,5-difluoro-2-methylpent-4-en-1-ol (Scheme 3, product 19)

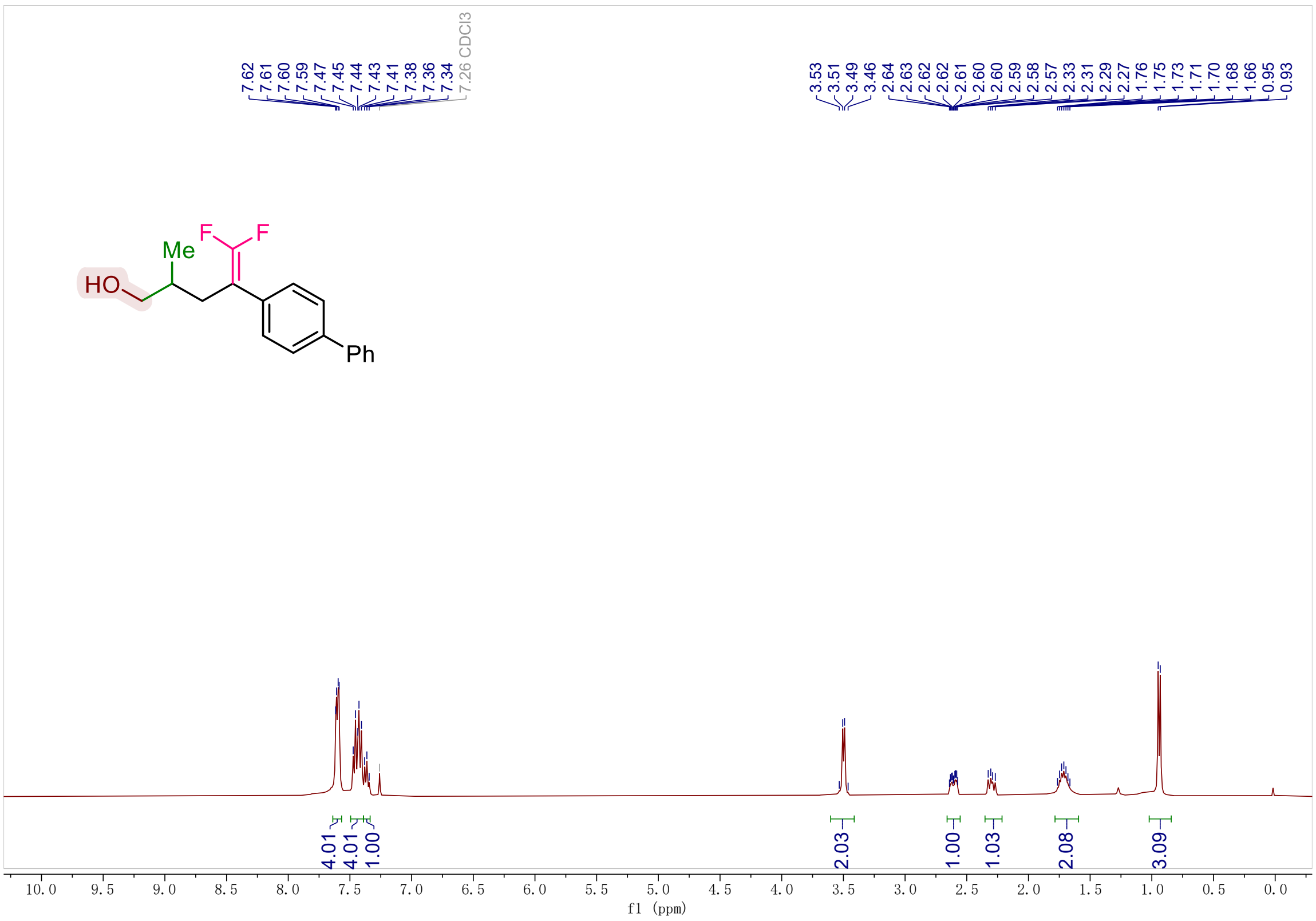

^{1}H NMR spectrum (400 MHz, Chloroform-*d*) of **19**

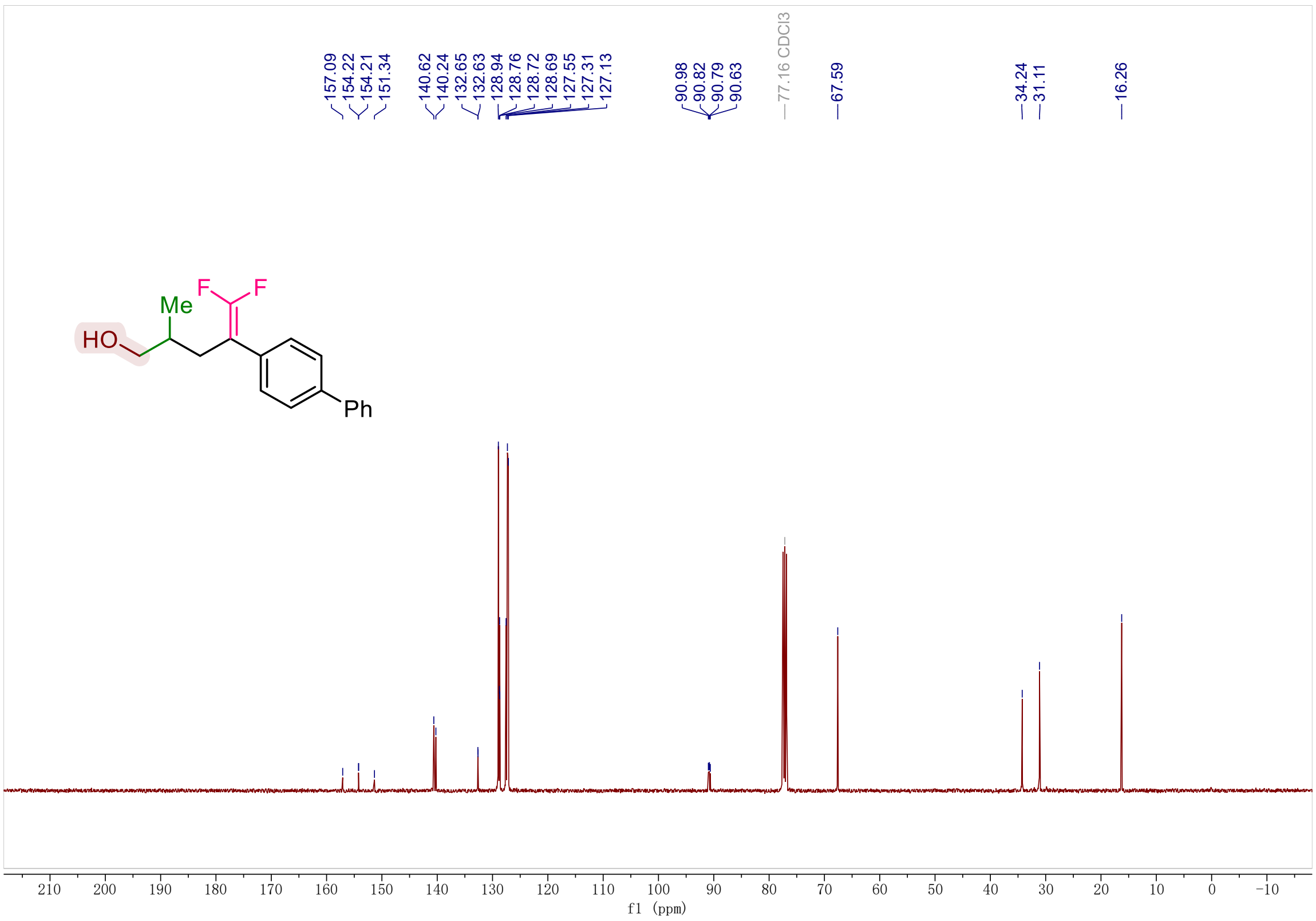

^{13}C NMR spectrum (100 MHz, Chloroform-*d*) of **19**

4-([1,1'-Biphenyl]-4-yl)-5,5-difluoro-2-methylpent-4-en-1-ol (Scheme 3, product 19)

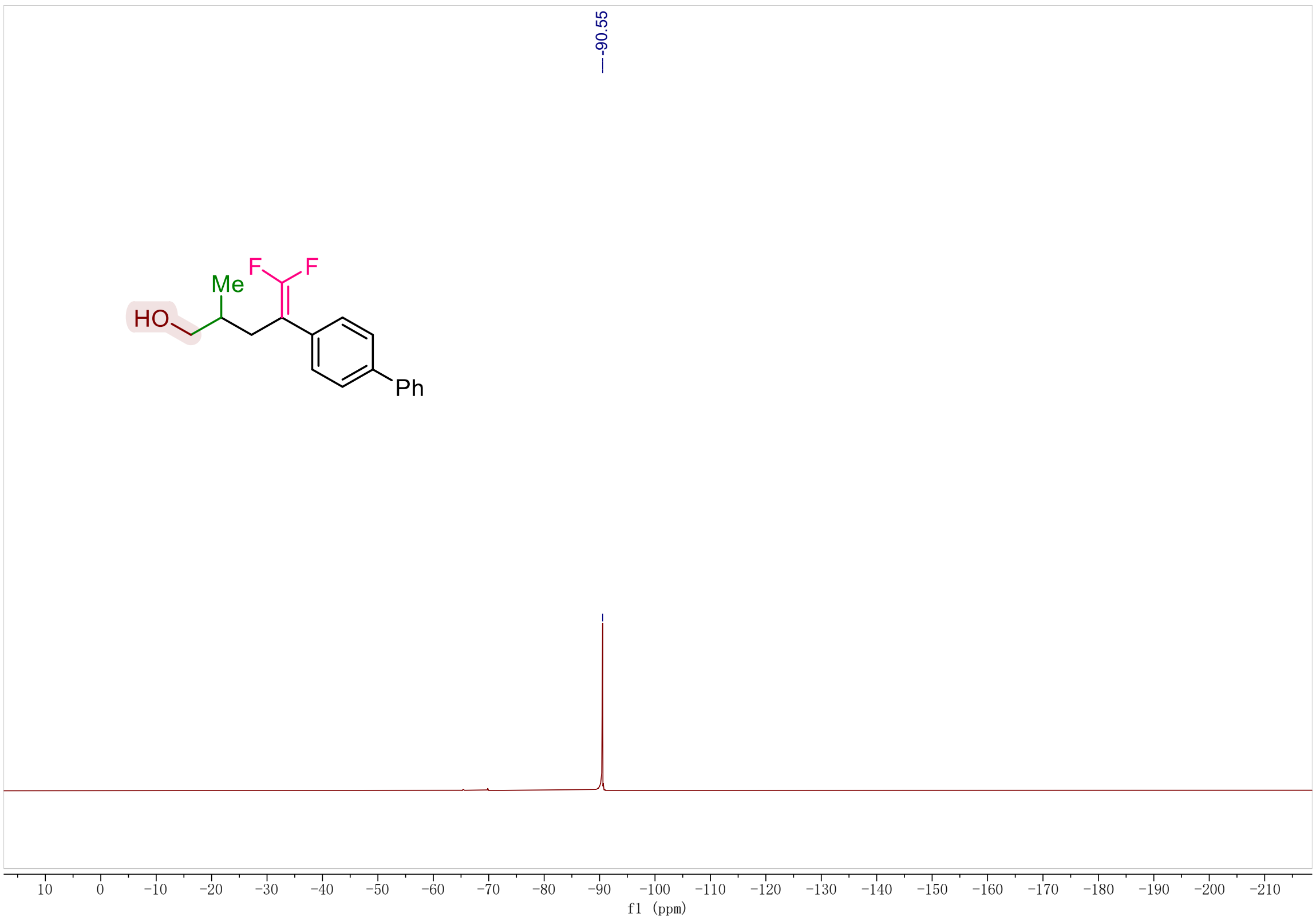

^{19}F NMR spectrum (471 MHz, Chloroform-*d*) of **19**

4-([1,1'-Biphenyl]-4-yl)-5,5-difluoro-2-methylpent-4-en-1-yl acetate (Scheme 3, product 20)

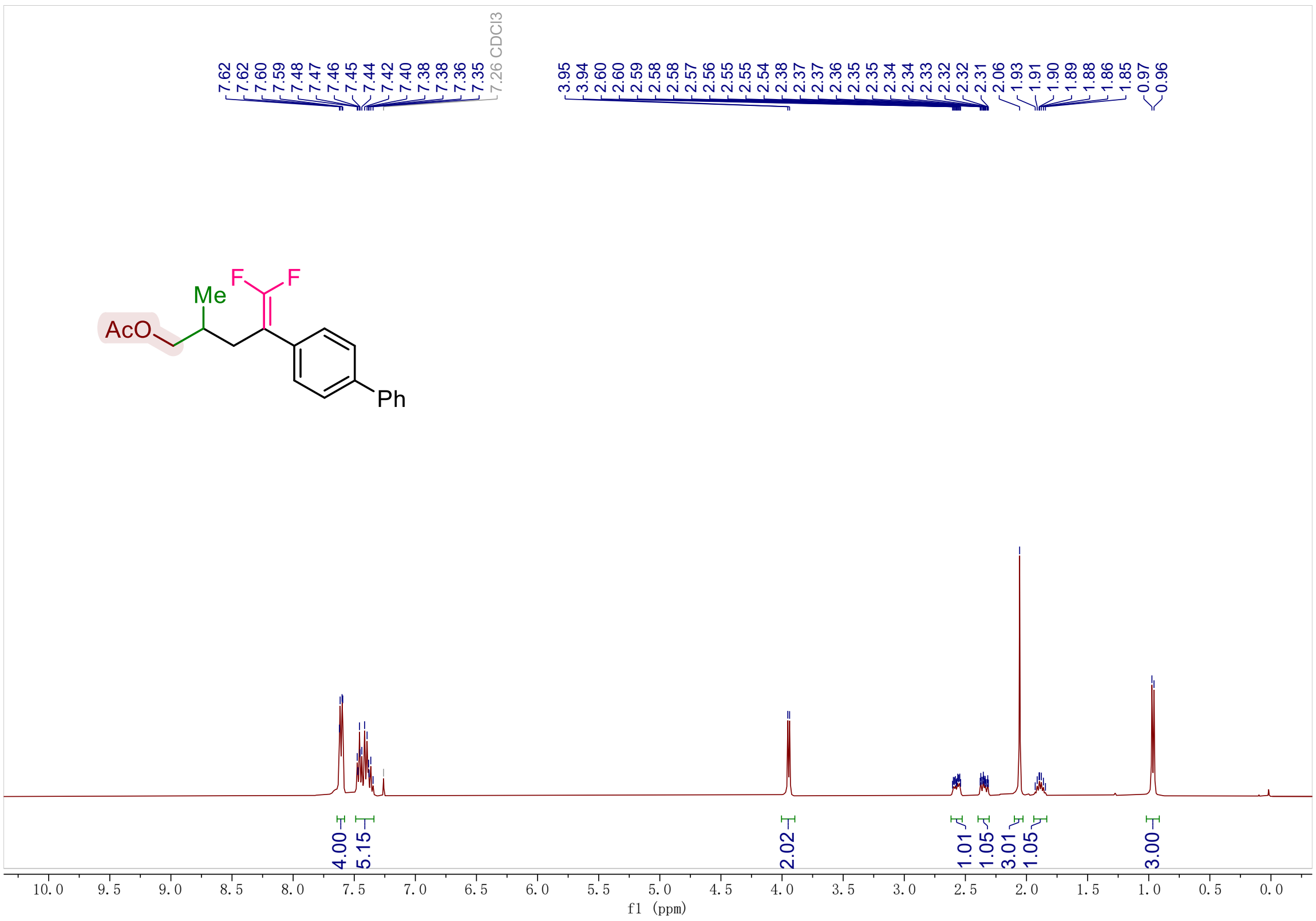

^{1}H NMR spectrum (400 MHz, Chloroform-*d*) of **20**

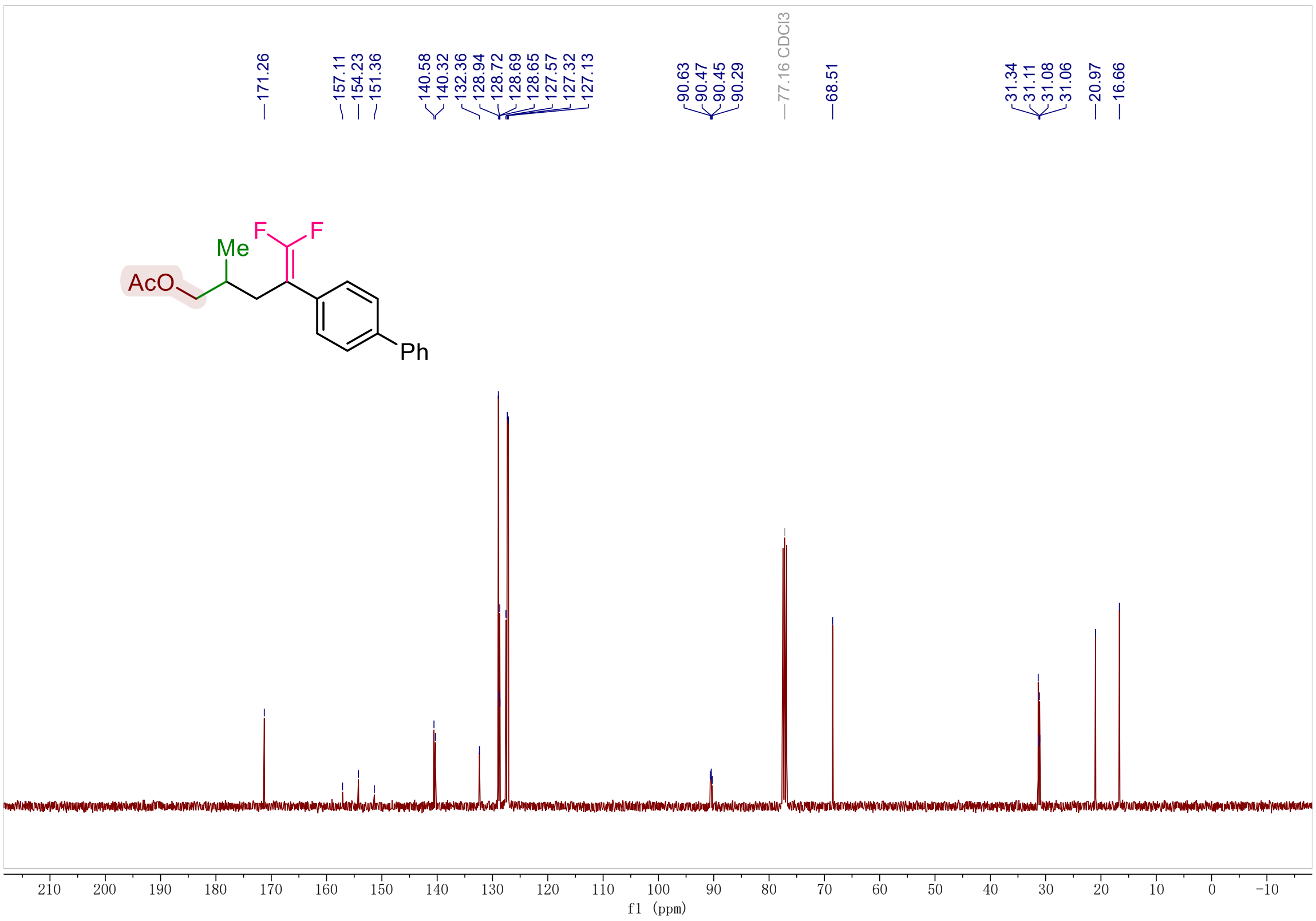

^{13}C NMR spectrum (100 MHz, Chloroform-*d*) of **20**

4-([1,1'-Biphenyl]-4-yl)-5,5-difluoro-2-methylpent-4-en-1-yl acetate (Scheme 3, product 20)

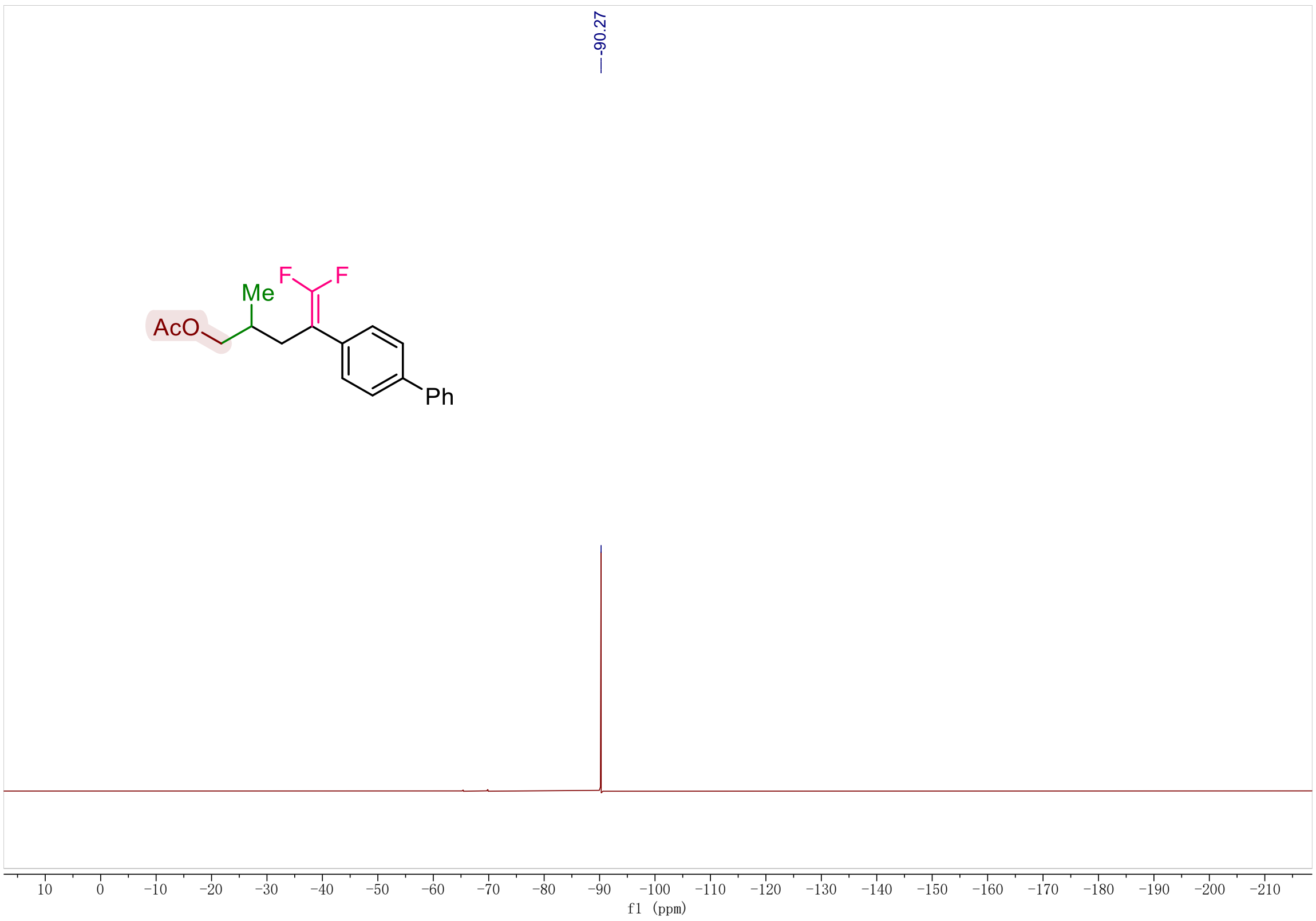

^{19}F NMR spectrum (377 MHz, Chloroform-*d*) of **20**

2-(2-([1,1'-Biphenyl]-4-yl)-3,3-difluoroallyl)-1-(phenylethynyl)cyclohexan-1-ol (Scheme 3, product 21 and 21')

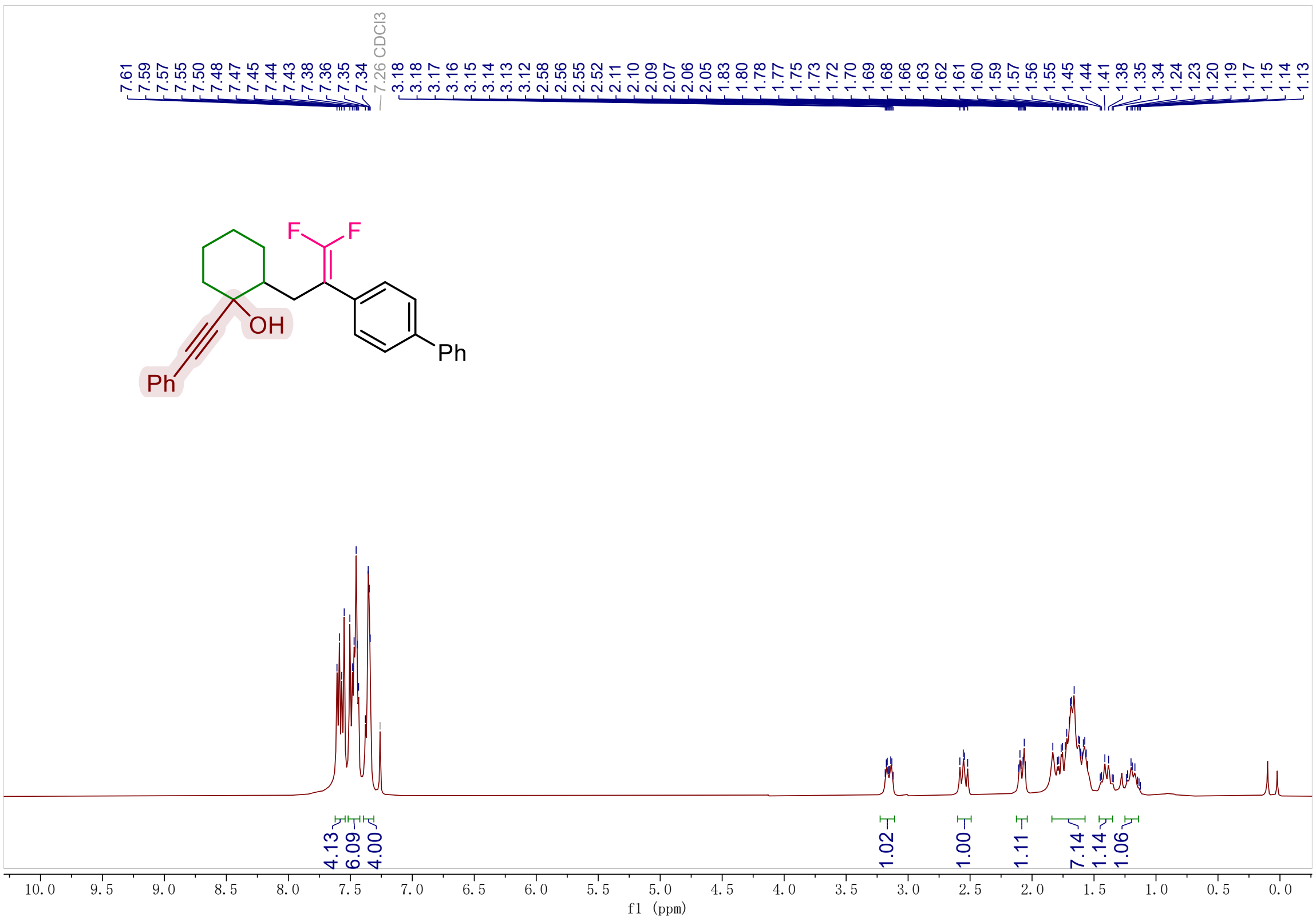

^{1}H NMR spectrum (400 MHz, Chloroform-*d*) of **21**

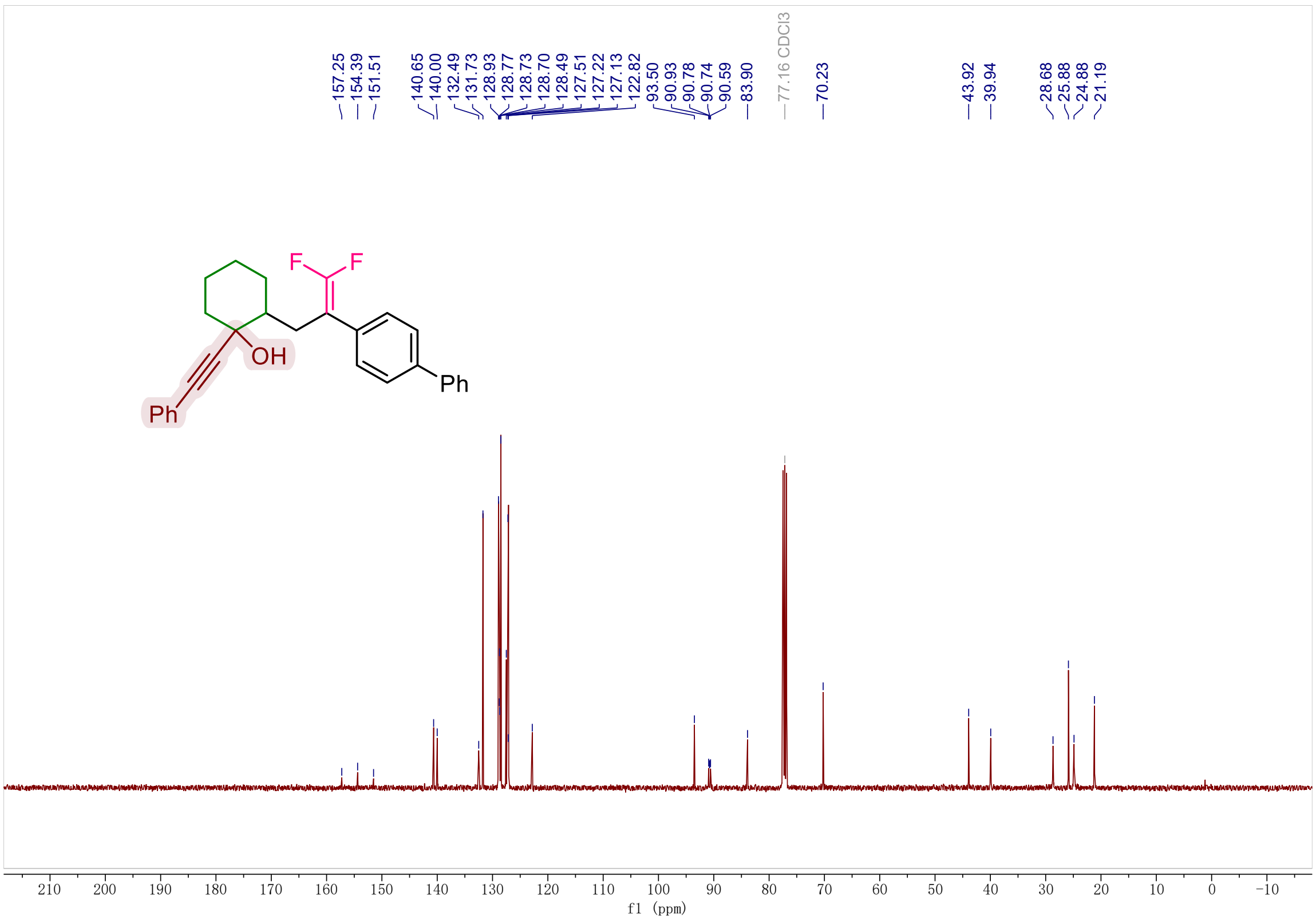

^{13}C NMR spectrum (100 MHz, Chloroform-*d*) of **21**

2-(2-([1,1'-Biphenyl]-4-yl)-3,3-difluoroallyl)-1-(phenylethynyl)cyclohexan-1-ol (Scheme 3, product 21 and 21')

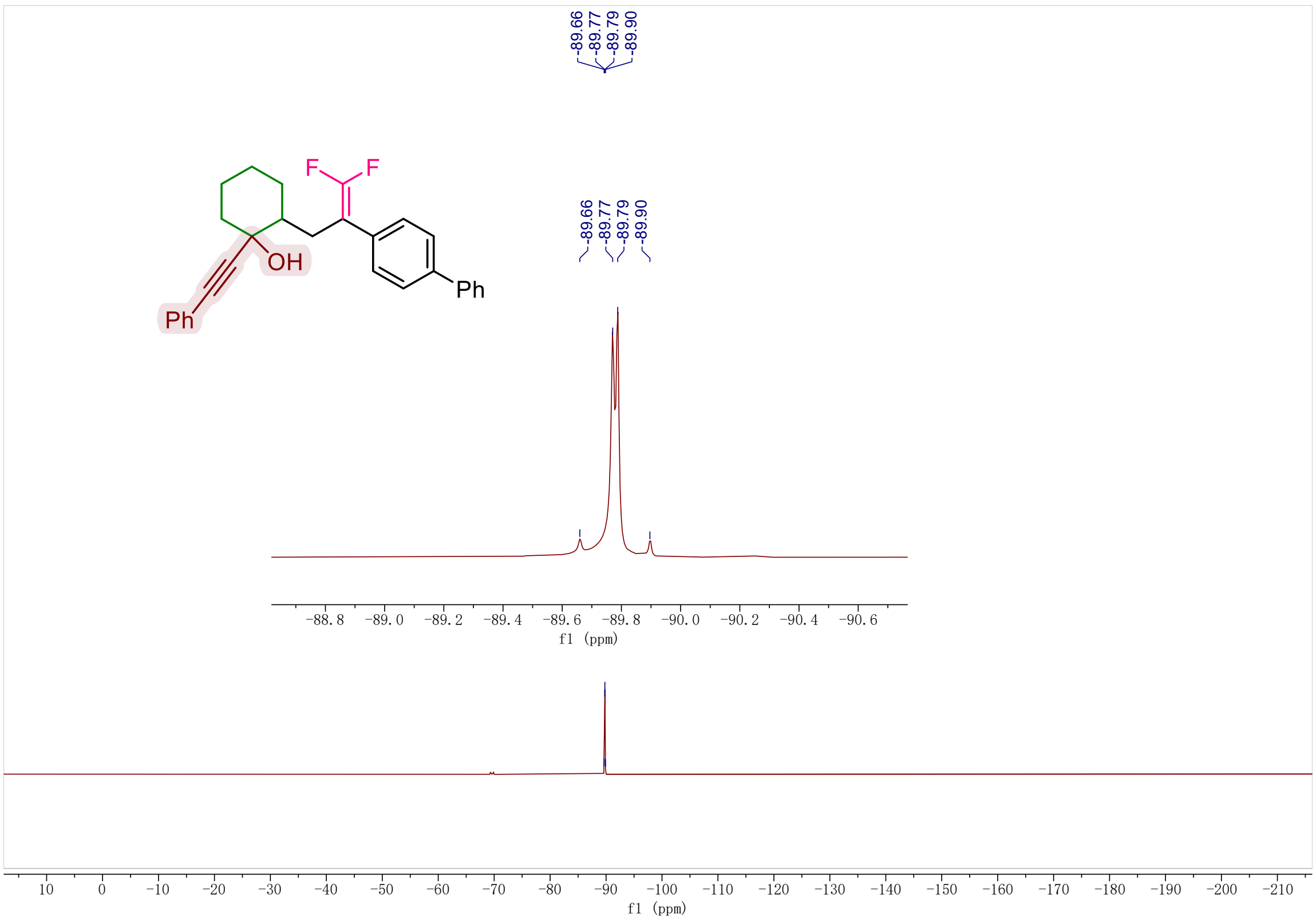

^{19}F NMR spectrum (377 MHz, Chloroform-*d*) of **21**

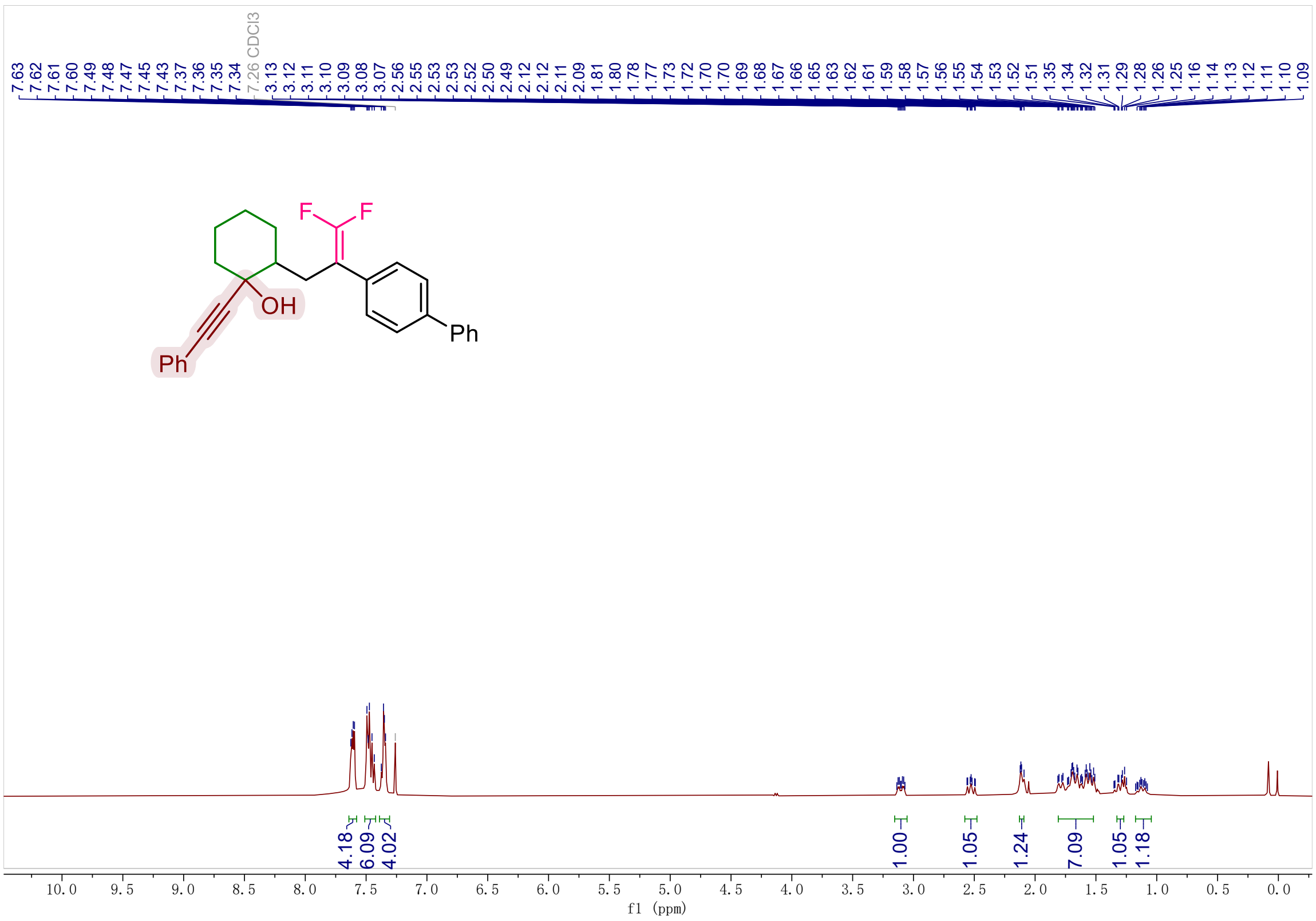

^{1}H NMR spectrum (400 MHz, Chloroform-*d*) of **21'**

2-(2-([1,1'-Biphenyl]-4-yl)-3,3-difluoroallyl)-1-(phenylethynyl)cyclohexan-1-ol (Scheme 3, product 21 and 21’)

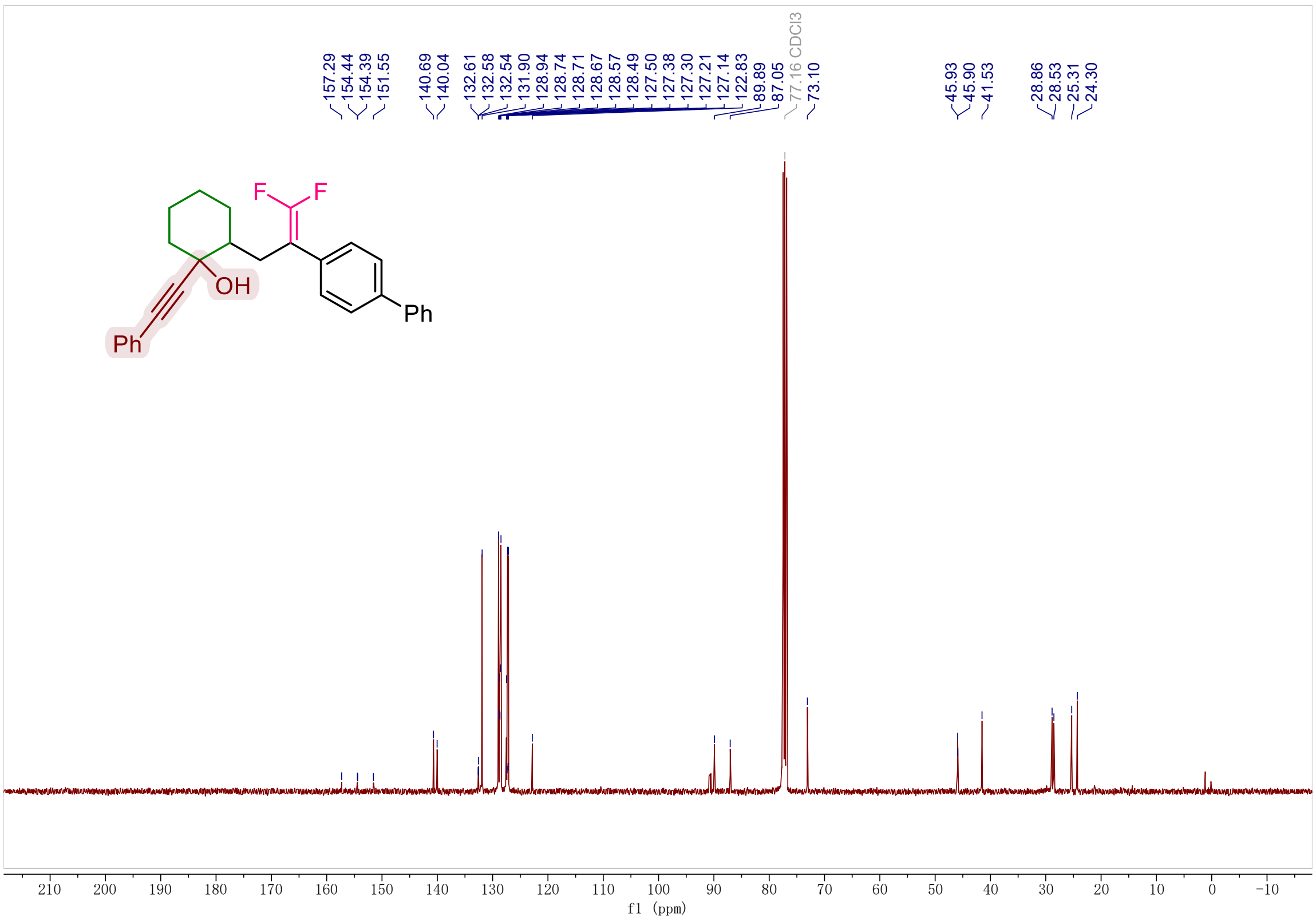

^{13}C NMR spectrum (100 MHz, Chloroform-*d*) of **21’**

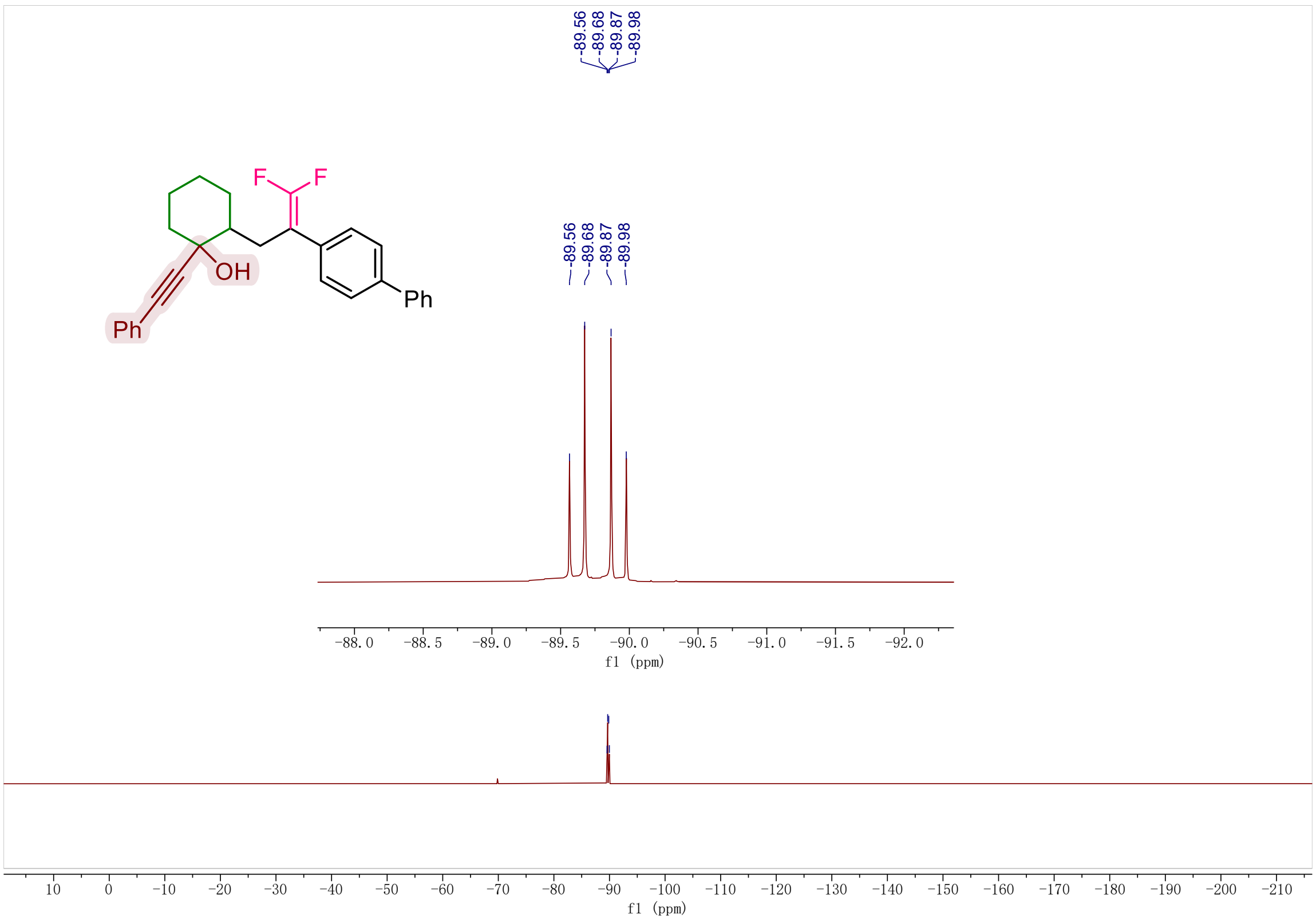

^{19}F NMR spectrum (377 MHz, Chloroform-*d*) of **21’**

4-(1,1-Difluoro-3-(2-methylenecyclohexyl)prop-1-en-2-yl)-1,1'-biphenyl (Scheme 3, product 22)

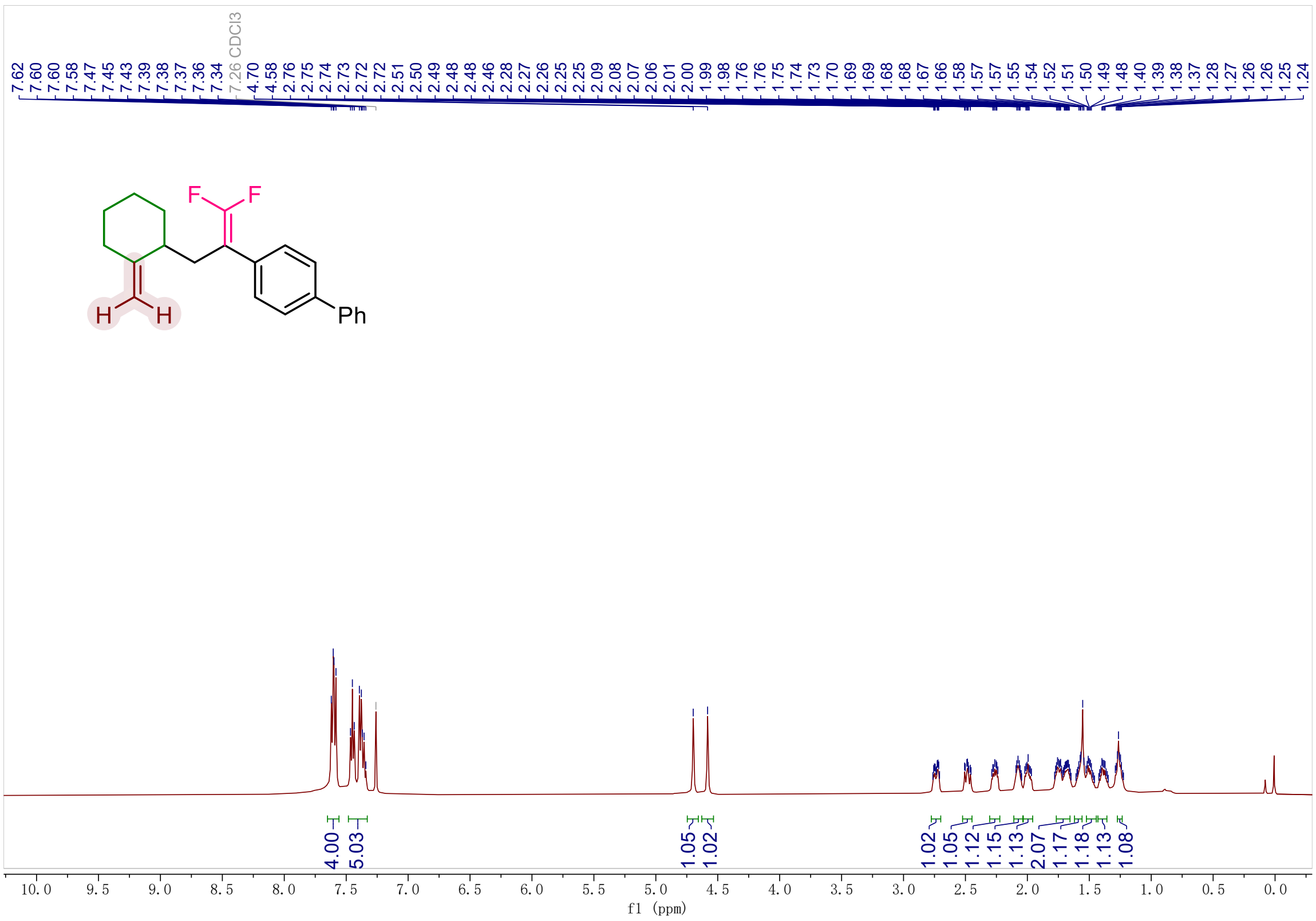

^{1}H NMR spectrum (500 MHz, Chloroform-*d*) of **22**

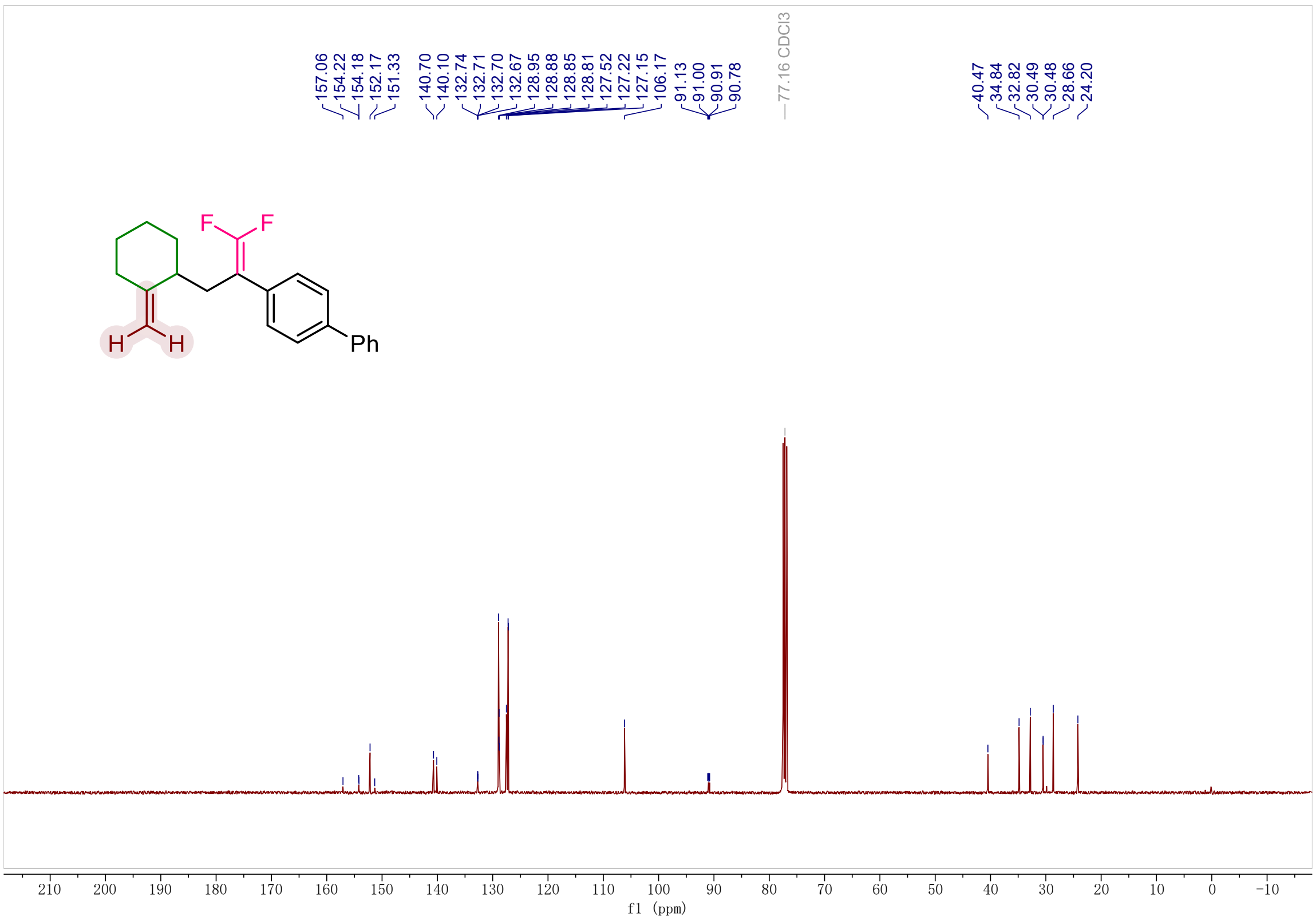

^{13}C NMR spectrum (100 MHz, Chloroform-*d*) of **22**

4-(1,1-Difluoro-3-(2-methylenecyclohexyl)prop-1-en-2-yl)-1,1'-biphenyl (Scheme 3, product 22)

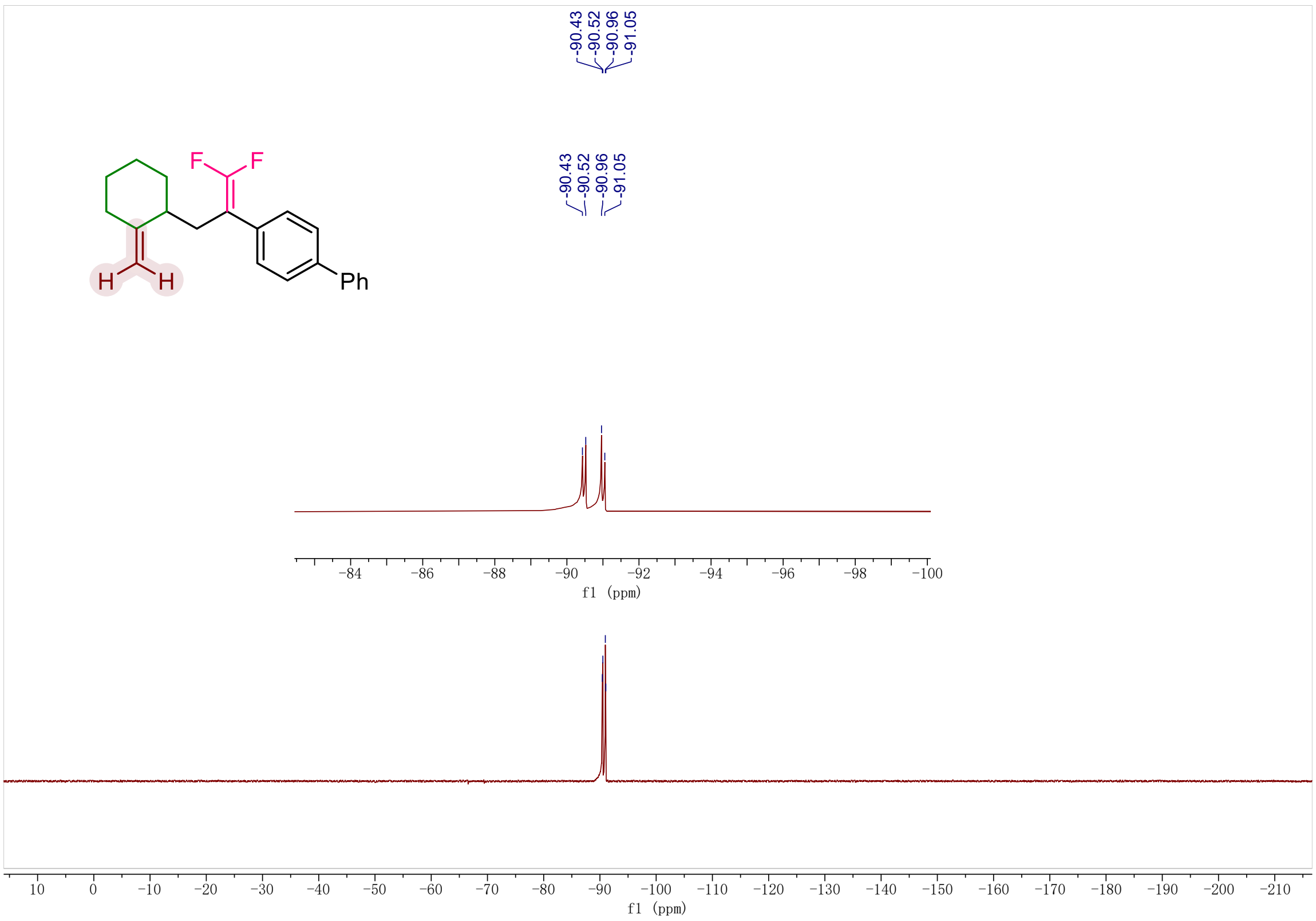

^{19}F NMR spectrum (471 MHz, Chloroform-*d*) of **22**

2-(2-([1,1'-Biphenyl]-4-yl)-3,3-difluoroallyl)cyclohexan-1-ol (Scheme 3, product 23 and 23')

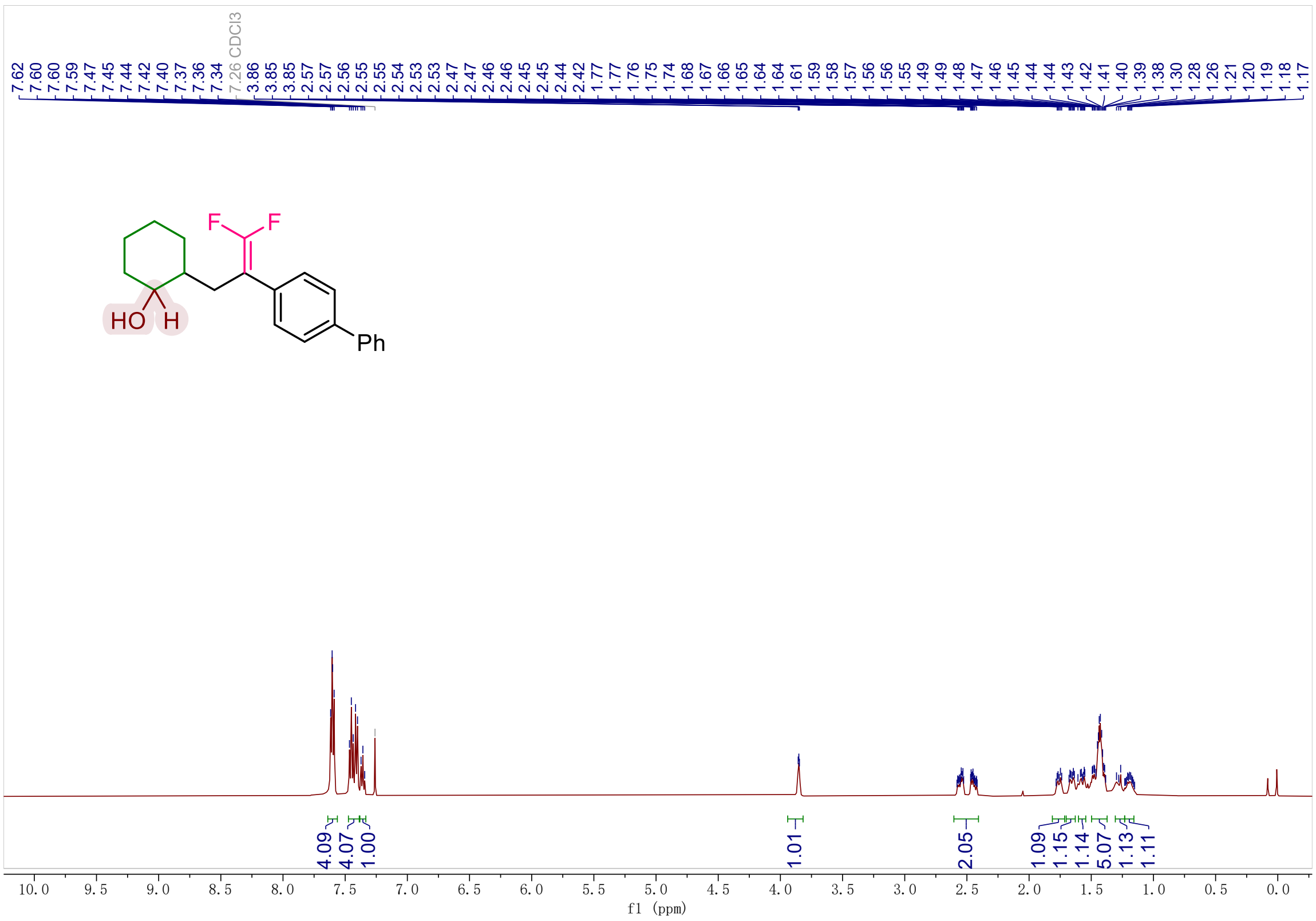

^{1}H NMR spectrum (500 MHz, Chloroform-*d*) of **23**

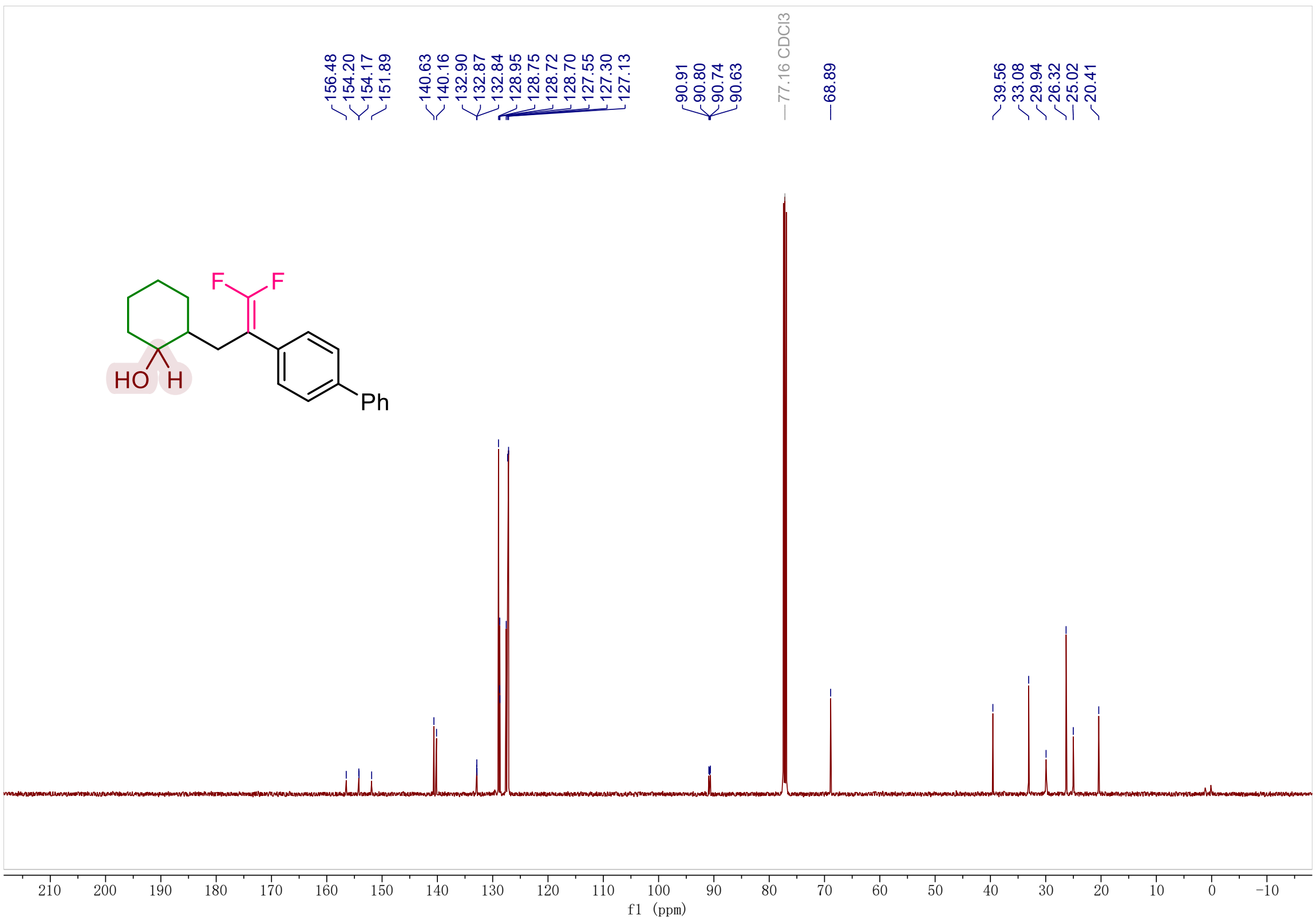

^{13}C NMR spectrum (125 MHz, Chloroform-*d*) of **23**

2-(2-([1,1'-Biphenyl]-4-yl)-3,3-difluoroallyl)cyclohexan-1-ol (Scheme 3, product 23 and 23')

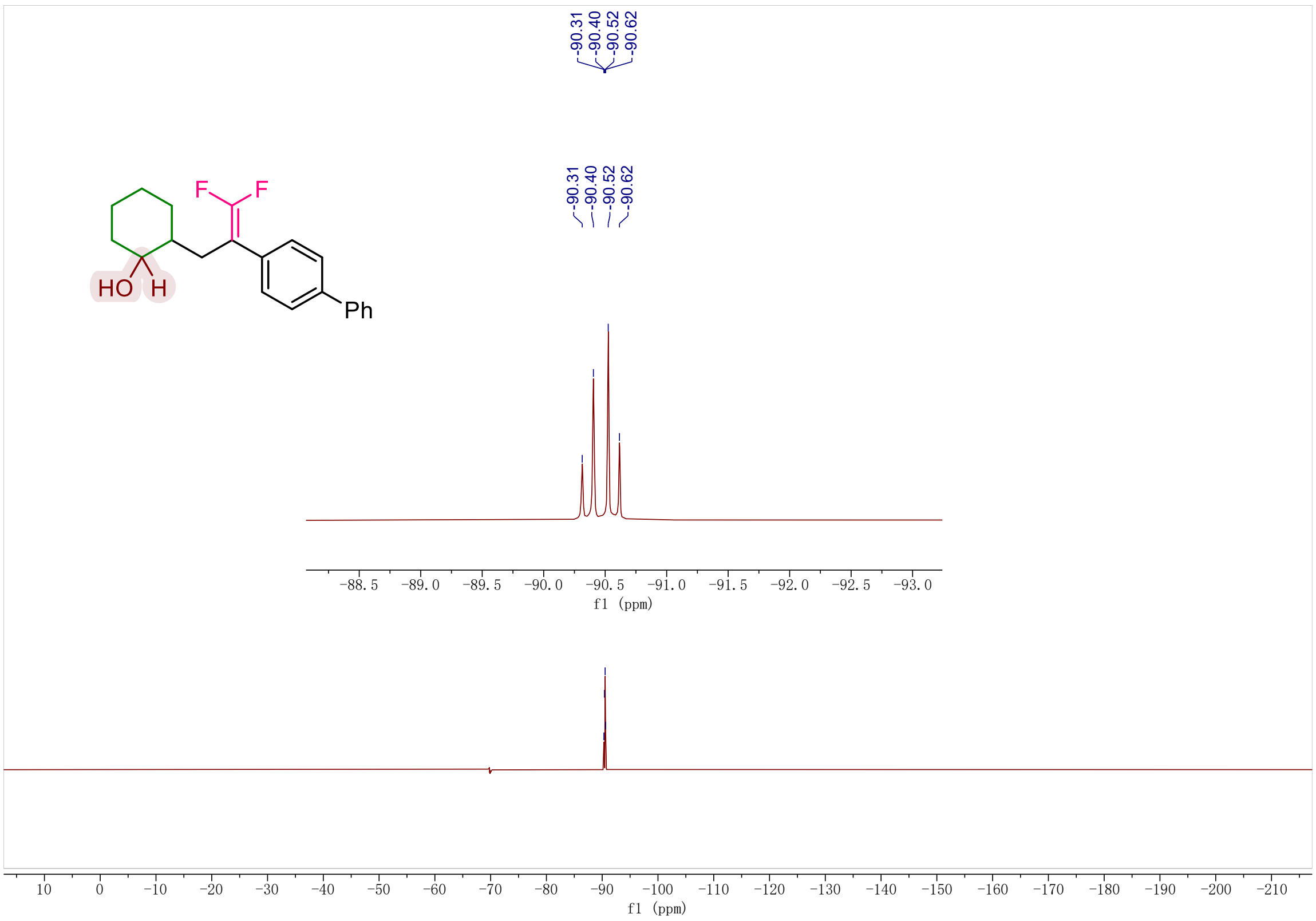

^{19}F NMR spectrum (471 MHz, Chloroform-*d*) of **23**

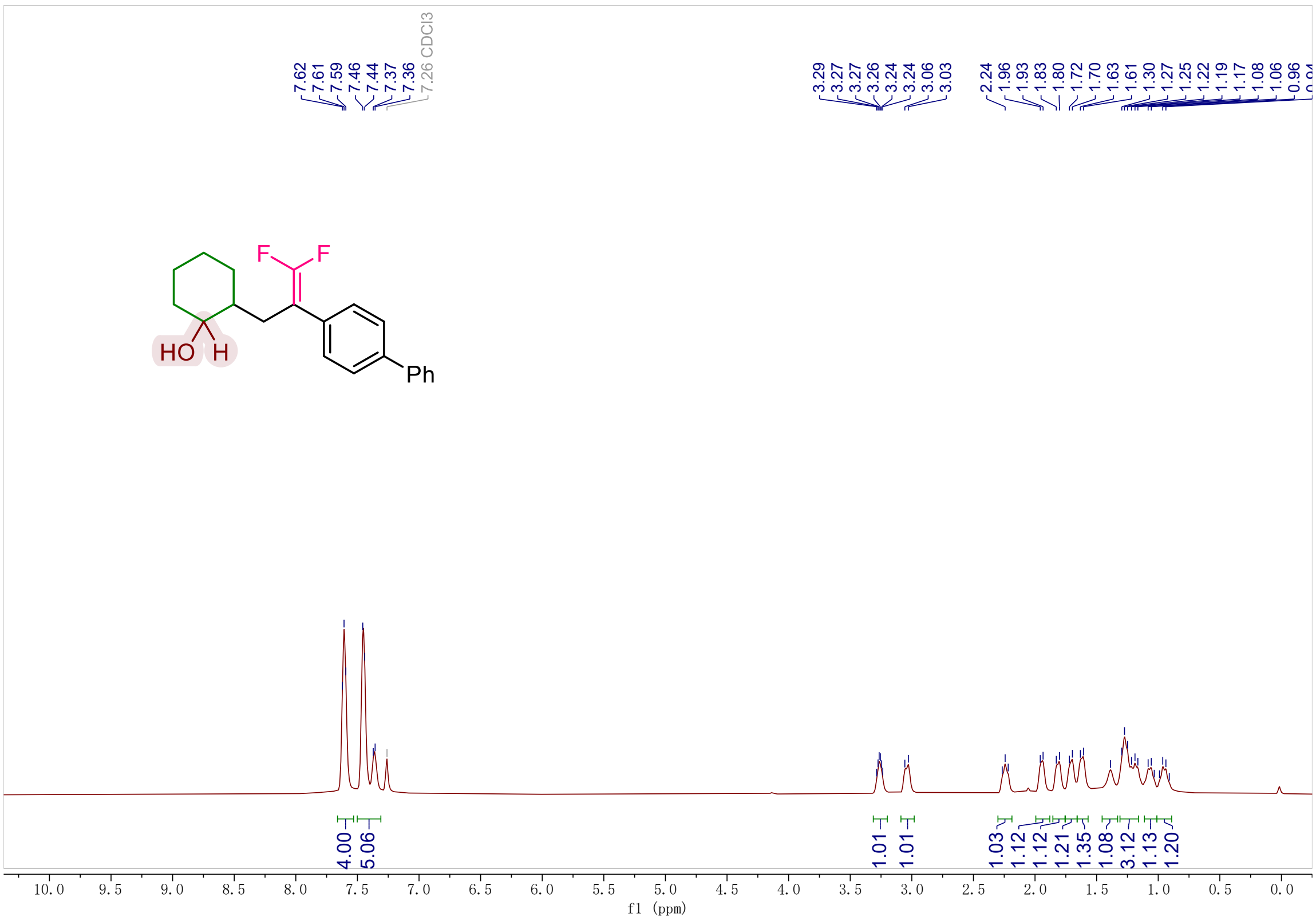

^{1}H NMR spectrum (500 MHz, Chloroform-*d*) of **23'**

2-(2-([1,1'-Biphenyl]-4-yl)-3,3-difluoroallyl)cyclohexan-1-ol (Scheme 3, product 23 and 23')

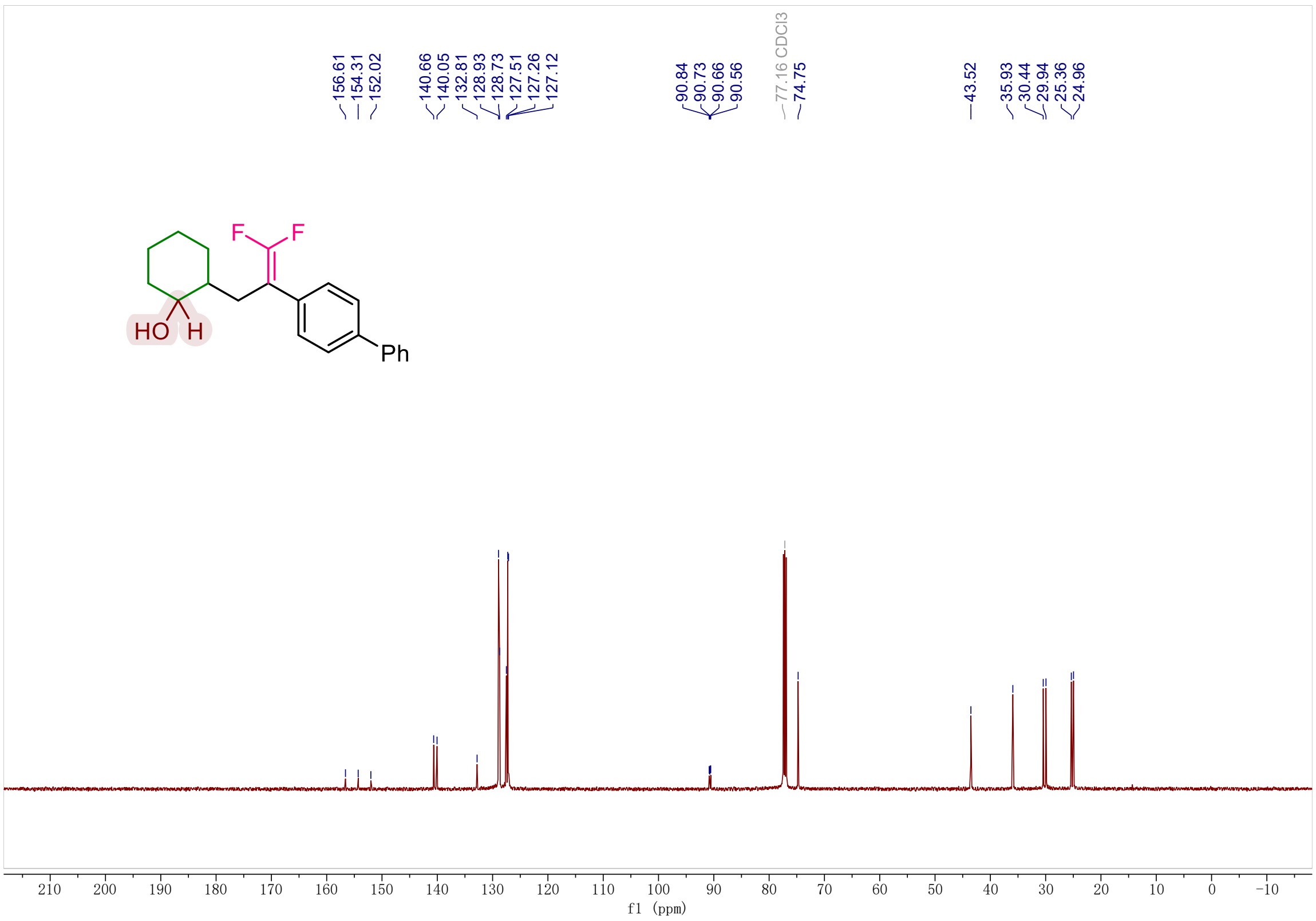

^{13}C NMR spectrum (125 MHz, Chloroform-*d*) of **23'**

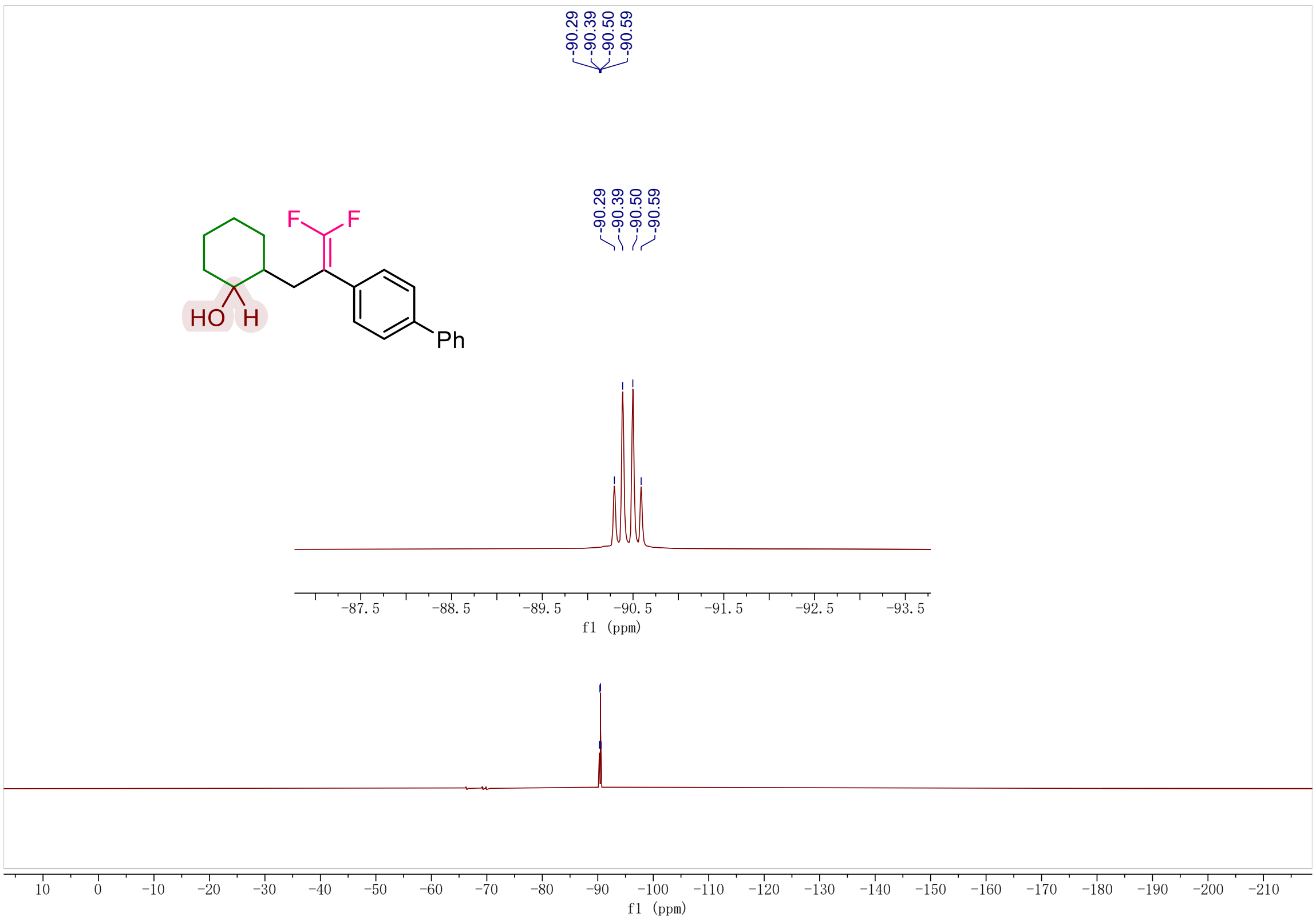

^{19}F NMR spectrum (471 MHz, Chloroform-*d*) of **23'**

3-([1,1'-Biphenyl]-4-yl)-2-fluoro-4a,5,6,7,8,8a-hexahydro-4*H*-chromene (Scheme 3, product 24)

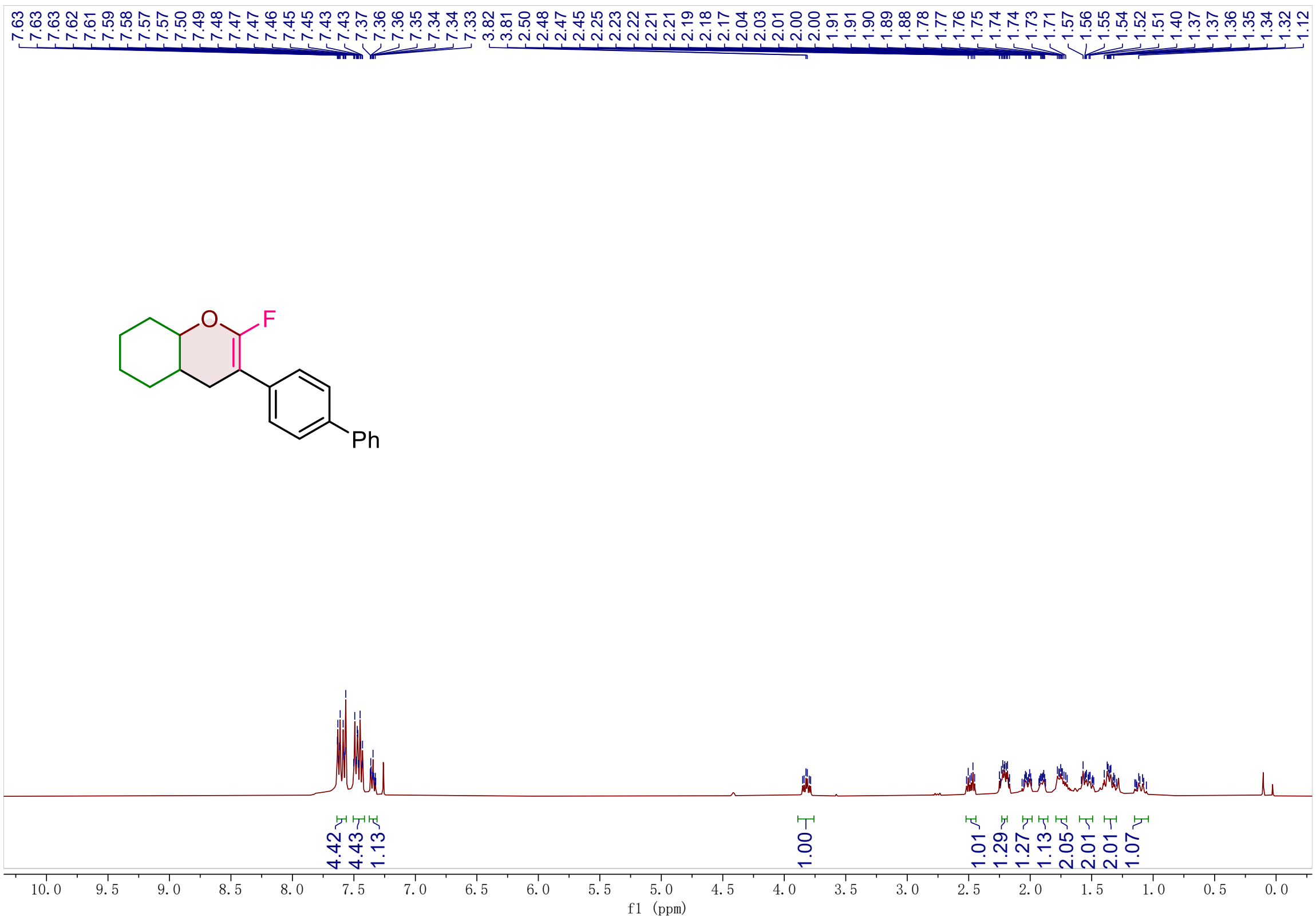

^{1}H NMR spectrum (400 MHz, Chloroform-*d*) of **24**

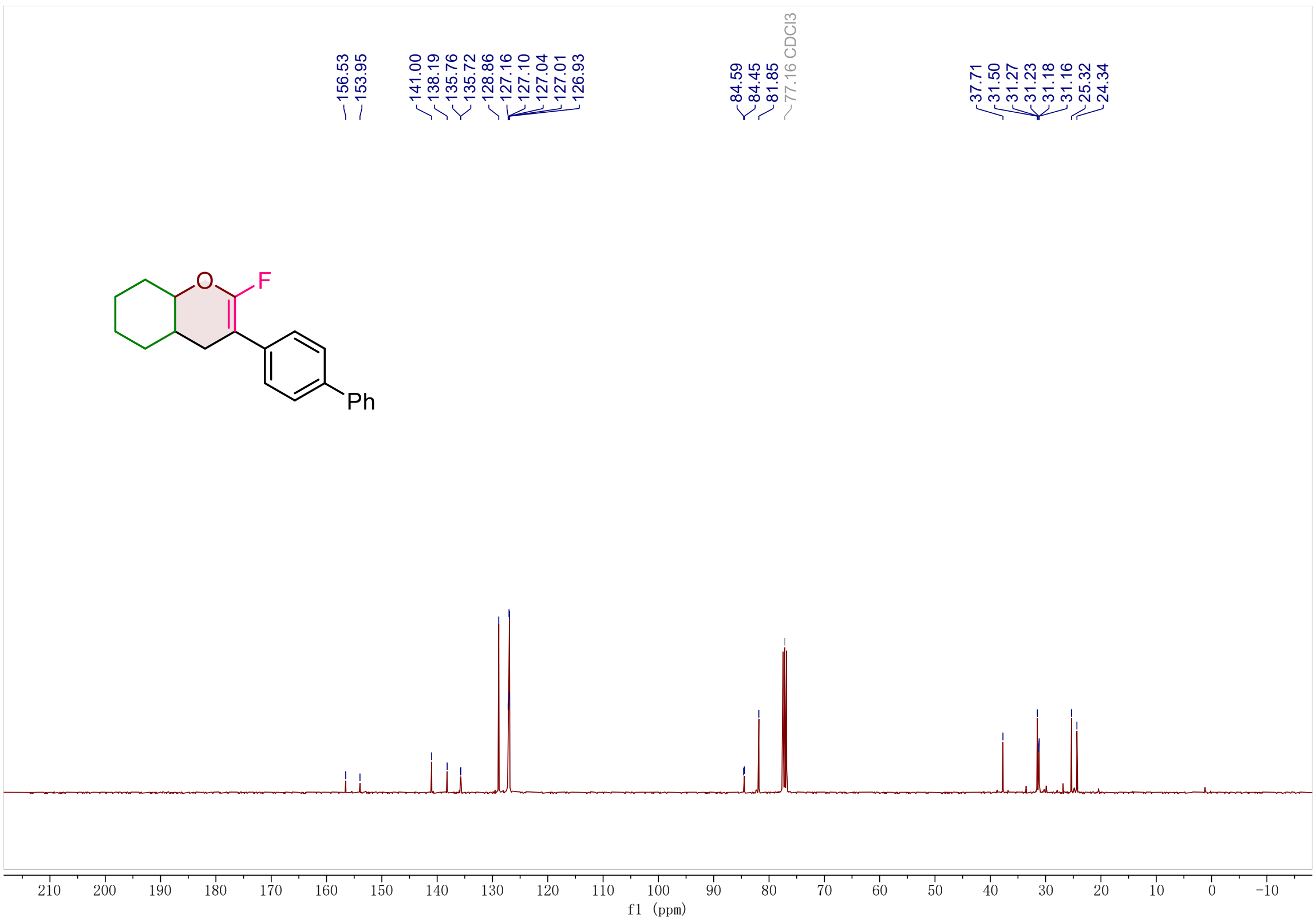

^{13}C NMR spectrum (100 MHz, Chloroform-*d*) of **24**

3-([1,1'-Biphenyl]-4-yl)-2-fluoro-4a,5,6,7,8,8a-hexahydro-4*H*-chromene (Scheme 3, product 24)

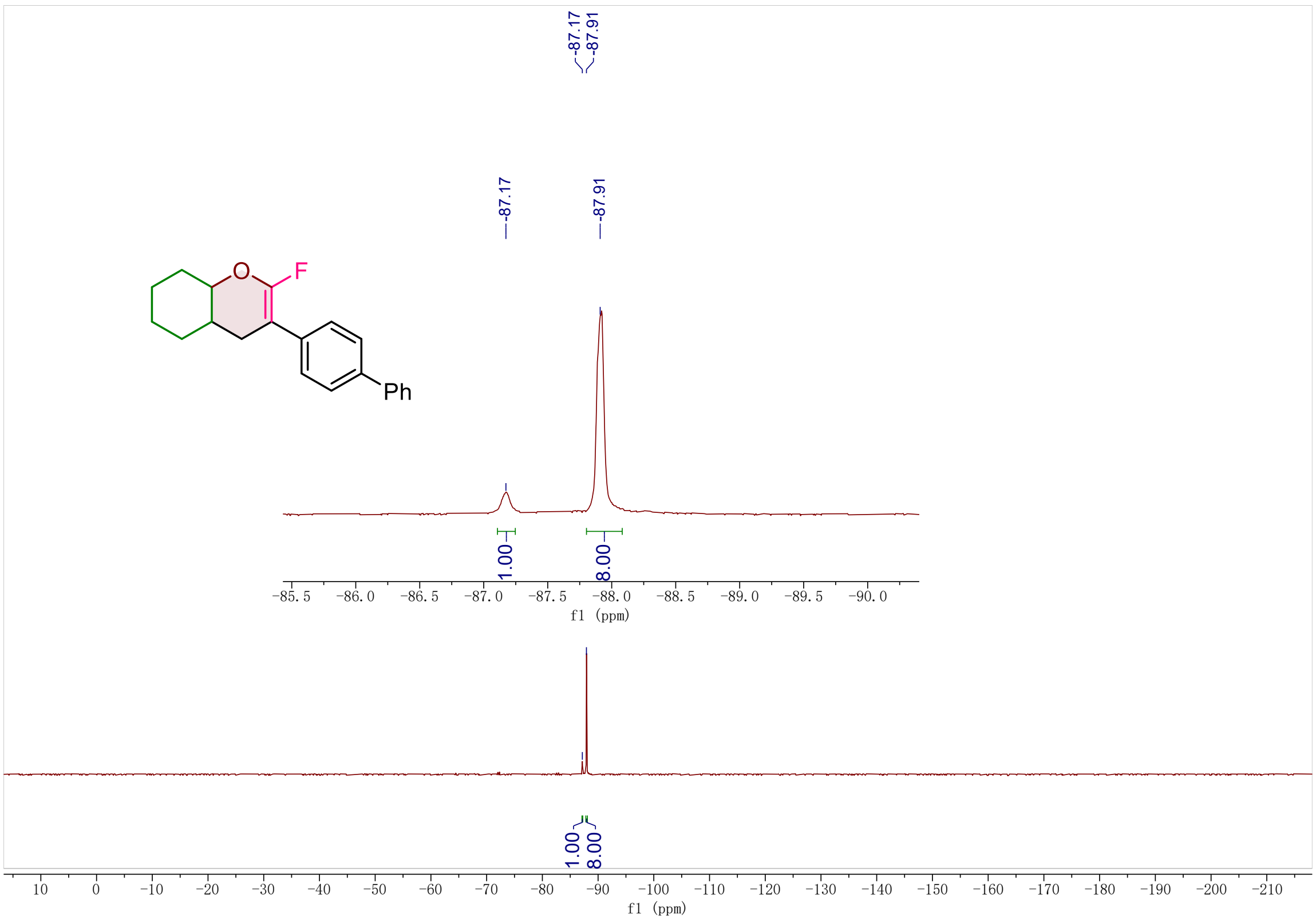

^{19}F NMR spectrum (471 MHz, Chloroform-*d*) of **24**

10. References

(1) Armarego, W. L. F.; Perrin, D. D. In Purification of Laboratory Chemicals, 4th, Ed. Butterworth-Heinemann: Oxford UK, 1996.

(2) Eckenhoff, W. T.; Biernesser, A. B.; Pintauer, T. Structural Characterization and Investigation of Iron(III) Complexes with Nitrogen and Phosphorus Based Ligands in Atom Transfer Radical Addition (ATRA). *Inorg. Chim. Acta.* **2012**, *382*, 84–95.

(3) Gao, Y.; Qin, W.; Tian, M.-Q.; Zhao, X.; Hu, X.-H. Defluorinative Alkylation of Trifluoromethyl Alkenes with Soft Carbon Nucleophiles Enabled by a Catalytic Amount of Base. *Adv. Synth. Catal.* **2022**, *364*, 2241–2247.

(4) Guan, Y.-Q.; Wang, T.-Z.; Qiao, J.-F.; Chen, Z.; Bai, Z.; Liang, Y.-F. Iron-Catalysed Reductive Coupling for the Synthesis of Polyfluorinated Compounds. *Chem. Commun.* **2022**, *58*, 13915–13918.

(5) Zhang, Z.; Zhang, Y.; Xie, X.; Liu, H.-W.; Zhu, T.; Zhang, J.-J.; Hu, M.-Y.; Chen, Z. Visible-Light-Induced Synergistic W/Cr Catalyzed *gem*-Difluoroallylation of Unactivated Alkanes. *Org. Lett.* **2025**, *27*, 2016–2021.

(6) Chen, B.; Chen, Q.; Liu, Y.; Chen, J.; Zhou, X.; Wang, H.; Yan, Q.; Wang, W.; Cai, Z.; Chen, F.-E. Visible-Light-Induced Defluorinative α-C(Sp3)–H Alkylation for the Synthesis of *gem*-Difluoroallylated α-Trifluoromethylamines. *Org. Lett.* **2023**, *25*, 9124–9129.

(7) Wang, A.-J.; Lou, Y.-G.; Zhao, L.; Gou, F.-H.; Wang, L.; Yu, Y.; Gu, J.; He, C.-Y. Palladium-Catalyzed Cross-Coupling of Benzylzinc Reagents with 2-Bromo-3,3,3-Trifluoropropene. *Synth. Commun.* **2019**, *49*, 3329–3334.

(8) Qiu, C.; Liu, L.; Zhang, K.; Du, S.; Chen, Y.; Wang, X.; Gong, H. A Unified Approach to Chiral α-Aryl Ketones and Aldehydes via Ni-Catalyzed Asymmetric Reductive Cross-Coupling. *J. Am. Chem. Soc.* **2025**, *147*, 17251–17259.

(9) Chen, H.; Jia, X.; Yu, Y.; Qian, Q.; Gong, H. Nickel-Catalyzed Reductive Allylation of Tertiary Alkyl Halides with Allylic Carbonates. *Angew. Chem. Int. Ed.* **2017**, *56*, 13103.

(10) (a) Ma, T.; Li, X.; Ping, Y.; Kong, W. Synthesis of *gem*-Difluoroalkenes via Ni-Catalyzed Three-Component Defluorinative Reductive Cross-Coupling of Organohalides, Alkenes and Trifluoromethyl Alkenes. *Chin.J.Chem.* **2022**, *40*, 2212–2218. (b) Xing, W.-L.; Wang, J.-X.; Fu, M.-C.; Fu, Y. Efficient Decarboxylative/Defluorinative Alkylation for the Synthesis of *gem*-Difluoroalkenes through an S_N2'-Type Route. *Chin.J.Chem.* **2022**, *40*, 323–328. (c) Xiong, Y.; Zhang, X.; Huang, T.; Cao, S. Synthesis of *N*-(α-Fluorovinyl)azoles by the Reaction of Difluoroalkenes with Azoles. *J. Org. Chem.* **2014**, *79*, 6395–6402.
